# Development of an Enantioselective Allylic Alkylation of Acyclic *α*‐Fluoro‐*β*‐ketoesters for Asymmetric Synthesis of 3‐Fluoropiperidines

**DOI:** 10.1002/chem.202201595

**Published:** 2022-08-10

**Authors:** Jiaxin Han, Larry Hoteite, Joseph P. A. Harrity

**Affiliations:** ^1^ Department of Chemistry The University of Sheffield Sheffield S3 7HF UK

**Keywords:** catalysis, enantioselectivity, functionalization, palladium, piperidine

## Abstract

The first useful enantioselective Pd‐catalyzed asymmetric allylic alkylation of *α*‐fluoro‐*β*‐ketoesters has been achieved using the Trost family of chiral ligands yielding products in up to 92 % ee. This work provides new insights regarding the typically modest selectivities associated with acyclic *α*‐fluoroenolates and shows experimental evidence that the typically poor levels of enantiocontrol associated with these systems are not necessarily due to the presence of *E/Z* enolate mixtures. Finally, this methodology allows the easy preparation of useful 3‐fluoropiperidine intermediates, and it is demonstrated that these systems are applicable to a range of functionalization reactions leading to new building blocks for the discovery of bioactive products.

## Graphical abstract

**Get into Gear**: The Trost ligand series promotes the enantioselective allylation of *α*‐fluoro‐*β*‐ketoesters to generate 3‐fluoropiperidines in up to 92 % ee. We have employed the Lloyd‐Jones Norrby model to explain the origins of enantiocontrol and propose that the catalyst backbone engenders a gearing effect whereby the ester adopts an *s‐trans* conformation that enforces the alkene moiety of the enoate to adopt an *s‐cis* orientation, thereby controlling facial to the π‐allyl complex.

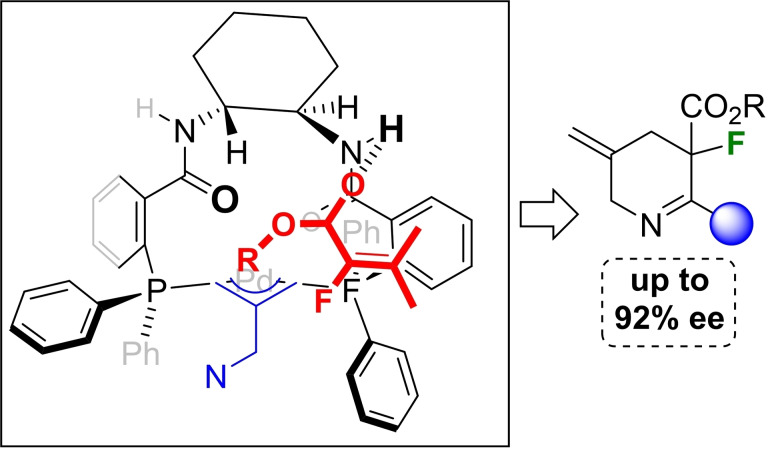

## Introduction

**Figure 1:**
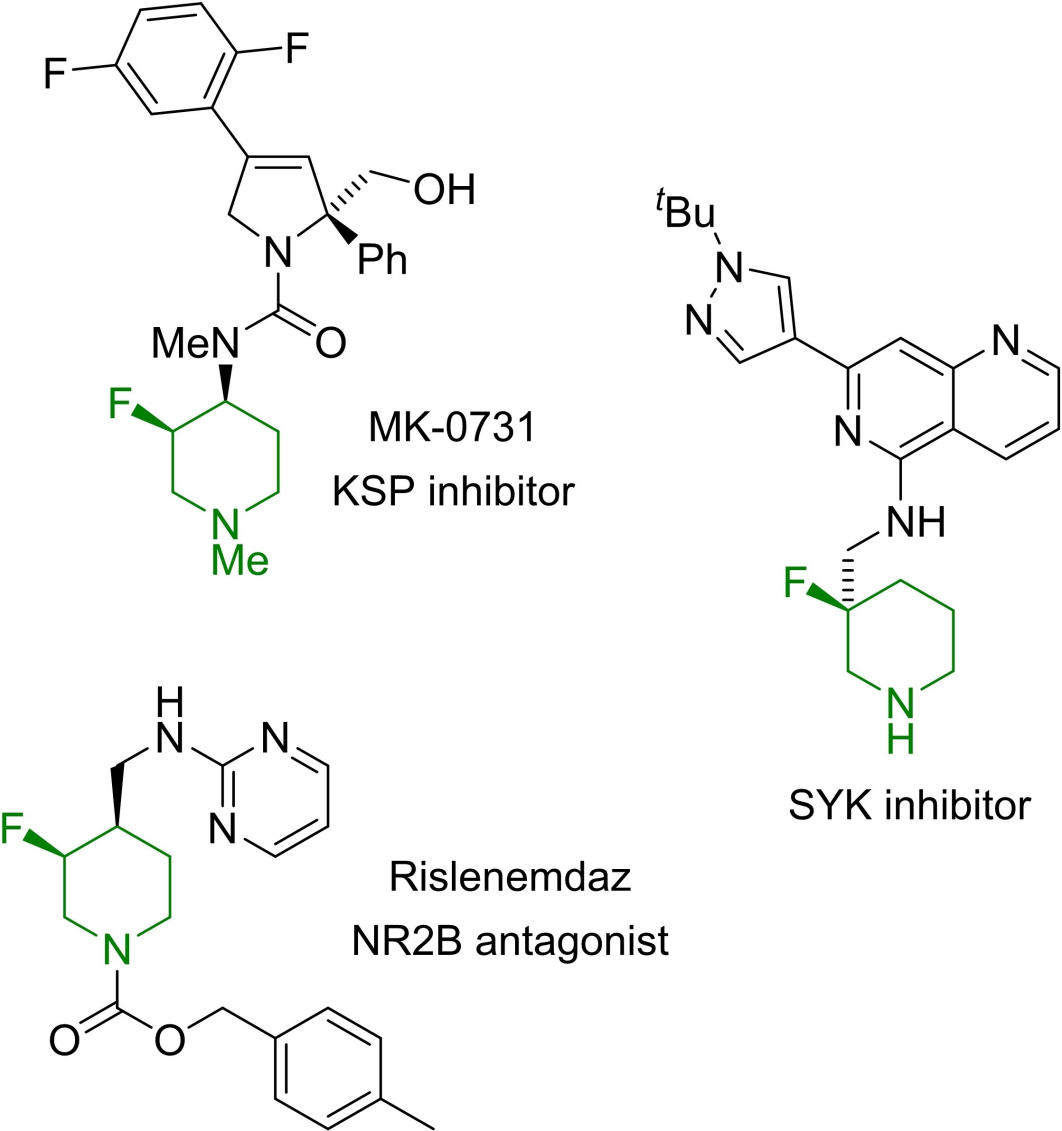
Bioactive compounds featuring 3‐fluoropiperidine fragments.

Piperidines are ubiquitous in natural products and are amongst the most prominent family of heterocycles in FDA approved drugs.^[1]^ Within this particular class, 3‐fluorinated piperidines have emerged as common motifs in small molecule pharmaceuticals (representative examples are shown in Figure 1) as the incorporation of fluorine offers enhanced metabolic stability while attenuating basicity that can have an important impact in the overall compound properties (eg ADME profile and hERG liability).^[2]^

Despite the importance of 3‐fluoropiperidine derivatives, relatively few methods for their synthesis exist^[3]^ and enantioselective variants are even rarer. The current best approaches for the synthesis of enantioenriched 3‐fluoropiperidines include intramolecular aminofluorination of olefins, however, these methods often require extensive pre‐functionalization and super‐stoichiometric quantities of high molecular weight oxidants, leading to low atom economy.^[4]^

We recently reported a Pd‐catalyzed allylation‐condensation sequence that delivered functionalized 3‐fluoropiperidine derivatives in high yield.^[5]^ An especially valuable aspect of this approach is the exploitation of *α*‐fluoro‐*β*‐ketoesters as substrates because these can be prepared in turn from inexpensive ethyl fluoroacetate,^[6]^ which offers a convenient and inexpensive source of the key fluorine atom thereby avoiding costly and hazardous electrophilic fluorinating agents. We reasoned that the use of a chiral ligand in the allylation step could allow this process to deliver these products with enantiocontrol. In this context, Stoltz and Nakamura pioneered the enantioselective Pd‐catalyzed decarboxylative allylic alkylation of *α*‐fluoro‐*β*‐ketoesters,^[7]^ and further powerful iterations of this idea were reported in subsequent years.[^8^, ^9^] However, while this approach delivers excellent levels of enantiocontrol for cyclic keto esters, the corresponding acyclic systems show only modest levels of enantioselectivity with typically <55 % ee, an observation that has been ascribed to the presence of enolate *E/Z* mixtures.

**Figure 2:**
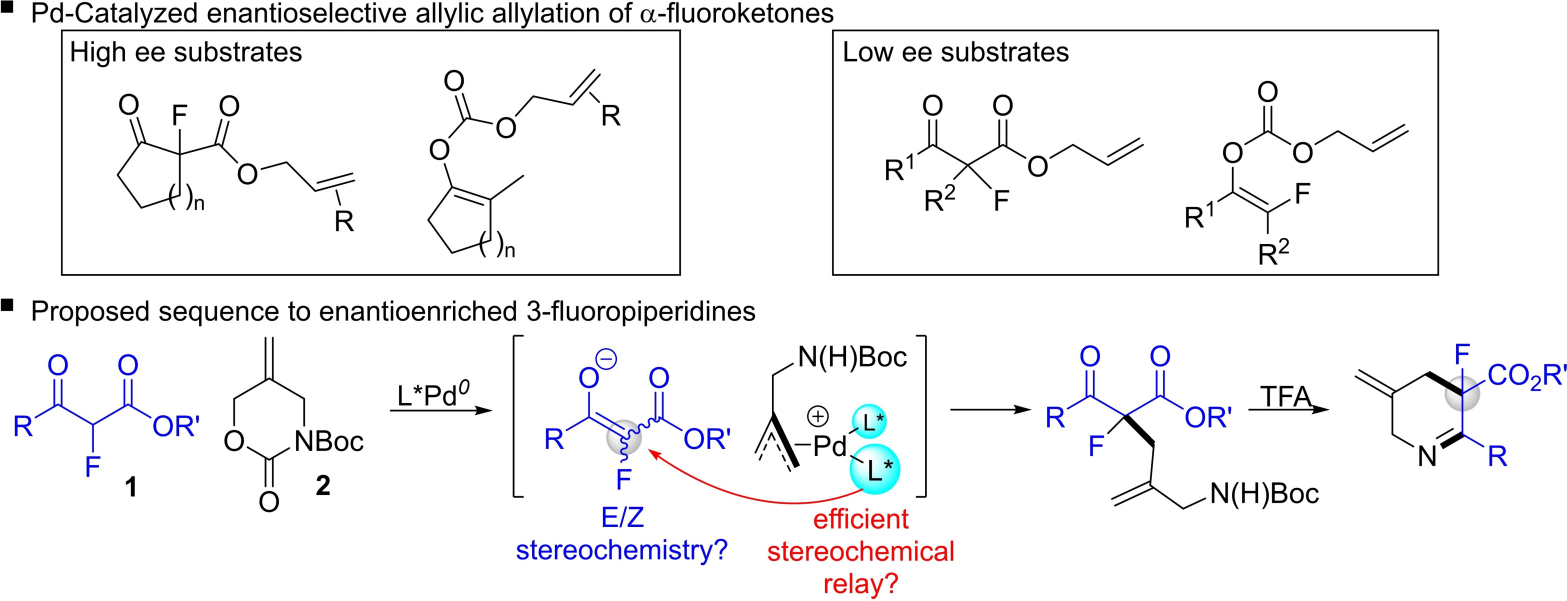
Pd‐catalyzed asymmetric allylic alkylation of *α*‐fluoroketone derivatives.

Adapting a Pd‐catalyzed asymmetric allylation^[10]^ strategy to our system raises several challenging issues that must be overcome to deliver an enantioselective route to 3‐fluoropiperidines: (1) our piperidine forming strategy requires the employment of acyclic *α*‐fluoro‐*β*‐ketoesters, and so the potential for enantiodivergence in *E/Z* enolate mixtures must be overcome; (2) the enolate functions as the prochiral fragment making the efficient relay of stereochemistry from chiral catalyst to prochiral substrate difficult (assuming an outer sphere mechanism^[11]^).^[12]^ With these challenges in mind, we set out to investigate if the Pd‐catalyzed allylic alkylation of **1** was viable for the generation of 3‐fluoropiperidine derivatives with useful levels of enantiocontrol (Figure 2).

## Results and Discussion

We began our studies by screening chiral phosphine ligands that are commonly employed in asymmetric allylation reactions, and selected results are highlighted in Table 1. The allylation of **1 a** with **2** proceeded in high yield in NMP (*N*‐methyl‐2‐pyrrolidone) and so this solvent was employed in our preliminary efforts to identify promising ligand classes. In the event, phosphoramidites such as **L1** all gave very poor selectivities (<10 % ee^[13]^) and PHOX ((*S*)‐4‐*tert*‐butyl‐2‐[2‐(diphenylphosphino)phenyl]‐2‐oxazoline) ligand **L2**, which has been used successfully in the synthesis of cyclic *α*‐fluoroketones,[^7^, ^8^, ^11^] was unselective under these conditions. Switching to the Trost ligand family^[14]^ was more encouraging and ligands **L3**, **L4** and **L6** provided our first promising enantioselectivities. Conducting a solvent screen with **L6** identified ether solvents optimal for enantioselectivity, but these led to low conversion of **1 a** to **3 a**. We envisaged that conversion could be increased by generating some of the enolate derived from **1 a** and so introduced triethylamine. While this had only a modest effect on conversion with ether solvents, the use of toluene delivered the product with useful conversion and an increased enantioselectivity. The use of *t*BuOH^[15]^ further increased ee levels and employing these conditions with **L4** and **L3** highlighted the latter as providing the best balance of conversion and selectivity. In an effort to avoid potential oligomeric Pd/ligand species^[16]^ we reduced the equivalents of **L3** and this in combination with Hünig's base provided optimal conversion and ee.

**Table 1:** Chiral ligand screening and optimization.

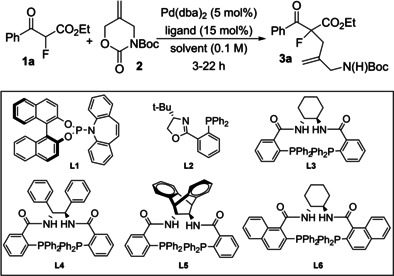						
Entry	Ligand	Additives	Solvent	T, time	Yield	ee
1	**L1**	–	NMP	rt, 3 h	70 %	−8 %
2	**L2**	–	NMP	rt, 3 h	73 %	−10 %
3	**L3**	–	NMP	rt, 3 h	70 %	34 %
4	**L4**	–	NMP	rt, 3 h	99 %	41 %
5	**L5**	–	NMP	rt, 3 h	79 %	25 %
6	**L6**	–	NMP	rt, 3 h	99 %	48 %
7	**L6**	–	CH_2_Cl_2_	rt, 3 h	75 %	42 %
8	**L6**	–	THF	rt, 22 h	20 %	62 %
9	**L6**	–	Dioxane	rt, 22 h	10 %	60 %
10	**L6**	–	PhMe	rt, 22 h	<5 %	–
11	**L6**	Et_3_N^[a]^	THF	rt, 22 h	30 %	62 %
12	**L6**	Et_3_N^[a]^	Dioxane	rt, 22 h	15 %	60 %
13	**L6**	Et_3_N^[a]^	PhMe	rt, 22 h	42 %	65 %
14	**L6**	Et_3_N, ^*t*^BuOH^[b]^	PhMe	rt, 22 h	38 %	70 %
15	**L4**	Et_3_N, ^*t*^BuOH^[b]^	PhMe	rt, 22 h	10 %	57 %
16	**L3**	Et_3_N, ^*t*^BuOH^[b]^	PhMe	rt, 22 h	78 %	56 %
17	**L3**	Et_3_N, ^*t*^BuOH^[b]^	PhMe	0 °C, 22 h	73 %	73 %
18	**L3** ^[c]^	Et_3_N, ^*t*^BuOH^[b]^	PhMe	0 °C, 22 h	42 %	78 %
19	**L3** ^[c]^	^i^Pr_2_NEt, ^*t*^BuOH^[b]^	PhMe	0 °C, 22 h	78 %	75 %

[a] 1.2 equiv. of Et_3_N was used. [b] 1.2 equiv. base and 5 equiv. of *t*BuOH were used. [c] 5.5 mol% of **L3** used in this case.

**Figure 3:**
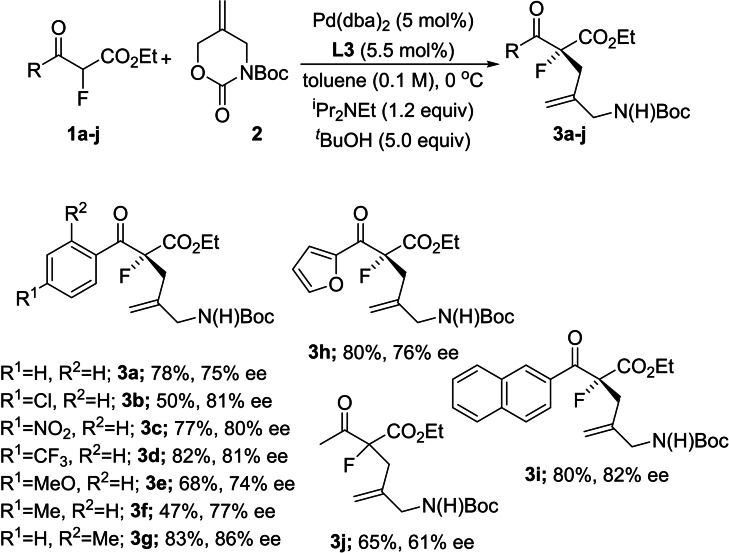
Scope of the asymmetric allylic alkylation reaction.

Next we investigated the scope of the enantioselective allylic alkylation of *α*‐fluoro‐*β*‐ketoesters under optimal conditions. Our results are summarized in Figure 3. Substrates bearing a range of electron withdrawing and donating groups gave similar levels of enantiocontrol (75–81 % ee) with the *ortho*‐tolyl derived substrate providing an enhanced ee of 86 % in the case of **3 g**. These selectivities were mirrored in the cases of furan and 2‐naphthyl‐substituted products **3 h** and **3 i**, but alkyl ketone derivative **1 j** was found to deliver the allylated product **3 j** with only modest levels of enantiocontrol.

**Figure 4:**
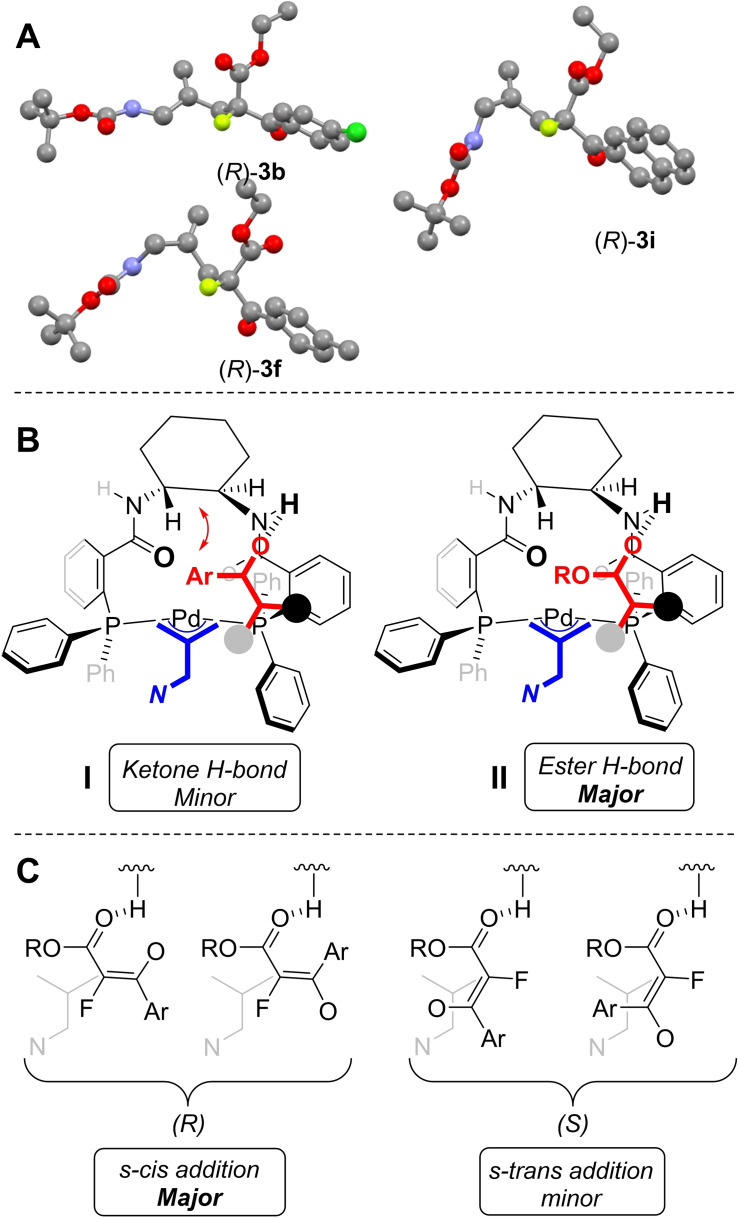
Proposed origin of enantiocontrol using the Lloyd‐Jones and Norrby model.

To understand the origin of enantiocontrol, we set out to establish the absolute stereochemistry of the major enantiomers. Pleasingly, recrystallization of compounds **3 b,f,i** from toluene/CH_2_Cl_2_ mixtures delivered highly enantiomerically enriched products (>95 % ee) and X‐ray crystal structure analysis showed them to exhibit the (*R*)‐configuration in all cases (Figure 4, panel A). The configuration of the major enantiomers of **3 a–i** are assigned as (*R*) by inference.^[17]^ Detailed mechanistic studies by Lloyd‐Jones and Norrby has provided a model that offers a rationale for enantioselectivity allylation reactions mediated by the Trost ligand set^[18]^ which is becoming widely adopted.^[19]^ As shown in Figure 4, panel B, we have attempted to use this model as a working hypothesis to explain the enantioselectivity of the formation of compounds **3**. If the reactive π‐allyl complex adopts the *endo*‐rotameric form with the amidomethyl moiety pointing away from the catalyst backbone cyclohexane, either the ketone (**I**) or ester (**II**) oxygen atoms can form hydrogen bonds to the amide N−H. However, coordination of the ketone would place the aromatic ring close to the catalyst backbone leading to an unfavorable steric interaction. Assuming that coordination to the catalyst takes place via the ester, this leads to an *E/Z*‐enolate pair that adopts a *s‐cis* conformation around the enoate fragment leading to the observed (*R*)‐enantiomer product, with the minor enantiomer arising from the corresponding *s‐trans* conformer (Figure 4, panel C).

**Scheme 1:**
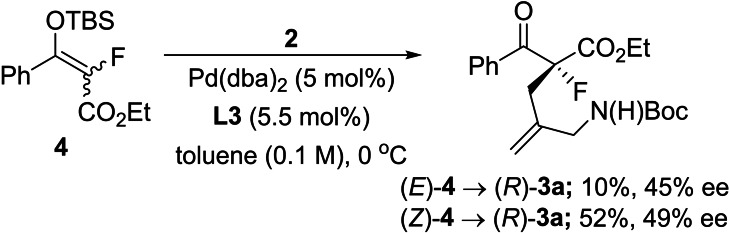
Reaction of *E/Z*‐silyl enol ether **4**.

In order to probe this issue further, we prepared silyl enol ethers *E*‐ and *Z*‐**4** and subjected these to **2** in the presence of Pd‐catalyst and **L3**. As shown in Scheme 1, both enol ether isomers converged to the (*R*)‐enantiomer of **3 a** with similar levels of enantiocontrol, albeit with quite different levels of conversion. This hypothesis therefore suggests that, in the case of the asymmetric allylic alkylation of using the Trost ligand series, the presence of *E/Z*‐enolate or enol mixtures does not necessarily result in enantiodivergence.

**Figure 5:**
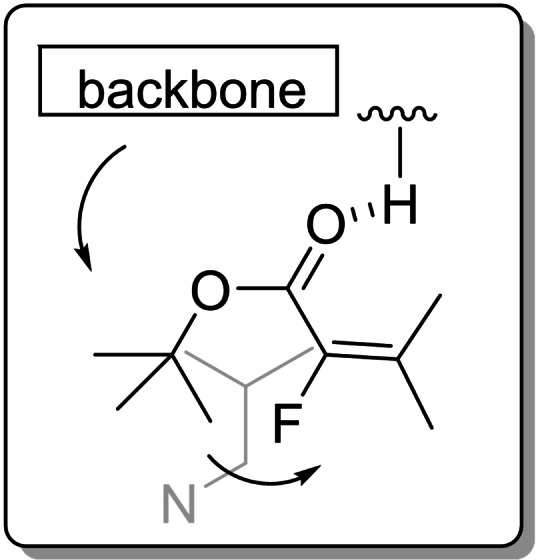
Encouraging *s‐cis* enoate conformation through an ester gearing effect.

Returning to the models in Figure 4, panel C, we wondered whether we could adapt the ester group in order to encourage a greater proportion of *s‐cis* conformer. The ester would be ideal for this purpose as it would not limit the scope of the chemistry in subsequent transformations. In this regard, we proposed that a bulky ester may undergo a steric clash with the catalyst backbone enforcing the ester *s‐trans* conformation that, in turn, would encourage the alkene moiety of the enoate to adopt an *s‐cis* orientation which would lead to better enantiocontrol in the allylic alkylation step (Figure 5).

**Figure 6:**
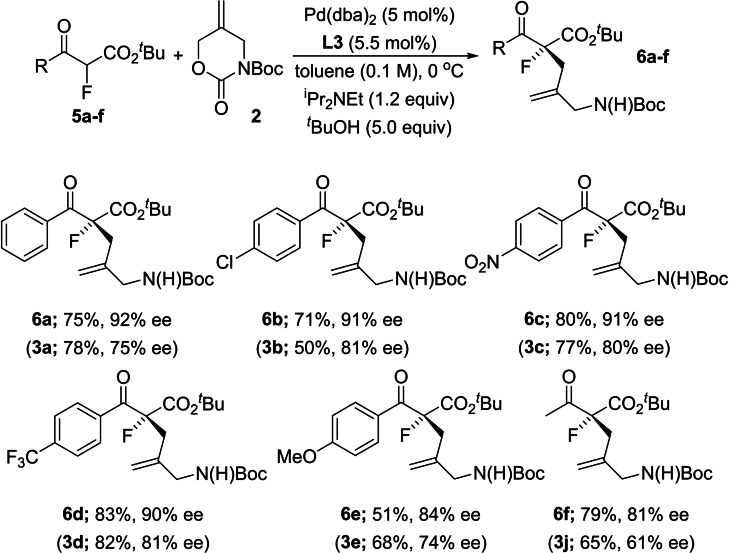
Improved enantioselectivity using *tert*‐butyl esters.

We prepared a small series of *tert*‐butyl esters and investigated their performance in the enantioselective allylic alkylation reaction under optimal conditions and our results are summarized in Figure 6. In general, higher enantioselectivities were indeed observed across the board when *tert*‐butyl esters **5 a–f** were employed, as compared to their ethyl ester analogs. The enantioselectivity observed in the case of **6 f** is especially pleasing as these substrates are typically formed in <50 % ee. Notably, converting an enantioenriched sample of **3 a** to **6 a** (KO*t*Bu, *t*BuOH, reflux) confirmed that the major enantiomer in each case had the same configuration, ruling out a switch in facial selectivity.

**Figure 7:**
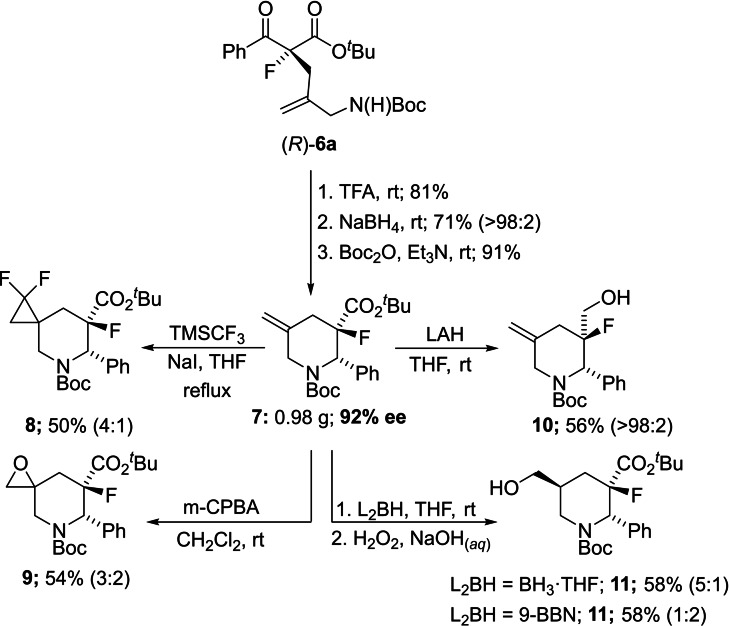
Synthesis of functionalized 3‐fluoropiperidines.

Finally, in order to confirm the suitability of this method for the stereocontrolled synthesis of 3‐fluoropiperidines we generated **6 a** on a gram scale and found that this could be smoothly converted to the functionalized piperidine **7** in high yield and with complete diasterecontrol (Figure 7). The stereochemistry of **7** was assigned on the basis of the known stereochemistry of the corresponding ethyl ester.^[5]^ In addition, compound **7** provided a platform to demonstrate the potential of these intermediates to be elaborated chemoselectively to enantioenriched building blocks.^[20]^ Specifically, the exocyclic olefin provided a convenient handle to generate a spiro‐fused difluorocyclopropane moiety in **8**, a motif that has recently gathered prominence in drug discovery.^[21]^ The alkene was also readily epoxidized to generate **9**, albeit with low diastereocontrol. Compound **7** also provided significant scope for installing a hydroxymethyl group with versatility in respect to position on the heterocycle and stereochemistry. For example, reduction of the ester generated **10** whereas hydroboration delivered the complementary substitution pattern in **11**, with borane and 9‐BBN (9‐borabicyclo[3.3.1]nonane) showing contrasting diastereoselectivities.^[22]^

## Conclusion

We have developed the first highly enantioselective allylic alkylation of acyclic *α*‐fluoro‐*β*‐ketoesters, a challenging class of substrates for this kind of transformation. In addition, we provide experimental evidence that the typically poor levels of enantiocontrol associated with these systems are not necessarily due to the presence of *E/Z* enolate mixtures. We propose an alternative hypothesis that relates to *s‐cis/trans* conformational mobility. This methodology enables the preparation of useful 3‐fluoropiperidine intermediates. We show that these systems can be applied to a range of functionalization reactions leading to progress in the discovery of bioactive compounds.

Deposition Numbers 2170247 (for **3 b**), 2170248 (for **3 f**), 2170249 (for **3 i**) contain the supplementary crystallographic data for this paper. These data are provided free of charge by the joint Cambridge Crystallographic Data Centre and Fachinformationszentrum Karlsruhe Access Structures service.

## Experimental Section

Gram scale synthesis of (*R*)‐7: A flame‐dried 250 mL round bottom flask was charged with Pd(dba)_2_ (144 mg, 5 mol%), (*R,R*)‐Dach‐phenyl Trost ligand L3 (190 mg, 5.5 mol%), *t*‐BuOH (2.4 mL, 25 mmol), DIPEA (1.0 mL, 6 mmol) and *tert*‐butyl 5‐methylene‐2‐oxo‐1,3‐oxazinane‐3‐carboxylate 2 (1.06 g, 5 mmol) under nitrogen. Anhydrous PhMe (25 mL) was then added, and the mixture stirred at 0 °C for 20 minutes. A solution of *tert*‐butyl 2‐fluoro‐3‐oxo‐3‐phenylpropanoate (5a) (1.54 g, 6.5 mmol) in PhMe (25 mL) was then added and the reaction stirred at 0 °C overnight. Afterwards the reaction was concentrated under vacuum and purified by flash column chromatography (15 % diethyl ether in petroleum ether) to provide (*R*)‐6a which was used directly in the next step. ^1^H NMR (400 MHz, CDCl_3_) δ 8.02 (d, *J*=8.0 Hz, 2H), 7.58 (t, *J*=7.5 Hz, 1H), 7.44 (t, *J*=8.0 Hz, 2H), 5.15 (s, 1H), 5.05 (s, 1H), 4.79 (br, 1H), 3.78 (br, 2H), 3.09 (dd, *J*=34.0, 15.5 Hz, 1H), 2.94 (dd, *J*=18.0, 15.5 Hz, 1H), 1.44 (s, 9H), 1.36 (s, 9H); ^13^C NMR (101 MHz, CDCl_3_) *δ* 191.4 (d, *J*=26.0 Hz), 165.8 (d, *J*=25.5 Hz), 156.9, 139.9, 133.9, 133.7 (d, *J*=7.5 Hz), 129.8 (d, *J*=4.0 Hz), 128.7, 116.1, 99.4 (d, *J*=200.0 Hz), 84.6, 79.5, 45.7, 38.1 (d, *J*=21.0 Hz), 28.5, 27.9; ^19^F NMR (377 MHz, CDCl_3_): *δ* −156.2 (dd, *J*=33.8, 18.0 Hz). FTIR: *ν*_max_/cm^−1^ (neat) 2979, 2929, 1707, 1512, 1422, 1366, 1223, 1155, 1059, 908, 839, 697; HRMS (ESI^+^): calculated for C_22_H_30_FNO_5_Na (ES^+^)(+Na^+^): 430.2006. Found: 430.2009. HPLC (Cellulose‐1, hexane: *i*PrOH 99.5:0.5, flow rate 1.0 mL/min, λ=254 nm, 25 °C) t_R_(major)=32.000 min, t_R_(minor)=34.937 min, ee=92 %.

(*R*)‐6a was dissolved in CH_2_Cl_2_ (86 mL) and TFA (16.4 mL, 50 equiv.) was added to the mixture. After stirring at room temperature for 30 minutes, the mixture was basified to pH 8 using sat. NaHCO_3_ before the extraction with CH_2_Cl_2_. The combined organic layers were then dried over anhydrous magnesium sulfate and concentrated under vacuum to afford *tert*‐butyl 3‐fluoro‐5‐methylene‐2‐phenyl‐3,4,5,6‐tetrahydropyridine‐3‐carboxylate as a yellow oil (1.00 g, 71 % yield over two steps). ^1^H NMR (400 MHz, CDCl_3_) *δ* 7.71 ‐ 7.65 (m, 2H), 7.42 ‐ 7.31 (m, 3H), 5.08 (s, 1H), 5.01 (s, 1H), 4.63 (dd, *J*=20.5, 5.5 Hz, 1H), 4.50 (dd, *J*=20.5, 5.5 Hz, 1H), 2.95 ‐ 2.78 (m, 2H), 1.25 (s, 9H). ^13^C NMR (101 MHz, CDCl_3_) *δ* 168.0 (d, *J*=27.0 Hz), 161.0 (d, *J*=18.5 Hz), 137.4, 136.9 (d, *J*=3.5 Hz), 130.0, 128.4, 127.4 (d, *J*=2.5 Hz), 112.4, 90.6 (d, *J*=195.0 Hz), 84.0, 56.1, 39.5 (d, *J*=24.1 Hz), 27.8; ^19^F NMR (377 MHz, CDCl_3_): *δ* −146.0 – −146.2 (m) FTIR: *ν*_max_/cm^−1^ (neat) 2978, 1749, 1636, 1447, 1369, 1321, 1260, 1076, 733, 704 cm^−1^; HRMS (ESI^+^): calculated for: C_17_H_21_FNO_2_ (ES^+^)(+H^+^): 290.1556. Found: 290.1569.

To a solution of *tert*‐butyl 3‐fluoro‐5‐methylene‐2‐phenyl‐3,4,5,6‐tetrahydropyridine‐3‐carboxylate (1.00 g, 3.46 mmol) in MeOH (17.3 mL) under nitrogen at 0 °C NaBH_4_ (523 mg, 13.84 mmol) was added and the resulting mixture warmed to room temperature and stirred overnight. The reaction was then diluted with NaHCO_3_ and extracted with EtOAc. The combined organic layers were dried over anhydrous MgSO_4_, concentrated under vacuum and the residue purified by flash column chromatography (20 % EtOAc in petroleum ether) to afford *tert*‐butyl (2*S*,3*R*)‐3‐fluoro‐5‐methylene‐2‐phenylpiperidine‐3‐carboxylate as a yellow oil that was used directly in the next step. To a solution of *tert*‐butyl 3‐fluoro‐5‐methylene‐2‐phenylpiperidine‐3‐carboxylate (0.845 g, 1.12 mmol) in THF (17.0 mL) under nitrogen Et_3_N (0.90 mL, 6.37 mmol) and di‐*tert*‐butyl dicarbonate (1.39 g, 6.38 mmol) were added and the resulting mixture stirred at room temperature overnight. The reaction was then diluted with H_2_O and extracted with DCM. The combined organic layers were then dried over anhydrous MgSO_4_, concentrated under vacuum and purified by FCC (4 % EtOAc in 40–60 petroleum ether) to afford di‐*tert*‐butyl (2*S*,3*R*)‐3‐fluoro‐5‐methylene‐2‐phenylpiperidine‐1,3‐dicarboxylate (7) as a colourless oil (0.978 g, 65 % yield over two steps). ^1^H NMR (400 MHz, CDCl_3_) δ 7.41 ‐ 7.12 (m, 5H), 5.42 (br, 1H), 5.07 (s, 1H), 4.95 (s, 1H), 4.43 (br, 1H), 3.82 (br, 1H), 3.12 (dd, *J*=43.0 16.0 Hz, 1H), 2.76 (br, 1H), 1.34 (s, 9H), 1.17 (s, 9H); ^13^C NMR (101 MHz, CDCl_3_) *δ* 167.1 (d, *J*=24.0 Hz), 154.9, 141.1, 137.0, 128.7, 128.5, 128.2, 113.9, 94.8 (d, *J*=185.5 Hz), 83.2, 80.5, 61.3, 45.7, 34.8 (d, *J*=88.0 Hz), 28.4, 27.5; ^19^F NMR (377 MHz, CDCl_3_) *δ* −144.6 (br), −145.6 (br); FTIR: *ν*_max_/cm^−1^ (neat) 2977, 2932, 1740, 1694, 1455, 1392, 1367, 1284, 1251, 1156, 1106, 1061, 972, 894, 839, 765, 700; HRMS (ESI+): calculated for C_22_H_30_FNO_4_Na (ES+)(+Na^+^): 414.2057. Found: 414.2079.

## Conflict of interest

The authors declare no conflict of interest.

1

## Supporting information

As a service to our authors and readers, this journal provides supporting information supplied by the authors. Such materials are peer reviewed and may be re‐organized for online delivery, but are not copy‐edited or typeset. Technical support issues arising from supporting information (other than missing files) should be addressed to the authors.

Supporting Information

## Data Availability

The data that support the findings of this study are available in the supplementary material of this article.
