# Supplementary material for: Development of an Enantioselective Allylic Alkylation of Acyclic α‐Fluoro‐β‐ketoesters for Asymmetric Synthesis of 3‐Fluoropiperidines
Source: Chemistry. 2022 Aug 10;28(56):e202201595. doi: 10.1002/chem.202201595 (PMC9804466; doi:10.1002/chem.202201595)
Supplement: Supplementary file 1 — Supporting Information [file CHEM-28-0-s001.pdf]

Chemistry–A European Journal

Supporting Information

Development of an Enantioselective Allylic Alkylation of Acyclic α-Fluoro-β-ketoesters for Asymmetric Synthesis of 3-Fluoropiperidines

Jiaxin Han, Larry Hoteite, and Joseph P. A. Harrity*

Table of Contents

General Considerations	S2
Phosphoramidite ligand screen	S3
Experimental Procedures	S3
Gram scale synthesis of compound (**7**)	S14
NMR Spectra	S19
HPLC traces of enantioenriched products	S68
X-ray crystallographic analysis for compound **3b**	S85
X-ray crystallographic analysis for compound **3f**	S90
X-ray crystallographic analysis for compound **3i**	S95
References	S101

General Considerations

All reactions were carried out in flame-dried glassware equipped with a magnetic stir bar under nitrogen atmosphere, unless stated otherwise. Solvents were purified using a PureSolv MD purification system and transferred under nitrogen. A DrySyn block combined with a temperature probe was used as the heating source, where required. Infrared (IR) spectra were recorded on a Perkin Elmer Paragon FTIR spectrometer (ν_{max} in cm^{-1}). Samples were recorded neat as thin films. ^{1}H NMR spectra were recorded on a Bruker AVIII HD 400 (400 MHz), Bruker AVI 400 (400 MHz) or Bruker AMX400 (400 MHz). Chemical shifts are reported in parts per million (ppm) from tetramethylsilane, using the residual protic solvent resonance as the internal reference: ($CHCl_3$: δ 7.26) unless otherwise stated. Data are reported as follows: chemical shift (integration, multiplicity (s = singlet, d = doublet, t = triplet, q = quartet, br = broad, m = multiplet), coupling constant (Hz)). ^{13}C NMR spectra were recorded on a Bruker AVIII HD 400 (101 MHz), Bruker AVI 400 (101 MHz) or Bruker AMX-400 (101 MHz) with broadband proton decoupling. Chemical shifts are reported in ppm from trimethylsilane with the solvent as the internal reference ($CDCl_3$: δ 77.16). ^{19}F NMR spectra were recorded on a Bruker AV III HD 400 (377 MHz) and are uncorrected. High resolution mass spectra (HRMS) recorded for accurate mass analysis, were performed on either a Micromass LCT operating in electrospray mode (TOF, ES^+) or a Micromass Prospec operating in FAB (FAB^+), EI (EI^+) or CI (CI^+) mode. Thin layer chromatography (TLC) was performed on aluminium-backed plates pre coated with silica (0.2 mm, Merck 60 F_{254}) which were developed using standard visualizing agents: UV light or potassium permanganate. Flash chromatography was performed on silica gel (Merck 40-63 µm). Melting points were recorded on Gallenkamp melting point apparatus and are uncorrected.

Phosphoramidite Ligand Screen

A range of phosphoramidites were screened for their potential to control enantioselectivity but they were found to be poorly selective across the board (Table S1):

Table S1 Phosphoramidite promoted enantioselective allylation

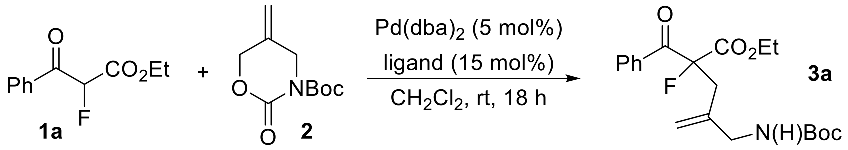

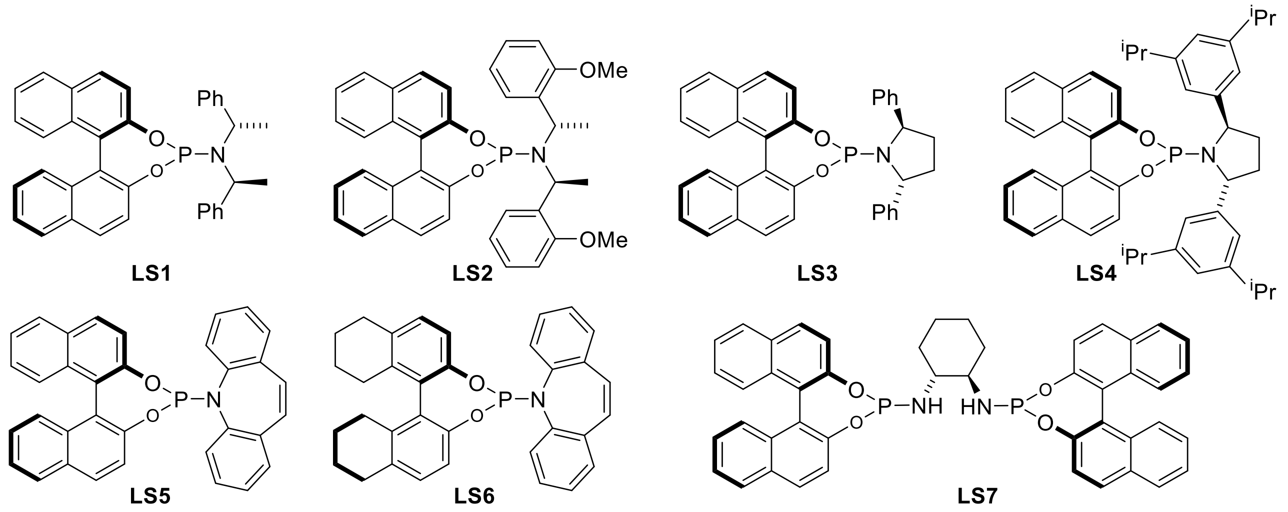

entry	ligand	solvent	conversion	ee
1	**LS1**	NMP	100%	<5%
2	**LS2**	NMP	100%	6%
3	**LS3**	NMP	100%	<5%
4	**LS4**	NMP	100%	<5%
5	**LS5**	NMP	100%	8%
6	**LS6**	NMP	100%	<5%
7	**LS7**	CH_2Cl_2	0%	--

Experimental Procedures

α-Fluoro β-keto esters **1** and **5** and carbamate **2** were prepared according to procedures previously described.[1] Optical rotations were only recorded for enantioenriched compounds of 95% ee or better.

General Procedure for the Asymmetric Allylation Reaction

A flame-dried test-tube was charged with *tert*-butyl 5-methylene-2-oxo-1,3-oxazinane-3-carboxylate (1 eq), *(R,R)*-Dach-phenyl Trost ligand (5.5 mol%), Pd(dba)$_2$ (5 mol%), *t*-BuOH (5 eq) and DIPEA (1.2 eq) under nitrogen. Anhydrous toluene (5 mL per mmol) was then added and the mixture stirred at 0 °C for 30 minutes. Fluorinated keto ester (1.3 eq) in toluene (5 mL per mmol) was added to the mixture and the reaction mixture was stirred overnight at 0 °C. The resulting mixture was then concentrated under vacuum and purified by flash silica column chromatography.

ethyl 2-benzoyl-4-{[(*tert*-butoxycarbonyl)amino]methyl}-2-fluoropent-4-enoate (3a)[1]

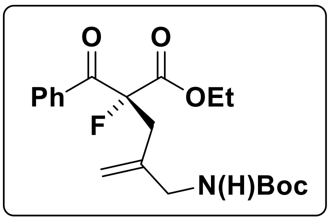

Following the General Procedure with ethyl 2-fluoro-3-oxo-3-phenylpropanoate (**1a**) (27 mg, 0.13 mmol), *tert*-butyl 5-methylene-2-oxo-1,3-oxazinane-3-carboxylate (**2**) (22 mg, 0.1 mmol), Pd(dba)$_2$ (2.9 mg, 0.005 mmol), *(R,R)*-Dach-phenyl Trost ligand (3.8 mg, 0.0055 mmol), *t*-BuOH (0.048 mL, 0.5 mmol) and DIPEA (0.021 mL, 0.12 mmol) in PhMe (1 mL) with FCC (gradient from 20-30% Et$_2$O in petroleum ether) afforded ethyl 2-benzoyl-4-{[(*tert*-butoxycarbonyl)amino]methyl}-2-fluoropent-4-enoate as a pale yellow oil (**3a**) (33 mg, 78%).

^{1}H NMR (400 MHz, CDCl$_3$): δ 8.02 (d, *J* = 8.0 Hz, 2H), 7.58 (t, *J* = 7.5 Hz, 1H), 7.44 (t, *J* = 8.0 Hz, 2H), 5.13 (s, 1H), 5.03 (s, 1H), 4.79 (br, 1H), 4.30 – 4.15 (m, 2H), 3.76 (d, *J* = 4.0 Hz, 2H), 3.13 (dd, *J* = 33.0, 15.0 Hz, 1H), 2.96 (dd, *J* = 18.5, 15.5 Hz, 1H), 1.43 (s, 9H), 1.19 (t, *J* = 7.0 Hz, 3H); **^{13}C NMR (101 MHz, CDCl$_3$)** δ 191.5 (d, *J* = 30.0 Hz), 167.3 (d, *J* = 26.0 Hz), 156.1, 140.0, 134.3, 134.0 (d, *J* = 3.0 Hz), 130.0 (d, *J*= 5.5 Hz), 129.0, 116.3, 100.2 (d, *J*= 200.0 Hz), 79.7, 63.1, 46.1, 38.4 (d, *J* = 20.5 Hz), 28.7, 14.3; **^{19}F NMR (377 MHz, CDCl$_3$)**: δ -160.1 (dd, *J* = 33.0, 18.5 Hz). **HPLC** (Cellulose-1, hexane: ^{i}PrOH 90:10, flow rate 1.0 mL/min, λ = 254 nm, 25 °C) t_R(major) = 6.730 min, t_R(minor) = 9.393 min, ee = 75%.

ethyl 4-{[(*tert*-butoxycarbonyl)amino]methyl}-2-fluoro-2-(4-chloromethylbenzoyl)pent-4-enoate (3b)[1]

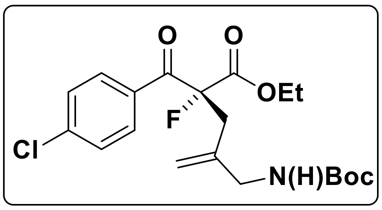

Following the General Procedure with ethyl 2-fluoro-3-(4-chloromethylphenyl)-3-oxopropanoate (**1b**) (32 mg, 0.13 mmol), *tert*-butyl 5-methylene-2-oxo-1,3-oxazinane-3-carboxylate (**2**) (22 mg, 0.1 mmol), Pd(dba)$_2$ (2.9 mg, 0.005 mmol), *(R,R)*-Dach-phenyl Trost ligand (3.8 mg, 0.0055 mmol), *t*-BuOH (0.048 mL, 0.5 mmol) and DIPEA (0.021 mL, 0.12 mmol) in PhMe (1 mL) with FCC (20% Et$_2$O in petroleum ether) afforded ethyl 4-{[(*tert*-butoxycarbonyl)amino]methyl}-2-fluoro-2-(4-chloromethylbenzoyl)pent-4-enoate as a white solid (**3b**) (21 mg, 50%). The product was recrystallized from toluene/CH_2Cl_2 to provide a colorless crystalline solid (>99% ee).

^{1}H NMR (400 MHz, $CDCl_3$): δ 7.97 (dd, *J* = 8.5, 1.5 Hz, 2H), 7.44 – 7.39 (m, 2H), 5.13 (s, 1H), 5.03 (s, 1H), 4.79 (br, 1H), 4.30 – 4.15 (m, 2H), 3.76 (d, *J* = 4.0 Hz, 2H), 3.13 (dd, *J* = 33.0, 15.0 Hz, 1H), 2.96 (dd, *J* = 18.5, 15.5 Hz, 1H), 1.43 (s, 9H), 1.19 (t, *J* = 7.0 Hz, 3H); **^{13}C NMR (101 MHz, $CDCl_3$)** δ 190.4 (d, *J* = 30.0 Hz), 167.0 (d, *J* = 26.0 Hz), 156.1, 141.0, 139.8, 132.2 (d, *J* = 3.5 Hz), 131.6 (d, *J* = 6.0 Hz), 129.3, 116.3, 100.2 (d, *J*= 200.0 Hz), 79.7, 63.2, 45.9, 38.3 (d, *J* = 20.5 Hz), 28.7, 14.3; **^{19}F NMR (377 MHz, $CDCl_3$)**: δ -157.7 (dd, *J* = 33.0, 19.0 Hz). **m.p**: 56.4-58.0°C. **HPLC** (Cellulose-1, hexane: ^{i}PrOH 90:10, flow rate 1.0 mL/min, λ = 254 nm, 25 °C) t_R(major) = 7.127 min, t_R(minor) = 17.703 min, ee = 81%; $[\alpha]_D^{22} = -30$ (*c* 1.0, $CHCl_3$).

ethyl 4-{[(*tert*-butoxycarbonyl)amino]methyl}-2-fluoro-2-(4-nitrobenzoyl)pent-4-enoate (3c)[1]

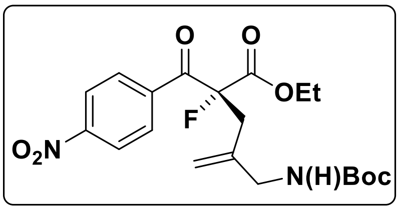

Following the General Procedure with ethyl 2-fluoro-3-(4-chloromethylphenyl)-3-oxopropanoate (**1c**) (32 mg, 0.13 mmol), *tert*-butyl 5-methylene-2-oxo-1,3-oxazinane-3-carboxylate (**2**) (22 mg, 0.1 mmol), $Pd(dba)_2$ (2.9 mg, 0.005 mmol), *(R,R)*-Dach-phenyl Trost ligand (3.8 mg, 0.0055 mmol), *t*-BuOH (0.048 mL, 0.5 mmol) and DIPEA (0.021 mL, 0.12 mmol) in PhMe (1 mL) with FCC (gradient from 20-30% Et_2O in petroleum ether) afforded ethyl 4-{[(*tert*-butoxycarbonyl)amino]methyl}-2-fluoro-2-(4-nitrobenzoyl)pent-4-enoate as an orange oil (**3c**) (32 mg, 77%).

^{1}H NMR (400 MHz, $CDCl_3$): δ 8.27 (d, *J* = 9.0 Hz, 2H), 8.17 (d, *J* = 9.0 Hz, 2H), 5.13 (s, 1H), 5.03 (s, 1H), 4.79 (br, 1H), 4.30 – 4.15 (m, 2H), 3.76 (d, *J* = 4.0 Hz, 2H), 3.13 (dd, *J* = 33.0, 15.0 Hz, 1H), 2.96 (dd, *J* = 18.5, 15.5 Hz, 1H), 1.43 (s, 9H), 1.19 (t, *J* = 7.0 Hz, 3H); **^{13}C NMR (101 MHz, $CDCl_3$)** δ 190.6 (d, *J* = 28.0 Hz), 166.3 (d, *J* = 26.0 Hz), 155.9, 150.7, 139.4, 138.9 (d, *J* = 106.5 Hz), 131.0 (d, *J* = 6.0 Hz), 123.9, 116.4, 100.2 (d, *J* = 200.0 Hz), 79.5, 62.9, 45.8, 38.2 (d, *J* = 20.5 Hz), 28.5, 14.1; **^{19}F NMR (377 MHz, $CDCl_3$)**: δ -158.3 (dd, *J* = 32.0, 20.5 Hz). **HPLC** (Cellulose-2, hexane: ^{i}PrOH 90:10, flow rate 1.0 mL/min, λ = 254 nm, 25 °C) t_R(major) = 15.393 min, t_R(minor) = 18.277 min, ee = 80%.

ethyl 4-{[(*tert*-butoxycarbonyl)amino]methyl}-2-fluoro-2-(4-trifluoromethylbenzoyl)pent-4-enoate (3d)[1]

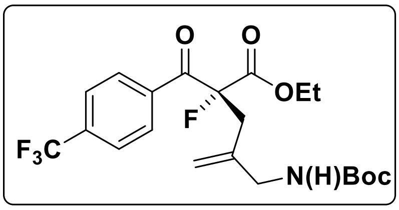

Following the General Procedure with ethyl 2-fluoro-3-(4-trifluoromethylphenyl)-3-oxopropanoate (**1d**) (37 mg, 0.13 mmol), *tert*-butyl 5-methylene-2-oxo-1,3-oxazinane-3-carboxylate (**2**) (22 mg, 0.1 mmol), $Pd(dba)_2$ (2.9 mg, 0.005 mmol), *(R,R)*-Dach-phenyl Trost ligand (3.8 mg, 0.0055 mmol), *t*-BuOH (0.048 mL, 0.5 mmol) and DIPEA (0.021 mL, 0.12 mmol) in PhMe (1 mL) with FCC (gradient from 20-30% Et_2O in petroleum ether) afforded ethyl 4-{[(*tert*-

butoxycarbonyl)amino]methyl}-2-fluoro-2-(4-trifluoromethylbenzoyl)pent-4-enoate as a pale yellow oil (**3d**) (37 mg, 82%).

^{1}H NMR (400 MHz, CDCl$_3$): δ 8.13 (d, *J* = 8.0 Hz, 2H), 7.71 (d, *J* = 8.0 Hz, 2H), 5.14 (s, 1H), 5.03 (s, 1H), 4.76 (br, 1H), 3.75 (d, *J* = 5.5 Hz, 2H), 3.13 (dd, *J* = 32.5, 15.5 Hz, 1H), 2.97 (dd, *J* = 19.5, 15.5 Hz, 1H), 1.42 (s, 9H), 1.21 (t, *J* = 7.0 Hz, 3H); **^{13}C NMR (101 MHz, CDCl$_3$)** δ 191.0 (d, *J* = 26.5 Hz), 166.8 (d, *J* = 26.0 Hz), 156.1, 139.8, 136.7, 135.4 (q, *J* = 33.0 Hz), 130.5 (d, *J* = 6.0 Hz), 126.0 (d, *J* = 3.5 Hz), 123.7 (q, *J* = 273.0 Hz), 116.5, 100.3 (d, *J* = 200.0 Hz), 79.8, 63.1, 46.0, 38.3 (d, *J* = 20.5 Hz), 28.7, 14.3; **^{19}F NMR (377 MHz, CDCl$_3$)**: δ -63.4 (s), -160.1 (dd, *J* = 32.5, 19.5 Hz). **HPLC** (Cellulose-2, hexane: ^{i}PrOH 90:10, flow rate 1.0 mL/min, λ = 254 nm, 25 °C) t$_R$(major) = 5.133 min, t$_R$(minor) = 6.020 min, ee = 81%.

ethyl 4-{[(*tert*-butoxycarbonyl)amino]methyl}-2-fluoro-2-(4-methoxybenzoyl)pent-4-enoate (3e)[1]

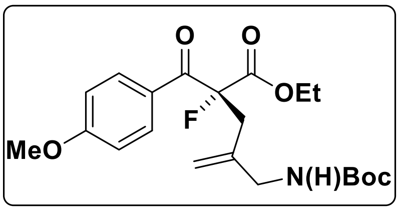

Following the General Procedure with ethyl 2-fluoro-3-(4-methoxyphenyl)-3-oxopropanoate (**1e**) (32 mg, 0.13 mmol), *tert*-butyl 5-methylene-2-oxo-1,3-oxazinane-3-carboxylate (**2**) (22 mg, 0.1 mmol), Pd(dba)$_2$ (2.9 mg, 0.005 mmol), *(R,R)*-Dach-phenyl Trost ligand (3.8 mg, 0.0055 mmol), *t*-BuOH (0.048 mL, 0.5 mmol) and DIPEA (0.021 mL, 0.12 mmol) in PhMe (1 mL) with FCC (gradient from 20-30% Et$_2$O in petroleum ether) afforded ethyl 4-{[(*tert*-butoxycarbonyl)amino]methyl}-2-fluoro-2-(4-methoxybenzoyl)pent-4-enoate as an orange oil (**3e**) (27 mg, 68%).

^{1}H NMR (400 MHz, CDCl$_3$): **^{1}H NMR (400 MHz, CDCl$_3$)**: δ 8.06 (dd, *J* = 9.0, 1.5 Hz, 2H), 6.91 (d, *J* = 9.0 Hz, 2H), 5.14 (s, 1H), 5.03 (s, 1H), 4.76 (br, 1H), 4.29 – 4.13 (m, 2H), 3.87 (s, 3H), 3.75 (d, *J* = 5.5 Hz, 2H), 3.13 (dd, *J* = 32.5, 15.5 Hz, 1H), 2.97 (dd, *J* = 19.5, 15.5 Hz, 1H), 1.42 (s, 9H), 1.21 (t, *J* = 7.0 Hz, 3H); **^{13}C NMR (101 MHz, CDCl$_3$)** δ 189.4 (d, *J* = 25.0 Hz), 167.4 (d, *J* = 26.0 Hz), 164.3, 155.9, 139.9, 132.5 (d, *J* = 6.0 Hz), 126.5 (d, *J* = 3.5 Hz), 116.0, 114.1, 100.0 (d, *J* = 200.5 Hz), 79.5, 62.7, 55.6, 45.8, 38.2 (d, *J* = 20.5 Hz), 28.5, 14.1; **^{19}F NMR (377 MHz, CDCl$_3$)**: δ -156.9 (dd, *J* = 33.8, 18.0 Hz). **HPLC** (Cellulose-1, hexane: ^{i}PrOH 90:10, flow rate 1.0 mL/min, λ = 254 nm, 25 °C) t$_R$(major) = 9.847 min, t$_R$(minor) = 14.267 min, ee = 74%.

ethyl 4-{[(*tert*-butoxycarbonyl)amino]methyl}-2-fluoro-2-(4-methylbenzoyl)pent-4-enoate (3f)[1]

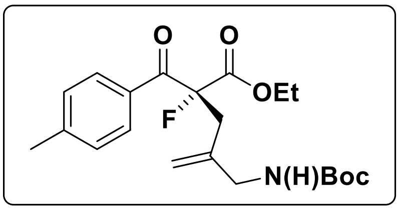

Following the General Procedure with ethyl 2-fluoro-3-(4-methylphenyl)-3-oxopropanoate (**1f**) (30 mg, 0.13 mmol), *tert*-butyl 5-methylene-2-oxo-1,3-oxazinane-3-carboxylate (**2**) (22 mg, 0.1 mmol), Pd(dba)$_2$ (2.9 mg, 0.005 mmol), *(R,R)*-Dach-phenyl Trost

ligand (3.8 mg, 0.0055 mmol), *t*-BuOH (0.048 mL, 0.5 mmol) and DIPEA (0.021 mL, 0.12 mmol) in PhMe (1 mL) with FCC (gradient from 20-30% Et_2O in petroleum ether) afforded ethyl 4-{[(*tert*-butoxycarbonyl)amino]methyl}-2-fluoro-2-(4-methylbenzoyl)pent-4-enoate as a white solid (**3f**) (18 mg, 47%). The product was recrystallized from toluene/CH_2Cl_2 to provide a colorless crystalline solid (96% ee).

^{1}H NMR (400 MHz, $CDCl_3$): δ 7.94 (dd, *J* = 8.0 Hz, 2H), 7.25 (d, *J* = 8.0 Hz, 2H), 5.14 (s, 1H), 5.03 (s, 1H), 4.78 (br, 1H), 4.30 – 4.16 (m, 2H), 3.77 (s, 2H), 3.13 (dd, *J* = 33.5, 15.5 Hz, 1H), 2.95 (dd, *J* = 18.0, 15.5 Hz, 1H), 2.40 (s, 3H), 1.43 (s, 9H), 1.19 (t, *J* = 7.0 Hz, 3H); **^{13}C NMR (101 MHz, $CDCl_3$)** δ 190.7 (d, *J* = 25.5 Hz), 167.2 (d, *J* = 26.0 Hz), 155.9, 145.3, 139.9, 131.2 (d, *J* = 3.5 Hz), 130.1 (d, *J* = 5.5 Hz), 129.5, 116.0, 99.9 (d, *J* = 200.5 Hz), 79.5, 62.8, 45.8, 38.2 (d, *J* = 20.5 Hz), 28.5, 21.9, 14.1; **^{19}F NMR (377 MHz, $CDCl_3$)**: δ -157.3 (dd, *J* = 33.5, 18.5 Hz). **m.p:** 72.5-74.6°C**. HPLC** (Cellulose-2, hexane: ^{i}PrOH 90:10, flow rate 1.0 mL/min, λ = 254 nm, 25 °C) t_R(major) = 9.873 min, t_R(minor) = 11.433 min, ee = 77%; $[\alpha]_D^{22} = -30$ (*c* 1.0, $CHCl_3$).

ethyl 4-{[(*tert*-butoxycarbonyl)amino]methyl}-2-fluoro-2-(4-methylbenzoyl)pent-4-enoate (3g)[1]

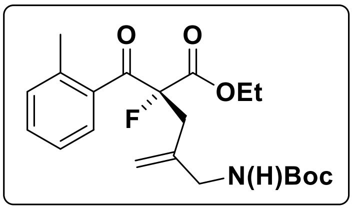

Following the General Procedure with ethyl 2-fluoro-3-(4-methylphenyl)-3-oxopropanoate (**1g**) (30 mg, 0.13 mmol), *tert*-butyl 5-methylene-2-oxo-1,3-oxazinane-3-carboxylate (**2**) (22 mg, 0.1 mmol), $Pd(dba)_2$ (2.9 mg, 0.005 mmol), *(R,R)*-Dach-phenyl Trost ligand (3.8 mg, 0.0055 mmol), *t*-BuOH (0.048 mL, 0.5 mmol) and DIPEA (0.021 mL, 0.12 mmol) in PhMe (1 mL) with FCC (gradient from 20-30% Et_2O in petroleum ether) afforded ethyl 4-{[(*tert*-butoxycarbonyl)amino]methyl}-2-fluoro-2-(4-methylbenzoyl)pent-4-enoate as a white solid (**3g**) (20 mg, 83%).

^{1}H NMR (400 MHz, $CDCl_3$): δ 7.67 (dd, *J* = 7.5, 3.0 Hz, 1H), 7.38 (td, *J* = 7.5, 1.0 Hz, 1H), 7.32 – 7.21 (m, 2H), 5.13 (s, 1H), 5.05 (s, 1H), 4.77 (br, 1H), 4.25 (qd, *J* = 7.0, 2.0 Hz, 2H), 3.73 (d, *J* = 5.5 Hz, 2H), 3.14 – 2.95 (m, 2H), 2.41 (s, 3H), 1.43 (s, 9H), 1.24 (t, *J* = 7.0 Hz, 3H); **^{13}C NMR (101 MHz, $CDCl_3$)** δ 195.4 (d, *J* = 26.5 Hz), 166.8 (d, *J* = 26.0 Hz), 155.9, 139.8, 139.3, 134.3 (d, *J* =3.0 Hz), 132.1, 132.0, 129.0 (d, *J* = 9.0 Hz), 125.5, 116.1, 100.3 (d, *J* = 202.0 Hz), 79.5, 62.9, 45.8, 38.6 (d, *J* = 20.5 Hz), 28.5, 20.9, 14.1; **^{19}F NMR (377 MHz, $CDCl_3$)**: δ -158.1 (dd, *J* = 29.0, 22.0 Hz). **HPLC** (Cellulose-1, hexane: ^{i}PrOH 90:10, flow rate 1.0 mL/min, λ = 254 nm, 25 °C) t_R(major) = 5.497 min, t_R(minor) = 6.563 min, ee = 86%.

ethyl 4-(((*tert*-butoxycarbonyl)amino)methyl)-2-fluoro-2-(furan-2-carbonyl)pent-4-enoate (3h)

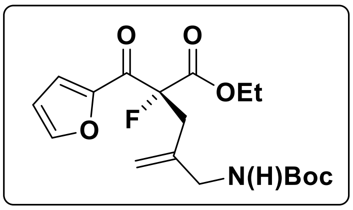

Following the General Procedure with ethyl 2-fluoro-3-oxo-3-(furan-2-yl)propanoate (**1h**) (26 mg, 0.13 mmol), *tert*-butyl 5-methylene-2-oxo-1,3-oxazinane-3-carboxylate (**2**) (22 mg, 0.1 mmol), Pd(dba)$_2$ (2.9 mg, 0.005 mmol), *(R,R)*-Dach-phenyl Trost ligand (3.8 mg, 0.0055 mmol), *t*-BuOH (0.048 mL, 0.5 mmol) and DIPEA (0.021 mL, 0.12 mmol) in PhMe (1 mL) with FCC (gradient from 30-40% Et$_2$O in petroleum ether) afforded ethyl 4-{[(*tert*-butoxycarbonyl)amino]methyl}-2-fluoro-2-(4-methylbenzoyl)pent-4-enoate as a yellow oil (**3h**) (29 mg, 80%).

^{1}H NMR (400 MHz, CDCl$_3$): δ 7.69 (br, 1H), 7.50 (t, *J* = 3.5 Hz, 1H), 6.57 (dd, *J* = 3.5, 1.5 Hz, 1H), 5.12 (s, 1H), 5.04 (s, 1H), 4.75 (br, 1H), 4.29 – 4.17 (m, 2H), 3.74 (d, *J* = 5.5 Hz, 2H), 3.10 (dd, *J* = 31.5, 15.5 Hz, 1H), 2.95 (dd, *J* = 20.5, 15.5 Hz, 1H), 1.43 (s, 9H), 1.23 (t, *J* = 7.0 Hz, 3H); **^{13}C NMR (101 MHz, CDCl$_3$)** δ 179.4 (d, *J* = 26.5 Hz), 166.3 (d, *J* = 26.5 Hz), 156.9, 149.0 (d, *J* = 3.5 Hz), 148.5, 139.5, 122.8 (d, *J* = 11.0 Hz), 116.2, 112.8(d, *J* = 1.5 Hz), 99.2 (d, *J* = 200.0 Hz), 79.6, 62.9, 45.8, 37.5 (d, *J* = 20.0 Hz), 28.5, 14.1; **^{19}F NMR (377 MHz, CDCl$_3$)**: δ -160.9 (dd, *J* = 30.5, 22.0 Hz); **FTIR:** ν_{max}/cm^{-1} (neat) 2979, 1756, 1689, 1512, 1461, 1366, 1249, 1169, 1028, 905, 862, 773 cm^{-1}; **HRMS (ESI$^+$):** calculated for $C_{18}H_{24}FNO_6Na$ (ES$^+$)(+Na$^+$): 392.1485. Found: 392.1472. **HPLC** (Cellulose-1, hexane: ^{i}PrOH 90:10, flow rate 1.0 mL/min, λ = 254 nm, 25 °C) t_R(major) = 10.737 min, t_R(minor) = 13.723 min, ee = 76%.

ethyl 2-(2-naphthoyl)-4-(((*tert*-butoxycarbonyl)amino)methyl)-2-fluoropent-4-enoate (3i)[1]

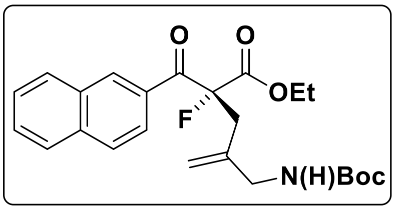

Following the General Procedure with ethyl 2-fluoro-3-(naphthalen-2-yl)-3-oxopropanoate (**1i**) (34 mg, 0.13 mmol), *tert*-butyl 5-methylene-2-oxo-1,3-oxazinane-3-carboxylate (2) (22 mg, 0.1 mmol), Pd(dba)$_2$ (2.9 mg, 0.005 mmol), *(R,R)*-Dach-phenyl Trost ligand (3.8 mg, 0.0055 mmol), *t*-BuOH (0.048 mL, 0.5 mmol) and DIPEA (0.021 mL, 0.12 mmol) in PhMe (1 mL) with FCC (gradient from 15-25% Et$_2$O in petroleum ether) afforded ethyl 2-(2-naphthoyl)-4-(((*tert*-butoxycarbonyl)amino)methyl)-2-fluoropent-4-enoate as a white solid (**3i**) (33 mg, 80%). The product was recrystallized from toluene/CH$_2$Cl$_2$ to provide a colorless crystalline solid (>99% ee).

^{1}H NMR (400 MHz, CDCl$_3$) δ 8.65 (s, 1H), 8.03 (dt, *J* = 8.5, 1.5 Hz, 1H), 7.97 (d, *J* = 8.0 Hz, 1H), 7.87 (t, *J* = 8.5 Hz, 2H), 7.65 – 7.59 (m, 1H), 7.58 – 7.52 (m, 1H), 5.16 (s, 1H), 5.07 (s, 1H), 4.79 (br, 1H), 4.34 – 4.15 (m, 2H), 3.80 (br, 2H), 3.29 – 2.97 (m, 2H), 1.44 (s, 9H), 1.20 (t, *J* = 7.0 Hz, 3H); **^{13}C NMR (101 MHz, CDCl$_3$)** δ 191.0 (d, *J* = 25.5 Hz), 167.3 (d, *J* = 26.0 Hz), 156.0, 139.9, 136.0, 132.4, 132.3, 131.0 (2C), 130.3, 129.4, 128.7, 127.9, 127.1, 124.9 (d, *J* = 3.0 Hz), 116.2, 100.3 (d, *J* = 200.5 Hz), 63.0, 45.8, 38.3 (d, *J* = 20.5 Hz), 28.6, 14.2; **^{19}F NMR (377 MHz, CDCl$_3$)**: δ -158.1 (dd, *J* = 33.5, 18.5 Hz). **m.p:** 98.6-

99.6°C. **HPLC** (Cellulose-2, hexane: ^{i}PrOH 90:10, flow rate 1.0 mL/min, λ = 254 nm, 25 °C) t_R(major) = 9.787 min, t_R(minor) = 11.630 min, ee = 82%; $[\alpha]_D^{22} = -30$ (*c* 1.0, $CHCl_3$).

ethyl 2-acetyl-4-{[(*tert*-butoxycarbonyl)amino]methyl}-2-fluoropent-4-enoate (3j)[1]

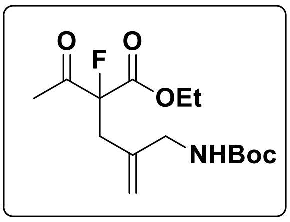

Following the General Procedure with ethyl 2-fluoro-3-oxobutanoate (**1j**) (18 mg, 0.13 mmol), *tert*-butyl 5-methylene-2-oxo-1,3-oxazinane-3-carboxylate (**2**) (22 mg, 0.1 mmol), $Pd(dba)_2$ (2.9 mg, 0.005 mmol), *(R,R)*-Dach-phenyl Trost ligand (3.8 mg, 0.0055 mmol), *t*-BuOH (0.048 mL, 0.5 mmol) and DIPEA (0.021 mL, 0.12 mmol) in PhMe (1 mL) with FCC (gradient from 20-30% Et_2O in petroleum ether) afforded ethyl 2-acetyl-4-{[(*tert*-butoxycarbonyl)amino]methyl}-2-fluoropent-4-enoate as a colorless oil (**3j**) (21 mg, 65%).
^{1}H NMR (400 MHz, $CDCl_3$) δ 5.10 (s, 1H), 4.99 (s, 1H), 4.73 (br, 1H), 4.24 (q, *J* = 7.0 Hz, 2H), 3.77 – 3.62 (m, 2H), 2.94 – 2.73 (m, 2H), 2.29 (d, *J* = 5.0 Hz, 3H), 1.43 (s, 9H), 1.28 (t, *J* = 7.0 Hz, 3H); **^{13}C NMR (101 MHz, $CDCl_3$)** δ 201.5 (d, *J* = 29.5 Hz), 165.8 (d, *J* = 25.5 Hz), 155.9, 139.5, 115.8, 100.4 (d, *J* = 200.0 Hz), 79.5, 62.9, 45.8, 37.3 (d, *J* = 20.0 Hz), 28.5, 26.0, 14.1; **^{19}F NMR (377 MHz, $CDCl_3$):** δ -163.6 – -163.7 (m);. **HPLC** (Cellulose-1, hexane: ^{i}PrOH 95:5, flow rate 1.0 mL/min, λ = 254 nm, 25 °C) t_R(major) = 11.583 min, t_R(minor) = 14.563 min, ee = 61%.

Synthesis and stereochemical assignment of silyl enol ethers (4)[2]

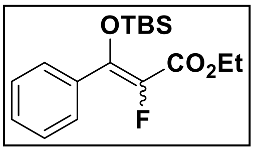

Ethyl 2-fluoro-3-oxo-3-phenylpropanoate (0.4 g, 1.9 mmol) was dissolved in CH_2Cl_2 (4 mL) and cooled to 0 °C. *tert*-Butyldimethylsilyl trifluoromethanesulfonate (1.0 g, 3.8 mmol) followed by Et_3N (0.68 g, 4.8 mmol) were each added dropwise and the reaction was allowed to warm to r.t. and stirred under nitrogen overnight. After solvent removal under reduced pressure, the crude material was subjected to silica gel flash column chromatography (petroleum ether-Et_2O (98:2)) to give silyl enol ether as a colourless oil (4) (0.38 g, 62%) Careful purification allowed each isomer to be separated; major isomer assigned as *Z*: 0.33g 87%, minor isomer assigned as *E*: 0.05 g, 13%). Stereochemical assignments were based on the propensity of α-fluoroketones to form Z-enolates under Lewis acid or strong base conditions.[2]

Major isomer: ^{1}H NMR (400 MHz, $CDCl_3$) δ 7.42 - 7.32 (m, 5H), 4.09 (q, J = 7.0 Hz, 2H), 1.10 (t, J = 7.0 Hz, 3H), 0.90 (s, 9H), 0.07 (d, J = 1.5 Hz, 6H); **^{13}C NMR (101 MHz, $CDCl_3$)** δ 161.7 (d, J = 30.0 Hz), 149.6 (d, J = 14.5 Hz), 139.3, 134.8, 129.5, 129.1 (d, J = 2.5 Hz), 127.7, 60.7, 25.4, 18.4, 13.9, -4.5 (d, J = 3.0 Hz); **^{19}F NMR (377 MHz, $CDCl_3$)** δ -148.2.

Minor isomer: ^{1}H NMR (400 MHz, $CDCl_3$) δ 7.57 - 7.49 (m, 2H), 7.43 - 7.34 (m, 3H), 4.34 (q, J = 7.0 Hz, 2H), 1.36 (t, J = 7.0 Hz, 3H), 0.92 (s, 9H), -0.05 (s, 6H); **^{13}C NMR (101 MHz, $CDCl_3$)** δ 161.7 (d, J = 30.0 Hz), 149.6 (d, J = 14.5 Hz), 136.9, 134.8, 129.9, 128.3 (d, J = 5.5 Hz), 128.1, 60.7, 25.7, 18.5, 14.5, -4.3; **^{19}F NMR (377 MHz, $CDCl_3$)** δ -158.7.

***tert*-butyl 2-benzoyl-4-(((*tert*-butoxycarbonyl)amino)methyl)-2-fluoropent-4-enoate (6a)**

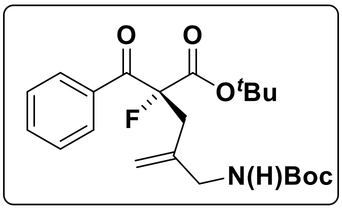

Following the General Procedure with *tert*-butyl 2-fluoro-3-oxo-3-phenylpropanoate (**5a**) (31 mg, 0.13 mmol), *tert*-butyl 5-methylene-2-oxo-1,3-oxazinane-3-carboxylate (**2**) (22 mg, 0.1 mmol), $Pd(dba)_2$ (2.9 mg, 0.005 mmol), *(R,R)*-Dach-phenyl Trost ligand (3.8 mg, 0.0055 mmol), *t*-BuOH (0.048 mL, 0.5 mmol) and DIPEA (0.021 mL, 0.12 mmol) in PhMe (1 mL) with FCC (gradient from 15-25% Et_2O in petroleum ether) afforded *tert*-butyl 2-benzoyl-4-(((*tert*-butoxycarbonyl)amino)methyl)-2-fluoropent-4-enoate as a yellow oil (**6a**) (30 mg, 75%).

^{1}H NMR (400 MHz, $CDCl_3$) δ 8.02 (d, *J* = 8.0 Hz, 2H) , 7.58 (t, *J* = 7.5 Hz, 1H), 7.44 (t, *J* = 8.0 Hz, 2H), 5.15 (s, 1H), 5.05 (s, 1H), 4.79 (br, 1H), 3.78 (br, 2H), 3.09 (dd, *J* = 34.0, 15.5 Hz, 1H), 2.94 (dd, *J* = 18.0, 15.5 Hz, 1H), 1.44 (s, 9H), 1.36 (s, 9H)**; ^{13}C NMR (101 MHz, $CDCl_3$)** δ 191.4 (d, *J* = 26.0 Hz), 165.8 (d, *J* = 25.5 Hz), 156.9, 139.9, 133.9, 133.7 (d, *J* = 7.5 Hz), 129.8 (d, *J* = 4.0 Hz), 128.7, 116.1, 99.4 (d, *J* = 200.0 Hz), 84.6, 79.5, 45.7, 38.1 (d, *J* = 21.0 Hz), 28.5, 27.9; **^{19}F NMR (377 MHz, $CDCl_3$)**: δ -156.2 (dd, *J* = 33.8, 18.0 Hz). **FTIR:** ν_{max}/cm^{-1} (neat) 2979, 2929, 1707, 1512, 1422, 1366, 1223, 1155, 1059, 908, 839, 697; **HRMS (ESI$^+$):** calculated for $C_{22}H_{30}FNO_5Na$ (ES$^+$)(+Na$^+$): 430.2006. Found: 430.2009. **HPLC** (Cellulose-1, hexane: ^{i}PrOH 99.5:0.5, flow rate 1.0 mL/min, λ = 254 nm, 25 °C) t_R(major) =32.000 min, t_R(minor) = 34.937 min, ee = 90%.

***tert*-butyl 4-(((*tert*-butoxycarbonyl)amino)methyl)-2-(4-chlorobenzoyl)-2-fluoropent-4-enoate (6b)**

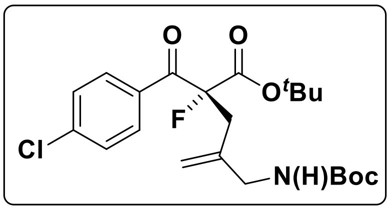

Following the General Procedure with *tert*-butyl 3-(4-chlorophenyl)-2-fluoro-3-oxopropanoate (**5b**) (35 mg, 0.13 mmol), *tert*-butyl 5-methylene-2-oxo-1,3-oxazinane-3-carboxylate (**2**) (22 mg, 0.1 mmol), $Pd(dba)_2$ (2.9 mg, 0.005 mmol), *(R,R)*-Dach-phenyl Trost ligand (3.8 mg, 0.0055 mmol), t-BuOH (0.048 mL, 0.5 mmol) and DIPEA (0.021 mL, 0.12 mmol) in PhMe (1 mL) with FCC (gradient from 10-15% Et_2O in petroleum ether) afforded *tert*-butyl 4-(((*tert*-butoxycarbonyl)amino)methyl)-2-(4-chlorobenzoyl)-2-fluoropent-4-enoate as a yellow oil (**6b**) (32 mg, 72%).

^{1}H NMR (400 MHz, $CDCl_3$) ^{1}H NMR (400 MHz, $CDCl_3$): δ 7.98 (dd, *J* = 8.5, 1.5 Hz, 2H), 7.41 (d, *J* = 8.5 Hz, 2H), 5.13 (s, 1H), 5.03 (s, 1H), 4.79 (br, 1H), 3.76 (d, *J* = 5.0 Hz, 2H), 3.07 (dd, *J* = 34.0, 15.5 Hz, 1H),

2.90 (dd, *J* = 18.0, 15.5 Hz, 1H), 1.42 (s, 9H), 1.36 (s, 9H) ; **^{13}C NMR (101 MHz, CDCl$_3$)** δ 190.4 (d, *J* = 26.0 Hz), 165.9 (d, *J* = 25.5 Hz), 156.1, 140.8, 140.1, 132.3, 131.5 (d, *J* = 5.5 Hz), 129.3, 116.3, 99.6 (d, *J* = 199.0 Hz), 85.0, 79.7, 45.9, 38.2 (d, *J* = 20.5 Hz), 28.7, 28.0; **^{19}F NMR (377 MHz, CDCl$_3$)**: δ -156.3 (dd, *J* = 34.0, 18.5 Hz). **FTIR:** $ν_{max}$/cm^{-1} (neat) 2924, 1750, 1460, 1379, 908, 725; **HRMS (ESI$^+$):** calculated for $C_{22}H_{29}{}^{35}ClFNO_5Na$ (ES$^+$)(+Na$^+$): 464.1616. Found: 464.2206. **HPLC** (Cellulose-1, hexane: ^{i}PrOH 95.0:5.0, flow rate 1.0 mL/min, λ = 254 nm, 25 °C) t_R(major) = 5.997 min, t_R(minor) =7.533 min, ee = 91%.

tert-butyl 4-(((_tert_-butoxycarbonyl)amino)methyl)-2-fluoro-2-(4-nitrobenzoyl)pent-4-enoate (6c)

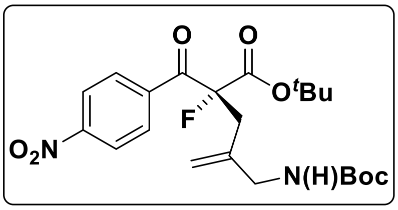

Following the General Procedure with *tert*-butyl 2-fluoro-3-oxo-3-phenylpropanoate (**5c**) (37 mg, 0.13 mmol), *tert*-butyl 5-methylene-2-oxo-1,3-oxazinane-3-carboxylate (**2**) (22 mg, 0.1 mmol), Pd(dba)$_2$ (2.9 mg, 0.005 mmol), *(R,R)*-Dach-phenyl Trost ligand (3.8 mg, 0.0055 mmol), *t*-BuOH (0.048 mL, 0.5 mmol) and DIPEA (0.021 mL, 0.12 mmol) in PhMe (1 mL) with FCC (20% Et$_2$O in petroleum ether) afforded *tert*-butyl 4-(((*tert*-butoxycarbonyl)amino)methyl)-2-fluoro-2-(4-nitrobenzoyl)pent-4-enoate as a yellow oil (**6c**) (35 mg, 80%).

^{1}H NMR (400 MHz, CDCl$_3$) δ 8.29 (d, *J* = 9.0 Hz, 2H) , 8.19 (dd, *J* = 9.0, 1.0 Hz, 1H), δ 5.16 (s, 1H), 5.06 (s, 1H), 4.76 (br, 1H), 3.76 (d, *J* = 5.5 Hz, 2H), 3.10 (dd, *J* = 32.5, 15.5 Hz, 1H), 2.94 (dd, *J* = 19.5, 15.5 Hz, 1H), 1.43 (s, 9H), 1.38 (s, 9H)**; ^{13}C NMR (101 MHz, CDCl$_3$)** δ 190.7 (d, *J* = 27.0 Hz), 165.2 (d, *J* = 25.5 Hz), 156.9, 150.7, 139.6, 138.5 (d, *J* = 3.5 Hz), 130.9 (d, *J* = 5.5 Hz), 123.8, 116.3, 99.7 (d, *J* = 199.0 Hz), 84.6, 79.5, 45.7, 37.9 (d, *J* = 20.5 Hz), 28.5, 27.9; **^{19}F NMR (377 MHz, CDCl$_3$)**: δ -156.9 (dd, *J* = 32.5, 19.5 Hz). **FTIR:** $ν_{max}$/cm^{-1} (neat) 3020, 1717, 1529, 1370, 1214, 1153, 747, 668; **HRMS (ESI$^+$):** calculated for $C_{22}H_{29}FN_2O_7Na$ (ES$^+$)(+Na$^+$): 475.1851. Found: 475.1866. **HPLC** (Cellulose-1, hexane: ^{i}PrOH 97.0:3.0, flow rate 1.0 mL/min, λ = 254 nm, 25 °C) t_R(major) = 22.683 min, t_R(minor) = 26.023 min, ee = 91%.

tert-butyl 4-(((_tert_-butoxycarbonyl)amino)methyl)-2-fluoro-2-(4-(trifluoromethyl)benzoyl)pent-4-enoate (6d)

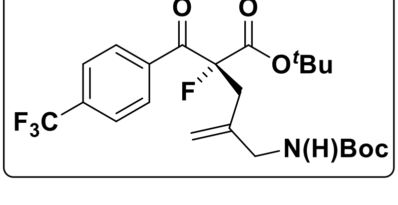

Following the General Procedure with *tert*-butyl 2-fluoro-3-oxo-3-(4-(trifluoromethyl)phenyl)propanoate (**5d**) (40 mg, 0.13 mmol), *tert*-butyl 5-methylene-2-oxo-1,3-oxazinane-3-carboxylate (22 mg, 0.1 mmol), Pd(dba)$_2$ (2.9 mg, 0.005 mmol) , *(R,R)*-Dach-phenyl Trost ligand (3.8 mg, 0.0055 mmol), t-BuOH (0.048 mL, 0.5 mmol) and DIPEA (0.021 mL, 0.12 mmol) in PhMe (1 mL) with FCC (15% Et$_2$O in petroleum ether) afforded *tert*-butyl 4-(((*tert*-

butoxycarbonyl)amino)methyl)-2-fluoro-2-(4-(trifluoromethyl)benzoyl)pent-4-enoate as a yellow oil (**6d**) (39 mg, 83%).

^{1}H NMR (400 MHz, CDCl$_3$) **^{1}H NMR (400 MHz, CDCl$_3$)**: δ 8.14 (d, *J* = 8.0 Hz, 2H), 7.72 (d, *J* = 8.0 Hz, 2H), 5.15 (s, 1H), 5.06 (s, 1H), 4.76 (br, 1H), 4 3.77 (d, *J* = 5.0 Hz, 2H), 3.08 (dd, *J* = 33.0, 15.5 Hz, 1H), 2.94 (dd, *J* = 19.0, 15.5 Hz, 1H), 1.44 (s, 9H), 1.38 (s, 9H); **^{13}C NMR (101 MHz, CDCl$_3$)** δ 190.9 (d, *J* = 26.5 Hz), 165.5 (d, *J* = 24.5 Hz), 155.9, 139.7, 136.7, 135.1 (q, *J* = 33.5 Hz), 130.2 (d, *J* = 5.0 Hz), 125.7 (d, *J* = 3.5 Hz), 123.6 (d, *J* = 274.0 Hz), 116.3, 99.6 (d, *J* = 199.5 Hz), 85.1, 79.6, 45.7, 38.0 (d, *J* = 20.6 Hz), 28.5, 27.9; **^{19}F NMR (377 MHz, CDCl$_3$)**: δ -63.3 (s), -156.8 (dd, J = 33.0, 19.0 Hz). **FTIR:** ν_{max}/cm^{-1} (neat) 2980, 1754, 1704, 1512, 1411, 1327, 1131, 1065, 858, 759; **HRMS (ESI$^+$):** calculated for $C_{23}H_{29}F_4NO_5Na$ (ES$^+$)(+Na$^+$): 475.1889. Found: 498.1874. **HPLC** (Cellulose-1, hexane: ^{i}PrOH 97.0:3.0, flow rate 1.0 mL/min, λ = 254 nm, 25 °C) t_R(major) =7.260 min, t_R(minor) = 9.993 min, ee = 90%.

tert-butyl 4-(((_tert_-butoxycarbonyl)amino)methyl)-2-fluoro-2-(4-methoxybenzoyl)pent-4-enoate (6e)

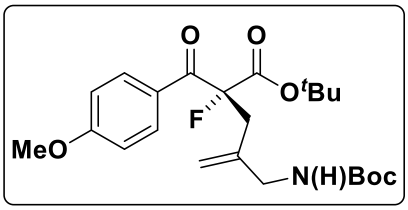

Following the General Procedure with *tert*-butyl 2-fluoro-3-(4-methoxyphenyl)-3-oxopropanoate (**5e**) (35 mg, 0.13 mmol), *tert*-butyl 5-methylene-2-oxo-1,3-oxazinane-3-carboxylate (**2**) (22 mg, 0.1 mmol), Pd(dba)$_2$ (2.9 mg, 0.005 mmol), *(R,R)*-Dach-phenyl Trost ligand (3.8 mg, 0.0055 mmol), t-BuOH (0.048 mL, 0.5 mmol) and DIPEA (0.021 mL, 0.12 mmol) in PhMe (1 mL) with FCC (10% EtOAc in petroleum ether) afforded *tert*-butyl 4-(((*tert*-butoxycarbonyl)amino)methyl)-2-fluoro-2-(4-methoxybenzoyl)pent-4-enoate as a yellow oil (**6e**) (22 mg, 51%).

^{1}H NMR (400 MHz, CDCl$_3$) **^{1}H NMR (400 MHz, CDCl$_3$)**: δ 8.04 (dd, *J* = 9.0, 1.5 Hz, 2H), 6.90 (d, *J* = 9.0 Hz, 2H), 5.13 (s, 1H), 5.04 (s, 1H), 4.82 (br, 1H), 3.86 (s, 3H), 3.77 (s, 2H), 3.08 (dd, *J* = 34.5, 15.5 Hz, 1H), 2.95 – 2.85 (m, 1H), 1.42 (s, 9H), 1.35 (s, 9H) ; **^{13}C NMR (101 MHz, CDCl$_3$)** δ 189.7 (d, *J* = 25.0 Hz), 166.5 (d, *J* = 26.0 Hz), 164.4, 156.1, 140.2, 132.6 (d, *J* = 5.5 Hz), 126.9 (d, *J* = 3.5 Hz), 116.1, 114.1, 99.6 (d, *J* = 199.5 Hz), 84.6, 79.6, 56.8, 45.9, 38.3 (d, *J* = 20.5 Hz), 28.7, 28.1 ; **^{19}F NMR (377 MHz, CDCl$_3$)**: δ -155.6 (dd, *J* = 34.5, 17.5 Hz). **FTIR:** ν_{max}/cm^{-1} (neat) 3004, 1710, 1420, 1359, 1221, 1092, 736; **HRMS (ESI$^+$):** calculated for $C_{23}H_{32}FNO_6Na$ (ES$^+$)(+Na$^+$): 460.2111. Found: 460.2127. **HPLC** (Cellulose-2, hexane: ^{i}PrOH 93.0:7.0, flow rate 1.0 mL/min, λ = 254 nm, 25 °C) t_R(major) =15.123 min, t_R(minor) =18.953 min, ee = 84%.

ethyl 2-acetyl-4-{[(_tert_-butoxycarbonyl)amino]methyl}-2-fluoropent-4-enoate (6f)

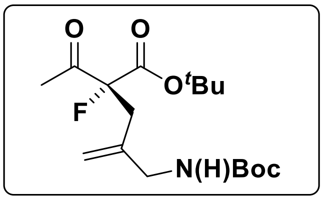

Following the General Procedure with *tert*-butyl 2-fluoro-3-oxobutanoate (**5f**) (23 mg, 0.13 mmol), *tert*-butyl 5-methylene-2-oxo-1,3-oxazinane-3-carboxylate (**2**) (22 mg, 0.1 mmol), $Pd(dba)_2$ (2.9 mg, 0.005 mmol), *(R,R)*-Dach-phenyl Trost ligand (3.8 mg, 0.0055 mmol), *t*-BuOH (0.048 mL, 0.5 mmol) and DIPEA (0.021 mL, 0.12 mmol) in PhMe (1 mL) with FCC (gradient from 20% Et_2O in petroleum ether) afforded ethyl 2-acetyl-4-{[(*tert*-butoxycarbonyl)amino]methyl}-2-fluoropent-4-enoate (**6f**) (27 mg, 79%).

^{1}H NMR (400 MHz, $CDCl_3$) δ 5.08 (s, 1H), 4.98 (s, 1H), 4.76 (br, 1H), 3.85 – 3.41 (m, 2H), 2.85 (dd, *J* = 27.5, 15.5 Hz, 1H), 2.71 (dd, *J* = 27.5, 15.5 Hz, 3H), 2.25 (s, 3H), 1.44 (s, 9H), 1.41 (s, 9H); **^{13}C NMR (101 MHz, $CDCl_3$)** δ 201.9 (d, *J* = 30.2 Hz), 164.9 (d, *J* = 25.4 Hz), 156.1, 139.9, 115.8, 100.4 (d, *J* = 199.0 Hz), 84.9, 79.7, 46.0, 37.3 (d, *J* = 20.0 Hz), 28.7, 28.1, 26.0; **^{19}F NMR (377 MHz, $CDCl_3$):** δ -162.4 – -162.5(m). **FTIR: ν_{max}/cm^{-1} (neat)** 2924, 1723, 1505, 1368, 1250, 1156, 910, 734; **HRMS (ESI$^+$):** calculated for $C_{17}H_{28}FNO_5Na$ (ES$^+$)(+Na$^+$): 368.1849. Found: 368.1926. **HPLC** (Cellulose-2, hexane: ^{i}PrOH 90:10, flow rate 1.0 mL/min, λ = 205 nm, 25 °C) t_R(major) = 5.643 min, t_R(minor) = 6.197 min, ee = 81%.

Interconversion of ethyl to *tert*-butyl ester (3e→6e): Evidence for homochirality across the ester substrates.

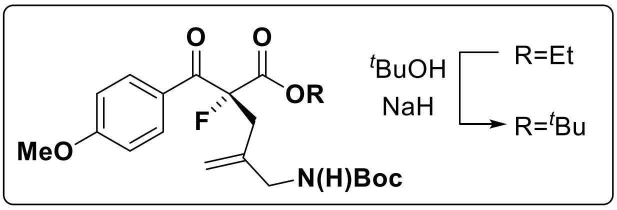

NaH (60% dispersion in mineral oil, 0.008 g, 0.20 mmol) and *t*BuOH (2 mL) were added successfully to ethyl 4-{[(*tert*-butoxycarbonyl)amino]methyl}-2-fluoro-2-(4-methoxybenzoyl)pent-4-enoate **(3e)** (0.033 g, 0.078 mmol), The resulting mixture was refluxed overnight. *t*BuOH was removed by vacuum and the mixture was purified by FCC (15% Et_2O in petroleum ether) afforded *tert*-butyl 4-(((*tert*-butoxycarbonyl)amino)methyl)-2-fluoro-2-(4-methoxybenzoyl)pent-4-enoate as a yellow oil (**6e**) (7 mg, 20%). This compound showed identical spectroscopic data and the same major enantiomer as **6e**.

Gram scale synthesis of compound (7)

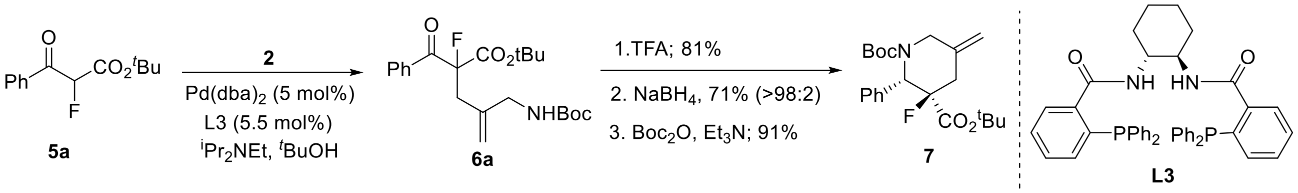

A flame-dried 250 mL round bottom flask was charged with Pd(dba)$_2$ (144 mg, 5 mol%), (*R,R*)-Dach-phenyl Trost ligand **L3** (190 mg, 5.5 mol%), *t*-BuOH (2.4 mL, 25 mmol), DIPEA (1.0 mL, 6 mmol) and *tert*-butyl 5-methylene-2-oxo-1,3-oxazinane-3-carboxylate **2** (1.06 g, 5 mmol) under nitrogen. Anhydrous PhMe (25 mL) was then added and the mixture stirred at 0 °C for 20 minutes. A solution of *tert*-butyl 2-fluoro-3-oxo-3-phenylpropanoate (**5a**) (1.54 g, 6.5 mmol) in PhMe (25 mL) was then added and the reaction stirred at 0 °C overnight. The reaction was then concentrated under vacuum and purified by flash silica column chromatography (15% diethyl ether in petroleum ether). The collected intermediate was then dissolved in CH_2Cl_2 (86 mL) and TFA (16.4 mL, 50 equiv.) was added to the mixture. After stirring at room temperature for 30 minutes, the mixture was basified to pH 8 using sat. $NaHCO_3$ before extraction with CH_2Cl_2. The combined organic layers were then dried over anhydrous magnesium sulfate and concentrated under vacuum to afford *tert*-butyl 3-fluoro-5-methylene-2-phenyl-3,4,5,6-tetrahydropyridine-3-carboxylate as a yellow oil (1.00 g, 71% yield over two steps).

To a solution of *tert*-butyl 3-fluoro-5-methylene-2-phenyl-3,4,5,6-tetrahydropyridine-3-carboxylate (1.00 g, 3.46 mmol) in MeOH (17.3 mL) under nitrogen at 0 °C was added $NaBH_4$ (0.523 mg, 13.84 mmol) and the resulting mixture warmed to room temperature and stirred overnight. The reaction was then diluted with $NaHCO_3$ and extracted with EtOAc. The combined organic layers were dried over anhydrous $MgSO_4$, concentrated under vacuum and the residue purified by FCC (20% EtOAc in 40-60 petroleum ether) to afford *tert*-butyl (2*S*,3*R*)-3-fluoro-5-methylene-2-phenylpiperidine-3-carboxylate as a yellow oil (0.845 g). To a solution of *tert*-butyl 3-fluoro-5-methylene-2-phenylpiperidine-3-carboxylate (0.845 g, 1.12 mmol) in THF (17.0 mL) under nitrogen was added Et_3N (0.90 mL, 6.37 mmol) and di-*tert*-butyl dicarbonate (1.39 g, 6.38 mmol) and the resulting mixture stirred at room temperature overnight. The reaction was then diluted with H_2O and extracted with DCM. The combined organic layers were then dried over anhydrous $MgSO_4$, concentrated under vacuum and purified by FCC (4% EtOAc in 40-60 petroleum ether) to afford di-*tert*-butyl (2*S*,3*R*)-3-fluoro-5-methylene-2-phenylpiperidine-1,3-dicarboxylate (**7**) as a colourless oil (0.978 g, 53% yield over four steps). The product was isolated as a mixture of rotamers. The stereochemistry of **7** was assigned on the basis of the known stereochemistry of the corresponding ethyl ester.[1]

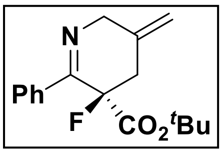

^{1}H NMR (400 MHz, CDCl$_3$) δ 7.71 – 7.65 (m, 2H), 7.42 – 7.31 (m, 3H), 5.08 (s, 1H), 5.01 (s, 1H), 4.63 (dd, *J* = 20.5, 5.5 Hz, 1H), 4.50 (dd, *J* = 20.5, 5.5 Hz, 1H), 2.95 – 2.78 (m, 2H), 1.25 (s, 9H). **^{13}C NMR (101 MHz, CDCl$_3$)** δ168.0 (d, *J* = 27.0 Hz), 161.0 (d, *J* = 18.5 Hz), 137.4, 136.9 (d, *J* = 3.5 Hz), 130.0, 128.4, 127.4 (d, *J* = 2.5 Hz), 112.4, 90.6 (d, *J* = 195.0 Hz), 84.0, 56.1, 39.5 (d, *J* = 24.1 Hz), 27.8; **^{19}F NMR (377 MHz, CDCl$_3$):** δ -146.0 – -146.2 (m) **FTIR:** ν_{max}/cm^{-1} (neat) 2978, 1749, 1636, 1447, 1369, 1321, 1260, 1076, 733, 704cm^{-1}; **HRMS (ESI$^+$):** calculated for: $C_{17}H_{21}FNO_2$ (ES$^+$)(+H$^+$): 290.1556. Found: 290.1569.

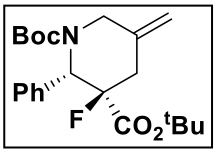

^{1}H NMR (400 MHz, CDCl$_3$) δ 7.41 – 7.12 (m, 5H), 5.42 (br, 1H), 5.07 (s, 1H), 4.95 (s, 1H), 4.43 (br, 1H), 3.82 (br, 1H), 3.12 (dd, *J* = 43.0 16.0 Hz, 1H), 2.76 (br, 1H), 1.34 (s, 9H), 1.17 (s, 9H); **^{13}C NMR (101 MHz, CDCl$_3$)** δ 167.1 (d, *J* = 24.0 Hz), 154.9, 141.1, 137.0, 128.7, 128.5, 128.2, 113.9, 94.8 (d, *J* = 185.5 Hz), 83.2, 80.5, 61.3, 45.7, 34.8 (d, *J* = 88.0 Hz), 28.4, 27.5; **^{19}F NMR (377 MHz, CDCl$_3$)** δ -144.6 (br), -145.6 (br); **FTIR:** ν_{max}/cm^{-1} (neat) 2977, 2932, 1740, 1694, 1455, 1392, 1367, 1284, 1251, 1156, 1106, 1061, 972, 894, 839, 765, 700; **HRMS (ESI+):** calculated for $C_{22}H_{30}FNO_4Na$ (ES+)(+Na$^+$): 414.2057. Found: 414.2079.

di-*tert*-butyl (*6S,7R*)-1,1,7-trifluoro-6-phenyl-5-azaspiro[2.5]octane-5,7-dicarboxylate (8)[3]

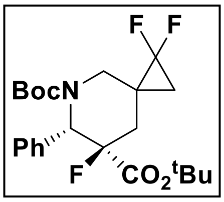

TMSCF$_3$ (0.037 mL, 0.25 mmol) and NaI (0.008 g, 0.05 mmol) were added in one portion to a solution containing di-*tert*-butyl 3-fluoro-5-methylene-2-phenylpiperidine-1,3-dicarboxylate **7** (0.039 g, 0.1mmol) in THF (2 mL). The resulting mixture was heated at reflux for 4 hours. After 4 hours, additional TMSCF$_3$ (0.037 mL, 0.25 mmol) was added and the resulting mixture was heated at reflux overnight. The residue was dissolved in CH_2Cl_2, washed with water, sodium thiosulfate (0.1 M), brine, dried over Na_2SO_4, filtered, and the solvent was removed under reduced pressure. The residue was diluted with H_2O (5 mL) and $Na_2S_2O_3$ (sat) and extracted with DCM (3 x 5 mL). The combined organic layers were then extracted with brine (15 mL) dried over anhydrous $MgSO_4$, concentrated under vacuum and purified by FCC (20% Et_2O in 40-60 petroleum ether) to afford di-*tert*-butyl (*6S,7R*)-1,1,7-trifluoro-6-phenyl-5-azaspiro[2.5]octane-5,7-dicarboxylate as a colourless solid (22 mg, 50%; 4:1 mixture of diastereomers). Each diastereomer was isolated as a mixture of rotamers. **FTIR (both isomers):** ν_{max}/cm^{-1} (neat) 2979, 2929, 1739, 1694, 1476, 1456, 1412, 1393, 1368, 1312, 1274, 1254, 1207, 1154, 1071, 1003, 972, 894, 839, 754, 700; **HRMS (ESI+) (both isomers):** calculated for $C_{23}H_{30}F_3NO_4Na$ (ES+)(+Na$^+$): 464.2025. Found: 464.2047.

Major isomer

^{1}H NMR (400 MHz, CDCl$_3$) δ 7.43 – 7.28 (m, 5H), 5.91 (br, 0.5H), 5.60 (br, 0.5H), 3.86 (br, 0.5H), 3.69 (br, 0.5H), 3.15 (br, 1H), 2.83 (ddd, *J* = 41.5, 15.5, 2.2 Hz, 1H), 1.95 (br, 1H), 1.44 (s, 9H), 1.26-1.19 (m, 11H); **^{13}C NMR (101 MHz, CDCl$_3$) :** δ 167.2 (d, *J* = 22.5 Hz), 156.4, 136.1, 129.1, 128.9, 128.7, 114.3 (t, *J* = 288.0 Hz), 94.2 (d, *J* = 184.5 Hz), 83.8, 81.2, 61.7, 41.3, 30.9, 29.9, 28.6, 27.7, 20.8; **^{19}F NMR (377 MHz, CDCl$_3$)** δ -138.4—-138.9 (m, 1F CF_2), -140.3—-140.9 (m, 1F CF_2),-146.9—-148.0 (m, 1F).

Minor isomer

^{1}H NMR (400 MHz, CDCl$_3$) δ 7.38 – 7.32 (m, 5H), 5.91 (br, 0.5H), 5.52 (br, 0.5H), 4.00 (br, 0.5H), 3.84 (br, 0.5H), 3.27 (br, 1H), 2.75 (ddd, *J* = 43.0, 15.5, 6.5 Hz, 1H), 2.17 (br, 1H), 1.45 (s, 9H), 1.26-1.21 (m, 11H); **^{13}C NMR (101 MHz, CDCl$_3$) :** δ 167.3 (d, *J* = 23.0 Hz), 154.8, 136.4, 129.1, 128.9, 128.6, 114.3 (t, *J* = 288.0 Hz), 93.1 (d, *J* = 181.0 Hz) 83.6, 81.2, 60.9, 40.7 (d, *J* = 52.5 Hz), 30.6, 30.0, 28.6, 27.7, 19.4; **^{19}F NMR (377 MHz, CDCl$_3$)** δ-137.2—-137.9 (m, 1F CF_2), -140.0—-140.8 (m, 1F CF_2), -149.2—-150.5 (m, 1F).

di-*tert*-butyl (6S,7R)-7-fluoro-6-phenyl-1-oxa-5-azaspiro[2.5]octane-5,7-dicarboxylate (9)

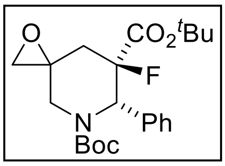

To a solution of di-*tert*-butyl 3-fluoro-5-methylene-2-phenylpiperidine-1,3-dicarboxylate **7** (0.058 g, 0.15 mmol) in CH_2Cl_2 (4.5 mL) was added the solution of m-CPBA in CH_2Cl_2 (0.75 mL) dropwise at 0 °C under N_2. The resulting mixture was stirred at room temperature for 20 hours. The mixture was quenched by $Na_2S_2O_5$ and sat. $NaHCO_3$ and stirred for 10 minutes before extraction with CH_2Cl_2 (3 x 15 mL). The combined organic layers were dried over $MgSO_4$, concentrated under vacuum and purified by FCC (10%-20% Et_2OAc in 40-60 petroleum ether) to afford di-*tert*-butyl (*6S,7R*)-7-fluoro-6-phenyl-1-oxa-5-azaspiro[2.5]octane-5,7-dicarboxylate (39 mg, 64%; 3:2 mixture of diastereomers). The diastereomers could be isolated individually after careful chromatography allowing their isolation as a colorless oil and white solid, respectively. **FTIR (both isomers):** ν_{max}/cm^{-1} (neat) 2978, 2932, 1741, 1699, 1395, 1369, 1312, 1289, 1253, 1157, 1099, 893, 808, 754, 701; **HRMS (ESI+) (both isomers):** calculated for $C_{22}H_{30}F_3NO_4Na$ (ES+)(+Na$^+$): 430.2006. Found: 430.2036.

Major isomer

^{1}H NMR (400 MHz, CDCl$_3$) δ 7.49-7.30 (m, 5H), 5.84 (br, 0.5H), 5.54 (br , 0.5H), 3.69 (br 1H), 3.43 (br, 1H), 3.00 (d, *J* = 15.5 Hz, 1H), 2.89 (d, *J* = 15.5 Hz, 1H), 2.80 (br, 1H), 2.09 – 1.97 (m, 1H), 1.45 (s, 9H), 1.23 (s, 9H); **^{13}C NMR (101 MHz, CDCl$_3$) :** δ 166.7 (d, *J* = 23.0 Hz), 155.0, 136.0, 129.0, 128.9, 128.7, 96.0 (d, *J* = 183.0 Hz), 83.8, 81.2, 61.5, 55.9, 44.8, 35.2 (br), 30.0, 28.6, 27.7; **^{19}F NMR (377 MHz, CDCl$_3$)** δ-144.2 (br), -145.3 (br).

Minor isomer

^{1}H NMR (400 MHz, CDCl$_3$) δ 7.35 (br, 5H), 5.68 (br, 1H), 3.74 (br, 1H), 3.61 (br, 1H), 3.03 (dd, *J* = 43.0, 16.0 Hz, 1H), 2.78 (d, *J* = 4.0 Hz, 1H), 2.74 (d, *J* = 4.0 Hz, 1H), 2.01 – 1.89 (m, 1H), 1.45 (s, 9H), 1.24 (s, 9H); **^{13}C NMR (101 MHz, CDCl$_3$)**167.1 (d, *J* = 23.0 Hz), 156.3, 136.3, 128.9, 128.8, 128.6, 94.4 (d, *J* = 186.5 Hz), 83.6, 81.2, 60.9, 49.4, 45.6, 34.5 (br), 30.0, 28.6, 27.7; **^{19}F NMR (377 MHz, CDCl$_3$)** δ-145.2(br), -146.4 (br).

tert-butyl (2*S*,3*R*)- 3-fluoro-3-(hydroxymethyl)-5-methylene-2-phenylpiperidine-1-carboxylate (10)

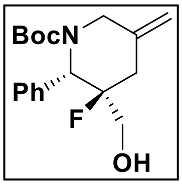

To a suspension of $LiAlH_4$ (7.6 mg, 0.2 mmol) in THF (2 mL) under nitrogen was added di-*tert*-butyl 3-fluoro-5-methylene-2-phenylpiperidine-1,3-dicarboxylate **7** (78 mg, 0.2 mmol) in THF (2 mL) and the resulting mixture stirred at room temperature for 22 hours. Et_2O (1.3 mL) was added to the resulting mixture at 0 °C and H_2O (0.026 mL) was then added dropwise and the reaction stirred for 15 minutes at room temperature before the addition of anhydrous $MgSO_4$. After 15 minutes the reaction mixture was filtered through celite and the volatiles removed under vacuum. Purification by FCC (20% EtOAc in 40-60 petroleum ether) afforded *tert*-butyl (2*S*,3*R*)-3-fluoro-3-(hydroxymethyl)-5-methylene-2-phenylpiperidine-1-carboxylate as a colourless oil (36 mg, 56%; >98:2).

^{1}H NMR (400 MHz, CDCl$_3$): δ 7.43-7.30 (m, 5H), 5.37 (br, 1H), 5.22 (s, 1H), 5.02 (s, 1H), 4.42 (d, *J* = 15.0 Hz, 1H), 4.26 (d, *J* = 15.0 Hz, 1H), 3.51 (d, *J* = 6.0 Hz, 1H), 3.46 (d, *J* = 6.0 Hz, 1H), 2.65 (q, *J* = 16.0 Hz, 1H), 2.51 (d, *J* = 16.0 Hz, 1H) 1.93 (br, 1H), 1.33 (s, 9H); **^{13}C NMR (101 MHz, CDCl$_3$):** δ 154.9, 141.1, 137.0, 129.2, 128.9, 128.4, 114.0, 97.5 (d, *J* = 176.0 Hz), 80.6, 66.2 (d, *J* = 21.5 Hz), 60.7, 47.2, 33.7 (d, *J* = 22.5 Hz), 28.7; **^{19}F NMR (377 MHz, CDCl$_3$)** δ -154.1 (dd, *J* = 33.0, 19.5 Hz); **FTIR:** ν_{max}/cm^{-1} (neat) 3445, 2926, 1676, 1457, 1378, 1162, 1053, 889 cm^{-1}; **HRMS (ESI+)**: calculated for $C_{18}H_{24}FNO_3Na$ (ES+)(+Na$^+$): 344.1638. Found: 344.1661. **HPLC** (Cellulose-1, hexane: ^{i}PrOH 98.0:2.0, flow rate 1.0 mL/min, λ = 254 nm, 25 °C) t_R(major) = 23.890 min, t_R(minor) =30.800 min, ee = 91%.

di-*tert*-butyl (*2S,3R*)-3-fluoro-5-(hydroxymethyl)-2-phenylpiperidine-1,3-dicarboxylate (11)

Borane Reduction method:

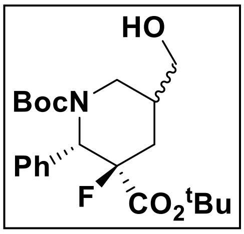

To a solution of BH_3·THF complex (1.0 M in THF, 2.25 ml, 2.25 mmol) was added di-*tert*-butyl 3-fluoro-5-methylene-2-phenylpiperidine-1,3-dicarboxylate **7** (0.058 g, 0.15 mmol) in THF (0.75 ml) dropwise at 0 °C under N_2. The resulting mixture was stirred at room temperature for 20 h and quenched with 3.0 mL of 2 M aqueous NaOH solution (dropwise) and 0.90 mL of 30% aqueous H_2O_2 solution. After stirring for 1.5 hours, the aqueous layer was extracted with diethyl ether (3 x 15 mL). The combined organic layers were dried over $MgSO_4$, concentrated

under vacuum and purified by FCC (20% Et_2OAc in 40-60 petroleum ether) to afford di-*tert*-butyl (2S,3R)-3-fluoro-5-(hydroxymethyl)-2-phenylpiperidine-1,3-dicarboxylate as a colourless oil (35 mg, 58%; 5:1 mixture of diastereomers). The product was isolated as a mixture of rotamers. **^{1}H NMR (400 MHz, $CDCl_3$) (Major diastereomer):** δ 7.42 – 7.27 (m, 5H), 5.79-5.62 (m, 1H), 4.05 (d, *J* = 14.5 Hz, 1H), 3.81-3.78 (m, 1H), 3.55 (br, 1H), 2.99 (dd, *J* = 14.5, 2.5 Hz, 1H), 2.60 (ddd, *J* = 49.5, 16.0, 6.5 Hz, 1H), 2.22-2.09 (m, 2H), 1.50 (s, 9H), 1.17 (s,9H); **^{13}C NMR (101 MHz, $CDCl_3$)** δ 167.6, 167 4, 136.0 (d, *J* = 10.5 Hz), 128.8, 128.7, 128.5, 94.8 (d, *J* = 181.0 Hz), 83.2, 81.3, 64.2 (d, *J* = 219.0 Hz), 60.9, 37.8, 34.9, 29.6 (d, *J* = 22.5 Hz), 28.7, 27.6; **^{19}F NMR (377 MHz, $CDCl_3$)** δ -145.6 – -146.0 (m), -146.9 – -147.1 (m). **FTIR:** ν_{max}/cm^{-1} (neat) 3450, 2921, 1740, 1674, 1369, 1161, 738 cm^{-1}; **HRMS (ESI+)**: calculated for $C_{22}H_{32}FNO_5Na$ (ES+)(+Na^+): 432.2162 Found: 432.2182.

9-BBN Reduction method:

To a solution of 9-BBN (0.5 M in THF, 4.5 ml, 2.25 mmol), di-*tert*-butyl 3-fluoro-5-methylene-2-phenylpiperidine-1,3-dicarboxylate **7** (0.058g, 0.15 mmol) in THF (0.75 ml) was added dropwise at 0 °C under N_2. The resulting mixture was stirred at room temperature for 20 h and quenched with 3.0 mL of 2 M aqueous NaOH solution (dropwise) and 0.90 mL of 30% aqueous H_2O_2 solution. After stirring for 1.5 hours, the aqueous layer was extracted with diethyl ether (3 x 15 mL). The combined organic layers were dried over $MgSO_4$, concentrated under vacuum and purified by FCC (20% Et_2OAc in 40-60 petroleum ether) to afford di-*tert*-butyl (2*S*,3*R*)-3-fluoro-5-(hydroxymethyl)-2-phenylpiperidine-1,3-dicarboxylate as a colorless oil (35 mg, 58%; 1:2 mixture of diastereomers). The product was isolated as a mixture of rotamers. Spectra are too complex to allow clean assignments but are displayed on pages S65-S67.

NMR Spectra

ethyl 2-benzoyl-4-{[(*tert*-butoxycarbonyl)amino]methyl}-2-fluoropent-4-enoate (3a)

^{1}H NMR, CDCl$_3$, 400 MHz

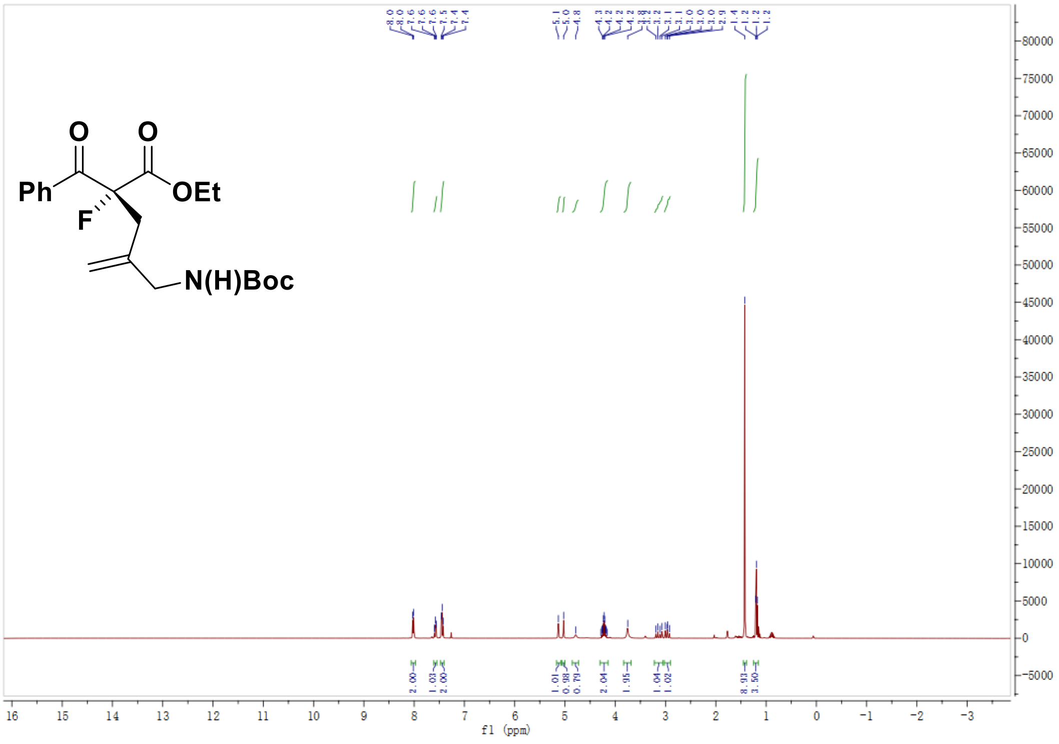

^{19}F NMR, CDCl$_3$, 377 MH

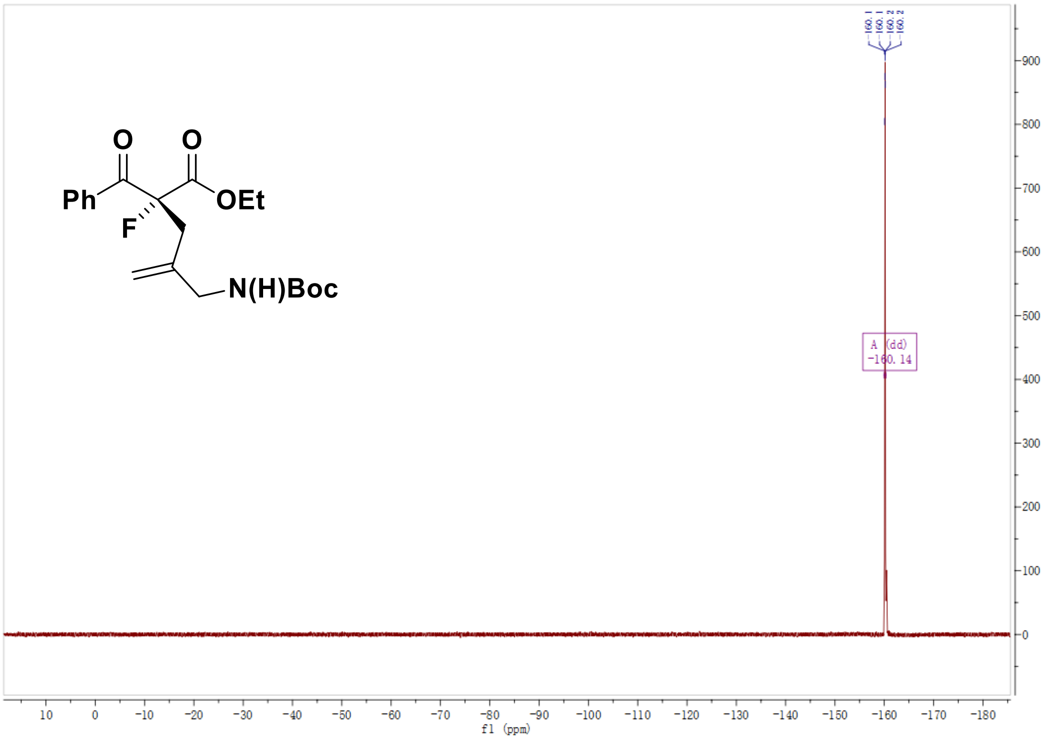

^{13}C NMR, $CDCl_3$, 101 MHz

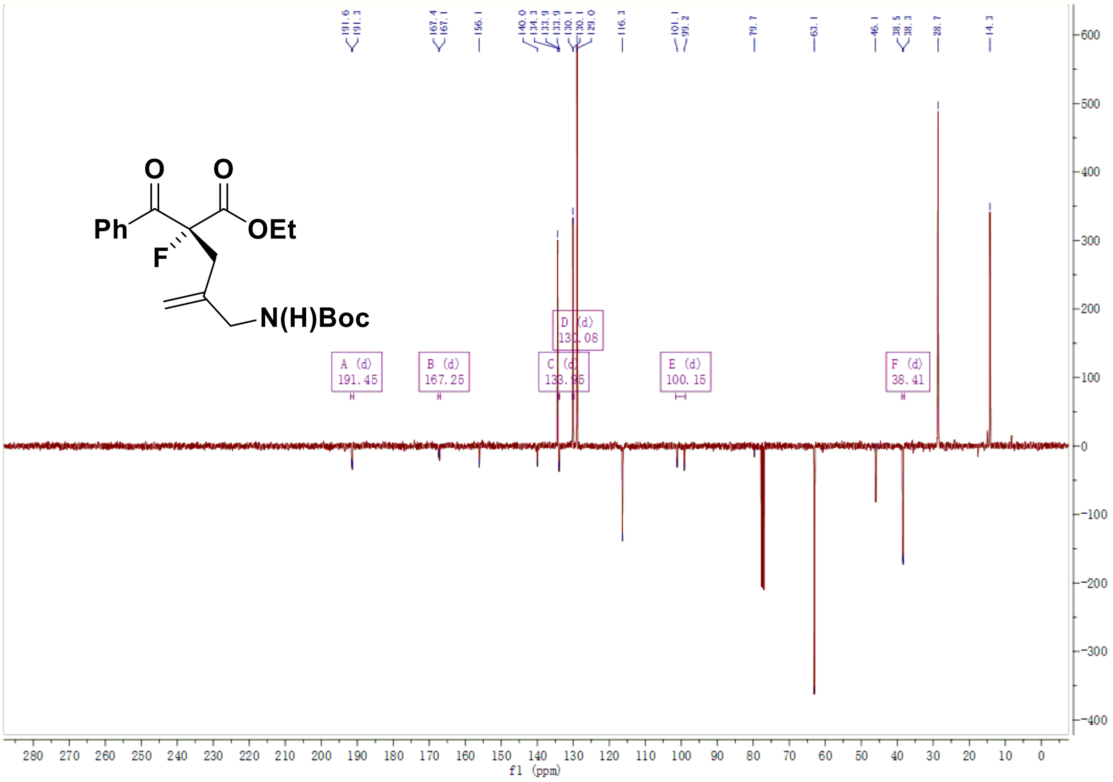

ethyl 4-{[(*tert*-butoxycarbonyl)amino]methyl}-2-fluoro-2-(4-chloromethylbenzoyl)pent-4-enoate (3b)

^{1}H NMR, CDCl$_3$, 400 MHz

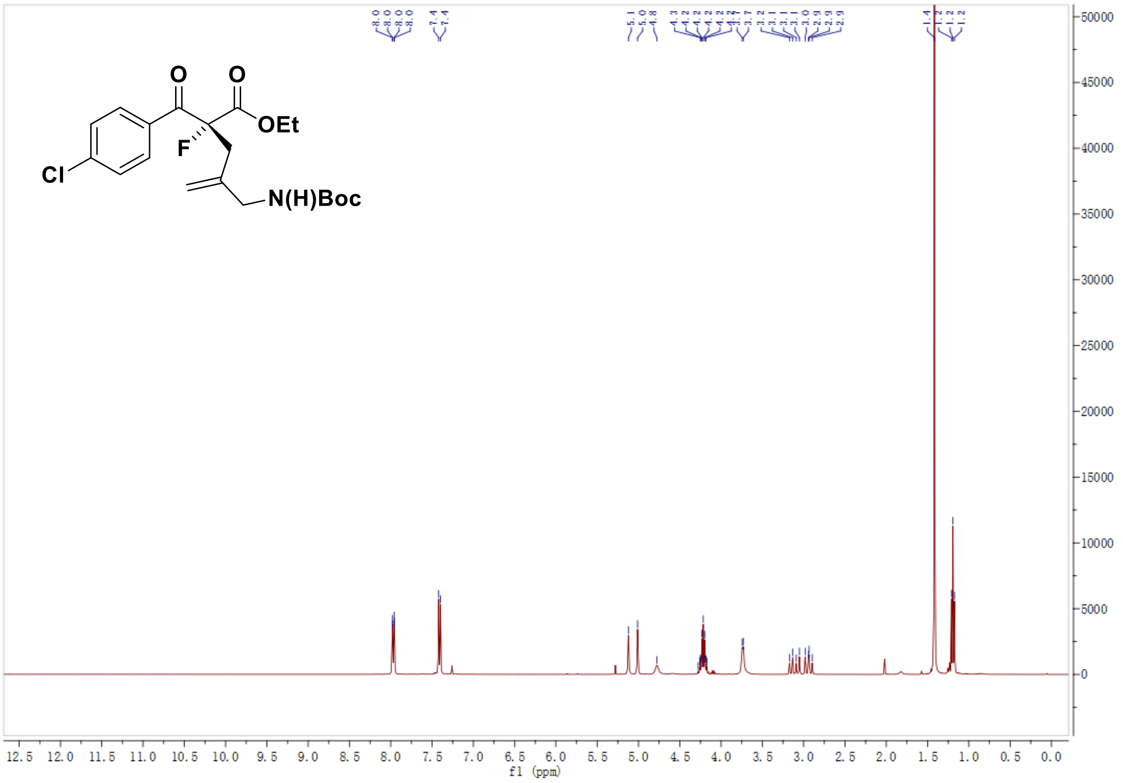

^{19}F NMR, CDCl$_3$, 377 MH

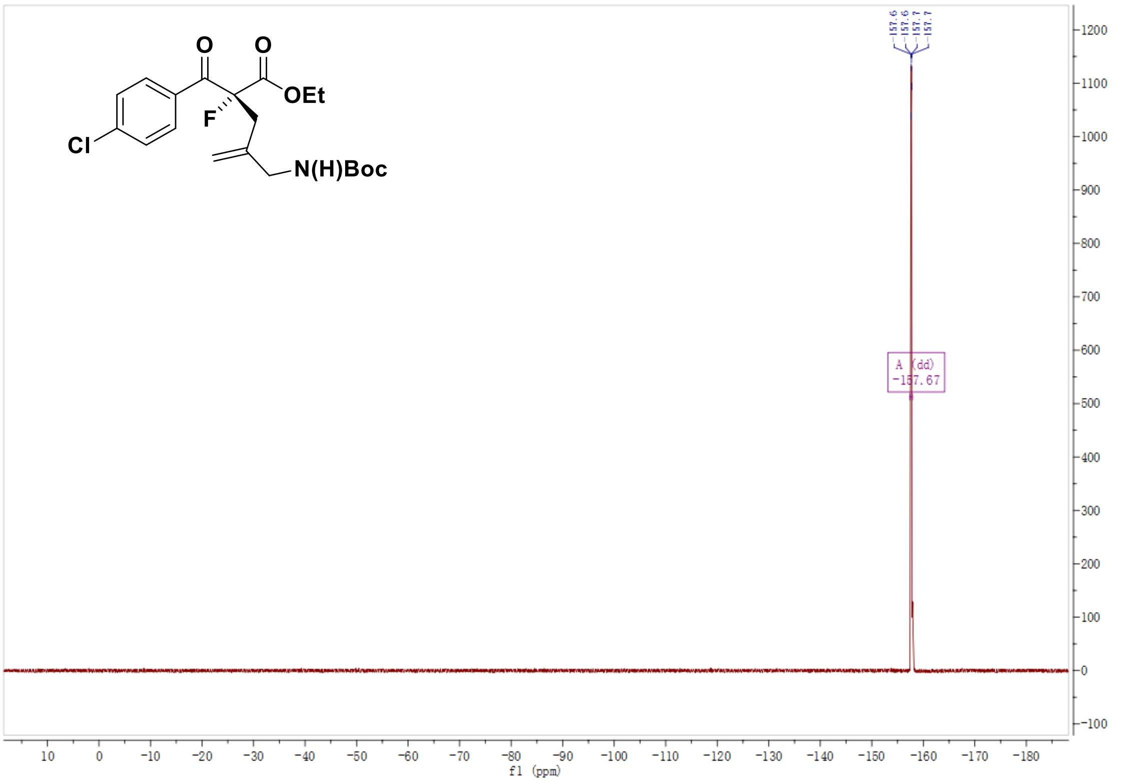

^{13}C NMR, $CDCl_3$, 101 MHz

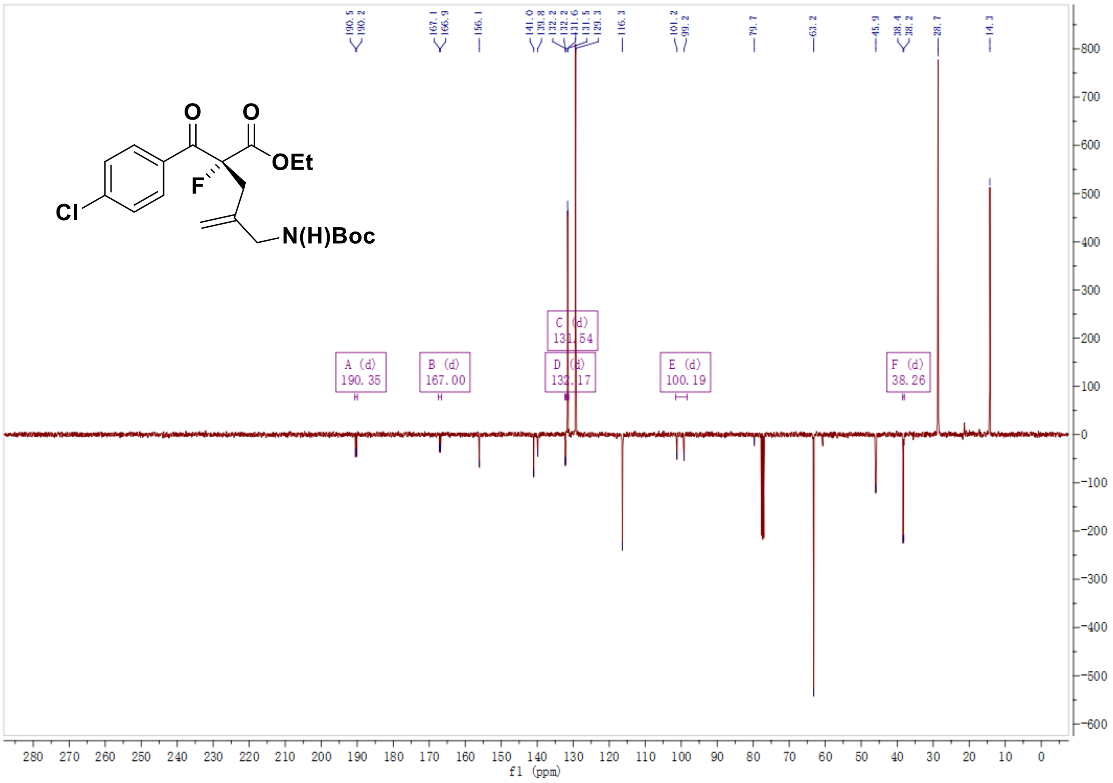

ethyl 4-{[(*tert*-butoxycarbonyl)amino]methyl}-2-fluoro-2-(4-methylbenzoyl)pent-4-enoate (3c)

^{1}H NMR, CDCl$_3$, 400 MHz

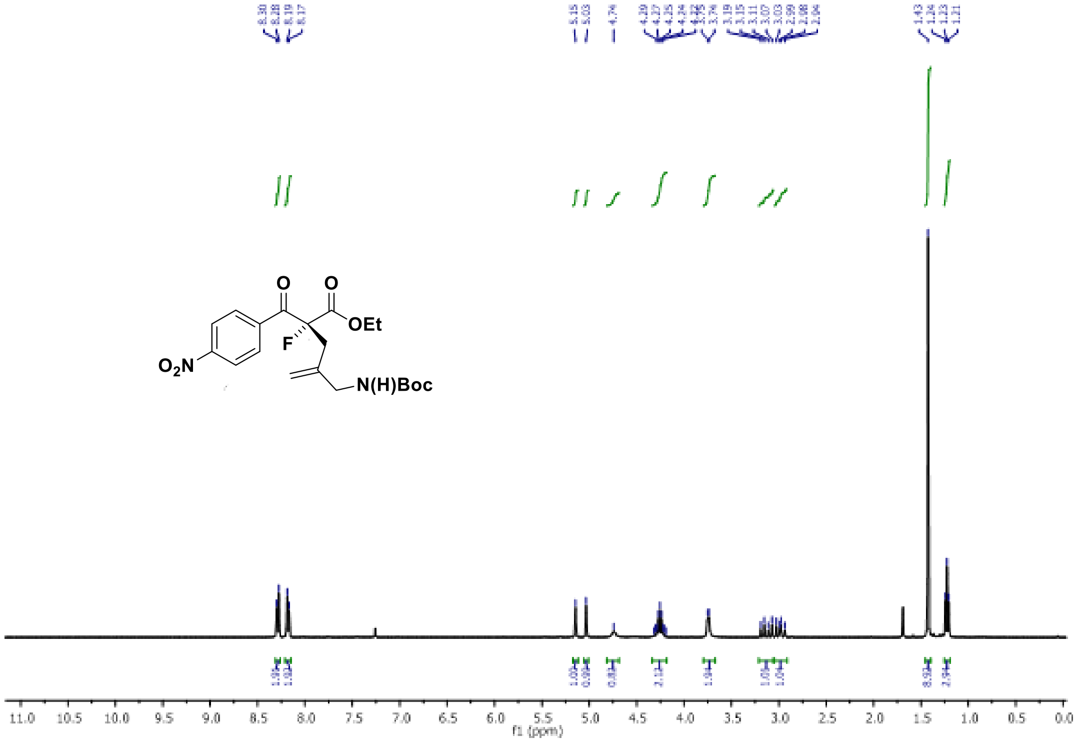

^{19}F NMR, CDCl$_3$, 377 MH

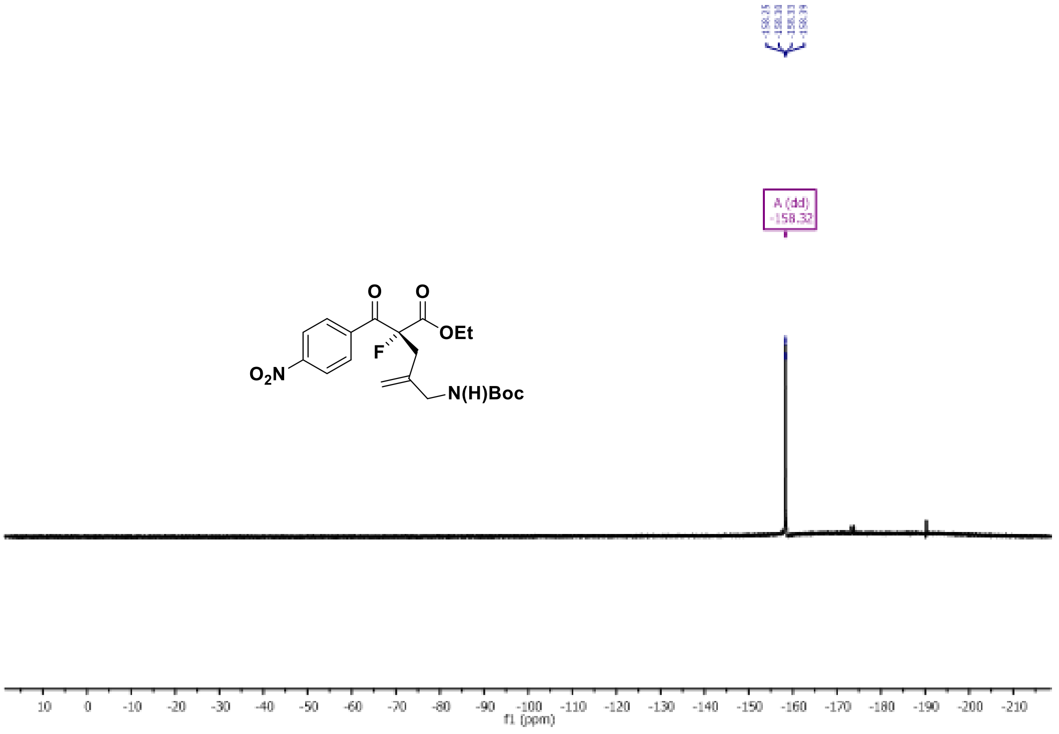

^{13}C NMR, $CDCl_3$, 101 MHz

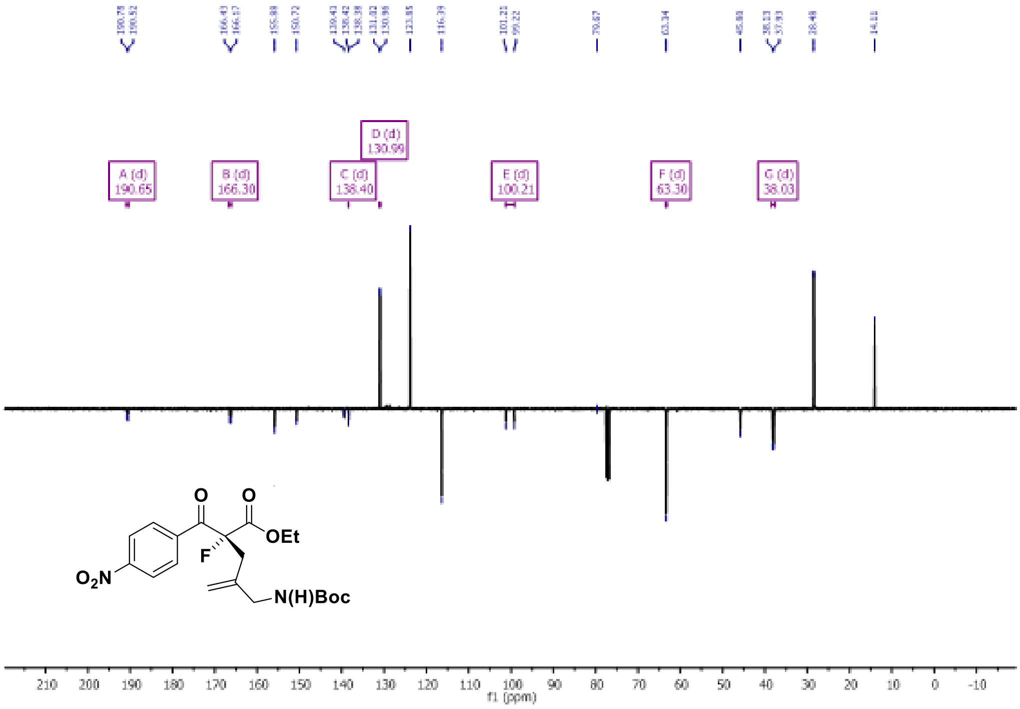

ethyl 4-{[(*tert*-butoxycarbonyl)amino]methyl}-2-fluoro-2-(4-trifluoromethylbenzoyl)pent-4-enoate (3d)

^{1}H NMR, $CDCl_3$, 400 MHz

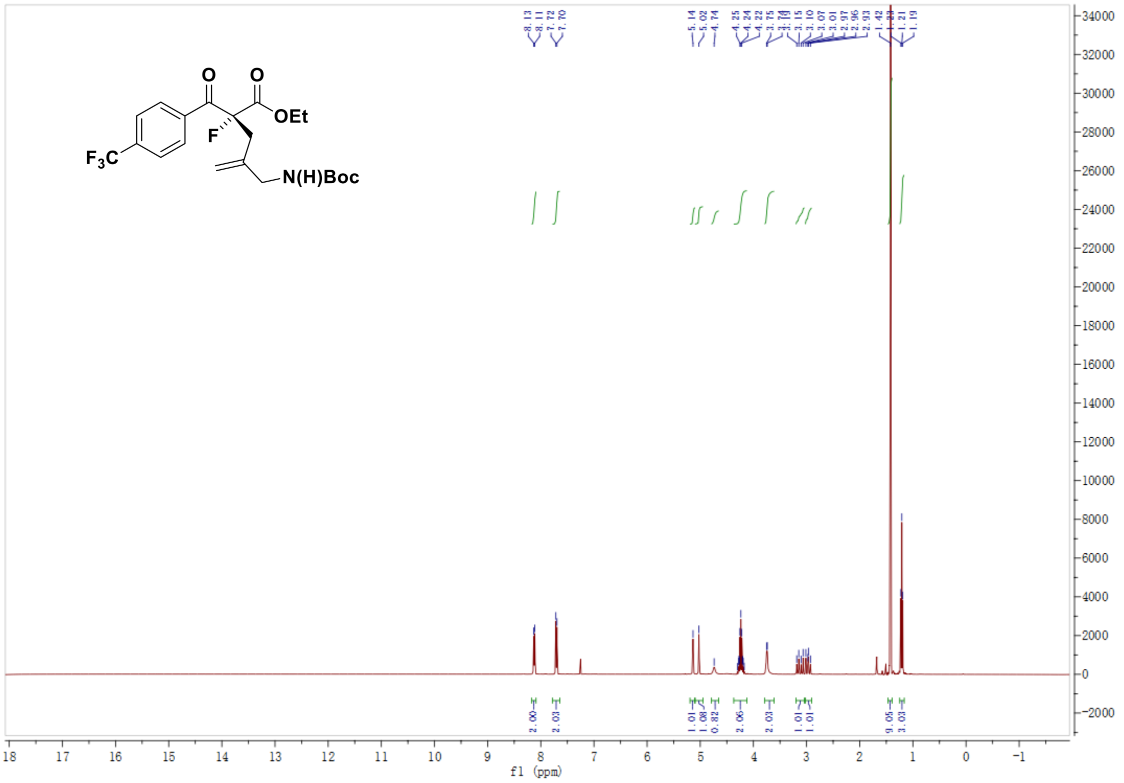

^{19}F NMR, $CDCl_3$, 377 MH

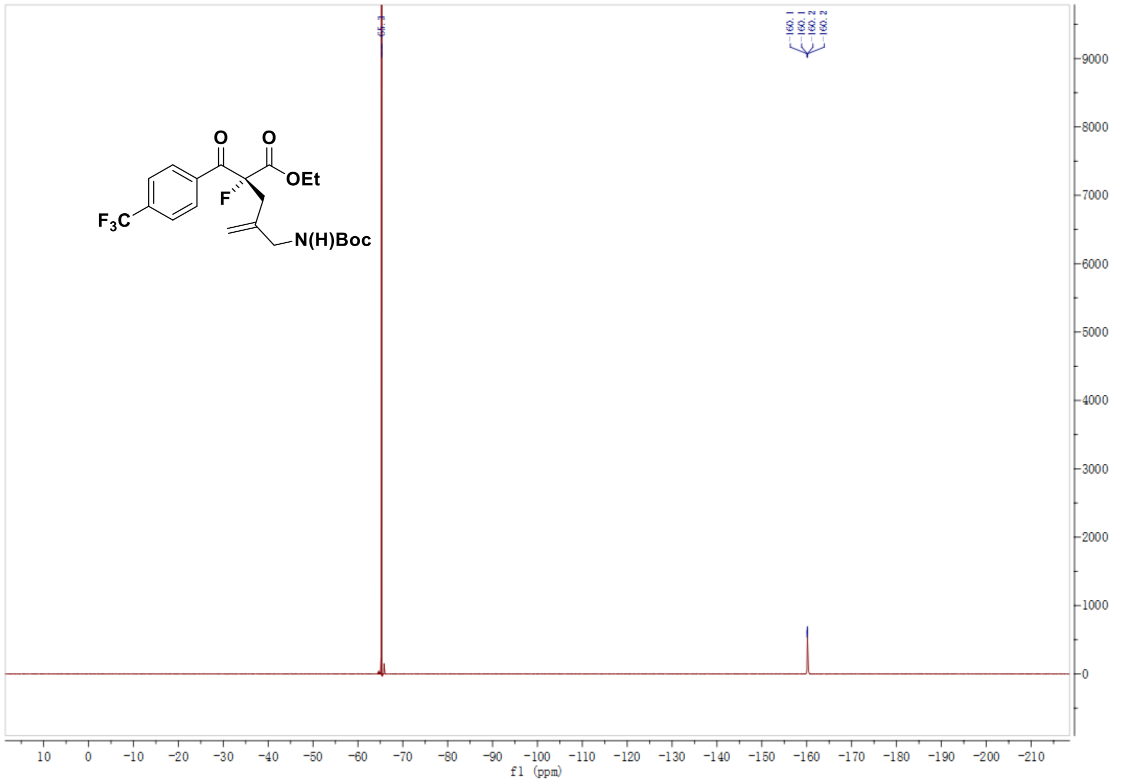

^{13}C NMR, $CDCl_3$, 101 MHz

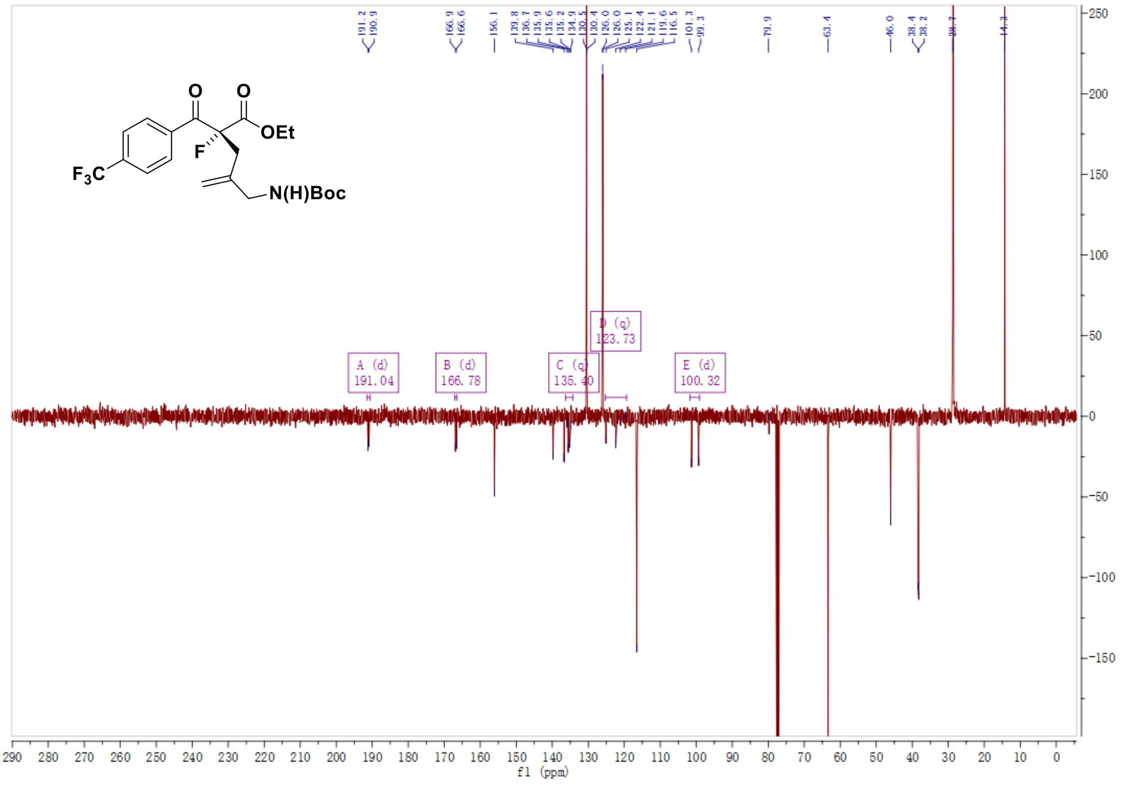

ethyl 4-{[(*tert*-butoxycarbonyl)amino]methyl}-2-fluoro-2-(4-methoxybenzoyl)pent-4-enoate (3e)

^{1}H NMR, CDCl$_3$, 400 MHz

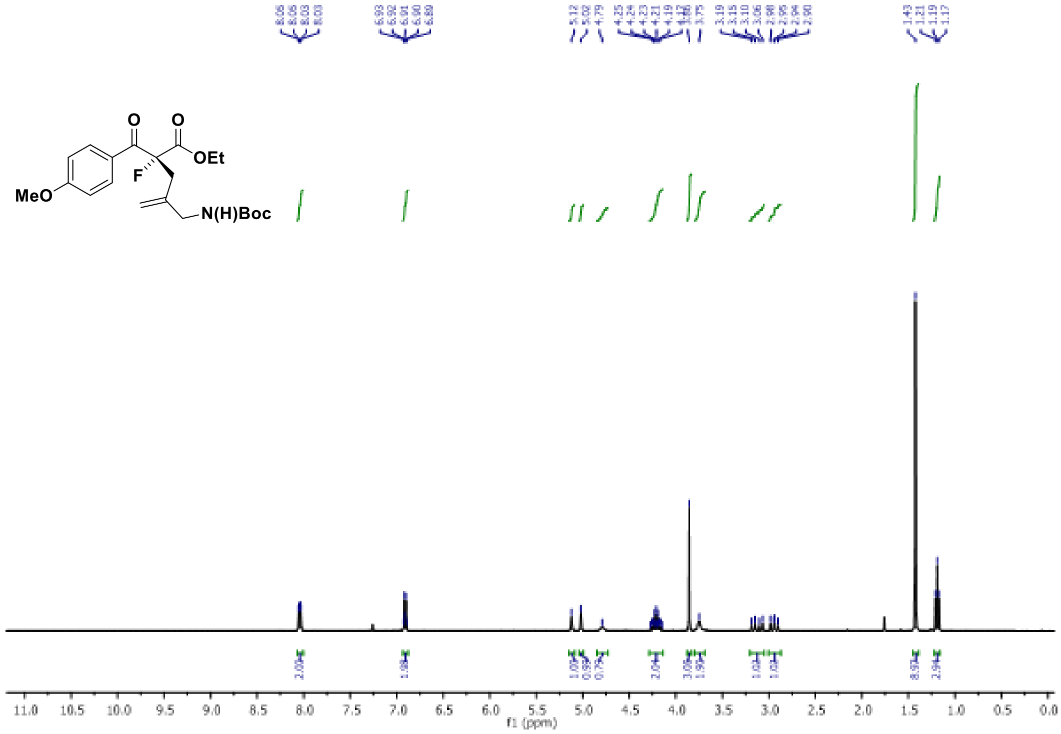

^{19}F NMR, CDCl$_3$, 377 MH

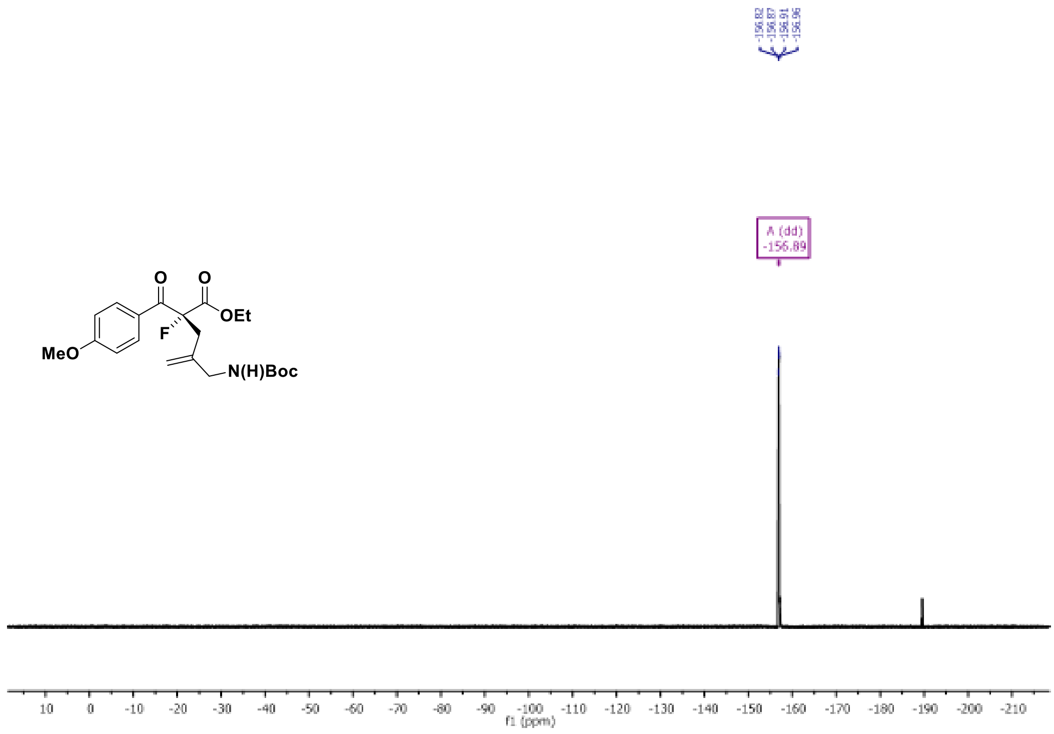

^{13}C NMR, $CDCl_3$, 101 MHz

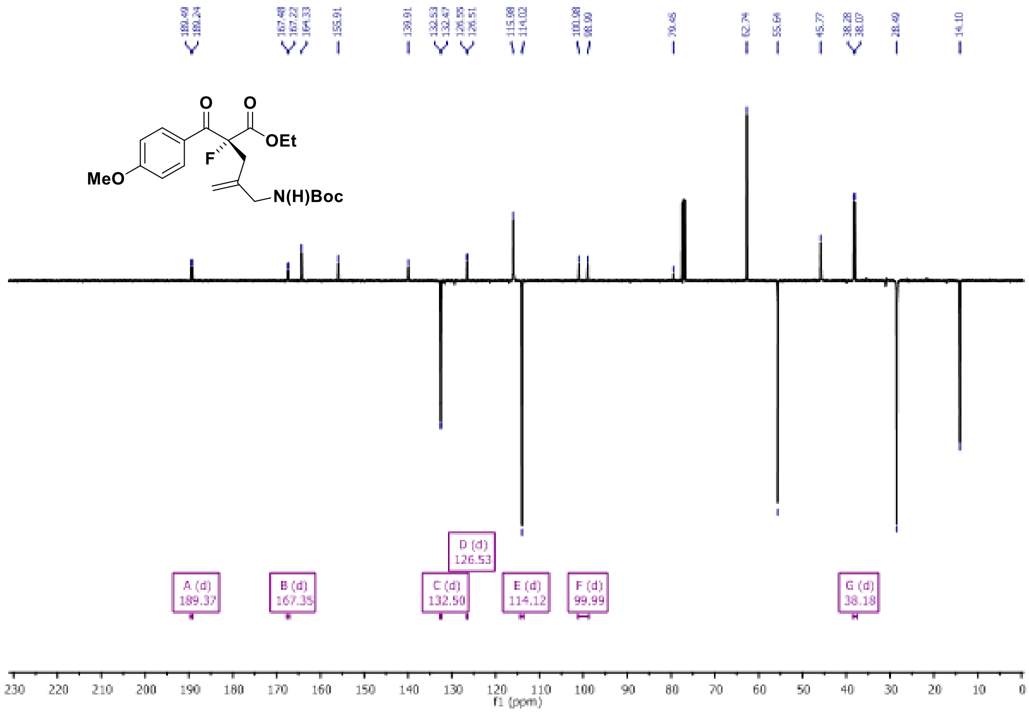

ethyl 4-{[(*tert*-butoxycarbonyl)amino]methyl}-2-fluoro-2-(4-methylbenzoyl)pent-4-enoate (3f)

^{1}H NMR, CDCl$_3$, 400 MHz

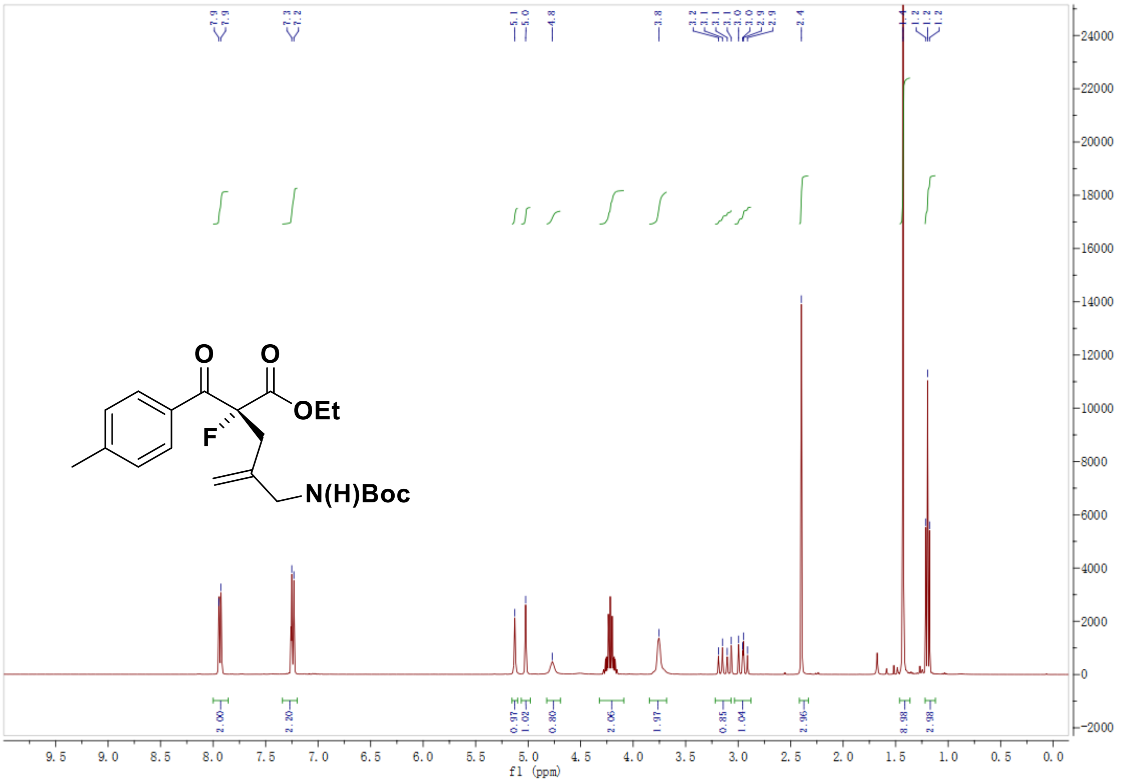

^{19}F NMR, CDCl$_3$, 377 MH

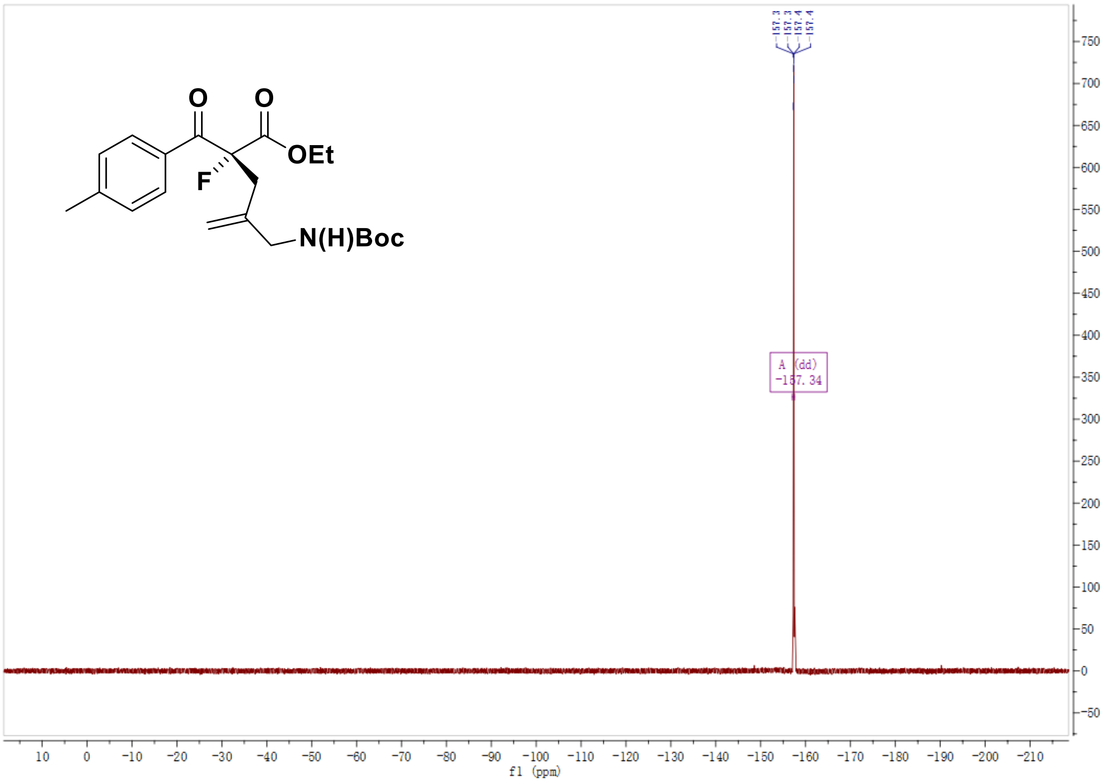

^{13}C NMR, $CDCl_3$, 101 MHz

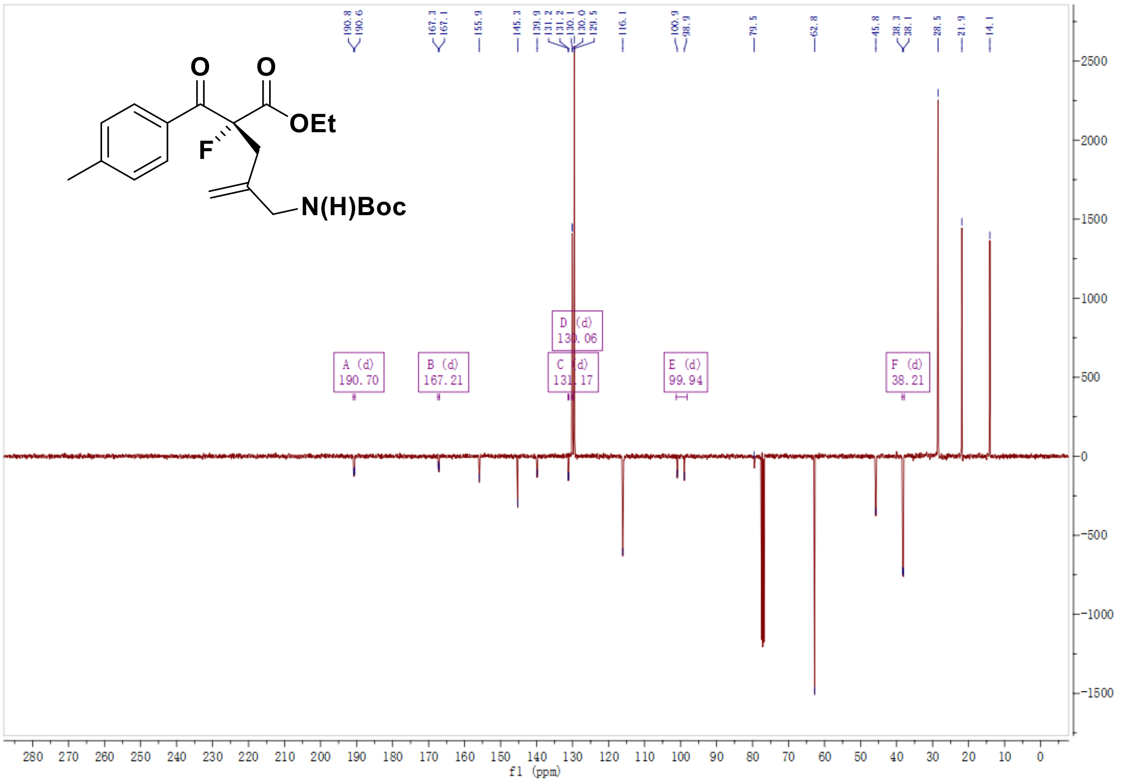

ethyl 4-{[(*tert*-butoxycarbonyl)amino]methyl}-2-fluoro-2-(4-methylbenzoyl)pent-4-enoate (3g)

^{1}H NMR, CDCl$_3$, 400 MHz

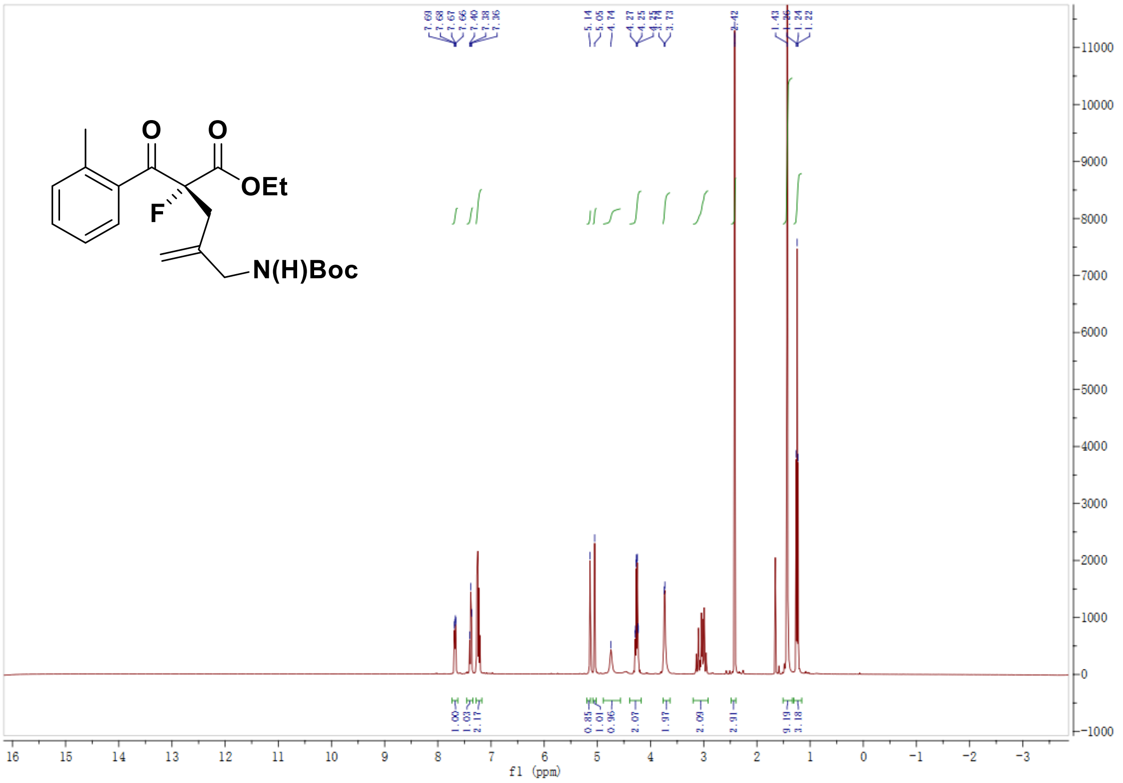

^{19}F NMR, CDCl$_3$, 377 MH

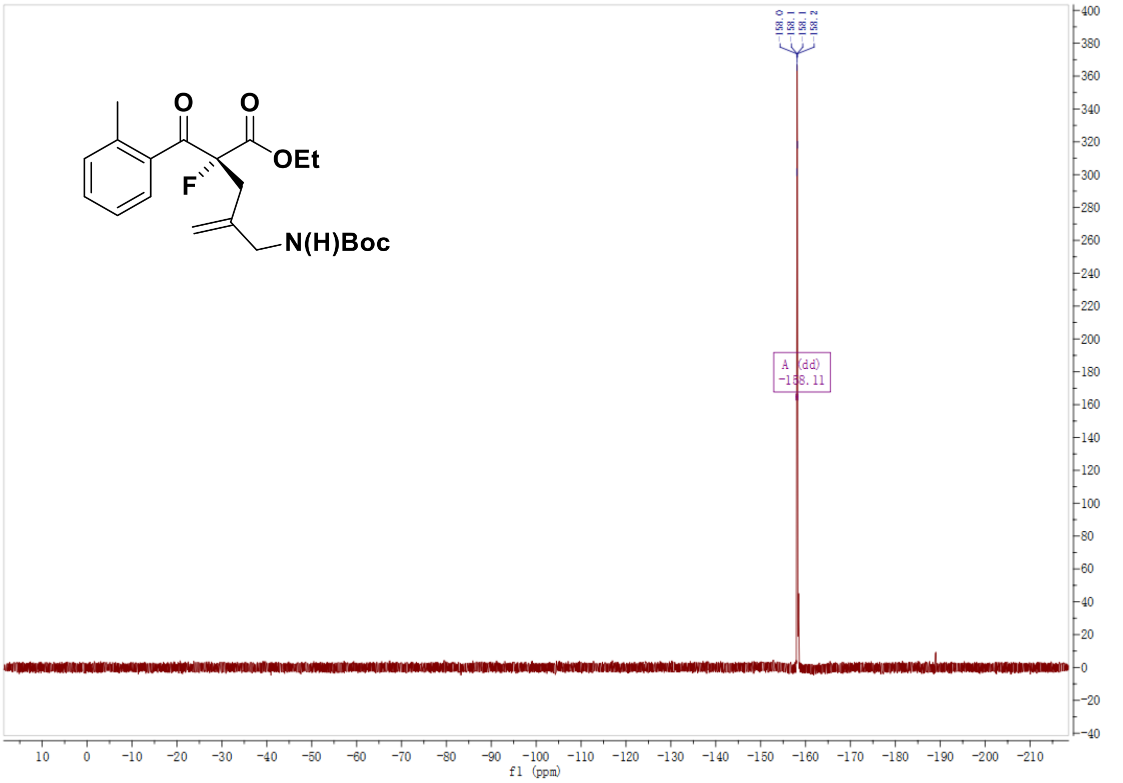

^{13}C NMR, $CDCl_3$, 101 MHz

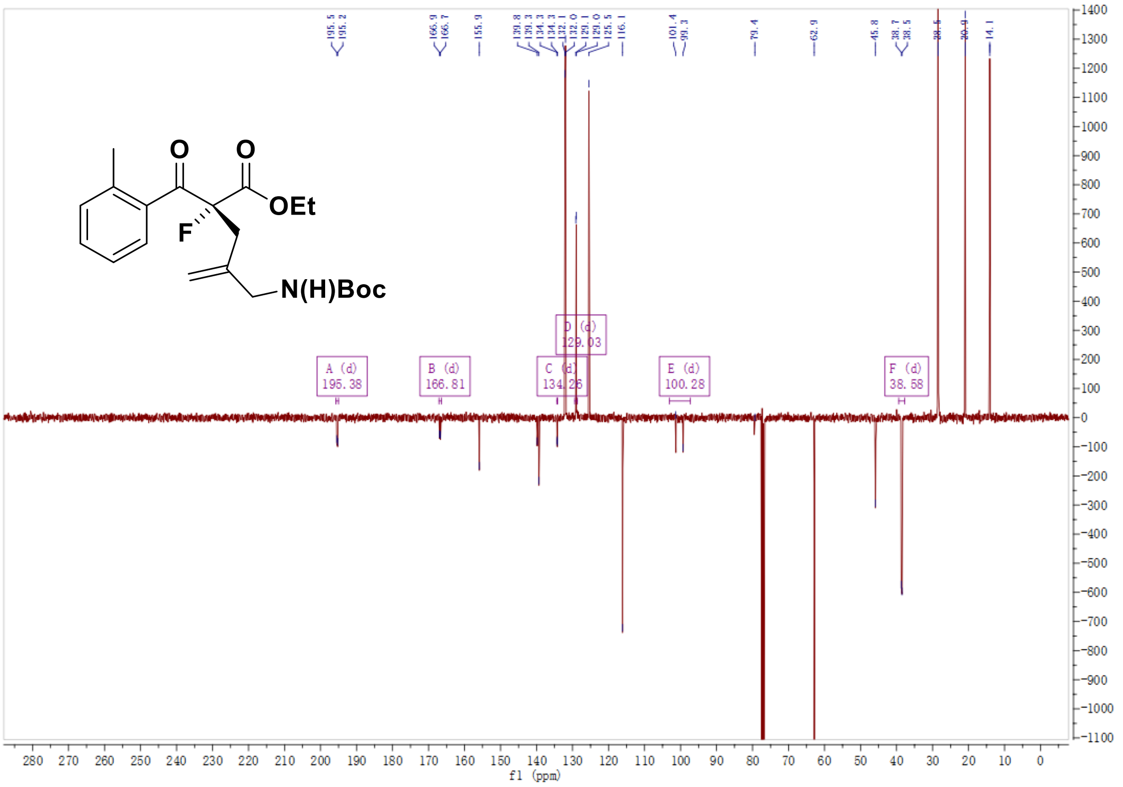

ethyl 4-(((*tert*-butoxycarbonyl)amino)methyl)-2-fluoro-2-(furan-2-carbonyl)pent-4-enoate (3h)

^{1}H NMR, CDCl$_3$, 400 MHz

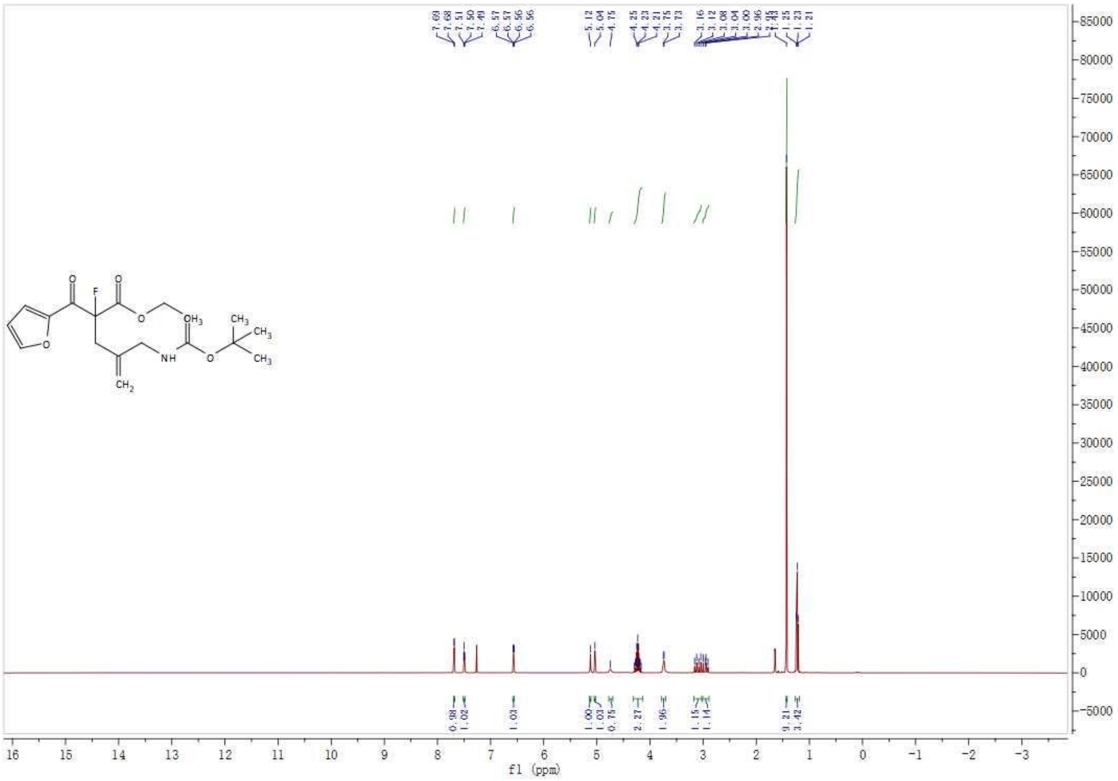

^{19}F NMR, CDCl$_3$, 377 MHz

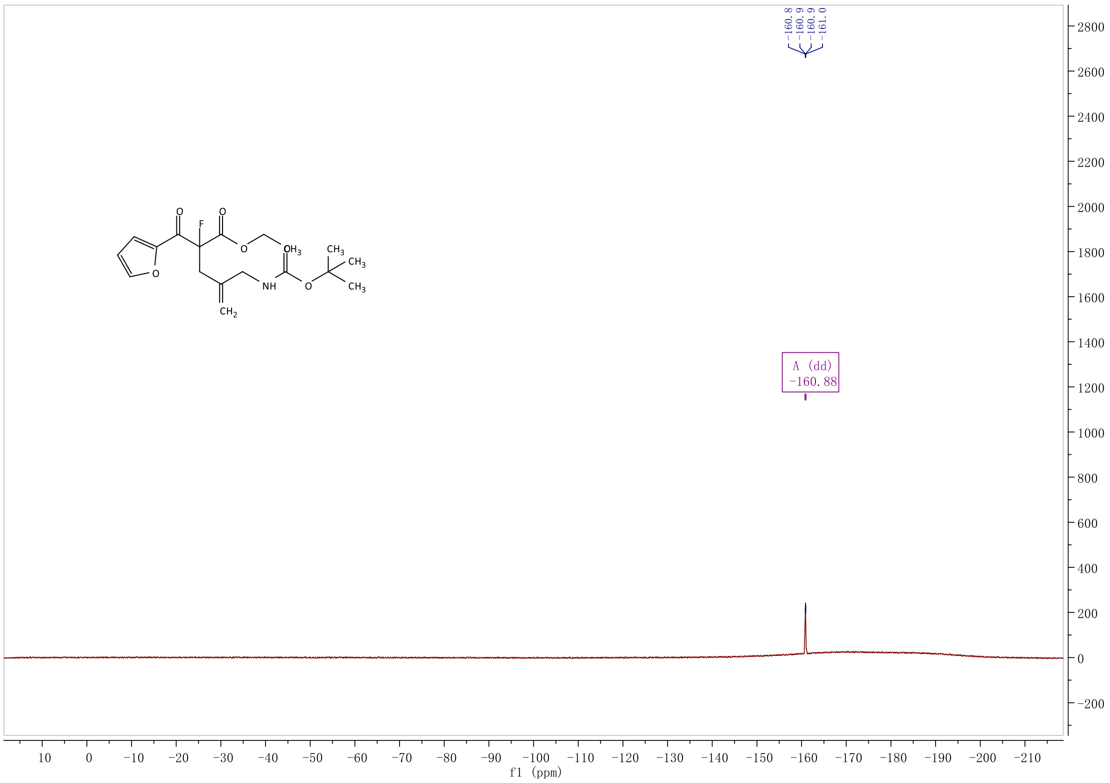

^{13}C NMR, $CDCl_3$, 101 MHz

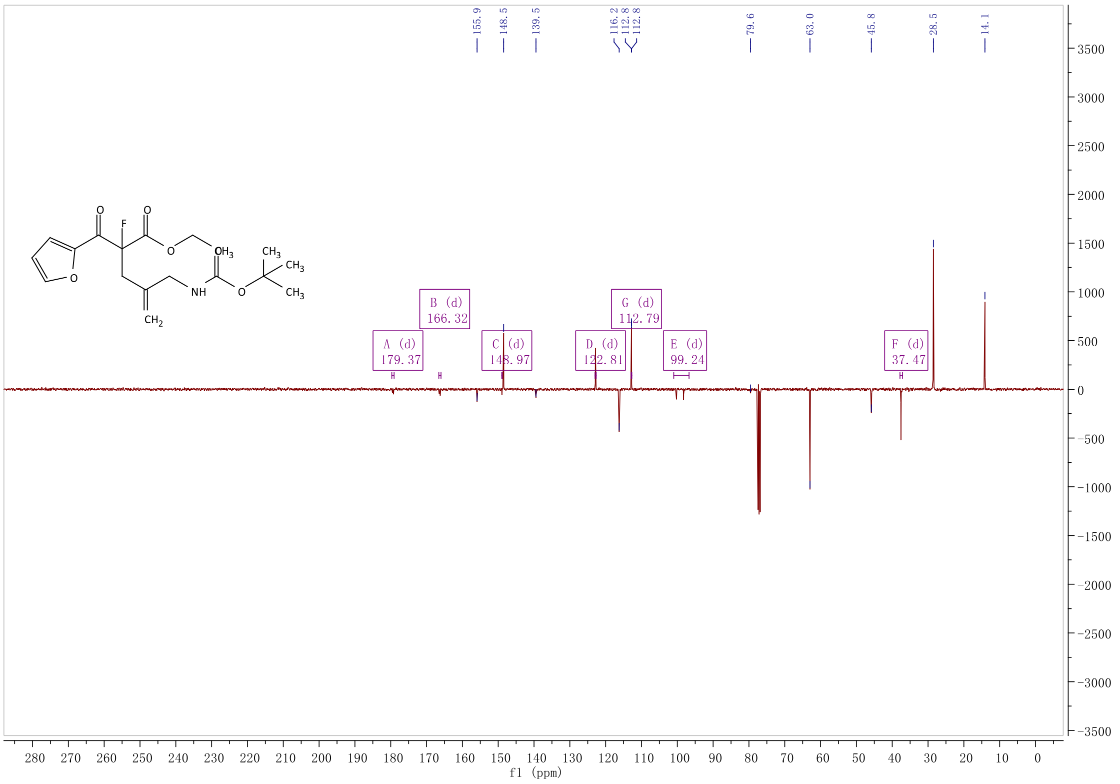

ethyl 2-(2-naphthoyl)-4-(((*tert*-butoxycarbonyl)amino)methyl)-2-fluoropent-4-enoate (3i)

^{1}H NMR, $CDCl_3$, 400 MHz

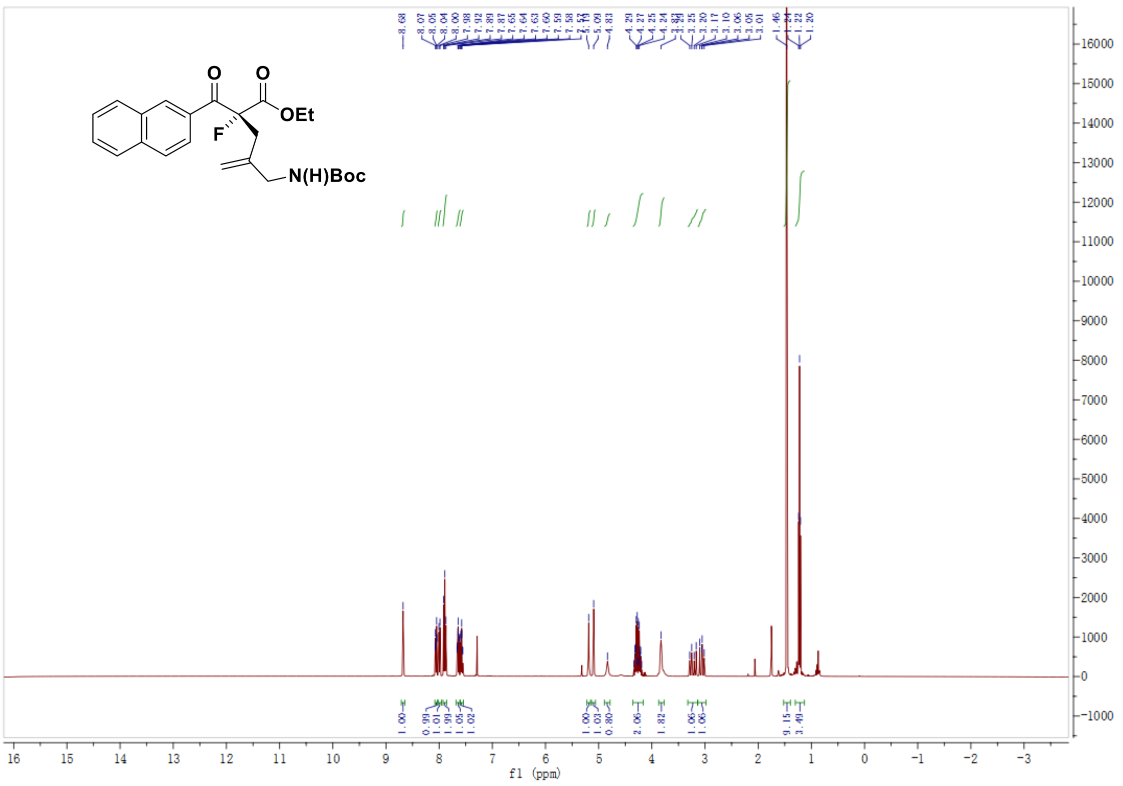

^{19}F NMR, $CDCl_3$, 377 MHz

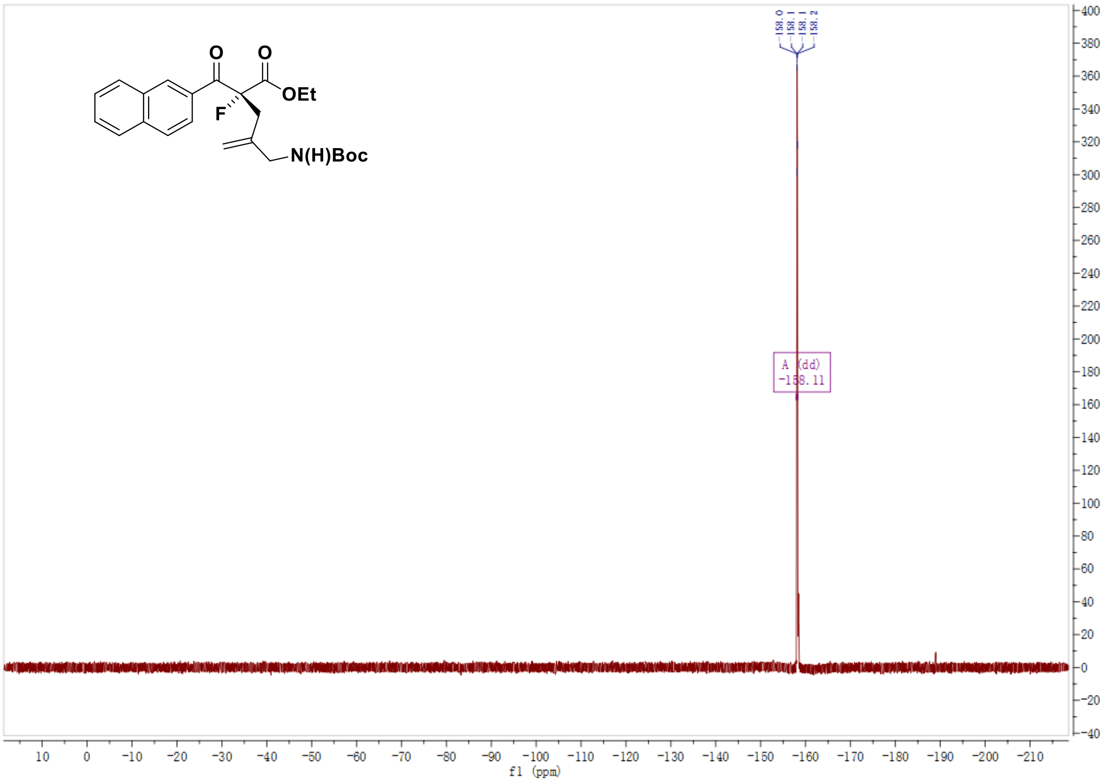

^{13}C NMR, $CDCl_3$, 101 MHz

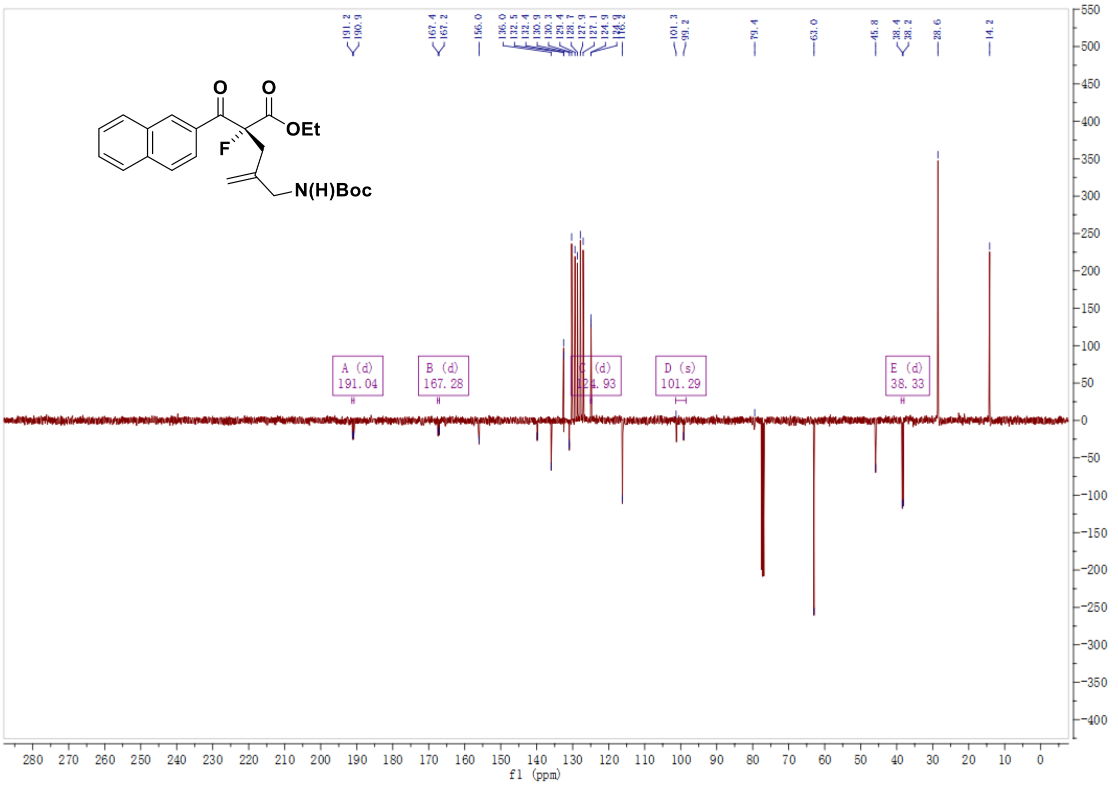

ethyl 2-acetyl-4-{[(*tert*-butoxycarbonyl)amino]methyl}-2-fluoropent-4-enoate (3j)

^{1}H NMR, $CDCl_3$, 400 MHz

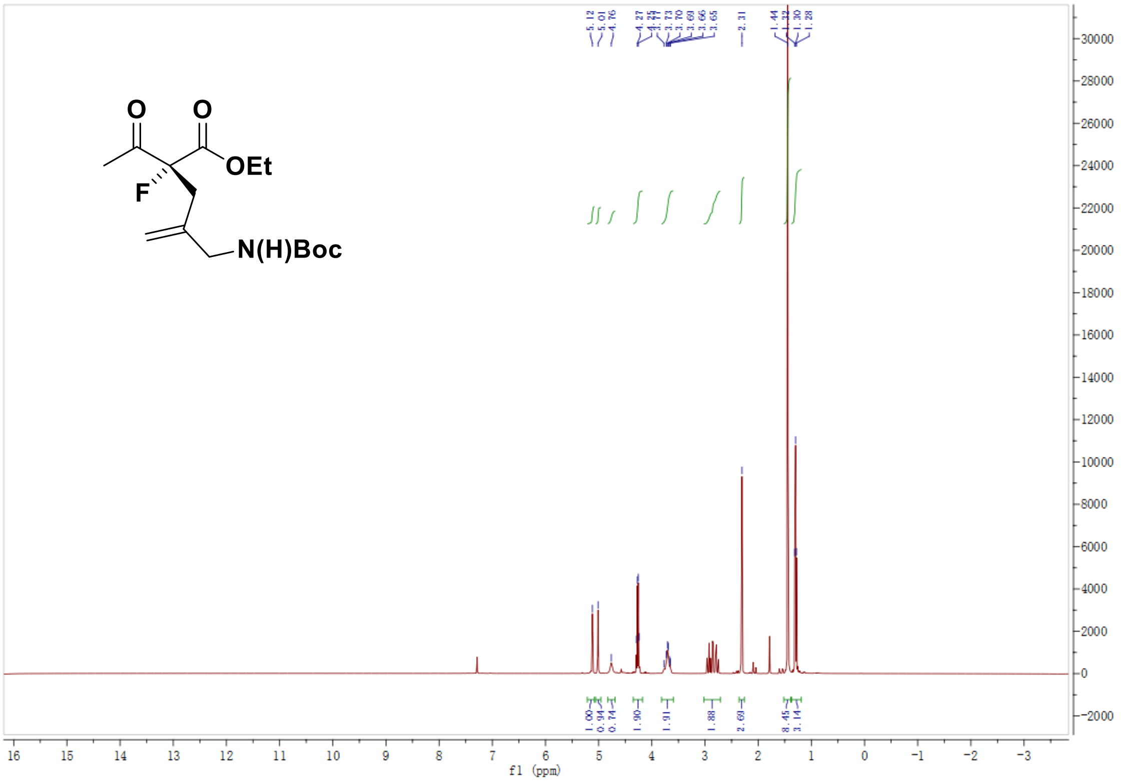

^{19}F NMR, $CDCl_3$, 377 MHz

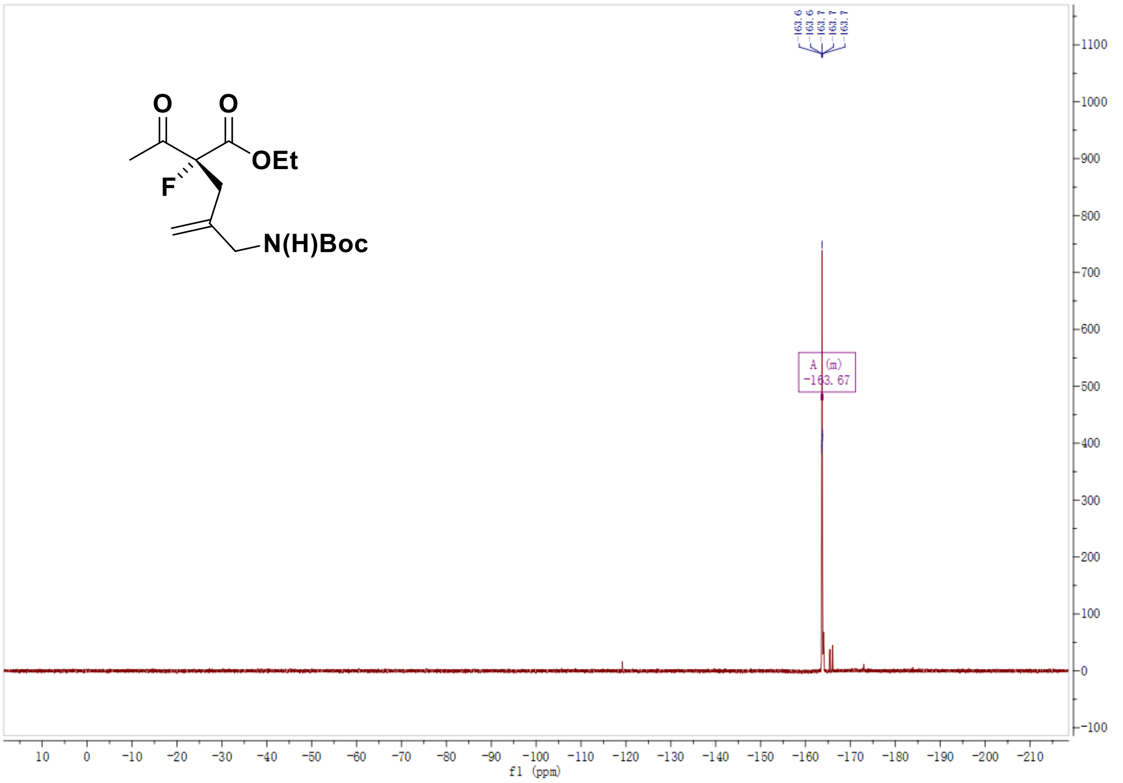

^{13}C NMR, CDCl$_3$, 101 MHz

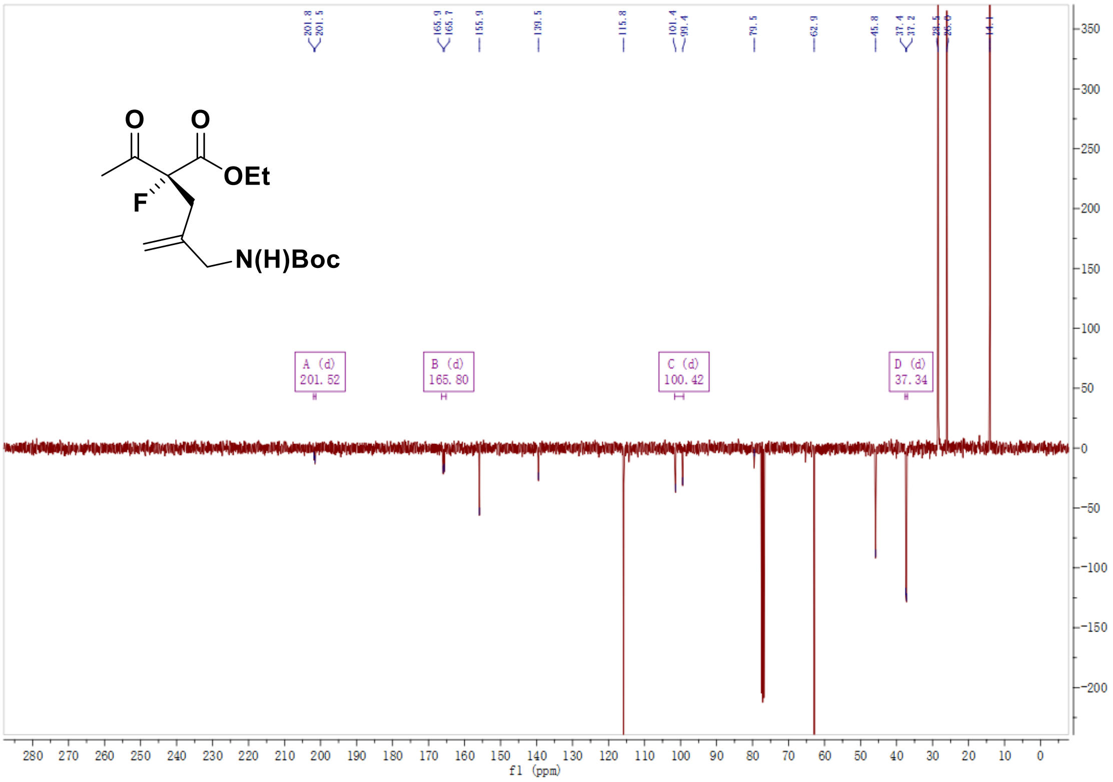

tert-butyl 2-benzoyl-4-(((tert-butoxycarbonyl)amino)methyl)-2-fluoropent-4-enoate (6a)

^{1}H NMR, $CDCl_3$, 400 MHz

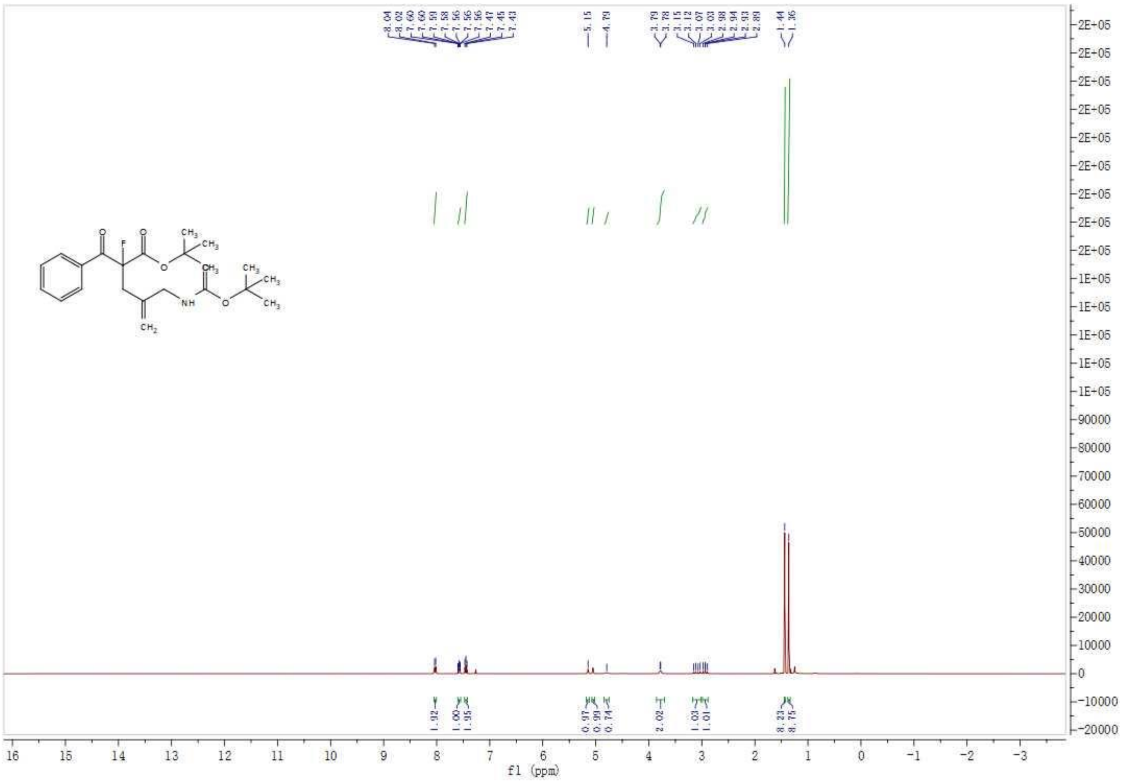

^{19}F NMR, $CDCl_3$, 377 MHz

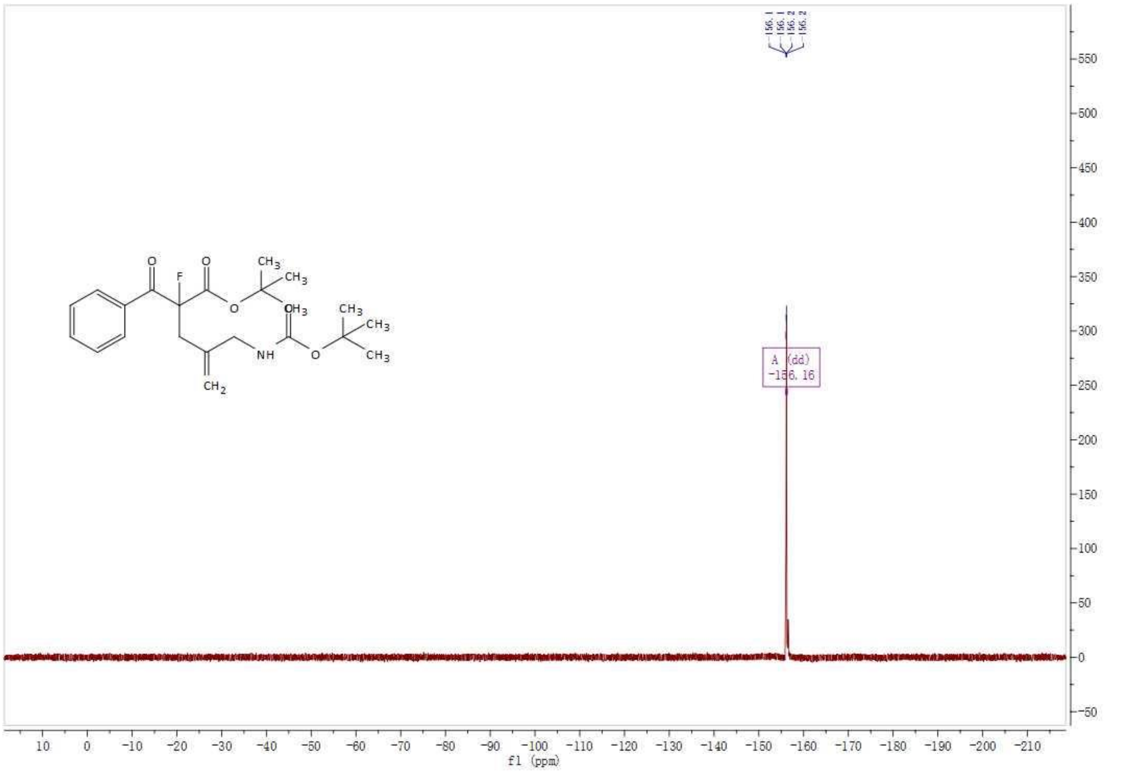

^{13}C NMR, $CDCl_3$, 101 MHz

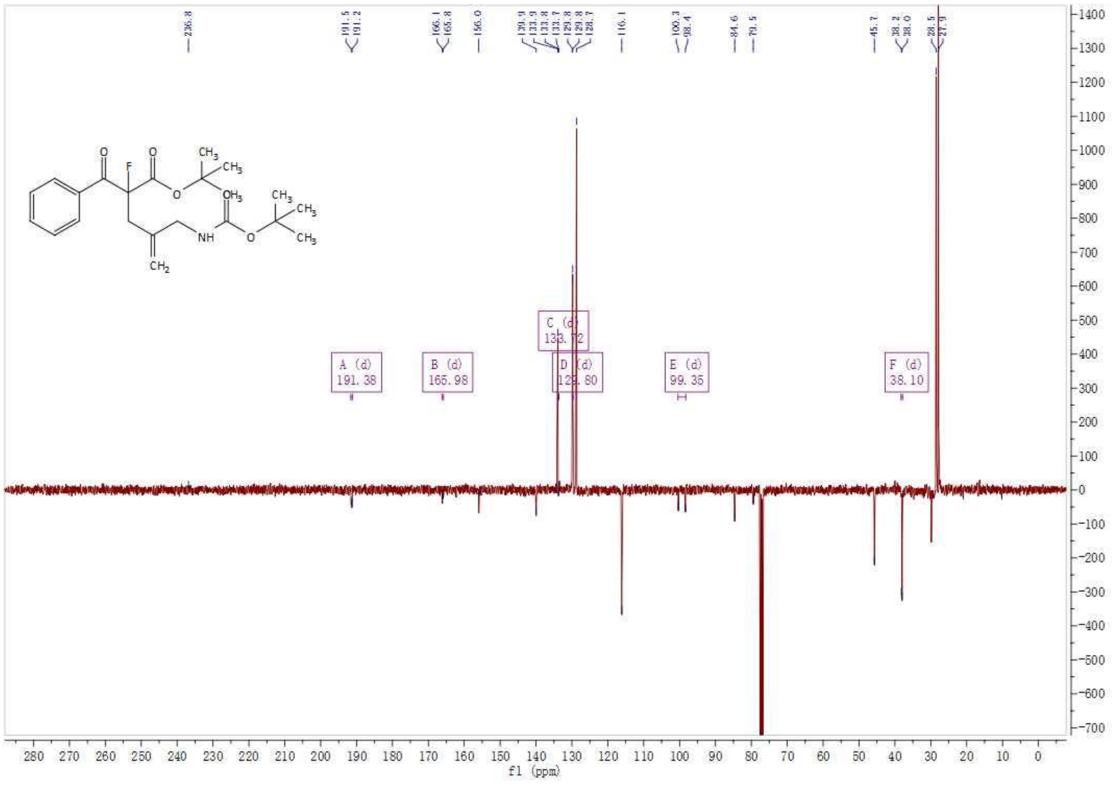

tert-butyl 4-(((_tert_-butoxycarbonyl)amino)methyl)-2-(4-chlorobenzoyl)-2-fluoropent-4-enoate (6b)

^{1}H NMR, $CDCl_3$, 400 MHz

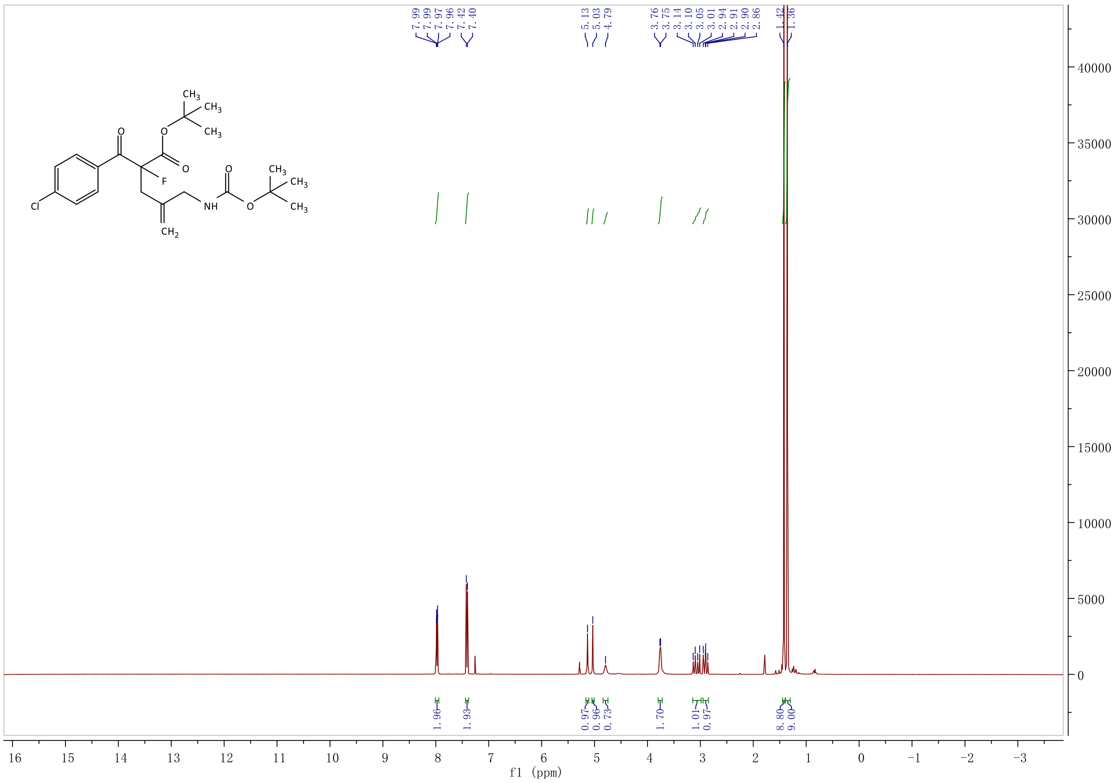

^{19}F NMR, $CDCl_3$, 377 MHz

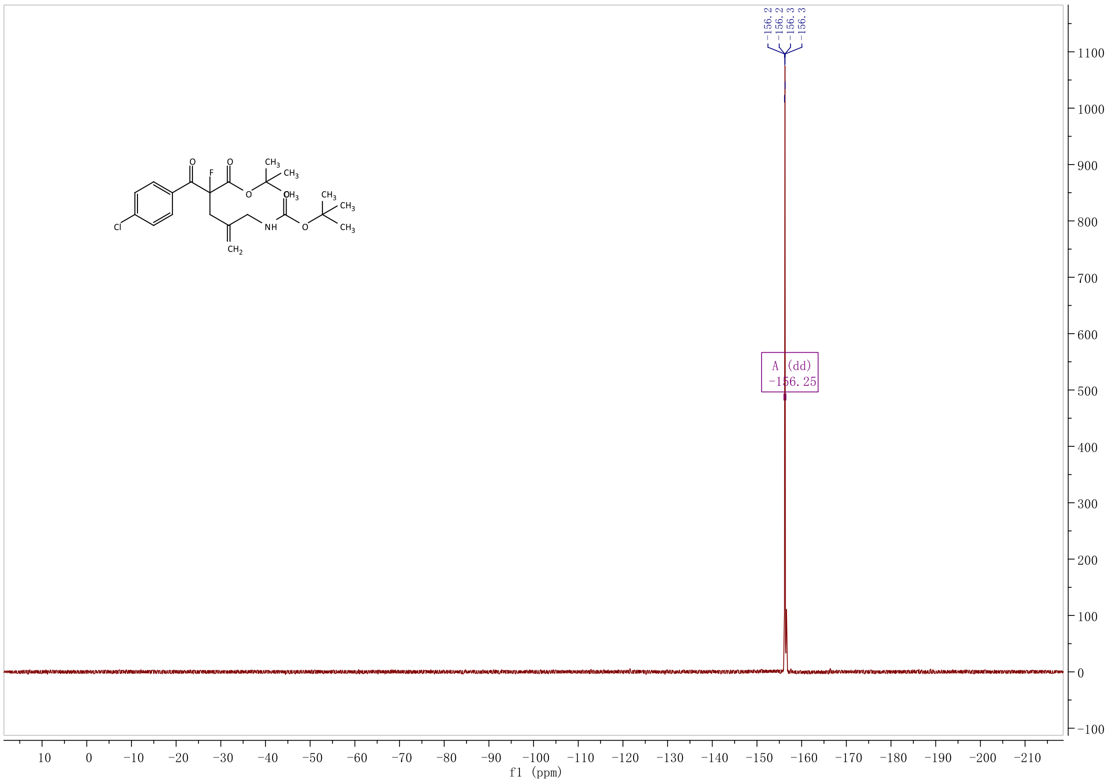

^{13}C NMR, $CDCl_3$, 101 MHz

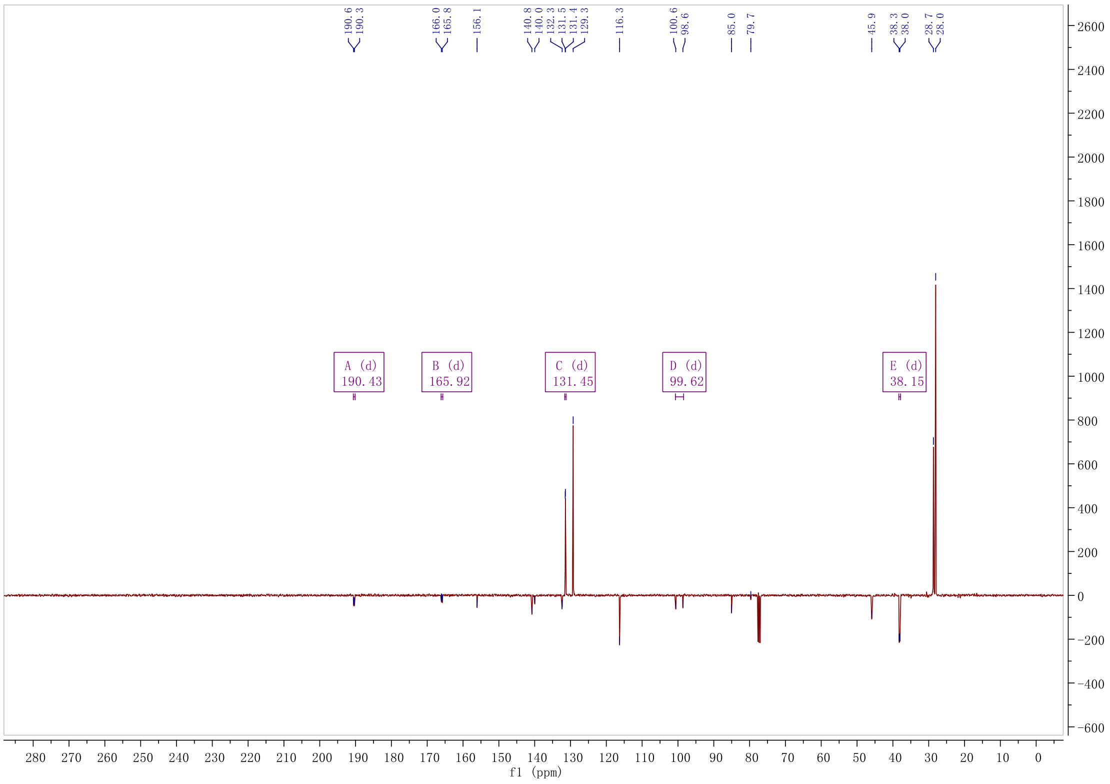

tert-butyl 4-(((tert-butoxycarbonyl)amino)methyl)-2-fluoro-2-(4-nitrobenzoyl)pent-4-enoate (6c)

^{1}H NMR, $CDCl_3$, 400 MHz

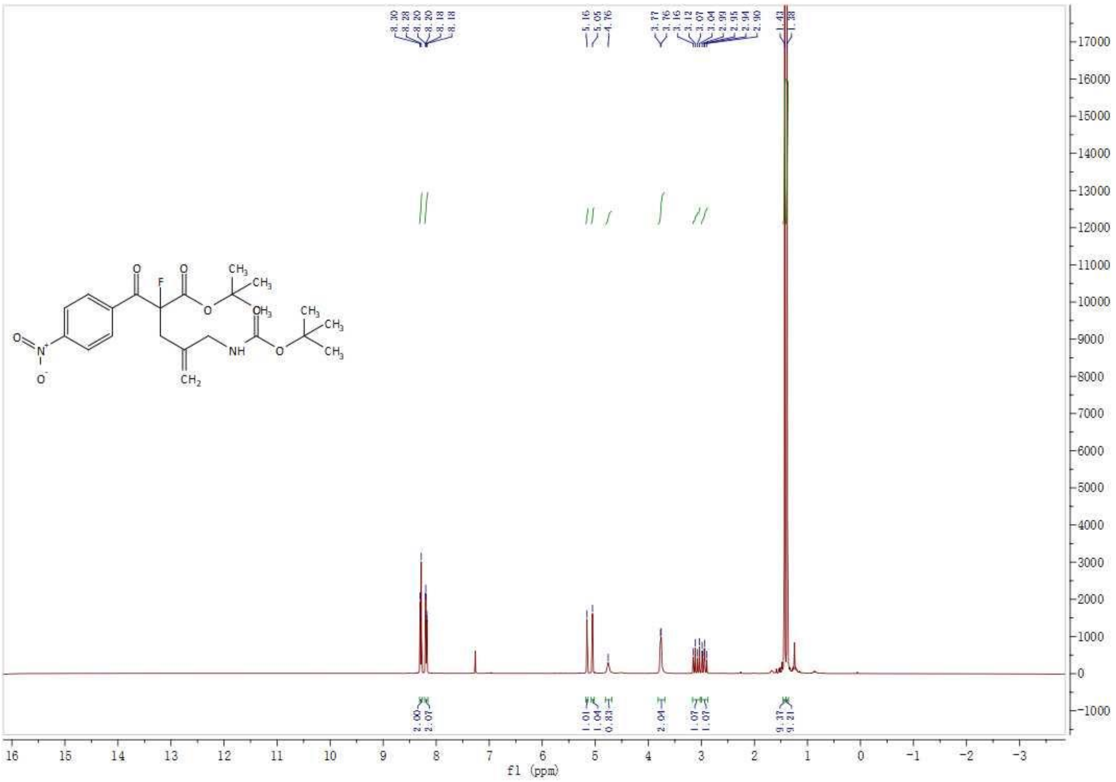

^{19}F NMR, $CDCl_3$, 377 MHz

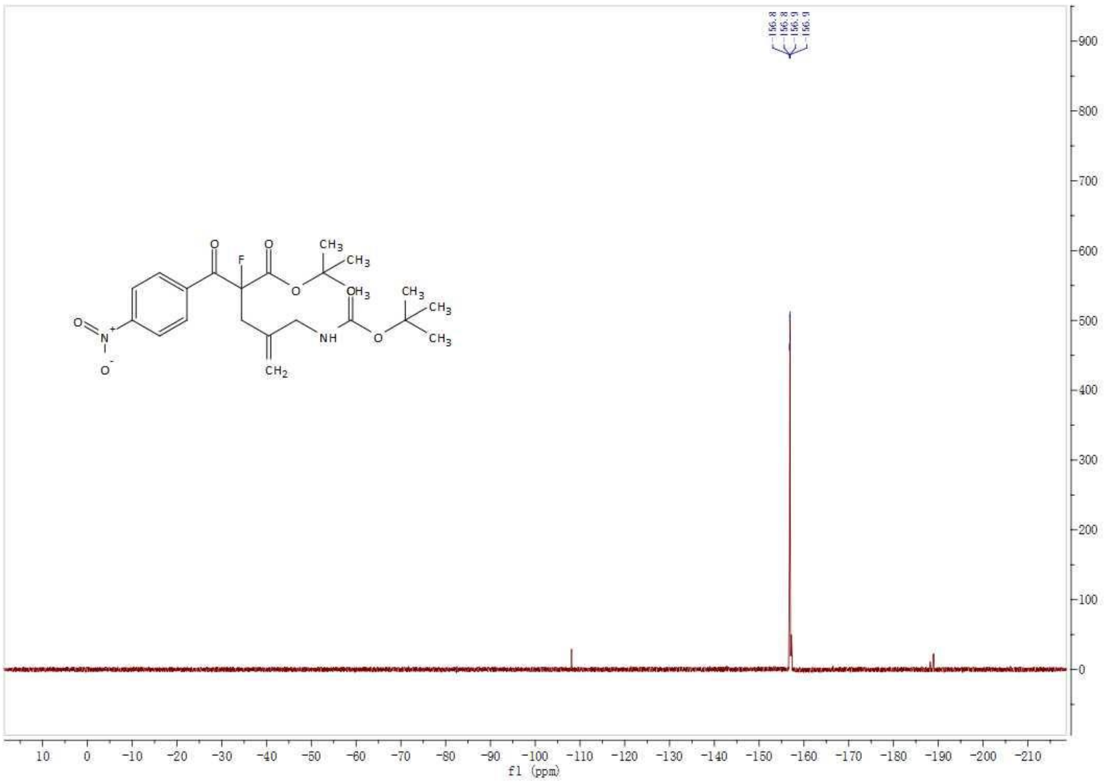

^{13}C NMR, $CDCl_3$, 101 MHz

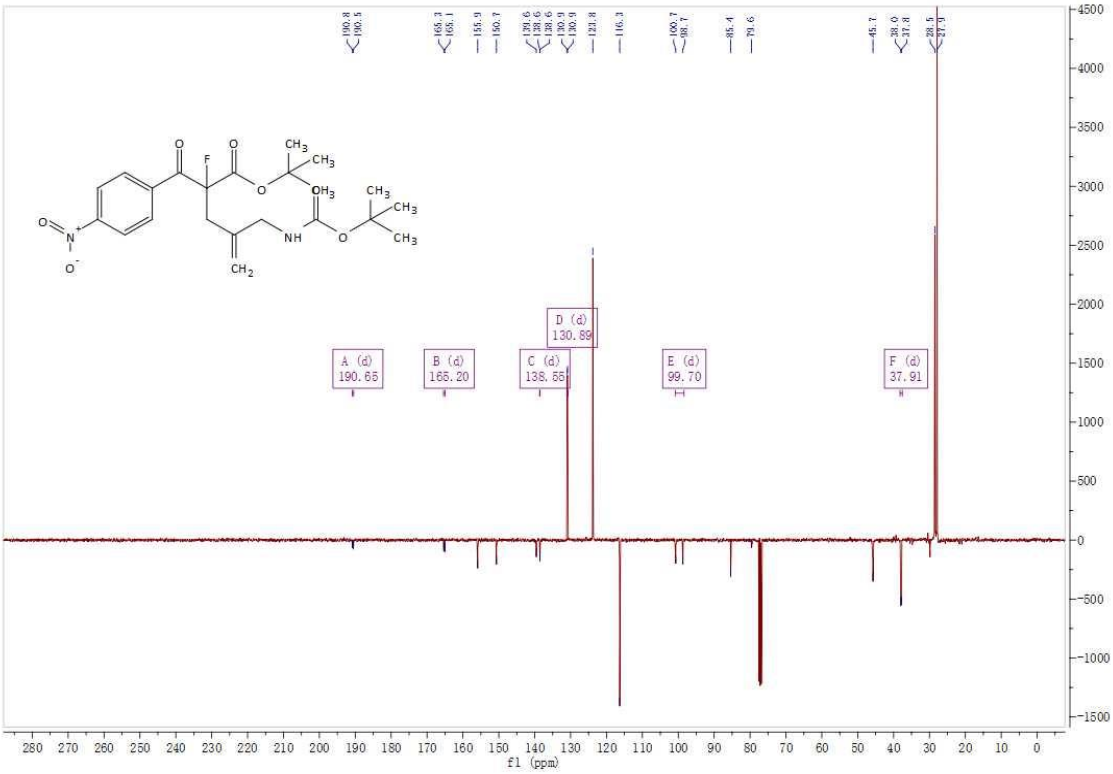

***tert*-butyl 4-(((tert-butoxycarbonyl)amino)methyl)-2-fluoro-2-(4-(trifluoromethyl)benzoyl)pent-4-enoate (6d)**

^{1}H NMR, $CDCl_3$, 400 MHz

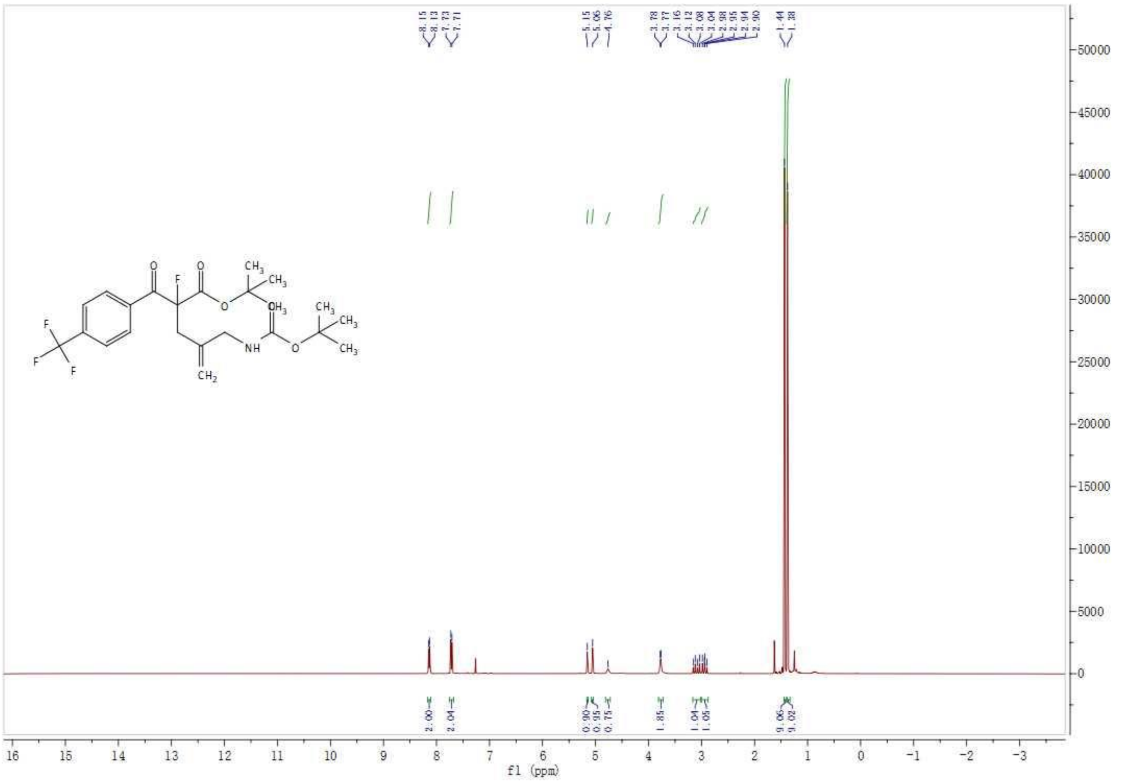

^{19}F NMR, $CDCl_3$, 377 MHz

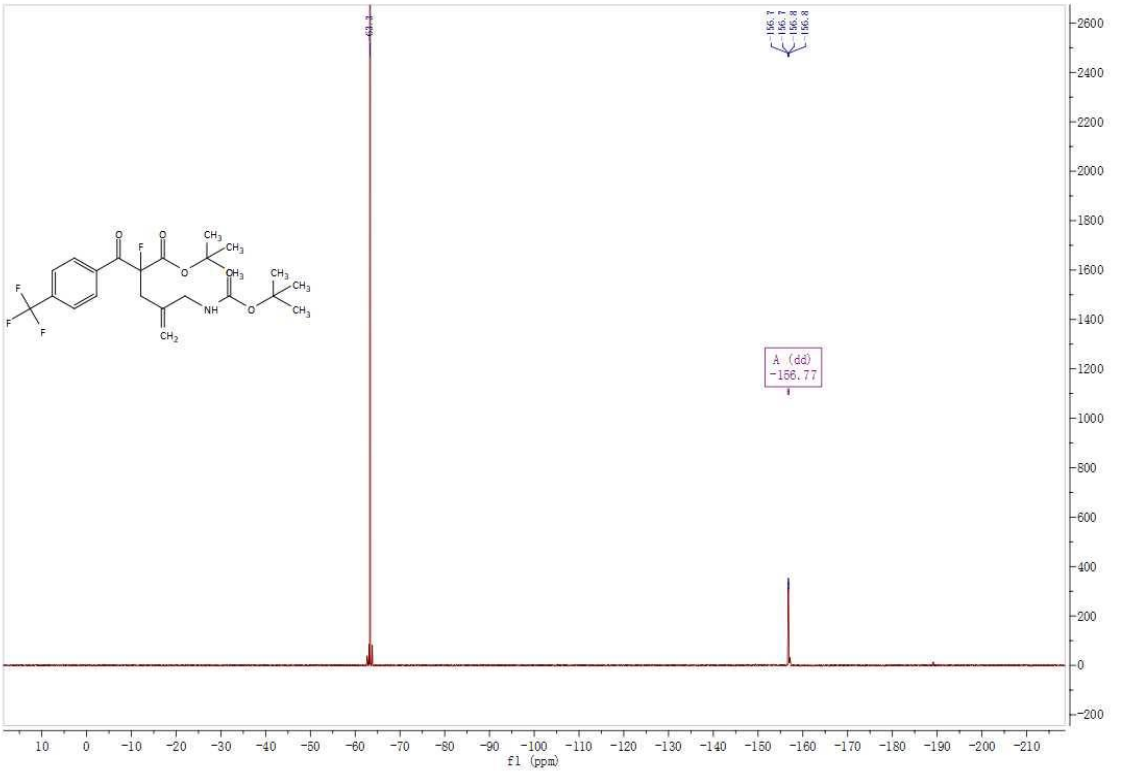

^{13}C NMR, $CDCl_3$, 101 MHz

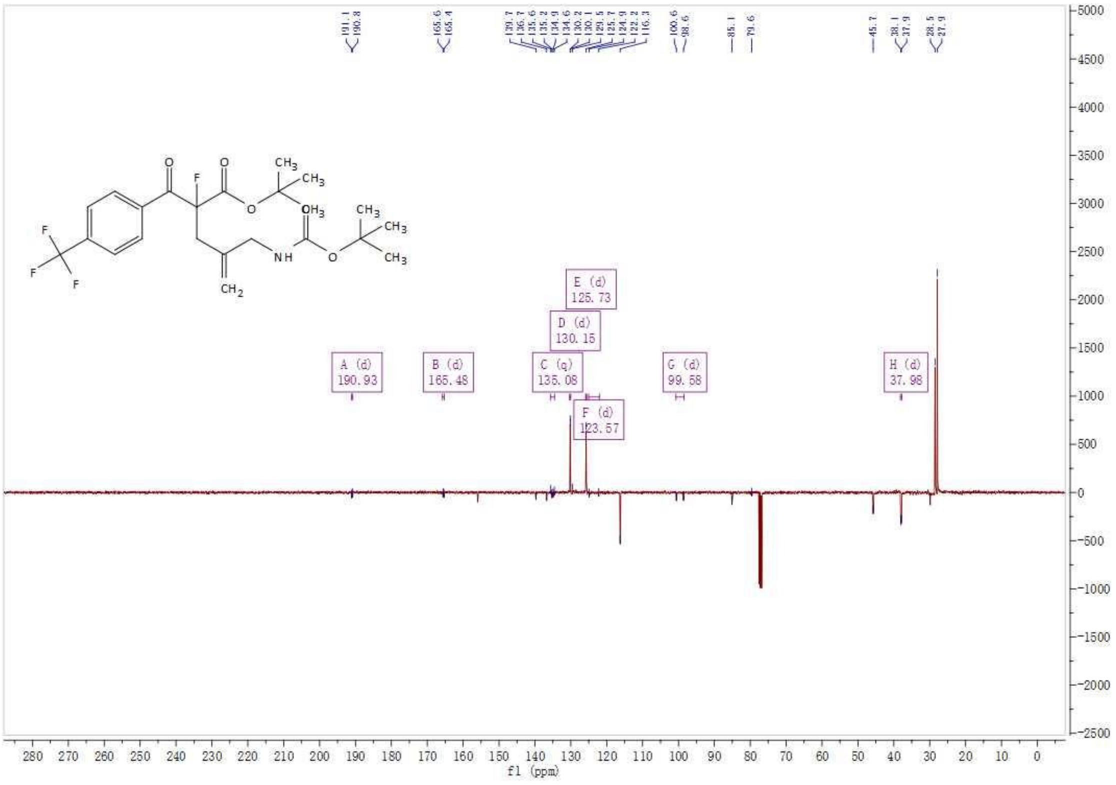

***tert*-butyl 4-(((tert-butoxycarbonyl)amino)methyl)-2-fluoro-2-(4-methoxybenzoyl)pent-4-enoate (6d)**

^{1}H NMR, $CDCl_3$, 400 MHz

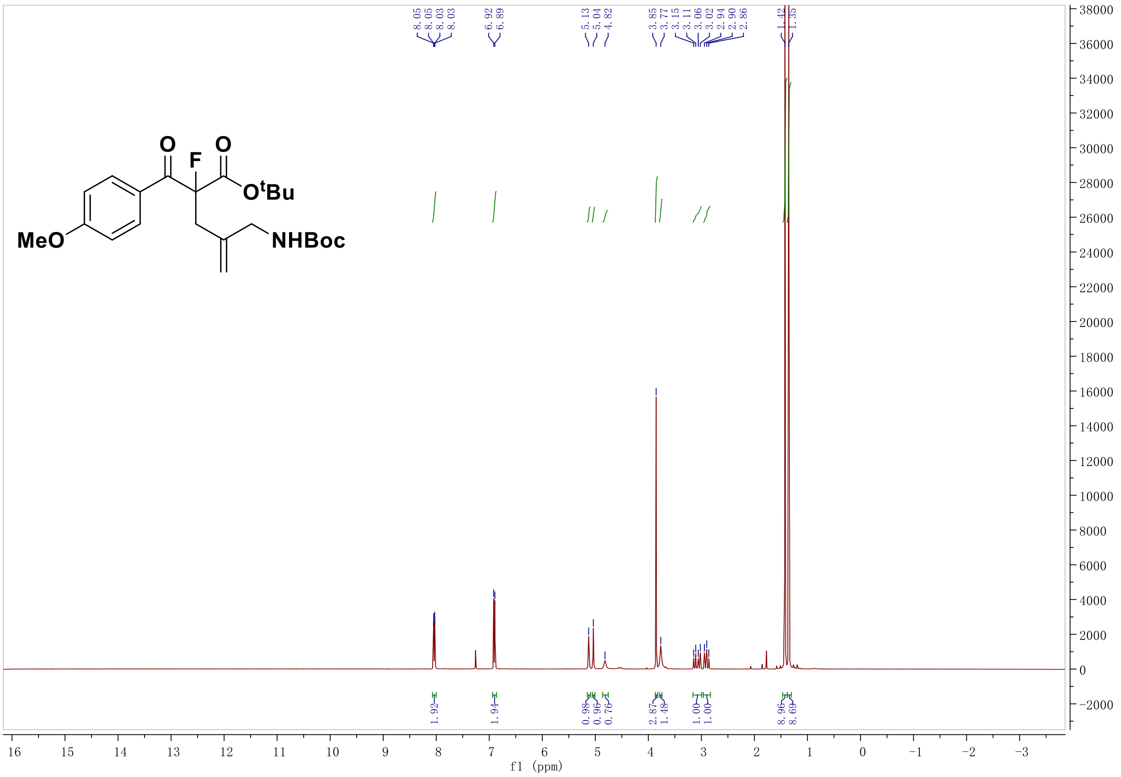

^{19}F NMR, $CDCl_3$, 377 MHz

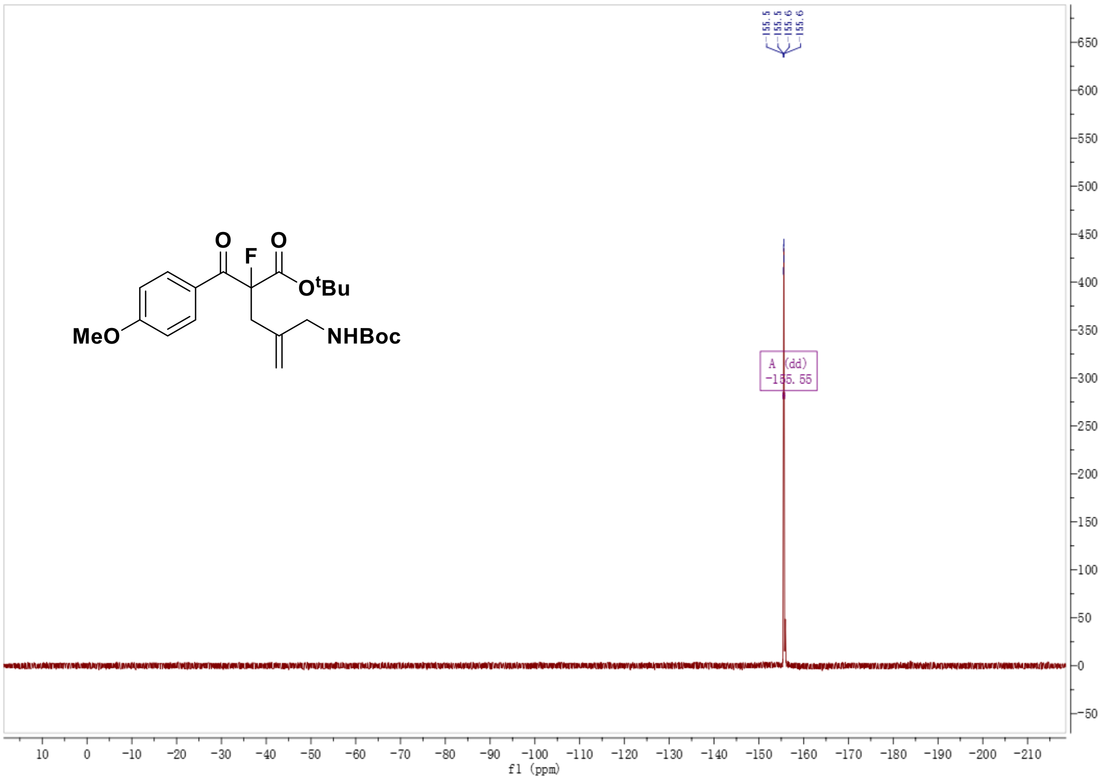

^{13}C NMR, $CDCl_3$, 101 MHz

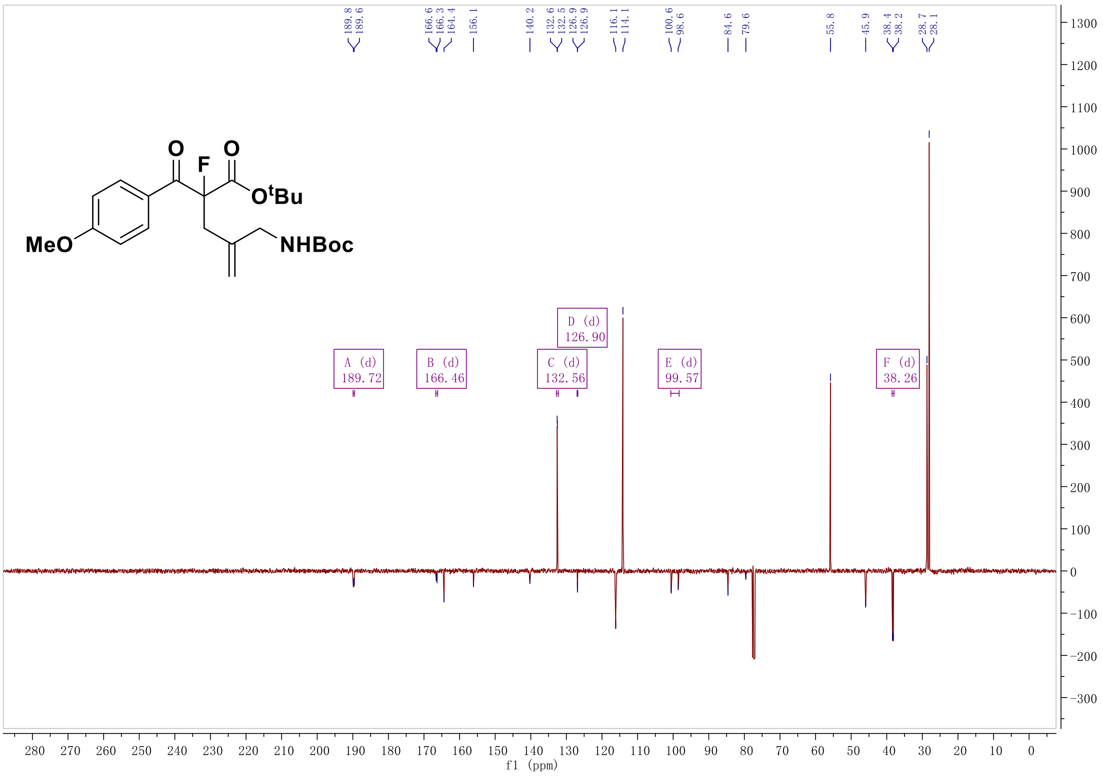

ethyl 2-acetyl-4-{[(*tert*-butoxycarbonyl)amino]methyl}-2-fluoropent-4-enoate (6f)

^{1}H NMR, CDCl$_3$, 400 MHz

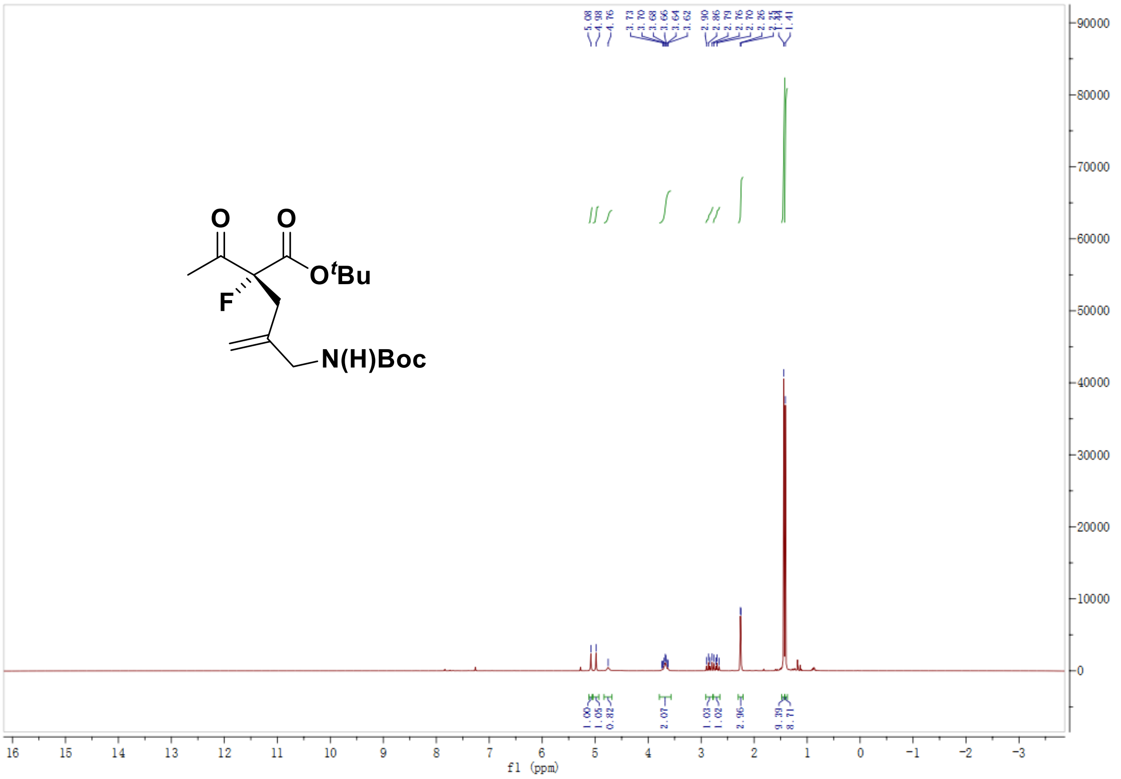

^{19}F NMR, CDCl$_3$, 377 MHz

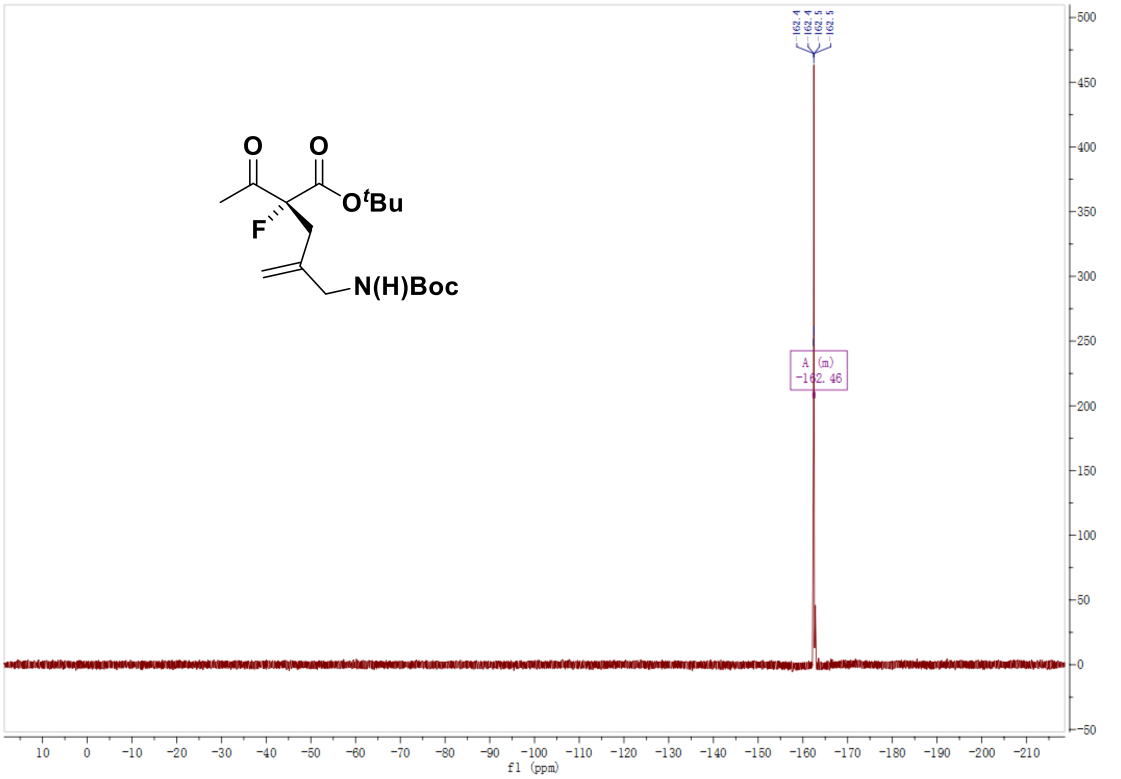

^{13}C NMR, CDCl$_3$, 101 MHz

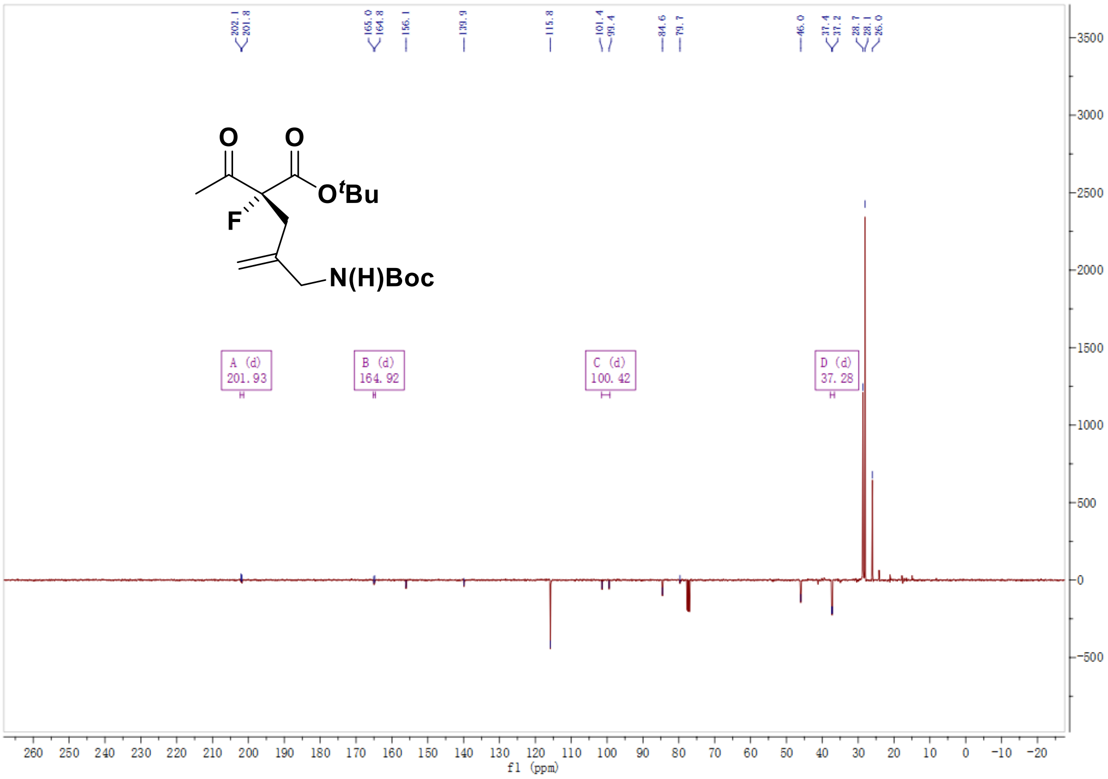

***tert*-butyl (2*S*,3*R*)- 3-fluoro-5-methylene-2-phenylpiperidine-3-carboxylate (>98:2 dr)**

^{1}H NMR, $CDCl_3$, 400 MHz

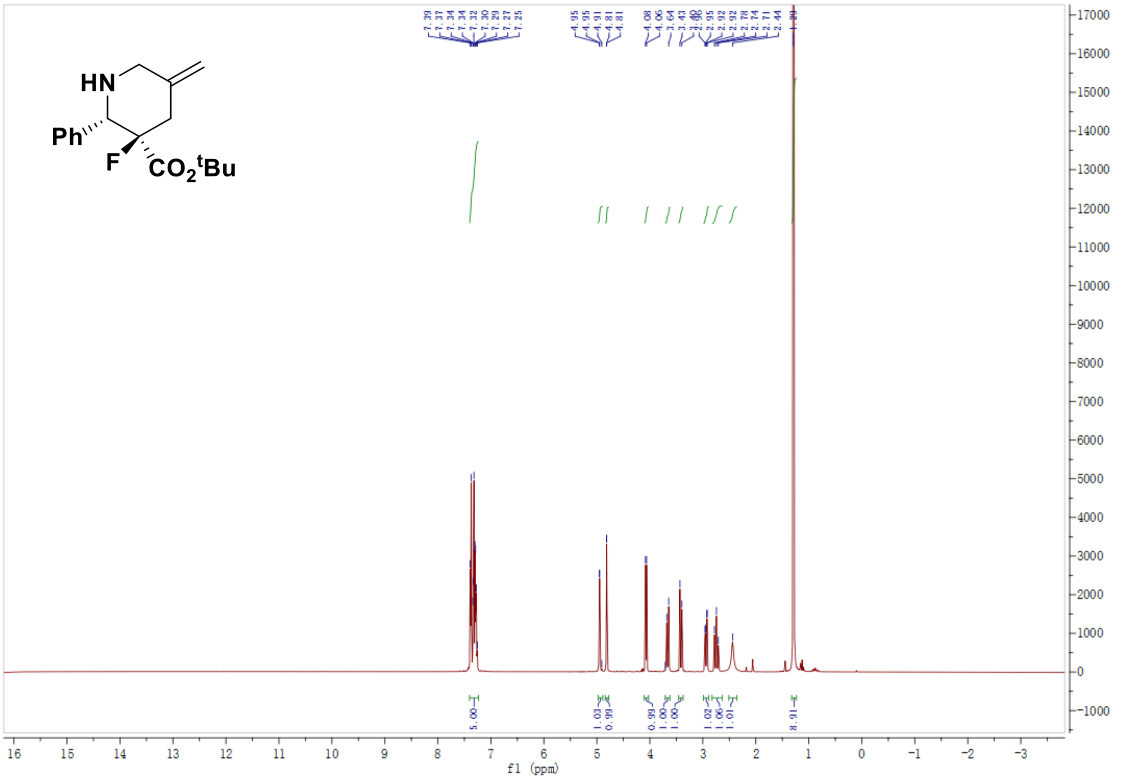

^{19}F NMR, $CDCl_3$, 377 MHz

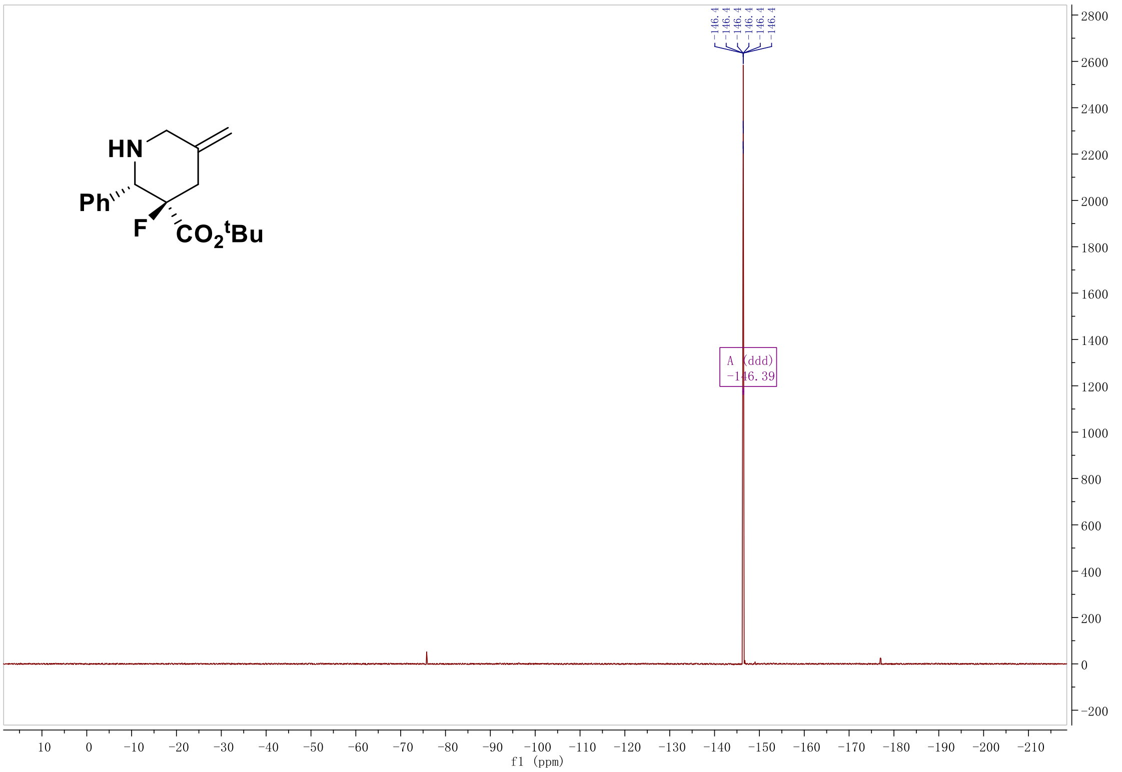

^{13}C NMR, $CDCl_3$, 101 MHz

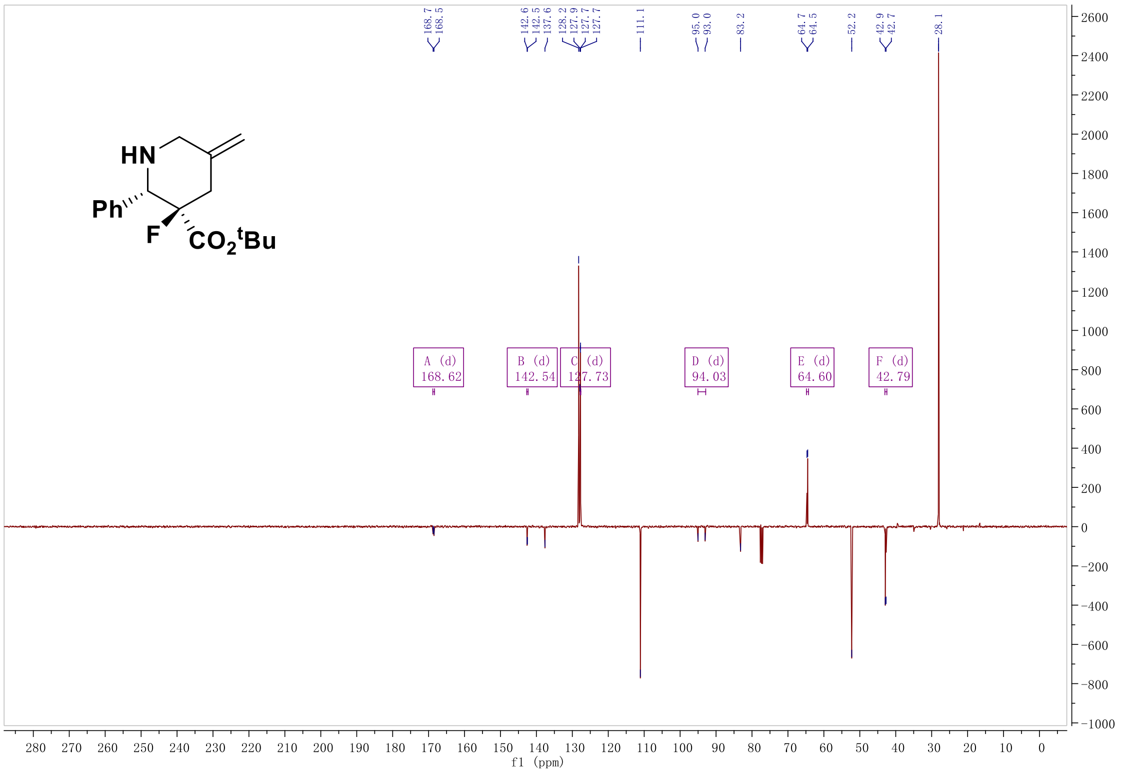

di-*tert*-butyl (2*S*,3*R*)- 3-fluoro-5-methylene-2-phenylpiperidine-1,3-dicarboxylate (7)

^{1}H NMR, $CDCl_3$, 400 MHz

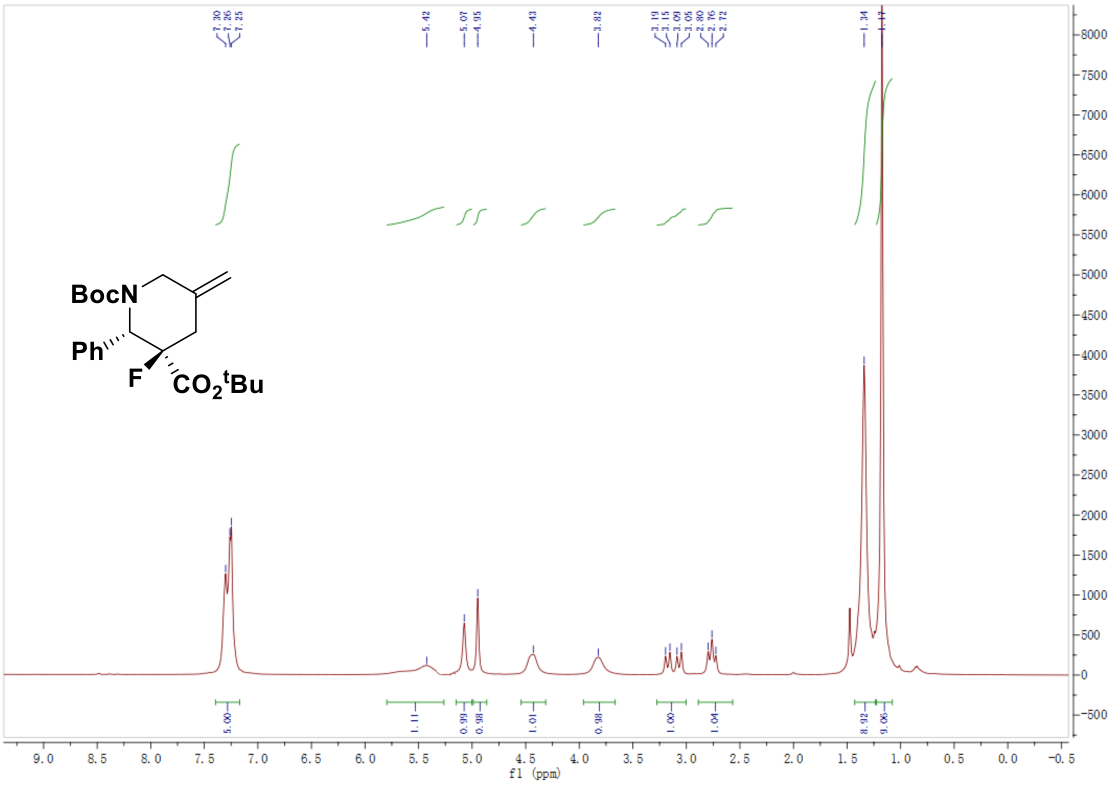

^{19}F NMR, $CDCl_3$, 377 MHz

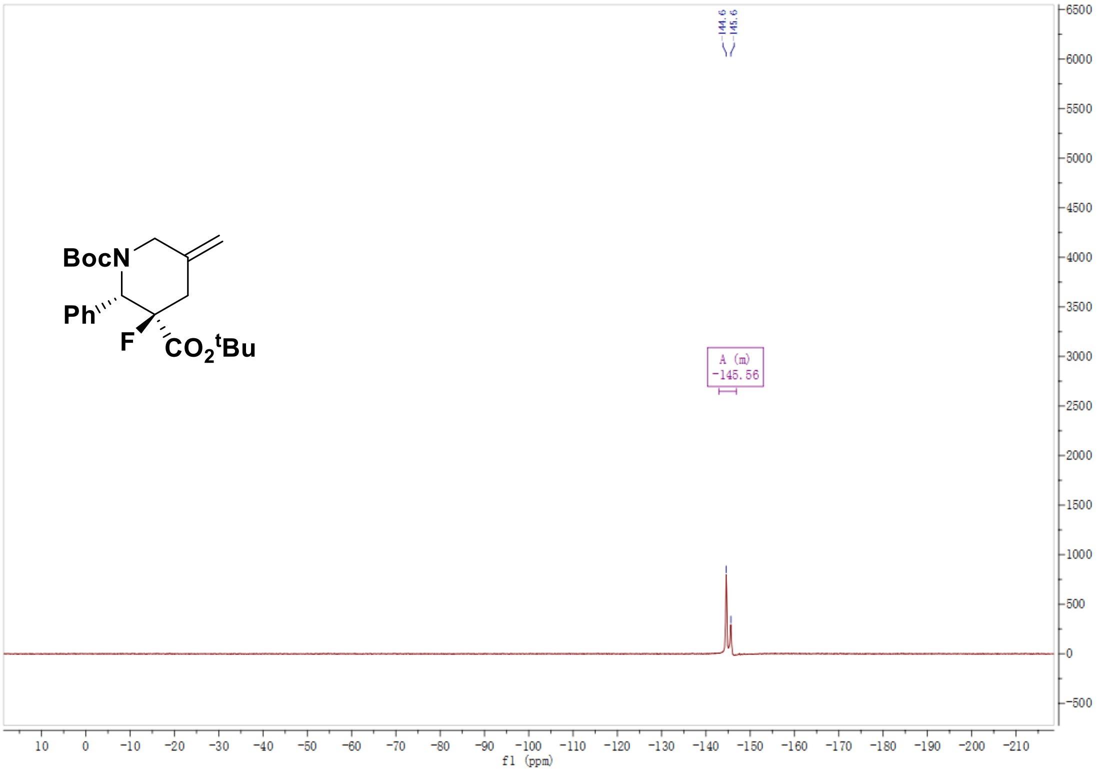

^{13}C NMR, $CDCl_3$, 101 MHz

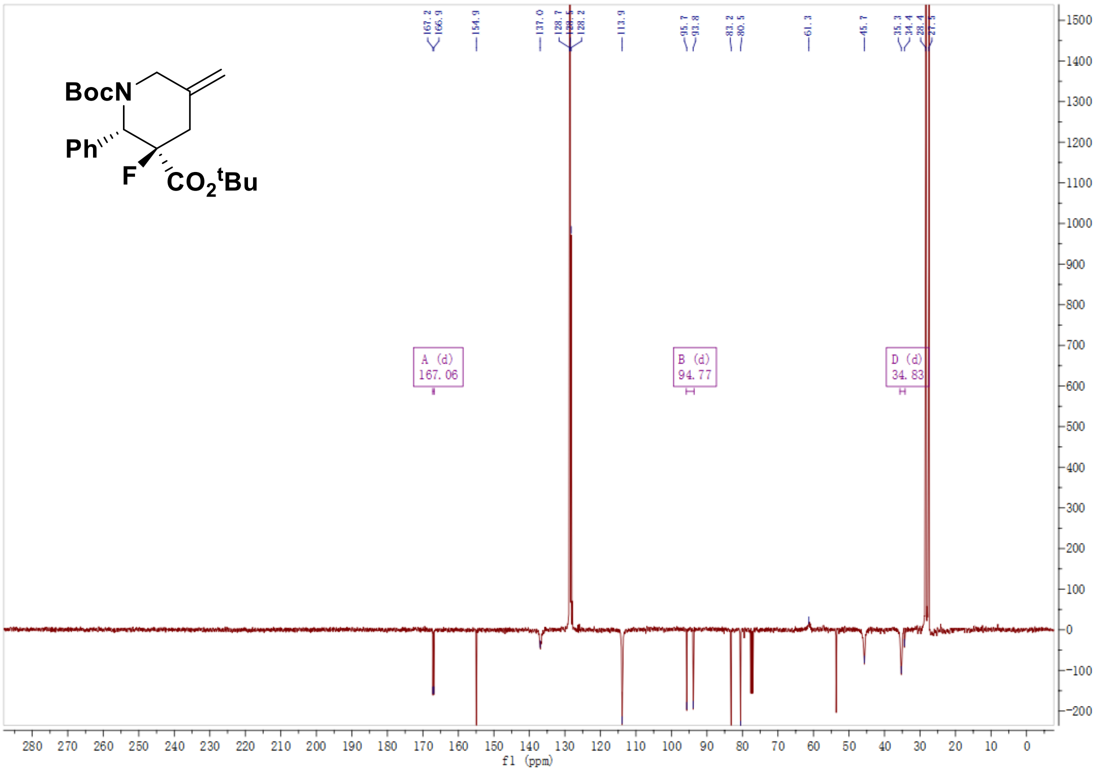

di-*tert*-butyl (6S,7R)-1,1,7-trifluoro-6-phenyl-5-azaspiro[2.5]octane-5,7-dicarboxylate (8)

Major isomer

^{1}H NMR, $CDCl_3$, 400 MHz

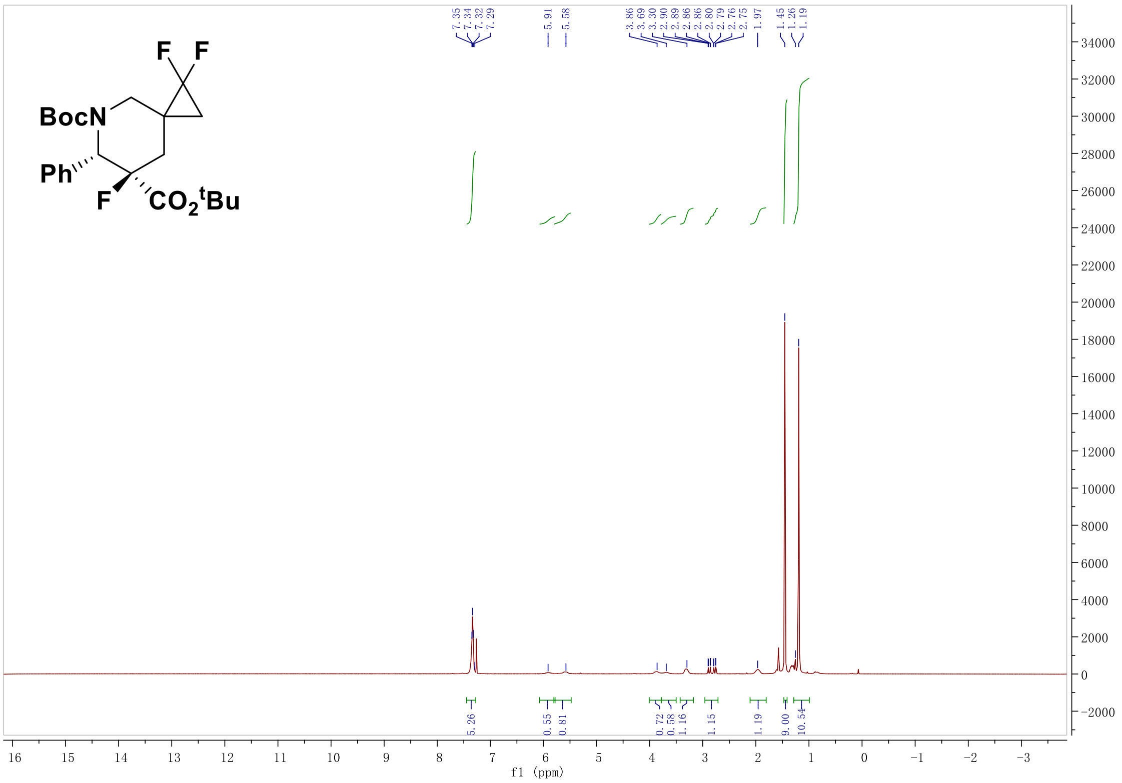

^{19}F NMR, $CDCl_3$, 377 MHz

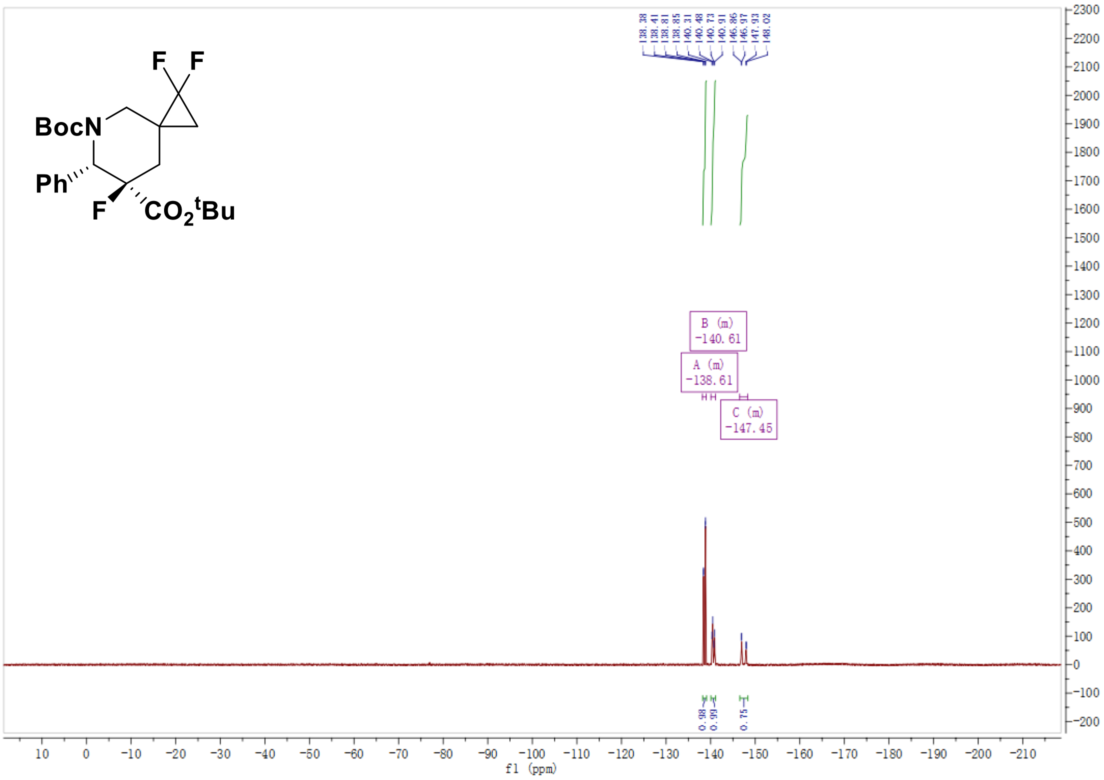

^{13}C NMR, $CDCl_3$, 101 MHz

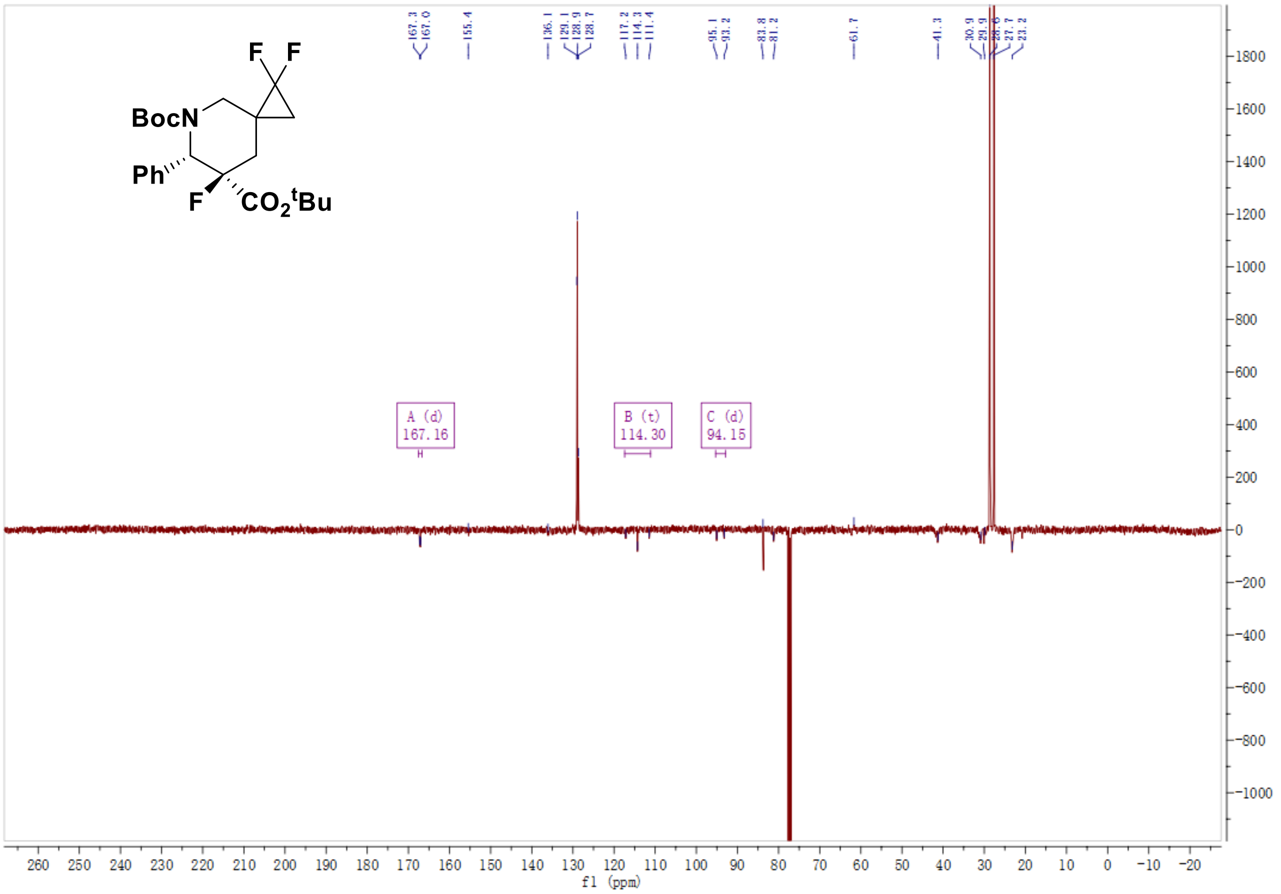

Minor Isomer

^{1}H NMR, $CDCl_3$, 400 MHz

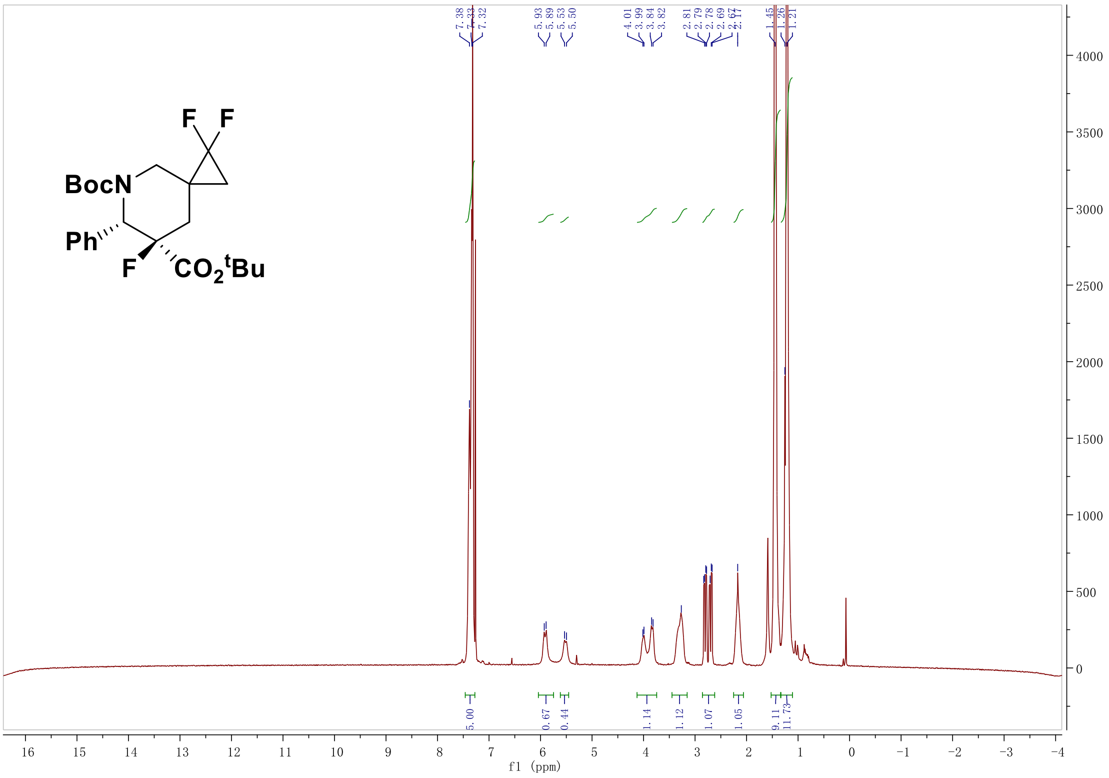

^{19}F NMR, $CDCl_3$, 377 MHz

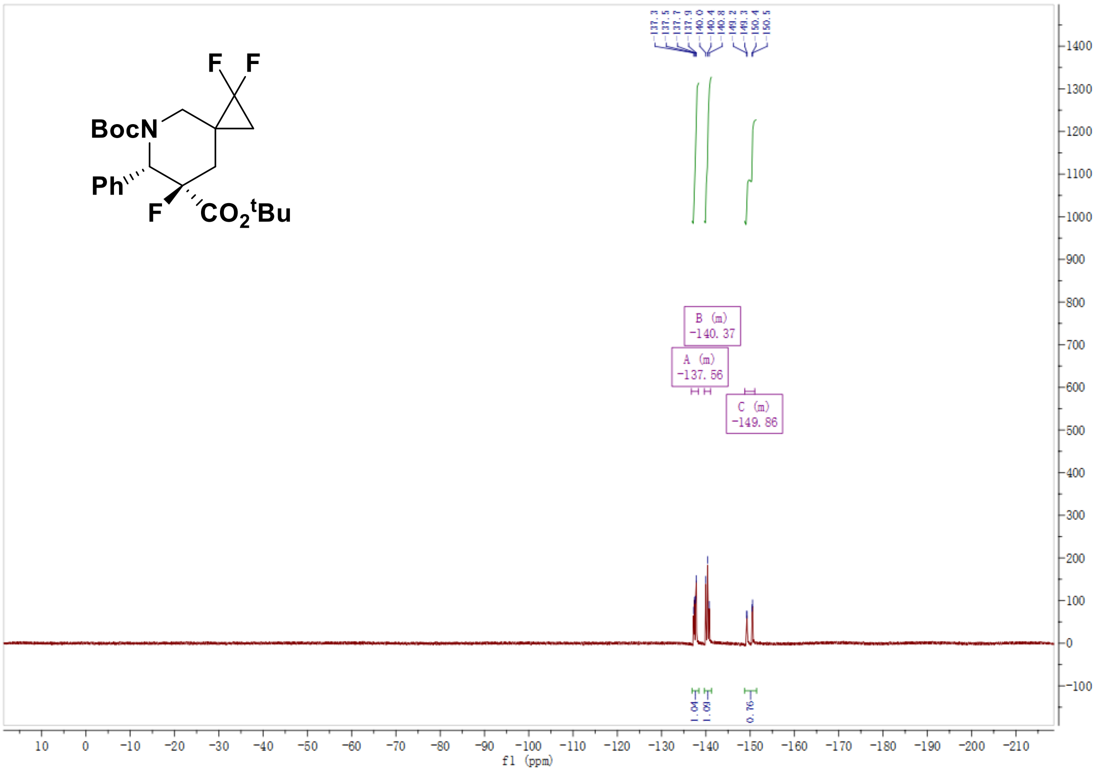

^{13}C NMR, $CDCl_3$, 101 MHz

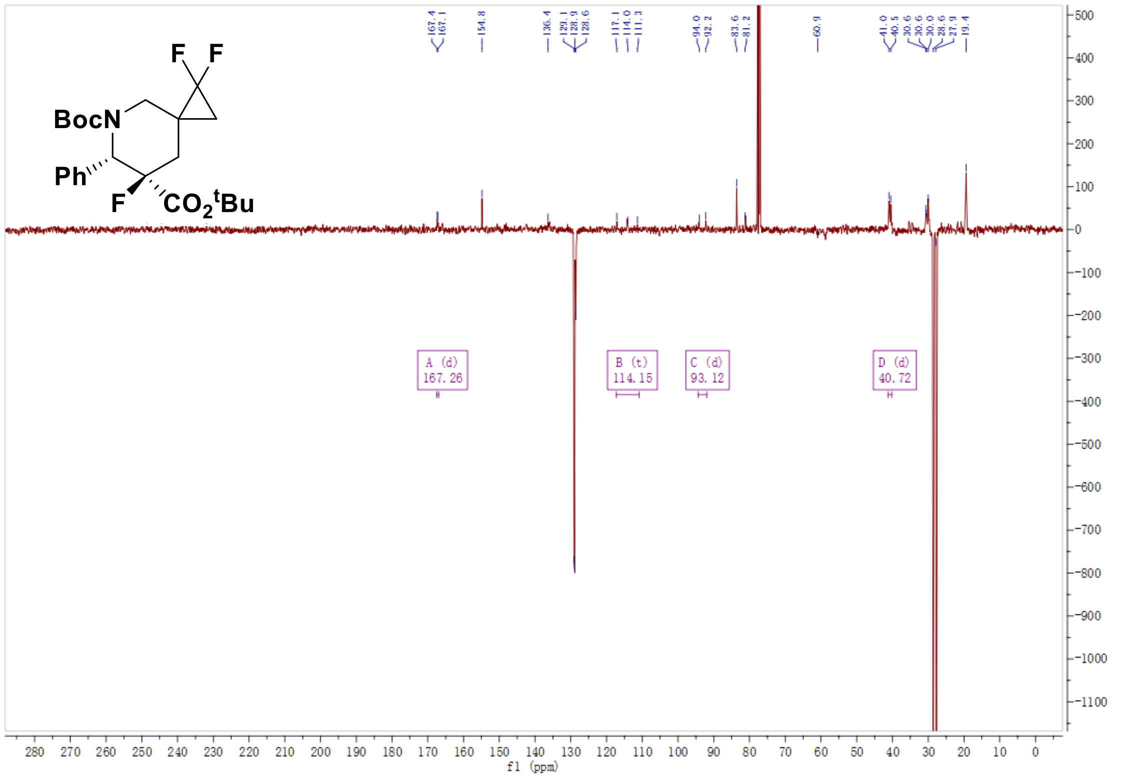

di-*tert*-butyl (6S,7R)-7-fluoro-6-phenyl-1-oxa-5-azaspiro[2.5]octane-5,7-dicarboxylate (9)

Major isomer

^{1}H NMR, $CDCl_3$, 400 MHz

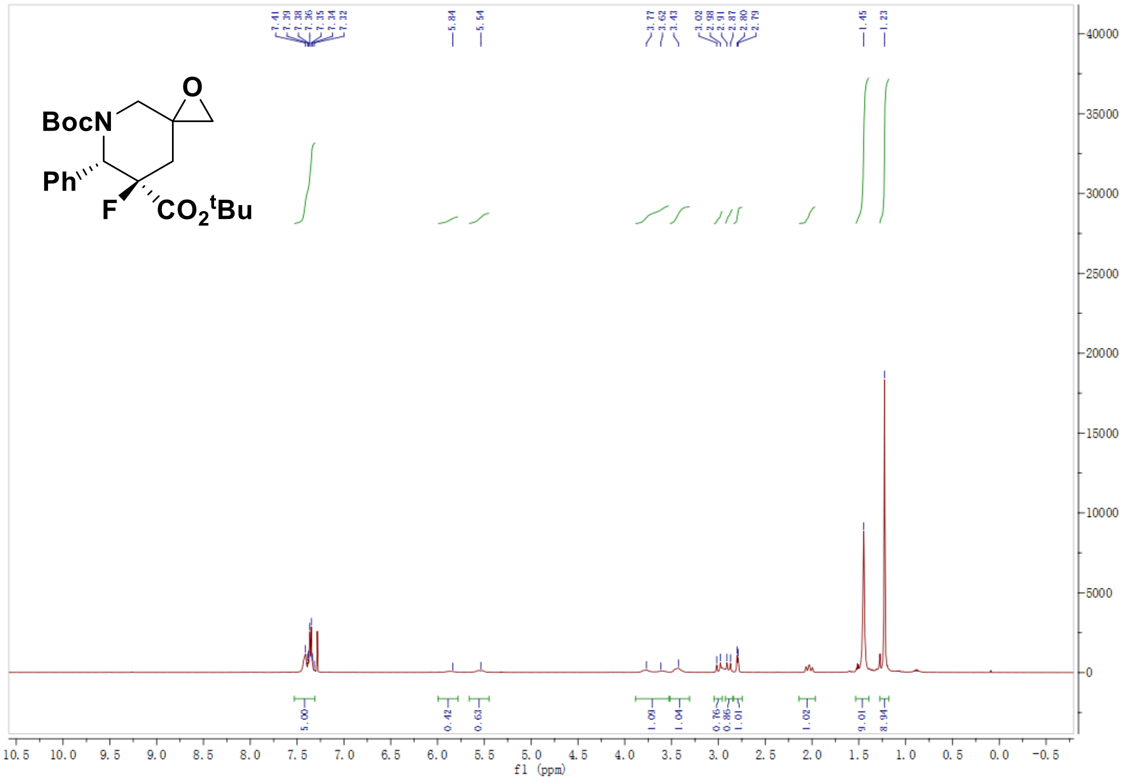

^{19}F NMR, $CDCl_3$, 377 MHz

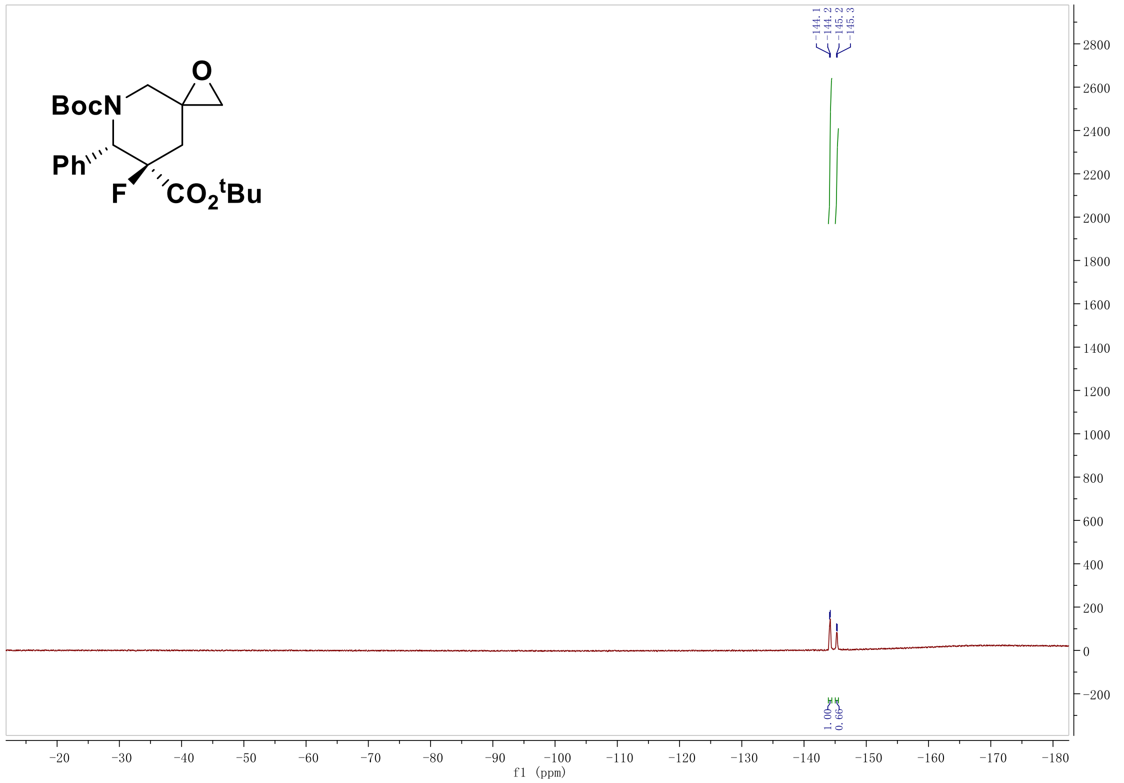

^{13}C NMR, $CDCl_3$, 101 MHz

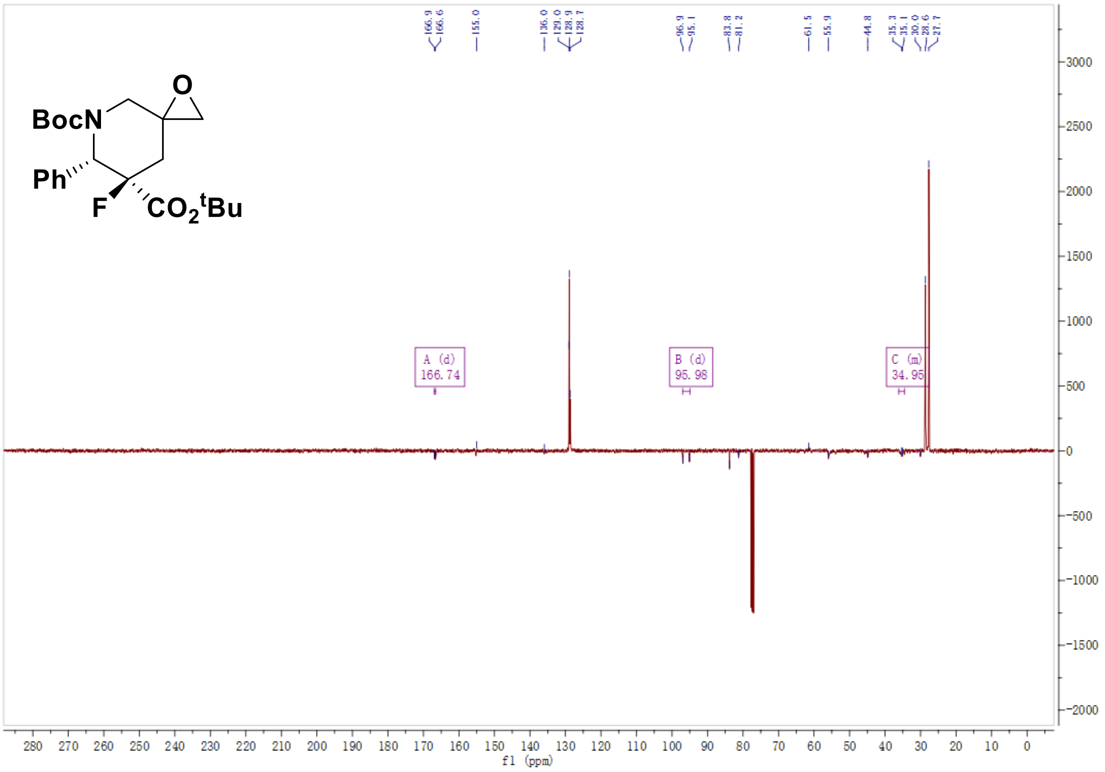

Minor isomer

^{1}H NMR, $CDCl_3$, 400 MHz

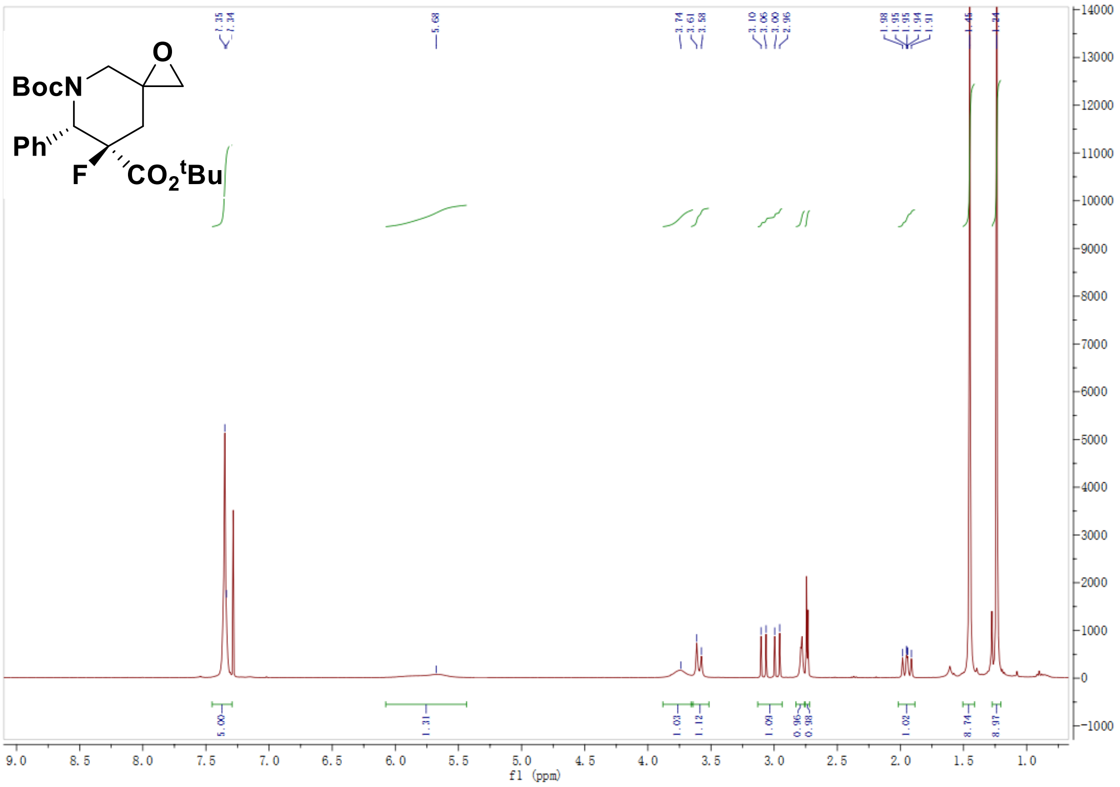

^{19}F NMR, $CDCl_3$, 377 MHz

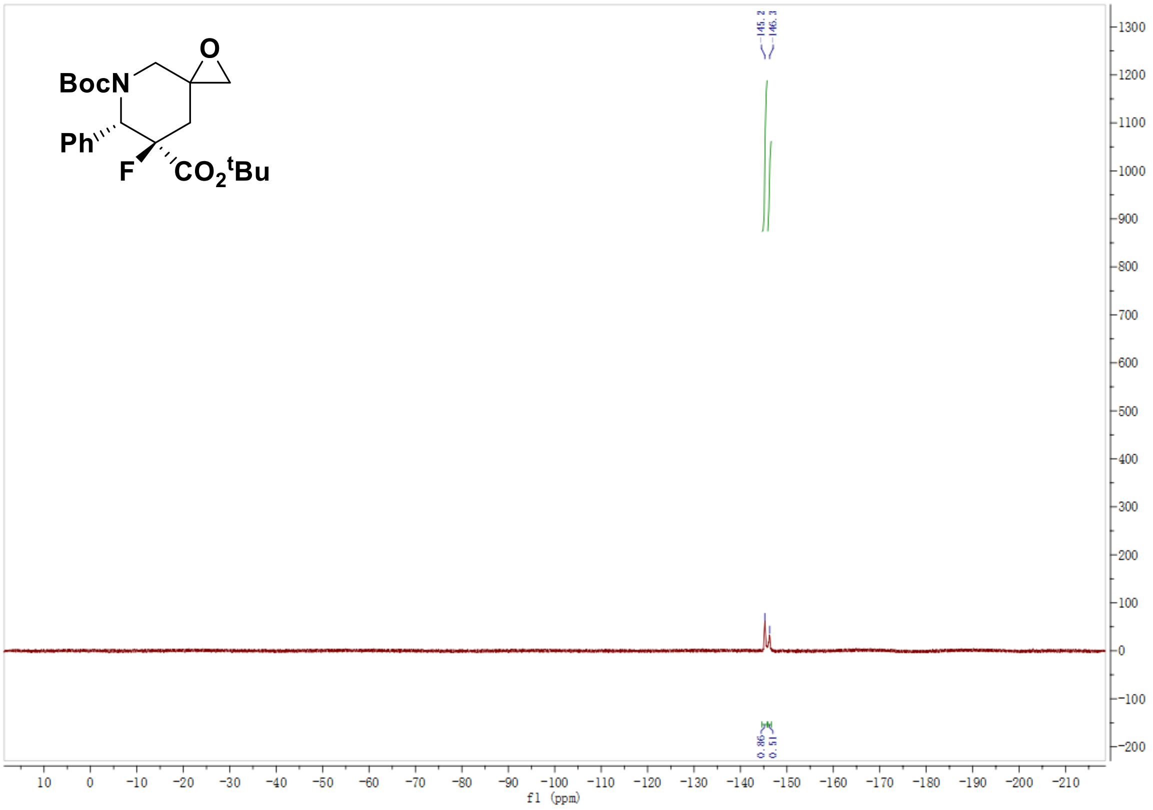

^{13}C NMR, $CDCl_3$, 101 MHz

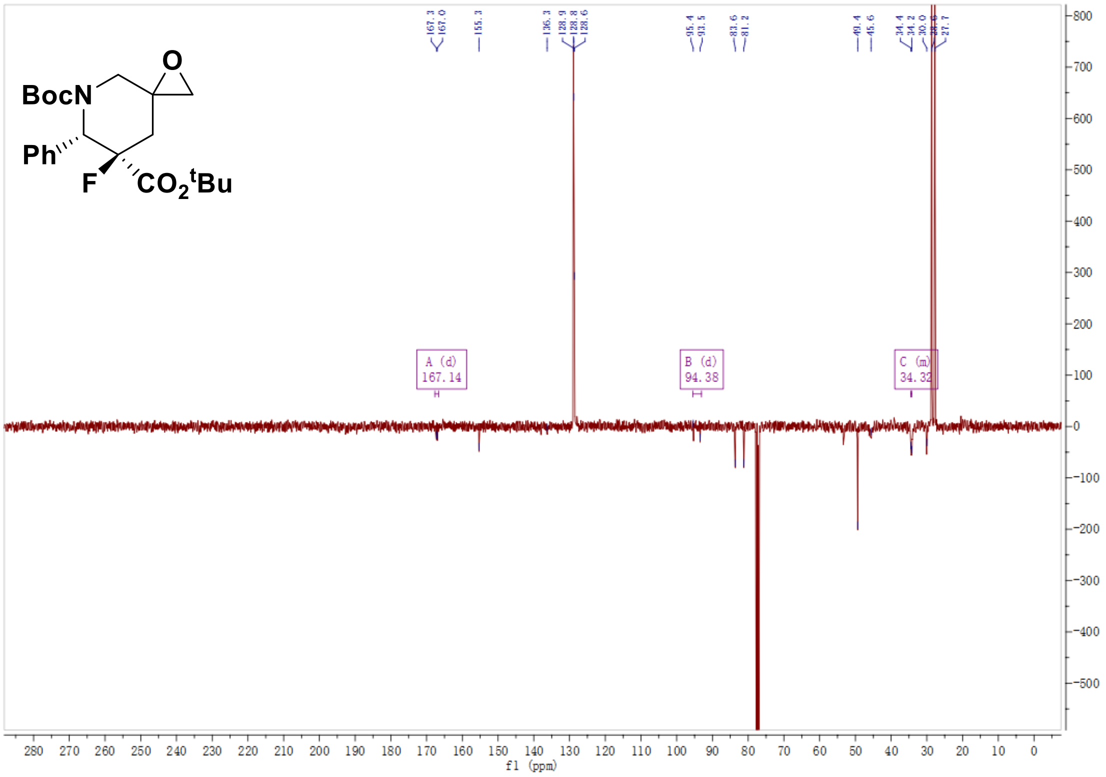

tert-butyl (2S,3R)- 3-fluoro-3-(hydroxymethyl)-5-methylene-2-phenylpiperidine-1-carboxylate (10)

^{1}H NMR, $CDCl_3$, 400 MHz

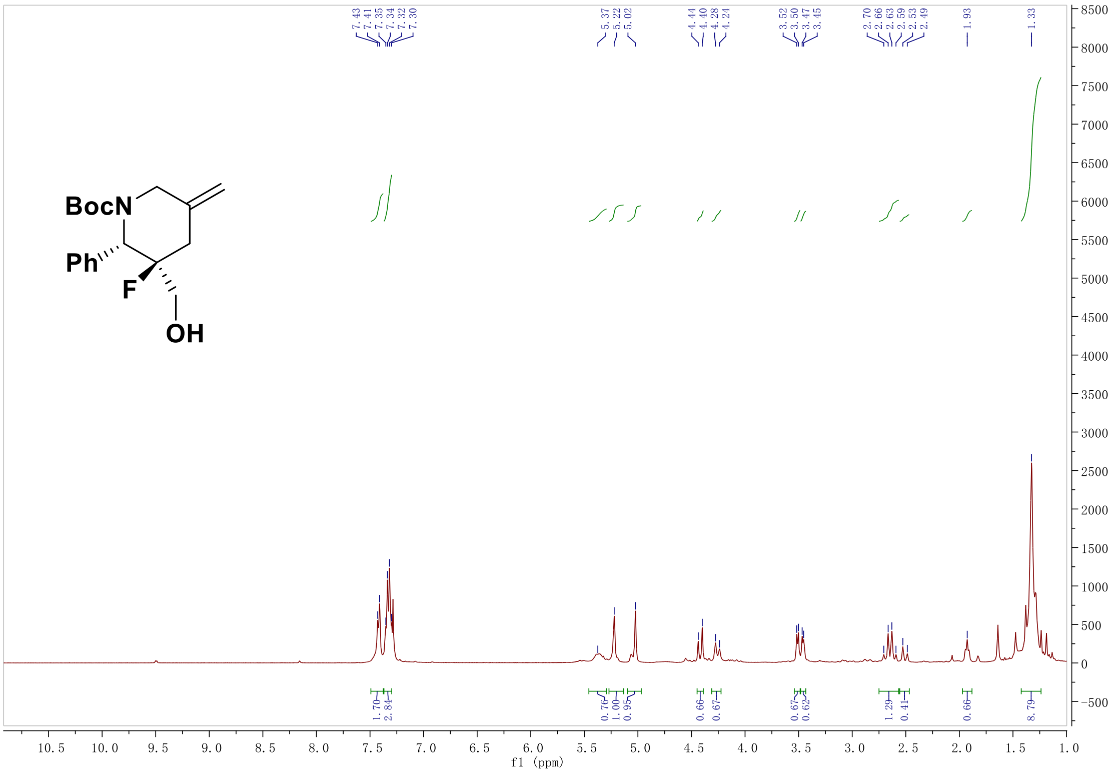

^{19}F NMR, $CDCl_3$, 377 MHz

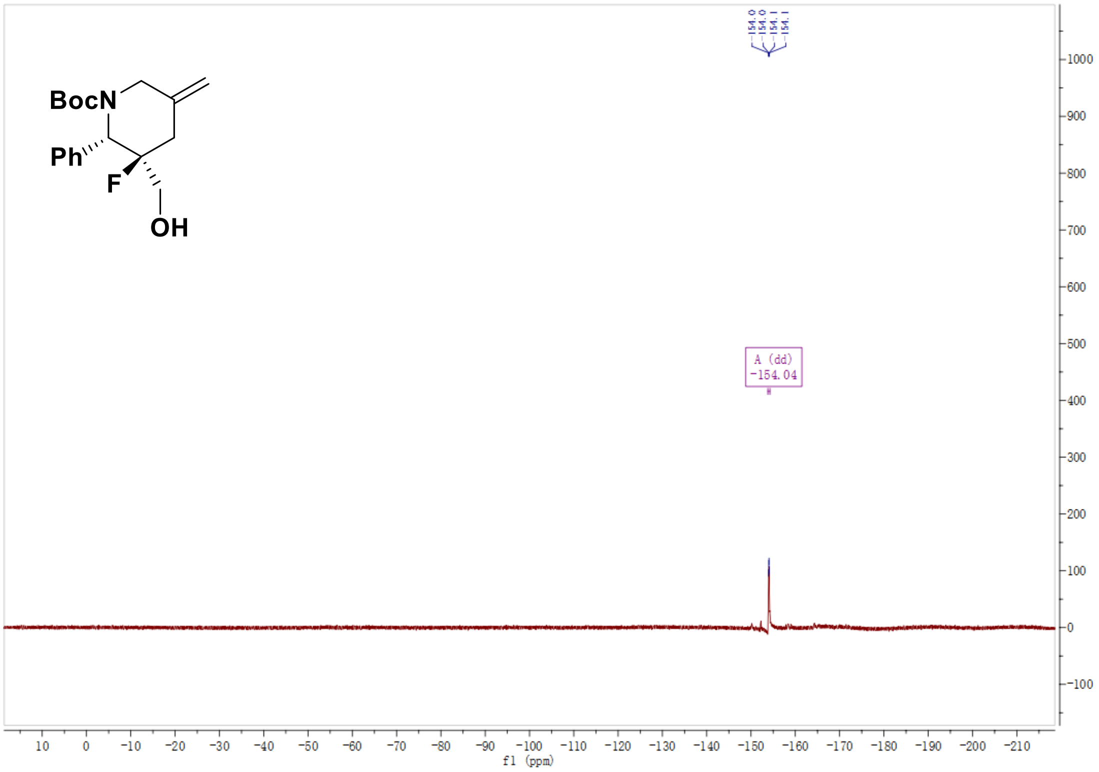

^{13}C NMR, CDCl$_3$, 101 MHz

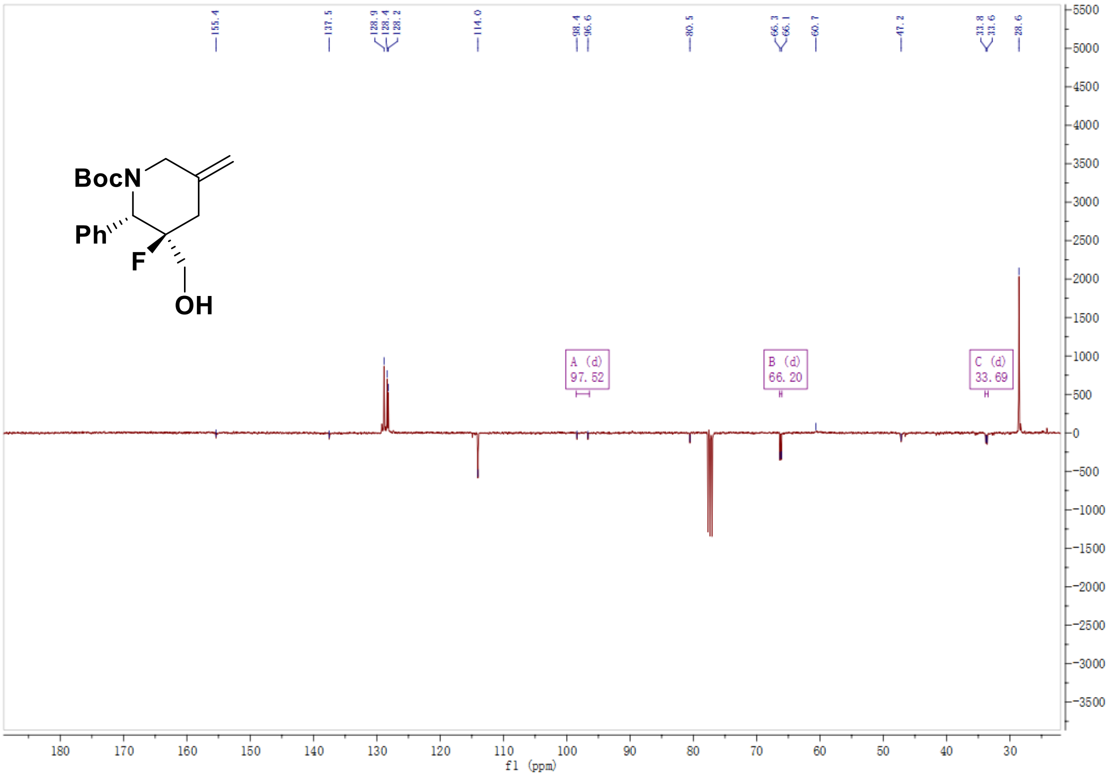

di-*tert*-butyl (*2S,3R*)-3-fluoro-5-(hydroxymethyl)-2-phenylpiperidine-1,3-dicarboxylate (11)

^{1}H NMR, $CDCl_3$, 400 MHz (borane reduction)

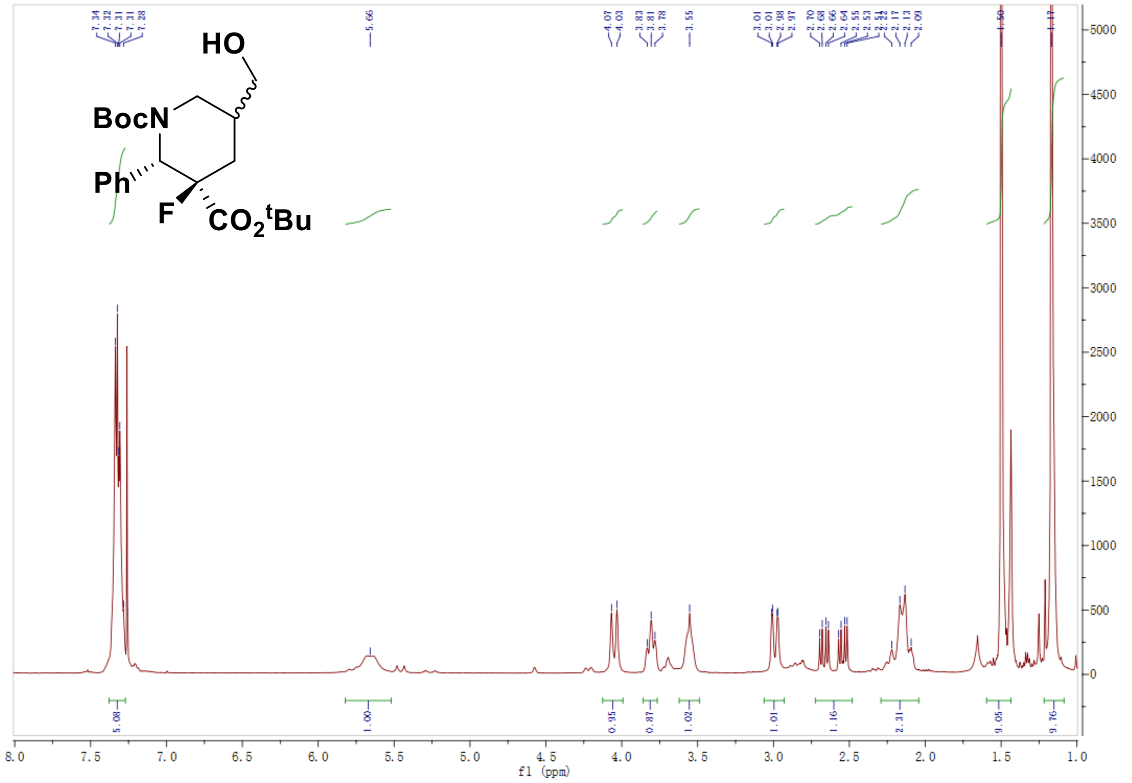

^{1}H NMR, $CDCl_3$, 400 MHz (9-BBN reduction)

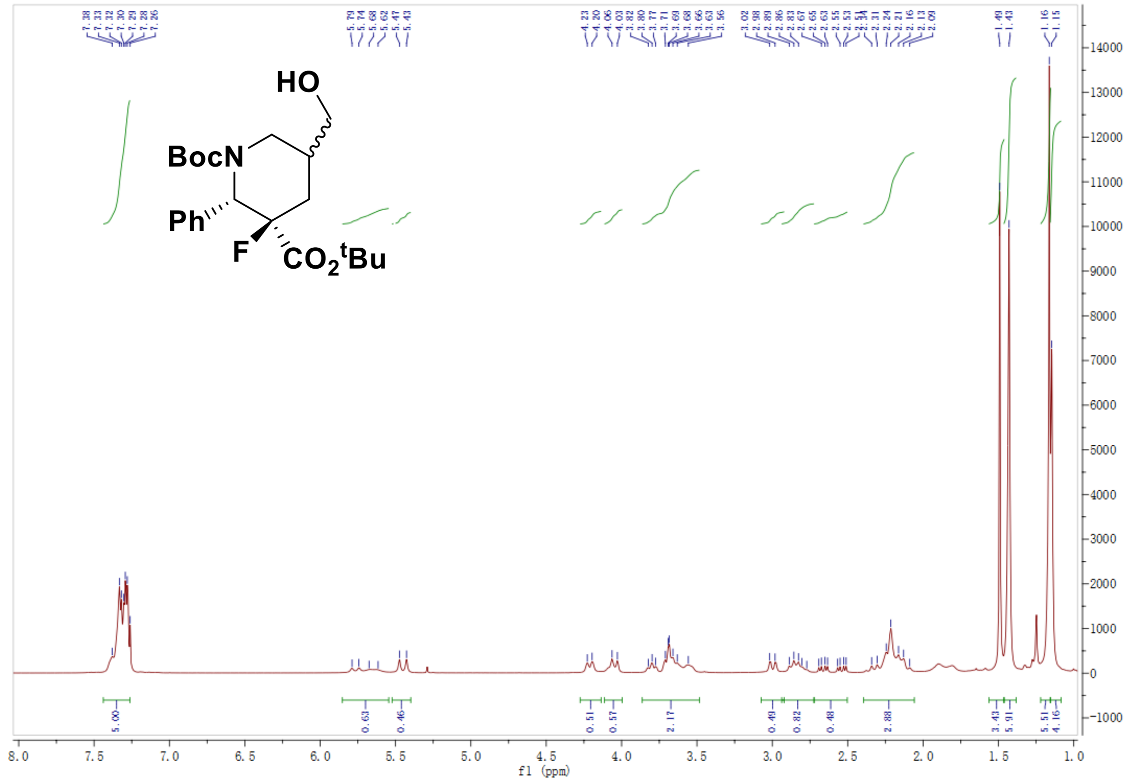

^{19}F NMR, CDCl$_3$, 377 MHz (borane reduction)

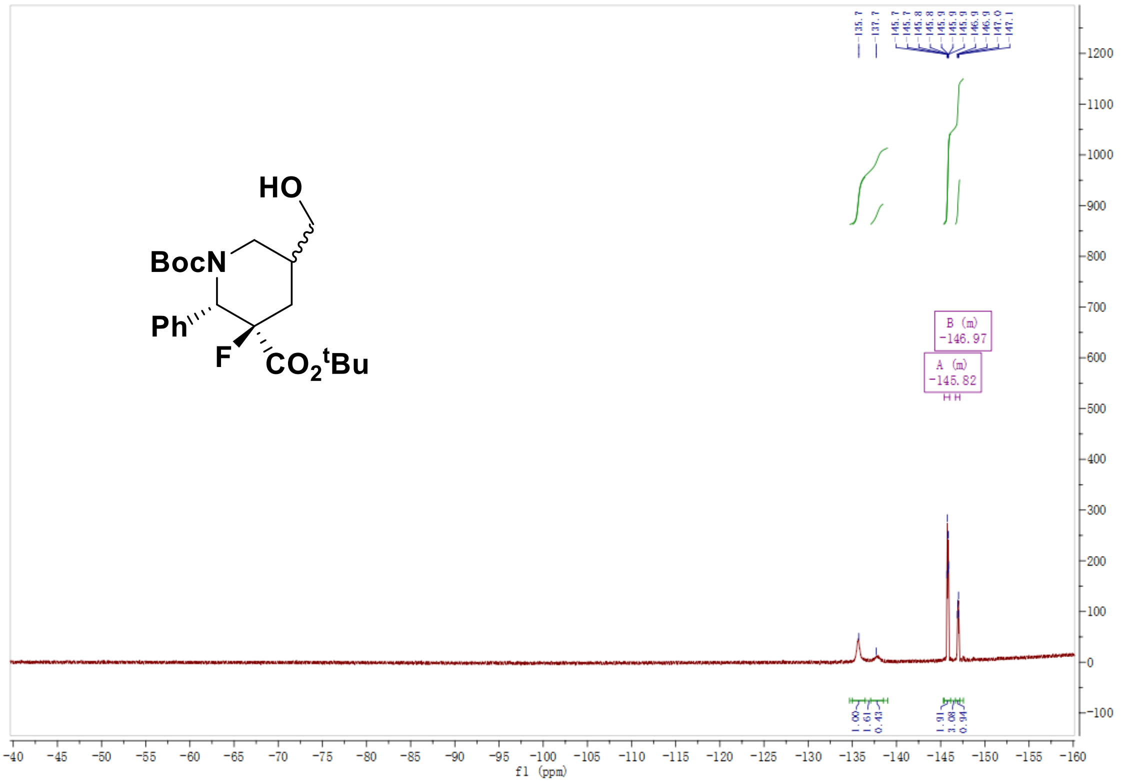

^{19}F NMR, CDCl$_3$, 377 MHz (9-BBN reduction)

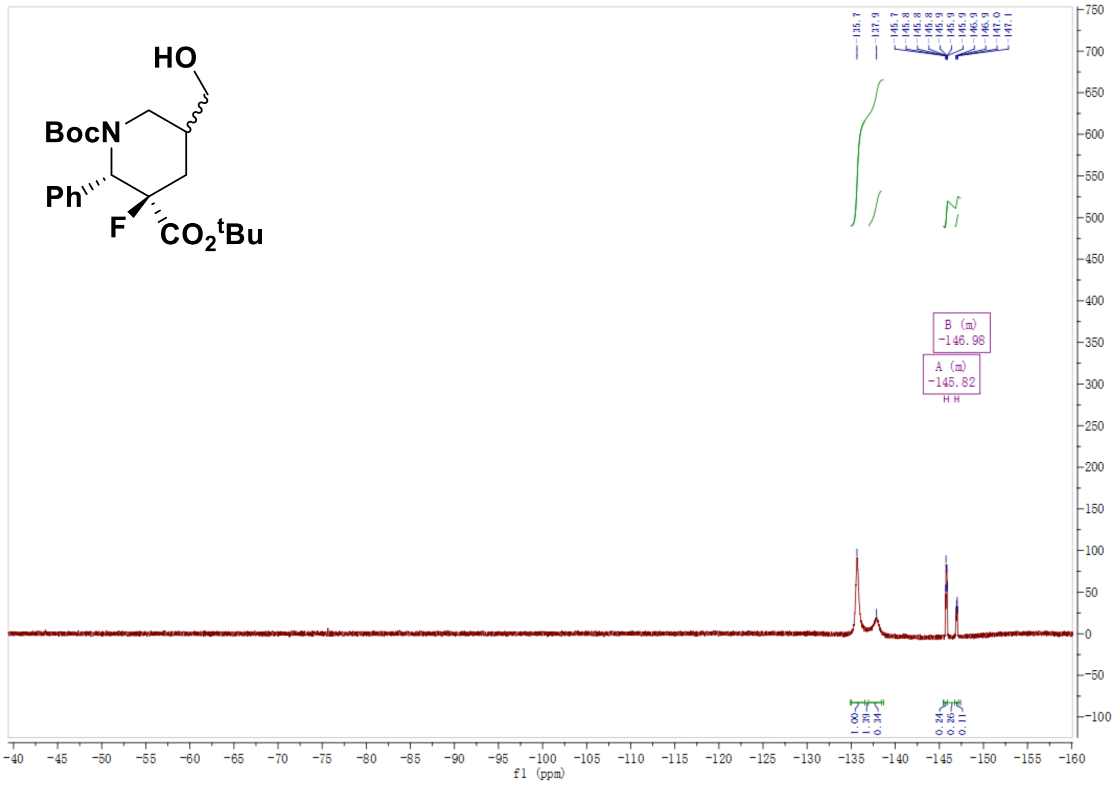

^{13}C NMR, $CDCl_3$, 101 MHz (borane reduction)

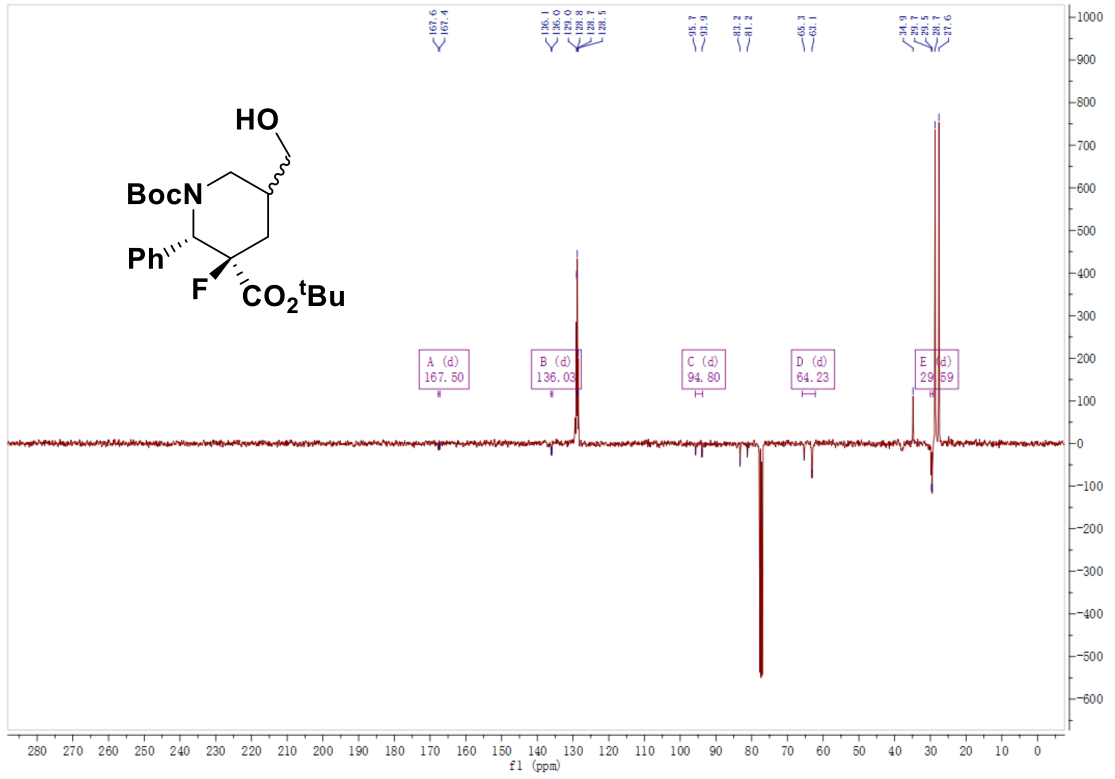

^{13}C NMR, $CDCl_3$, 101 MHz (9-BBN reduction)

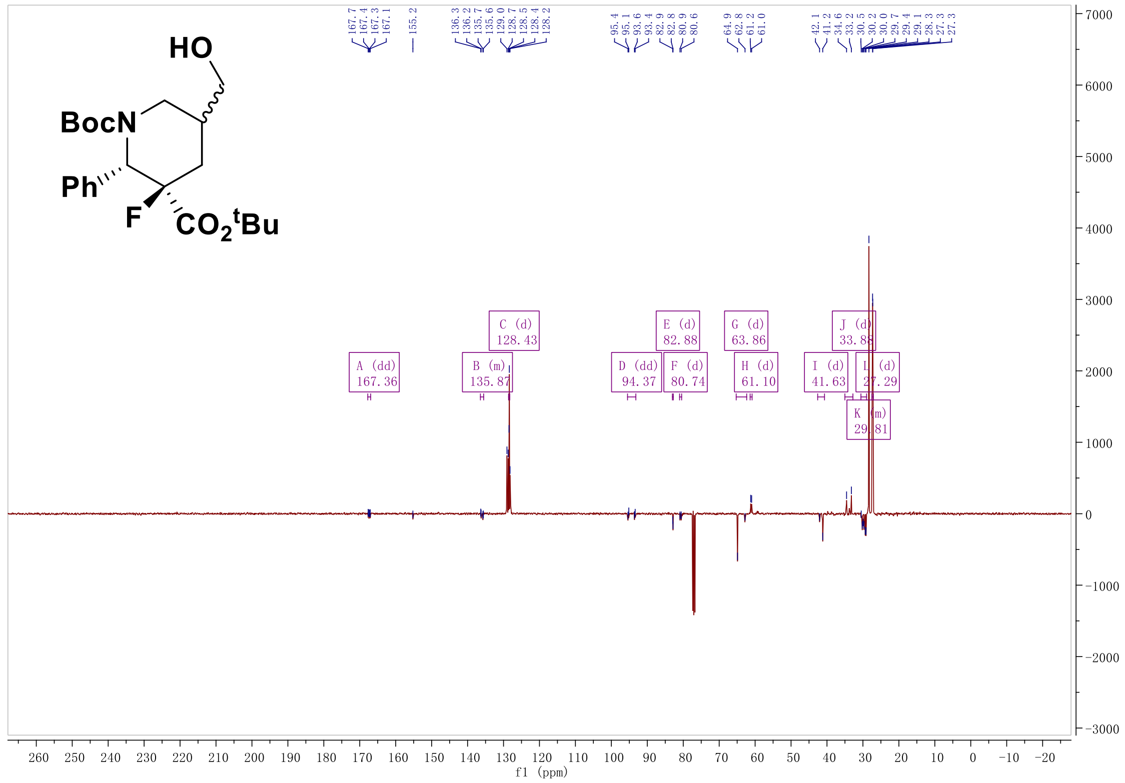

HPLC Traces

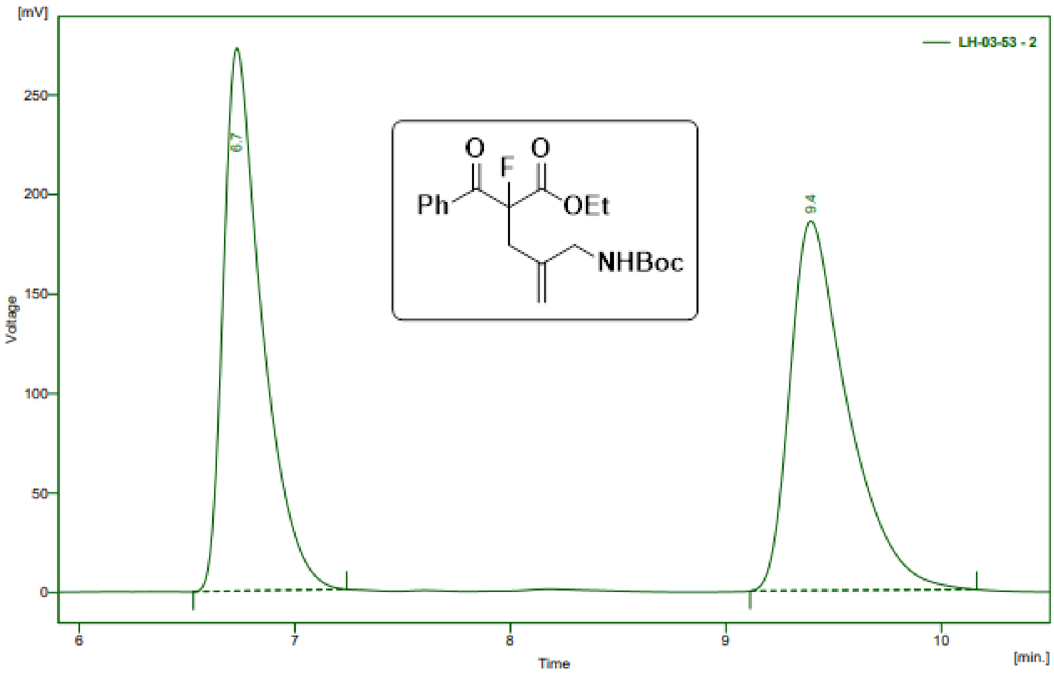

Result Table - Calculation Method Uncal

	Reten. Time [min]	Area [mV.s]	Height [mV]	Area [%]	Height [%]	W05 [min]
1	6.730	3413.484	272.845	49.6	59.5	0.19
2	9.393	3466.846	185.778	50.4	40.5	0.28
	Total	6880.329	458.623	100.0	100.0	

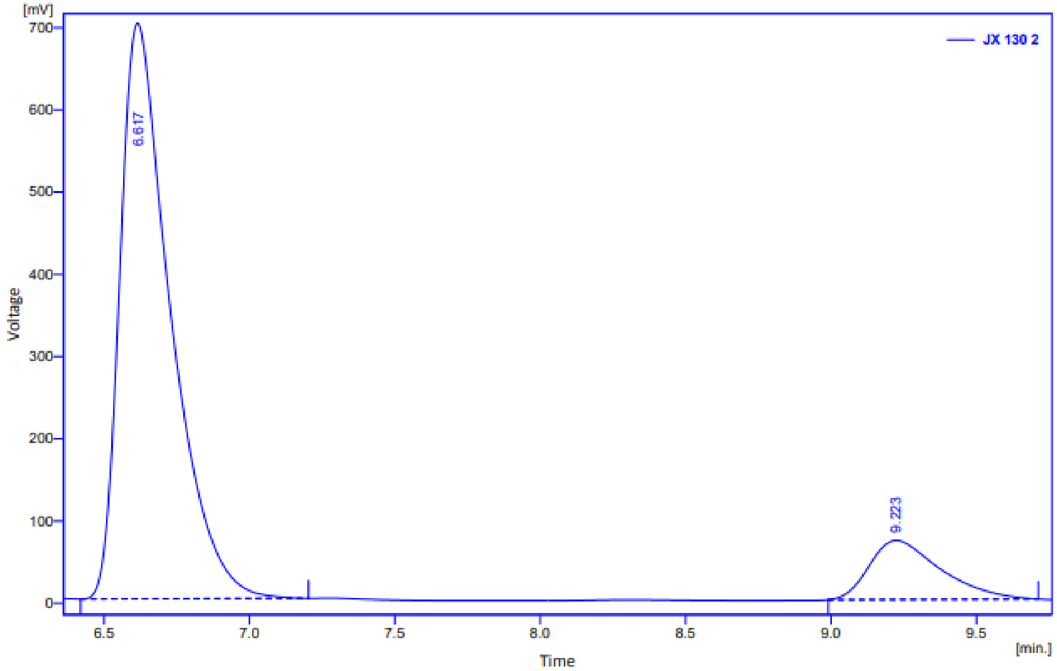

Result Table - Calculation Method Uncal

	Reten. Time [min]	Area [mV.s]	Height [mV]	Area [%]	Height [%]	W05 [min]
1	6.617	8457.831	699.950	87.4	90.7	0.18
2	9.223	1220.211	71.973	12.6	9.3	0.26
	Total	9678.042	771.923	100.0	100.0	

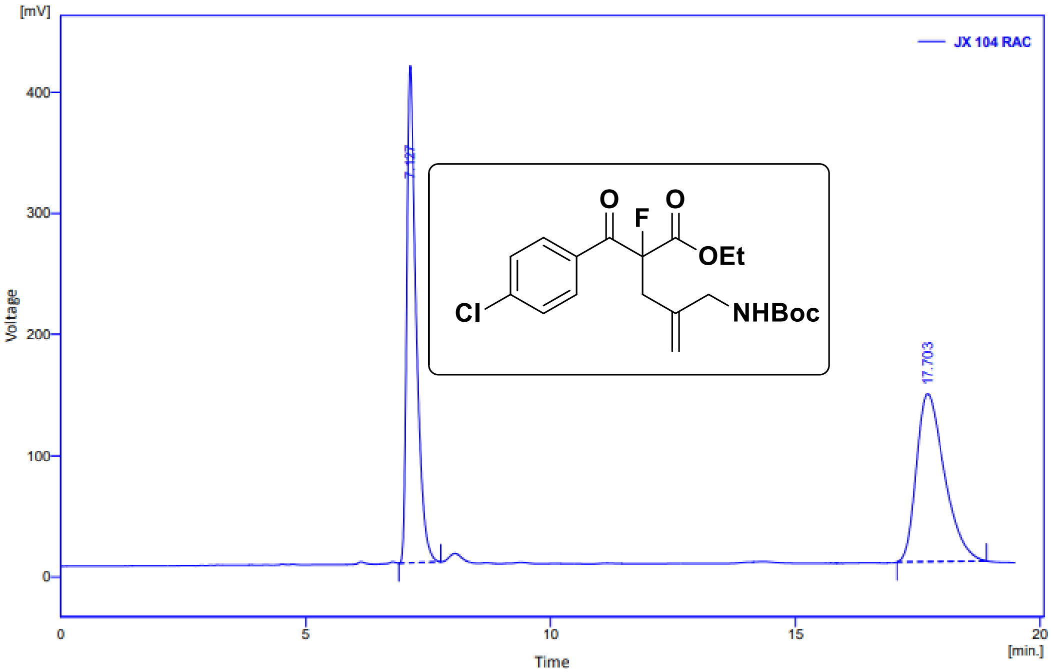

Result Table - Calculation Method Uncal

	Reten. Time [min]	Area [mV.s]	Height [mV]	Area [%]	Height [%]	W05 [min]
1	7.127	5627.052	410.432	50.4	74.7	0.21
2	17.703	5539.728	138.779	49.6	25.3	0.62
	Total	11166.780	549.212	100.0	100.0	

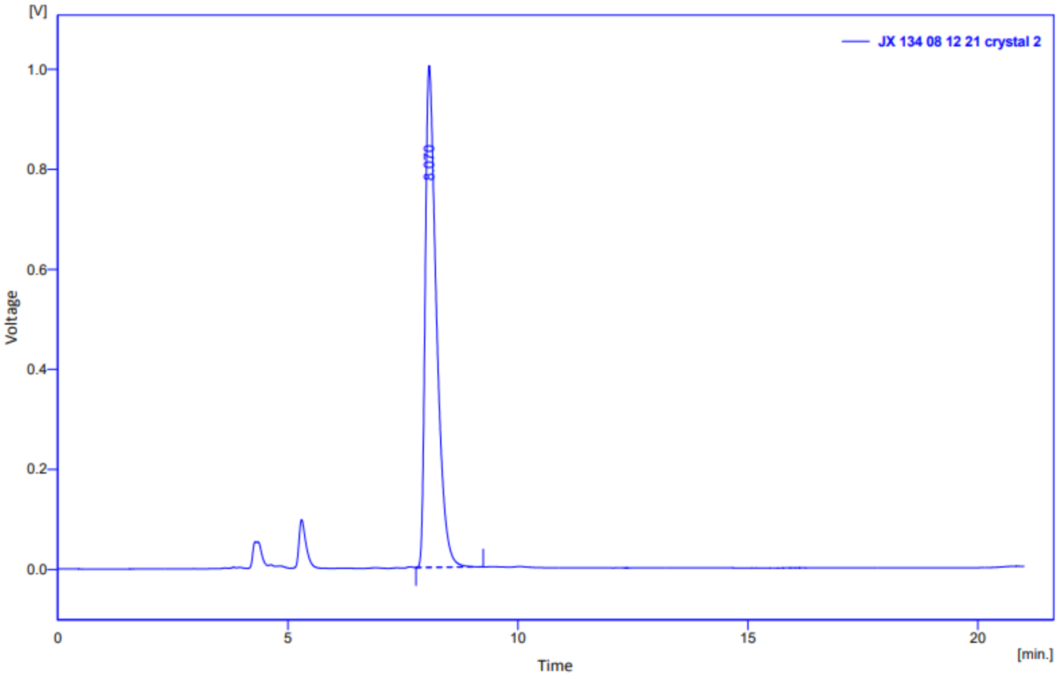

Result Table - Calculation Method Uncal

	Reten. Time [min]	Area [mV.s]	Height [mV]	Area [%]	Height [%]	W05 [min]
1	8.070	17049.277	1003.211	100.0	100.0	0.26
	Total	17049.277	1003.211	100.0	100.0	

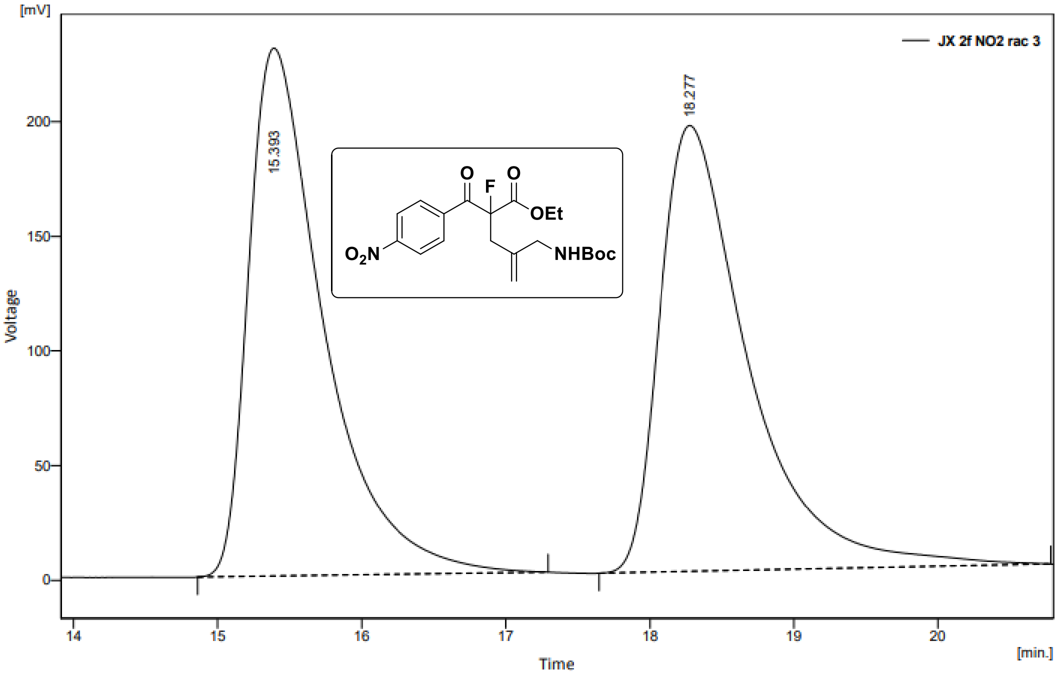

Result Table - Calculation Method Uncal

	Reten. Time [min]	Area [mV.s]	Height [mV]	Area [%]	Height [%]	W05 [min]
1	15.393	8322.978	230.171	49.9	54.2	0.53
2	18.277	8348.458	194.408	50.1	45.8	0.62
	Total	16671.436	424.580	100.0	100.0	

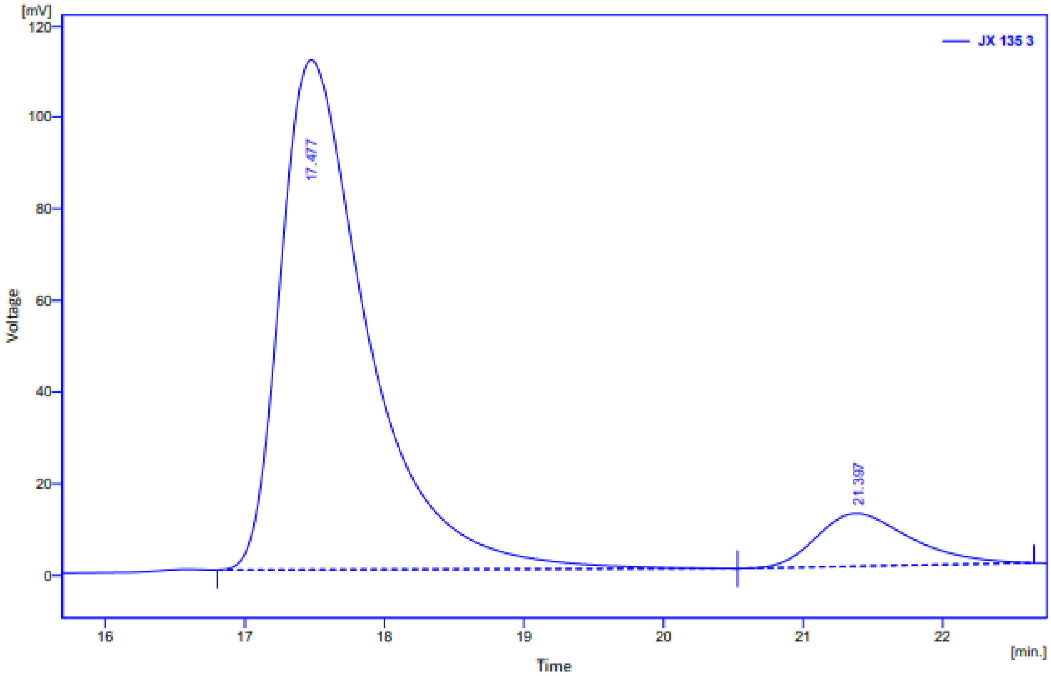

Result Table - Calculation Method Uncal

	Reten. Time [min]	Area [mV.s]	Height [mV]	Area [%]	Height [%]	W05 [min]
1	17.477	4851.648	111.547	90.0	90.7	0.62
2	21.397	541.185	11.495	10.0	9.3	0.73
	Total	5392.833	123.042	100.0	100.0	

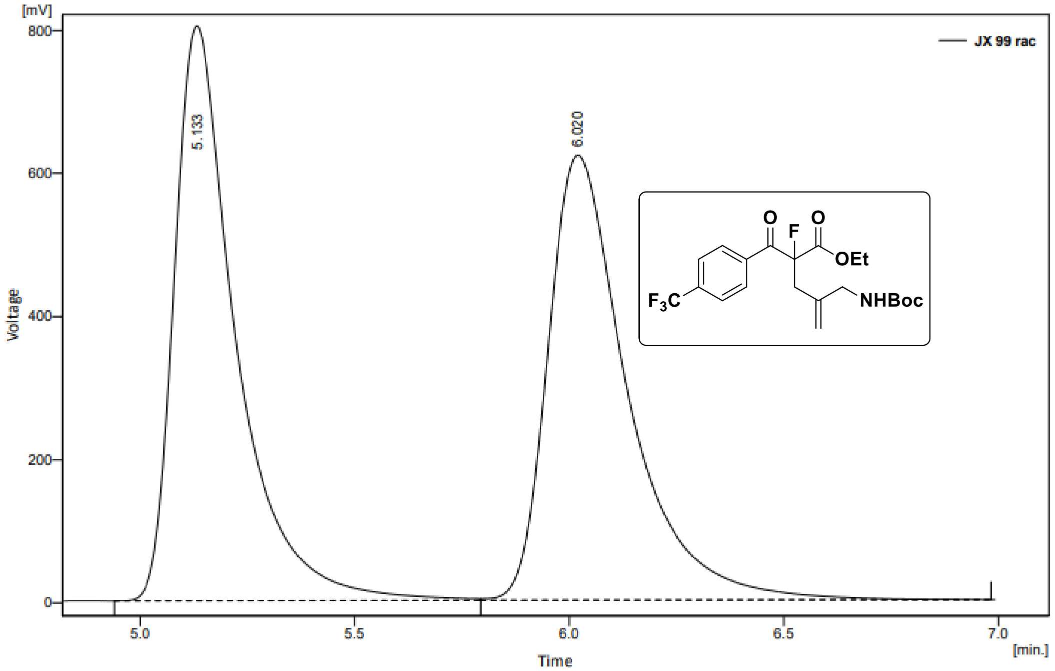

Result Table - Calculation Method Uncal

	Reten. Time [min]	Area [mV.s]	Height [mV]	Area [%]	Height [%]	W05 [min]
1	5.133	8032.259	803.050	49.9	56.4	0.14
2	6.020	8065.111	621.689	50.1	43.6	0.19
	Total	16097.371	1424.739	100.0	100.0	

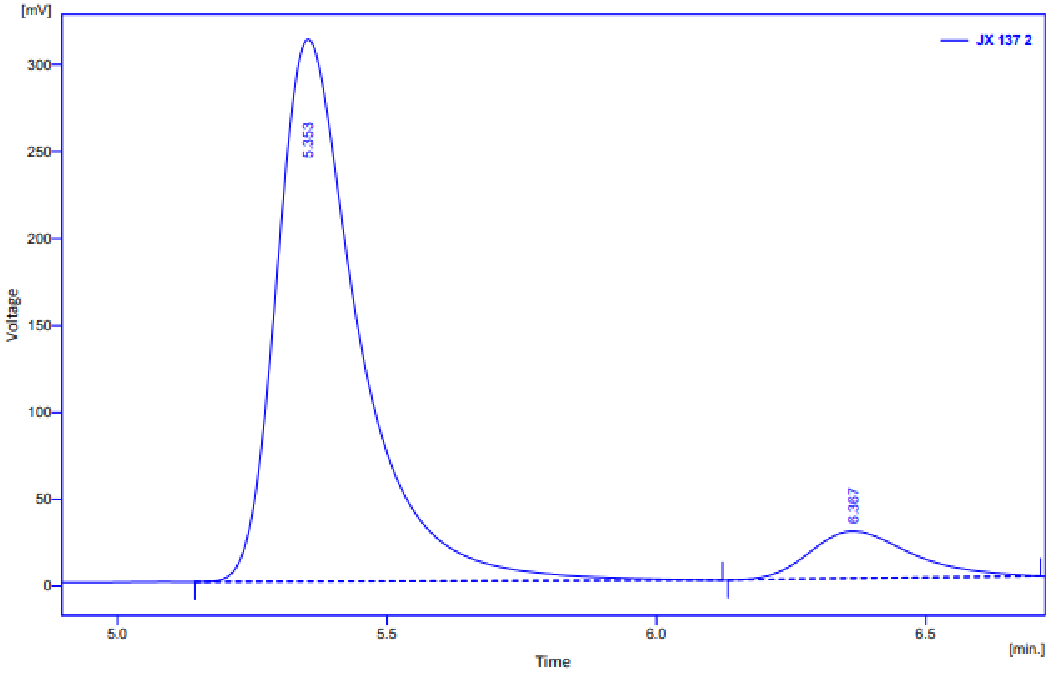

Result Table - Calculation Method Uncal

	Reten. Time [min]	Area [mV.s]	Height [mV]	Area [%]	Height [%]	W05 [min]
1	5.353	3315.628	311.866	90.3	92.0	0.15
2	6.367	356.404	27.152	9.7	8.0	0.20
	Total	3672.032	339.018	100.0	100.0	

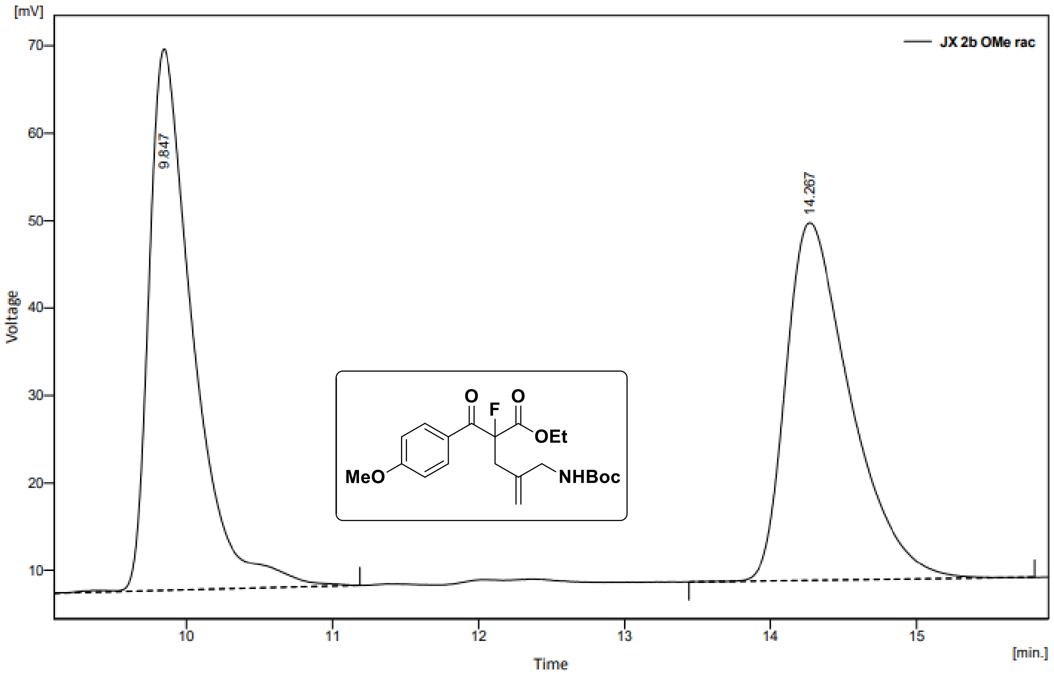

Result Table - Calculation Method Uncal

	Reten. Time [min]	Area [mV.s]	Height [mV]	Area [%]	Height [%]	W05 [min]
1	9.847	1298.282	61.911	50.8	60.2	0.30
2	14.267	1258.822	40.913	49.2	39.8	0.47
	Total	2557.104	102.824	100.0	100.0	

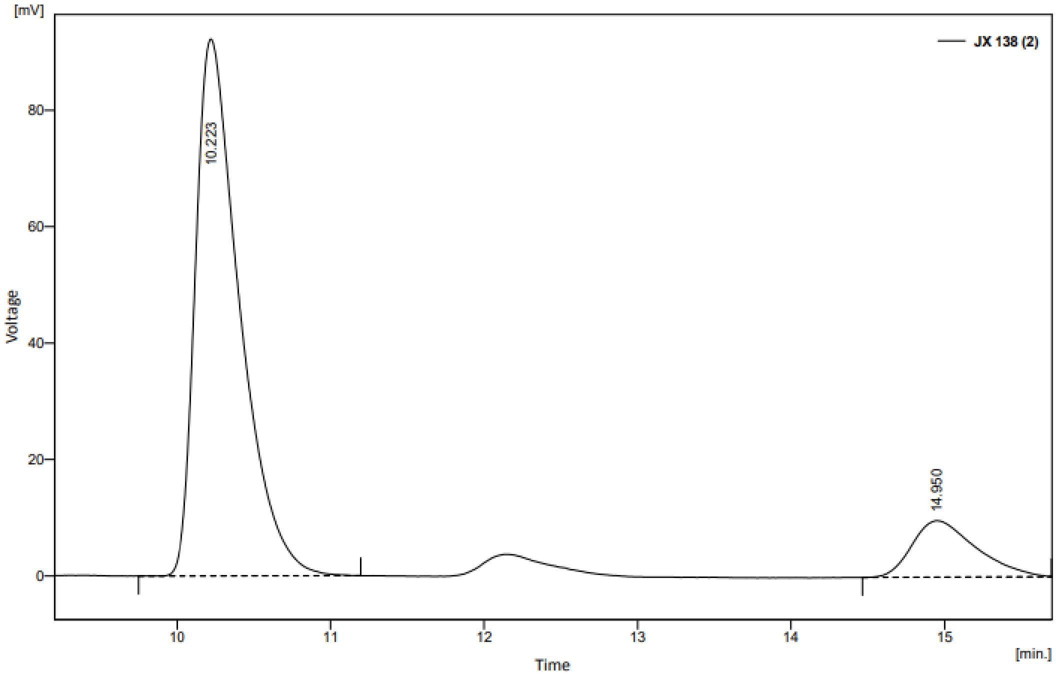

Result Table - Calculation Method Uncal

	Reten. Time [min]	Area [mV.s]	Height [mV]	Area [%]	Height [%]	W05 [min]
1	10.223	1837.814	92.243	86.9	90.5	0.30
2	14.950	278.039	9.658	13.1	9.5	0.45
	Total	2115.853	101.902	100.0	100.0	

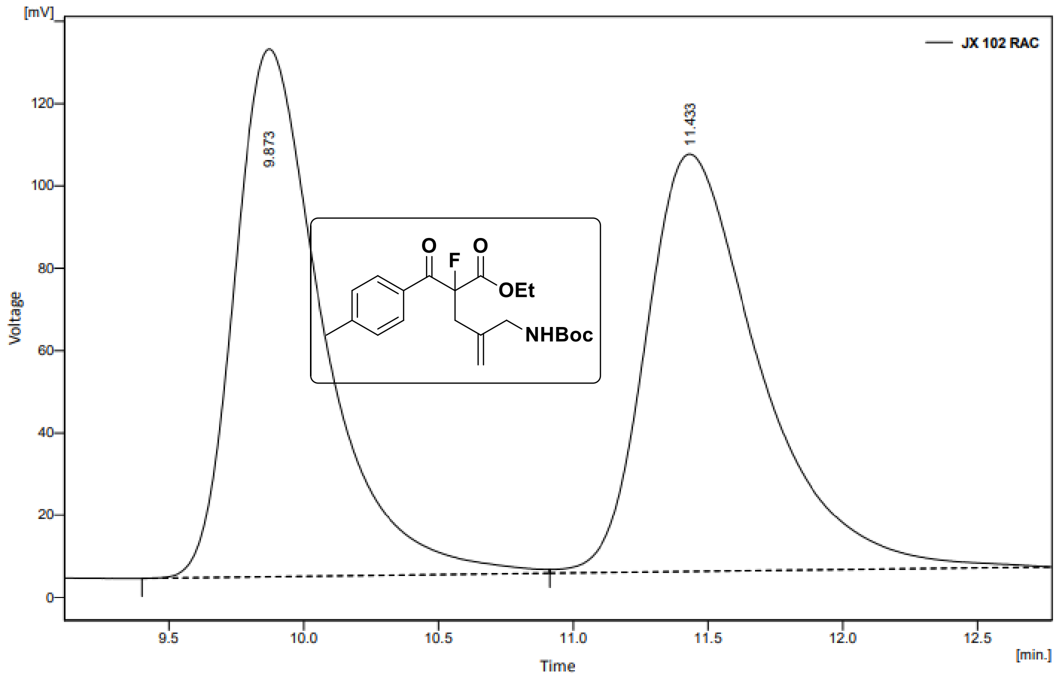

Result Table - Calculation Method Uncal

	Reten. Time [min]	Area [mV.s]	Height [mV]	Area [%]	Height [%]	W05 [min]
1	9.873	2942.053	128.104	49.5	55.8	0.34
2	11.433	2996.364	101.368	50.5	44.2	0.44
	Total	5938.417	229.472	100.0	100.0	

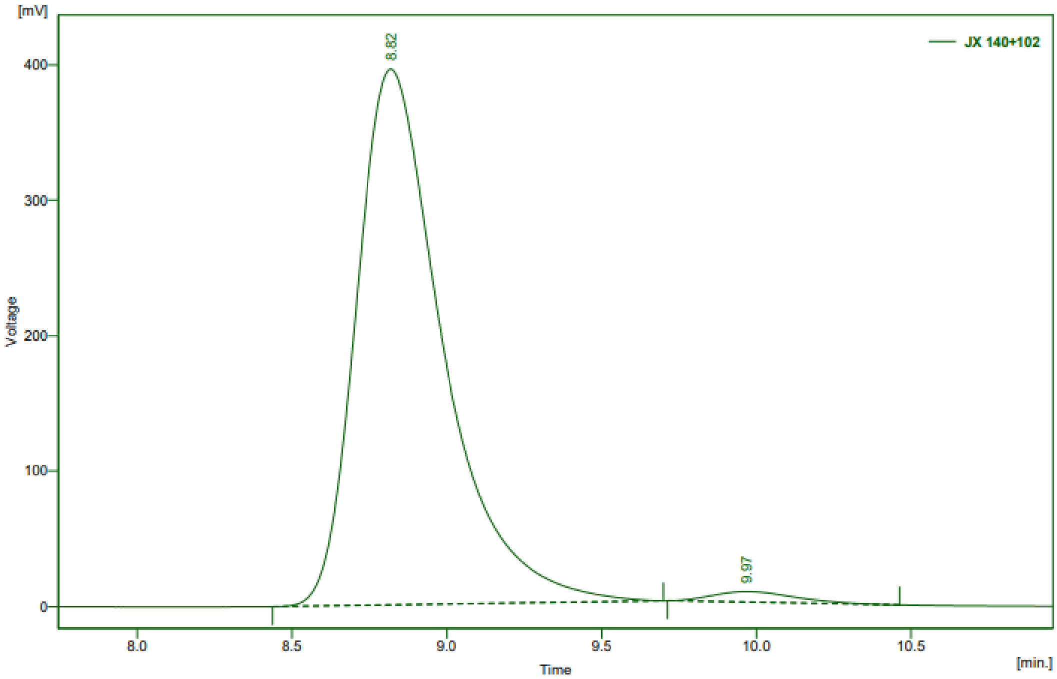

Result Table - Calculation Method Uncal

	Reten. Time [min]	Area [mV.s]	Height [mV]	Area [%]	Height [%]	W05 [min]
1	8.820	7687.398	395.576	98.1	98.1	0.29
2	9.970	146.507	7.835	1.9	1.9	0.29
	Total	7833.905	403.411	100.0	100.0	

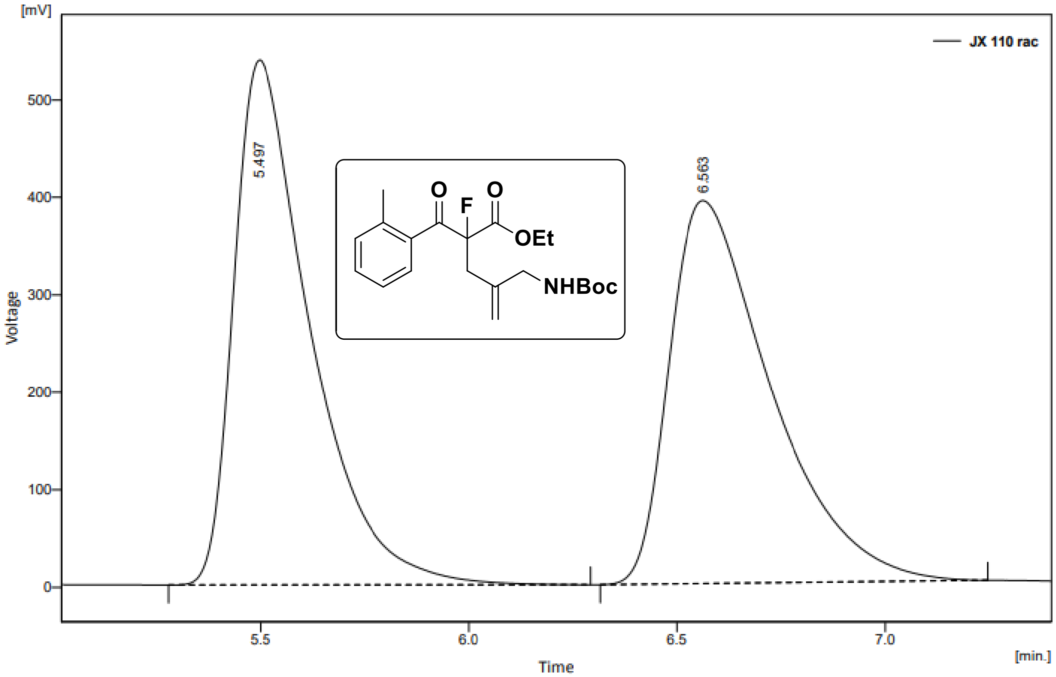

Result Table - Calculation Method Uncal

	Reten. Time [min]	Area [mV.s]	Height [mV]	Area [%]	Height [%]	W05 [min]
1	5.497	6858.209	538.620	50.7	57.8	0.19
2	6.563	6667.813	392.723	49.3	42.2	0.26
	Total	13526.022	931.343	100.0	100.0	

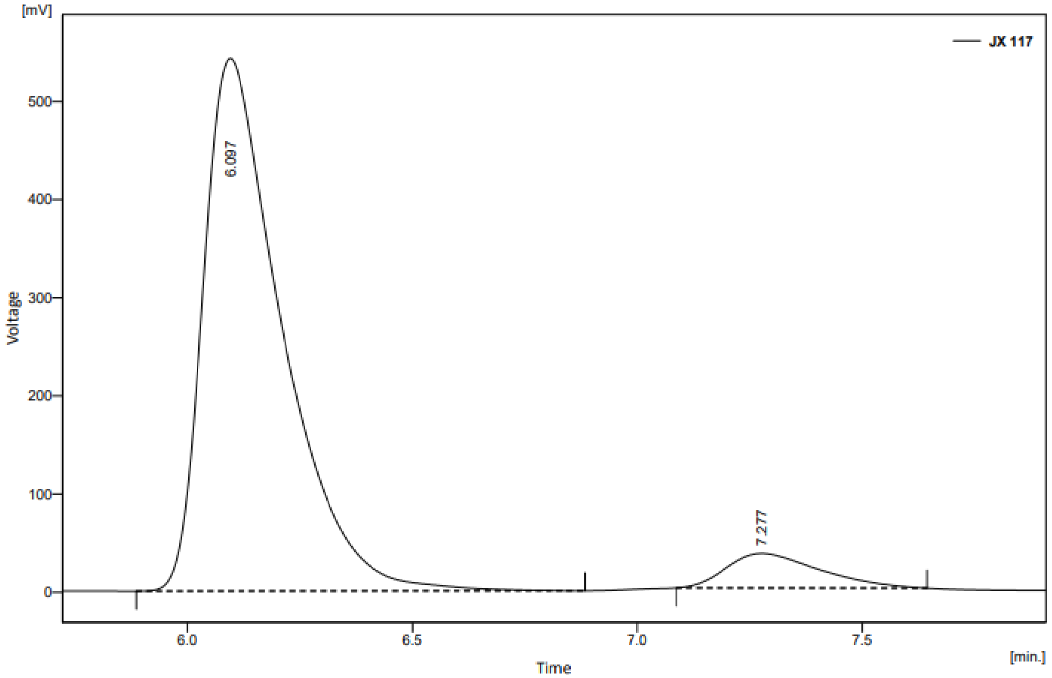

Result Table - Calculation Method Uncal

	Reten. Time [min]	Area [mV.s]	Height [mV]	Area [%]	Height [%]	W05 [min]
1	6.097	6574.472	542.660	92.9	93.9	0.18
2	7.277	504.736	35.129	7.1	6.1	0.23
	Total	7079.208	577.789	100.0	100.0	

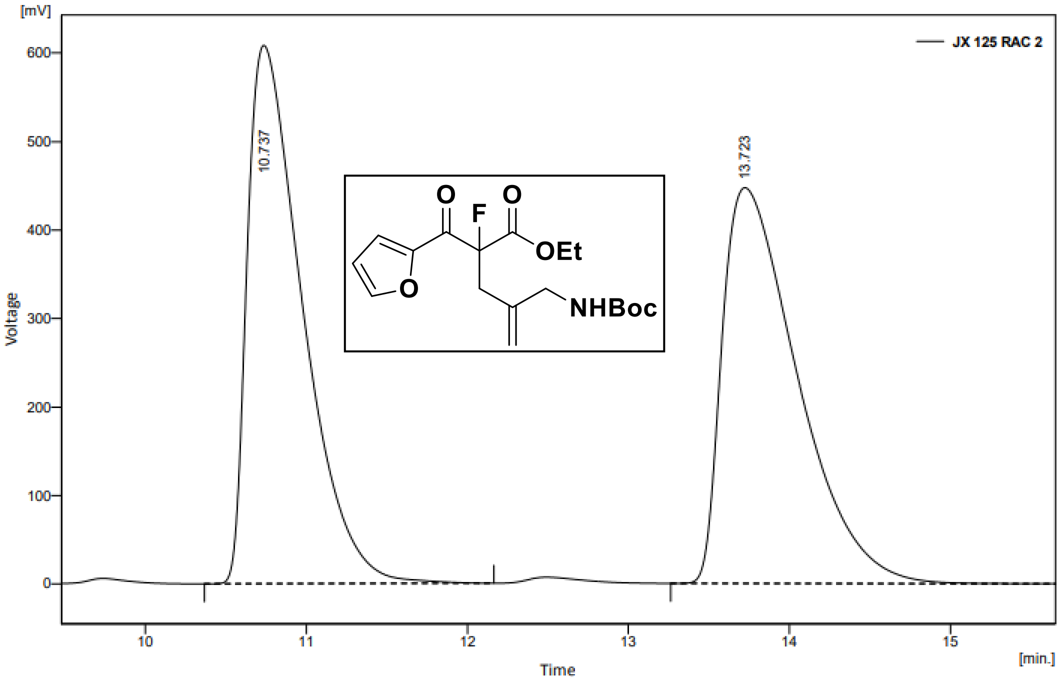

Result Table - Calculation Method Uncal

	Reten. Time [min]	Area [mV.s]	Height [mV]	Area [%]	Height [%]	W05 [min]
1	10.737	14496.283	608.395	49.9	57.6	0.37
2	13.723	14536.514	447.575	50.1	42.4	0.51
	Total	29032.797	1055.970	100.0	100.0	

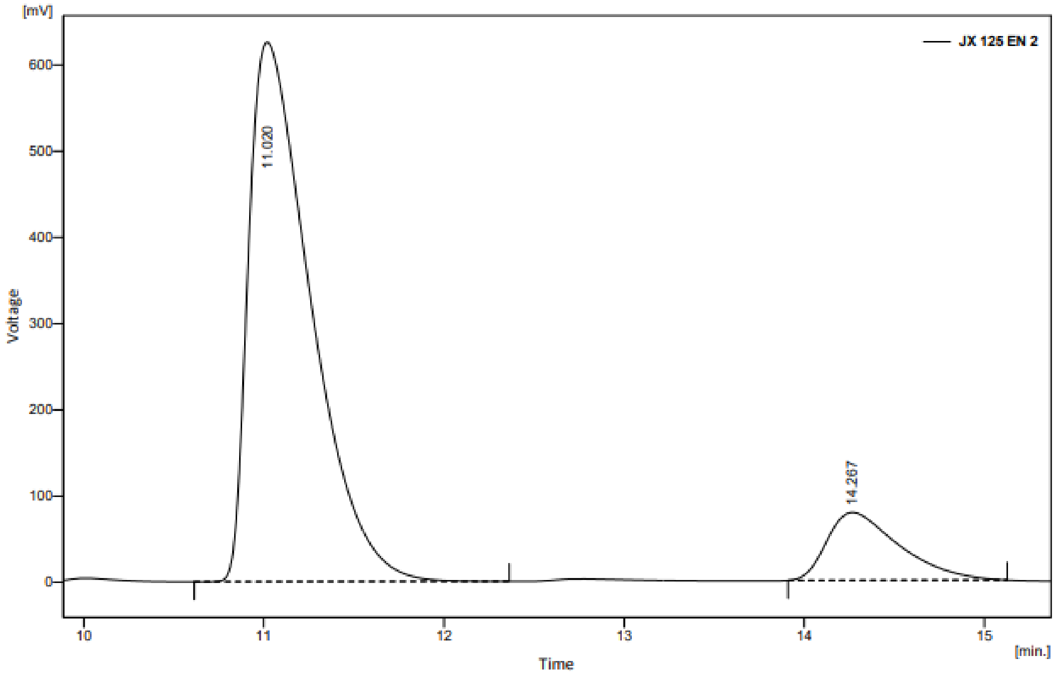

Result Table - Calculation Method Uncal

	Reten. Time [min]	Area [mV.s]	Height [mV]	Area [%]	Height [%]	W05 [min]
1	11.020	15007.810	625.905	87.3	88.8	0.37
2	14.267	2189.724	78.705	12.7	11.2	0.43
	Total	17197.533	704.610	100.0	100.0	

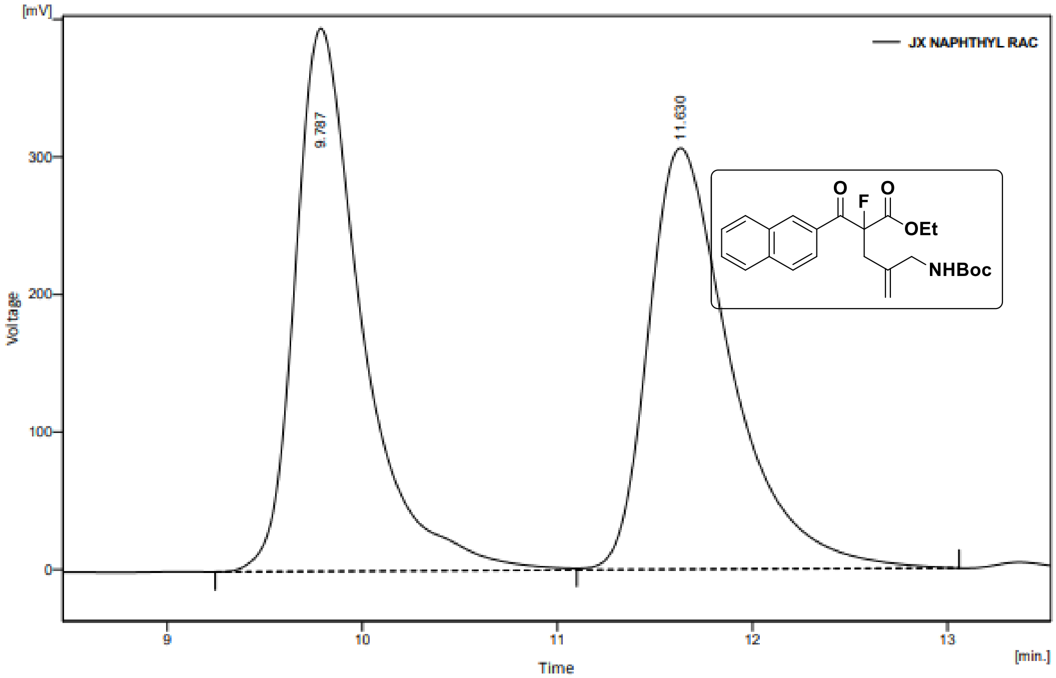

Result Table - Calculation Method Uncal

	Reten. Time [min]	Area [mV.s]	Height [mV]	Area [%]	Height [%]	W05 [min]
1	9.787	9433.867	394.619	50.9	56.3	0.34
2	11.630	9117.082	306.304	49.1	43.7	0.44
	Total	18550.949	700.923	100.0	100.0	

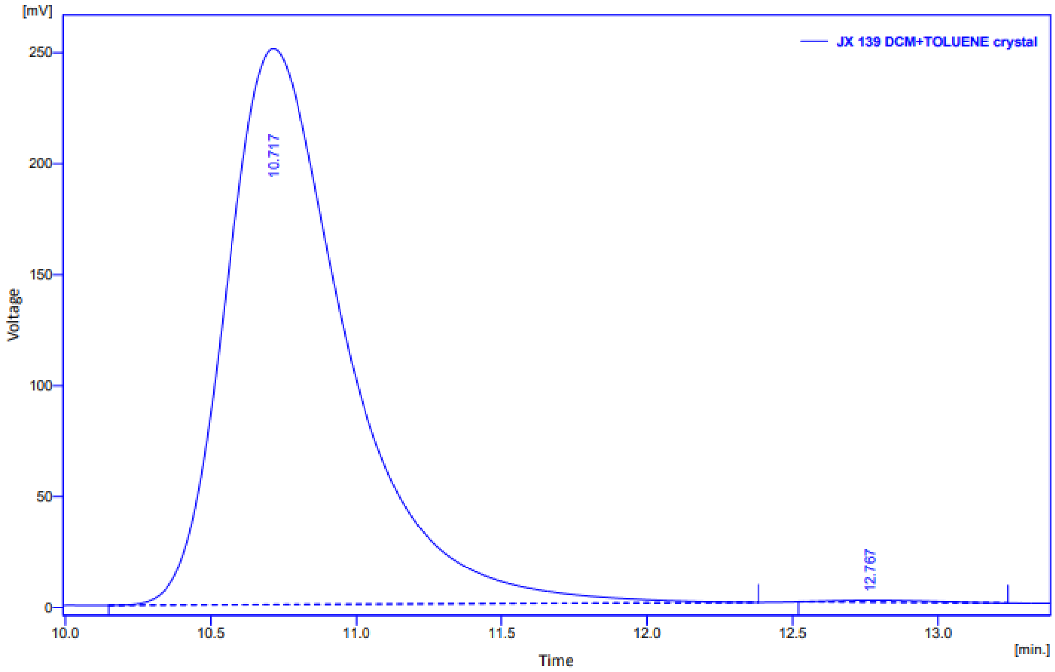

Result Table - Calculation Method Uncal

	Reten. Time [min]	Area [mV.s]	Height [mV]	Area [%]	Height [%]	W05 [min]
1	10.717	7203.743	250.347	99.7	99.6	0.42
2	12.767	21.833	0.901	0.3	0.4	0.38
	Total	7225.576	251.248	100.0	100.0	

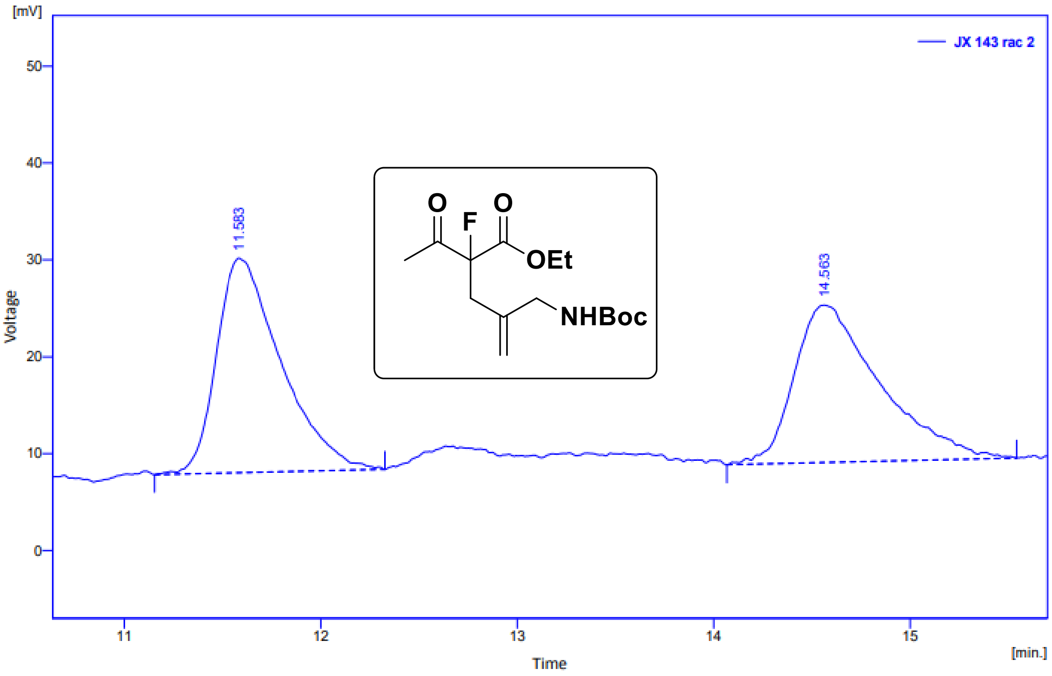

Result Table - Calculation Method Uncal

	Reten. Time [min]	Area [mV.s]	Height [mV]	Area [%]	Height [%]	W05 [min]
1	11.583	504.597	22.112	50.6	57.6	0.35
2	14.563	493.554	16.258	49.4	42.4	0.45
	Total	998.152	38.370	100.0	100.0	

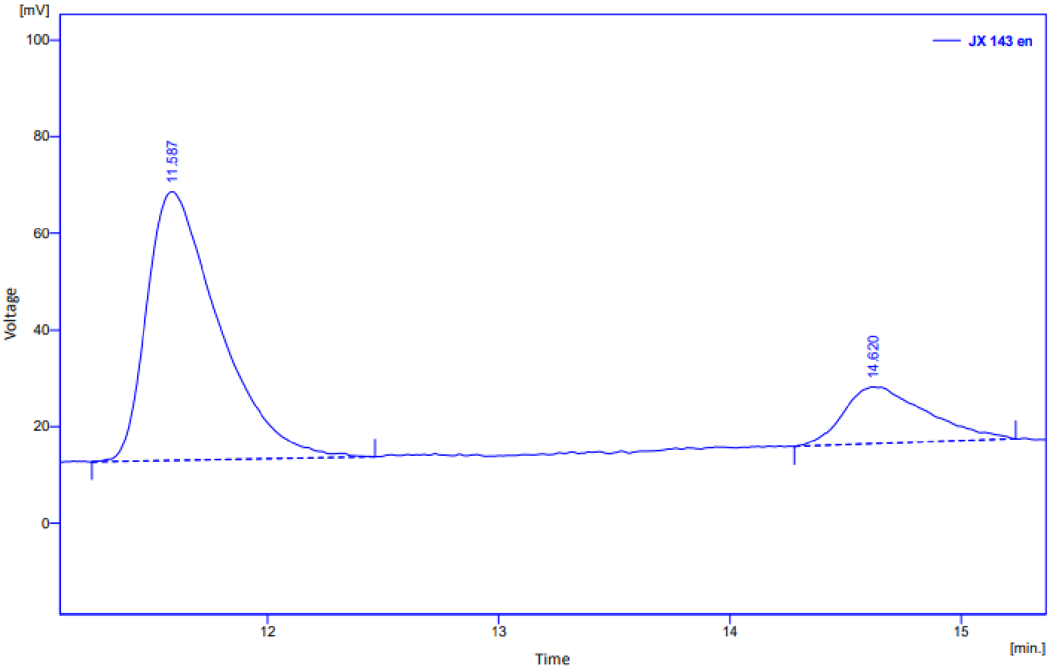

Result Table - Calculation Method Uncal

	Reten. Time [min]	Area [mV.s]	Height [mV]	Area [%]	Height [%]	W05 [min]
1	11.587	1205.905	55.701	80.3	82.5	0.33
2	14.620	295.157	11.775	19.7	17.5	0.40
	Total	1501.062	67.476	100.0	100.0	

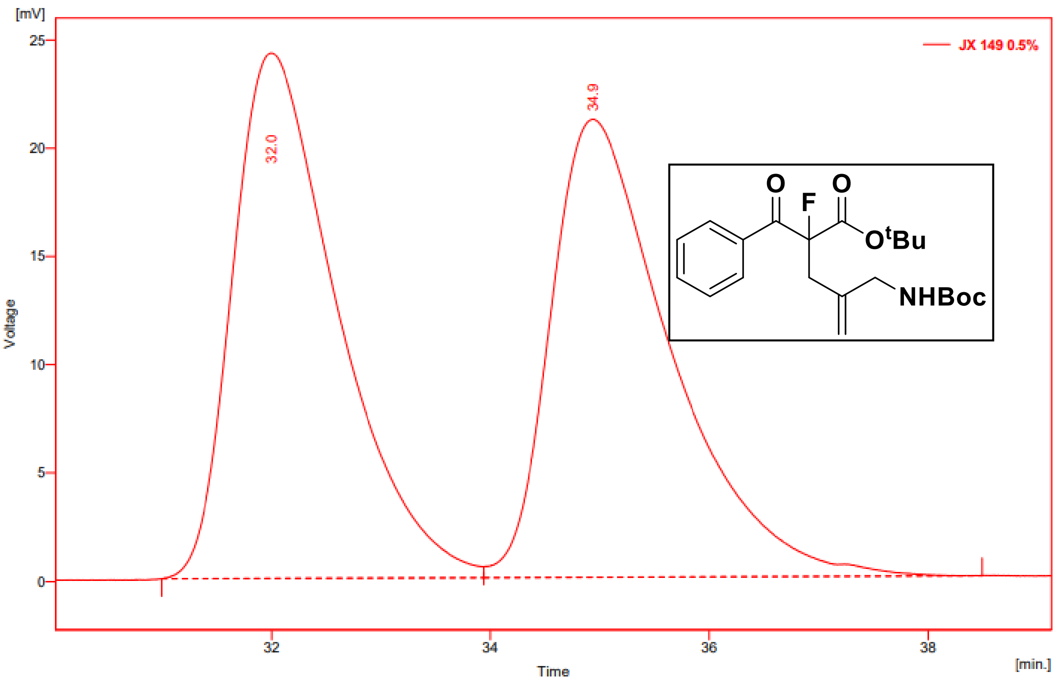

Result Table - Calculation Method Uncal

	Reten. Time [min]	Area [mV.s]	Height [mV]	Area [%]	Height [%]	W05 [min]
1	32.000	1615.333	24.253	50.0	53.4	1.01
2	34.937	1612.580	21.137	50.0	46.6	1.13
	Total	3227.913	45.390	100.0	100.0	

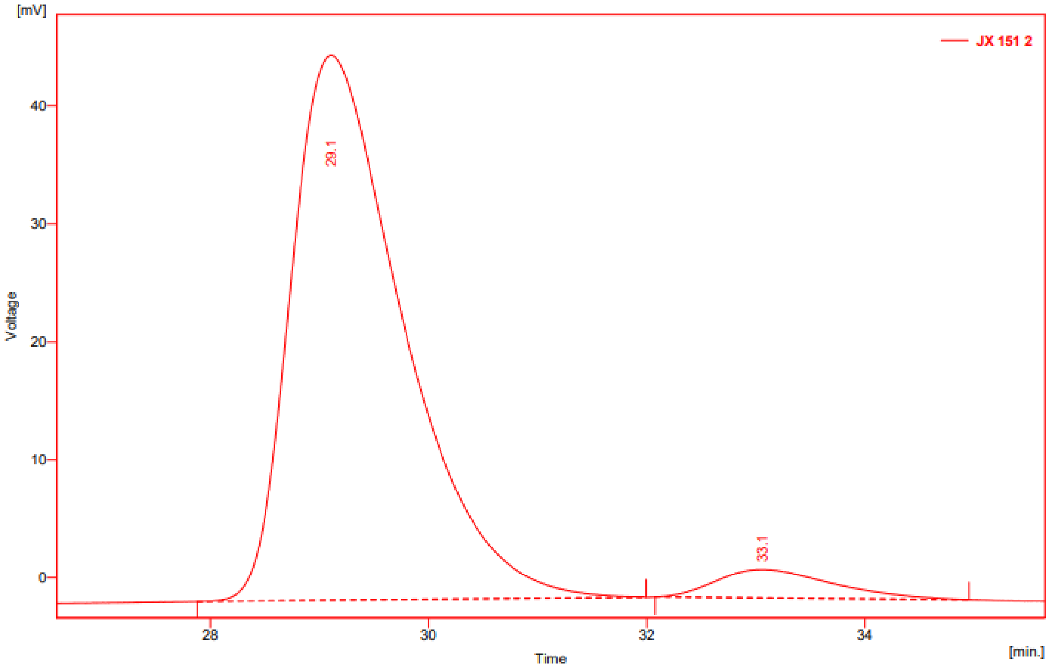

Result Table - Calculation Method Uncal

	Reten. Time [min]	Area [mV.s]	Height [mV]	Area [%]	Height [%]	W05 [min]
1	29.107	3290.714	46.206	94.9	95.1	1.09
2	33.060	178.537	2.403	5.1	4.9	1.14
	Total	3469.251	48.609	100.0	100.0	

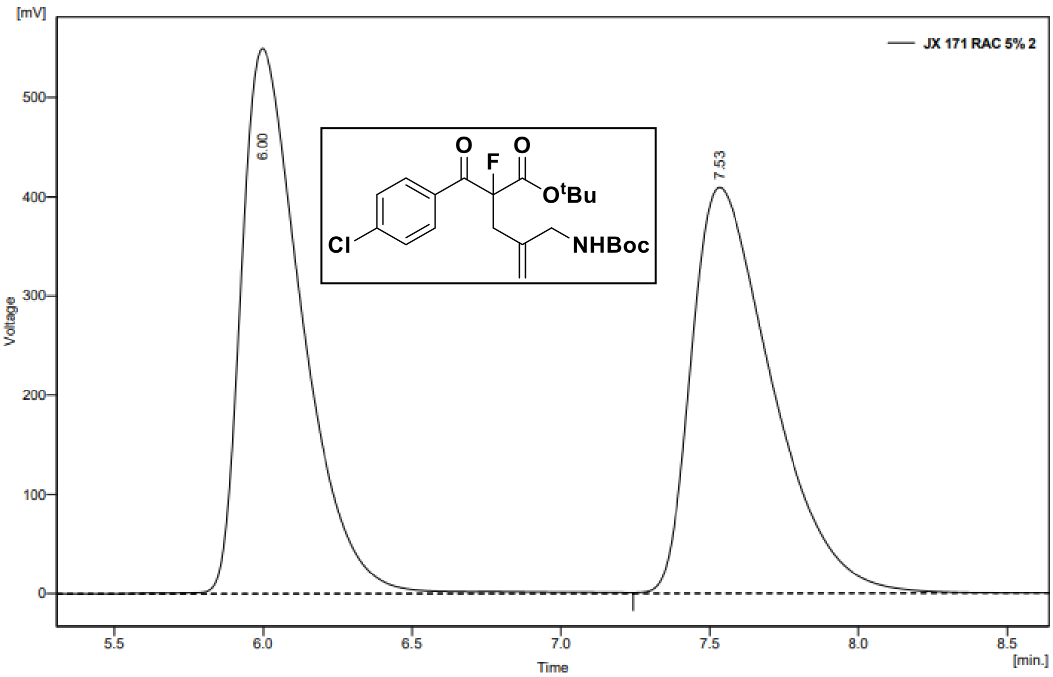

Result Table - Calculation Method Uncal

	Reten. Time [min]	Area [mV.s]	Height [mV]	Area [%]	Height [%]	W05 [min]
1	5.997	7847.601	549.837	50.4	57.3	0.22
2	7.533	7711.772	409.710	49.6	42.7	0.29
	Total	15559.373	959.547	100.0	100.0	

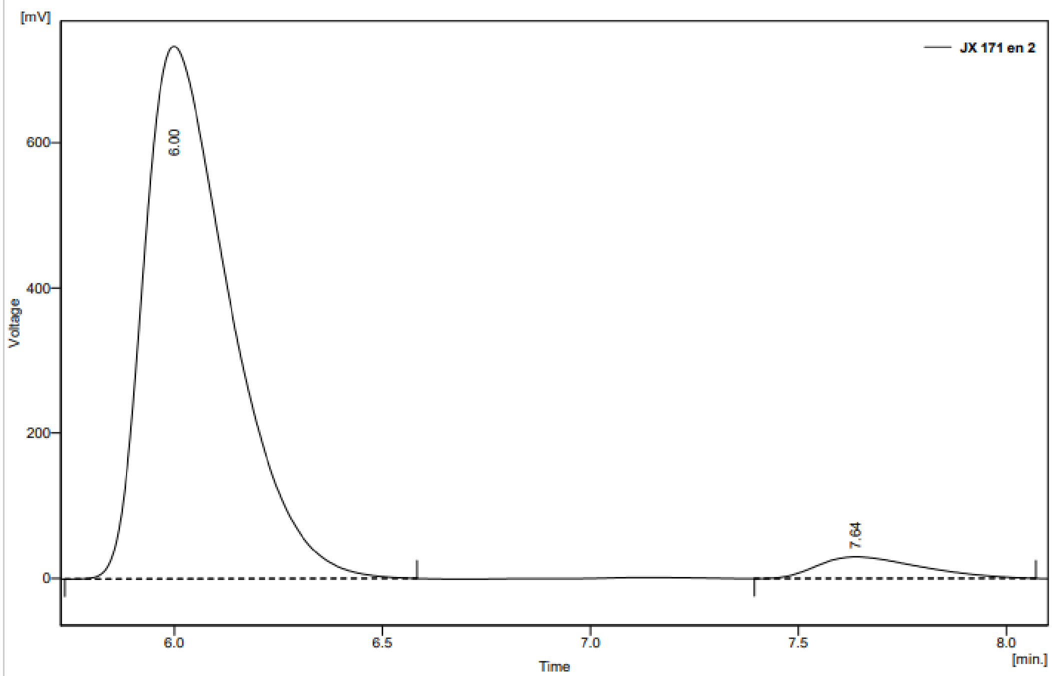

Result Table - Calculation Method Uncal

	Reten. Time [min]	Area [mV.s]	Height [mV]	Area [%]	Height [%]	W05 [min]
1	6.000	10542.544	733.410	95.4	96.1	0.22
2	7.637	509.796	29.493	4.6	3.9	0.28
	Total	11052.340	762.903	100.0	100.0	

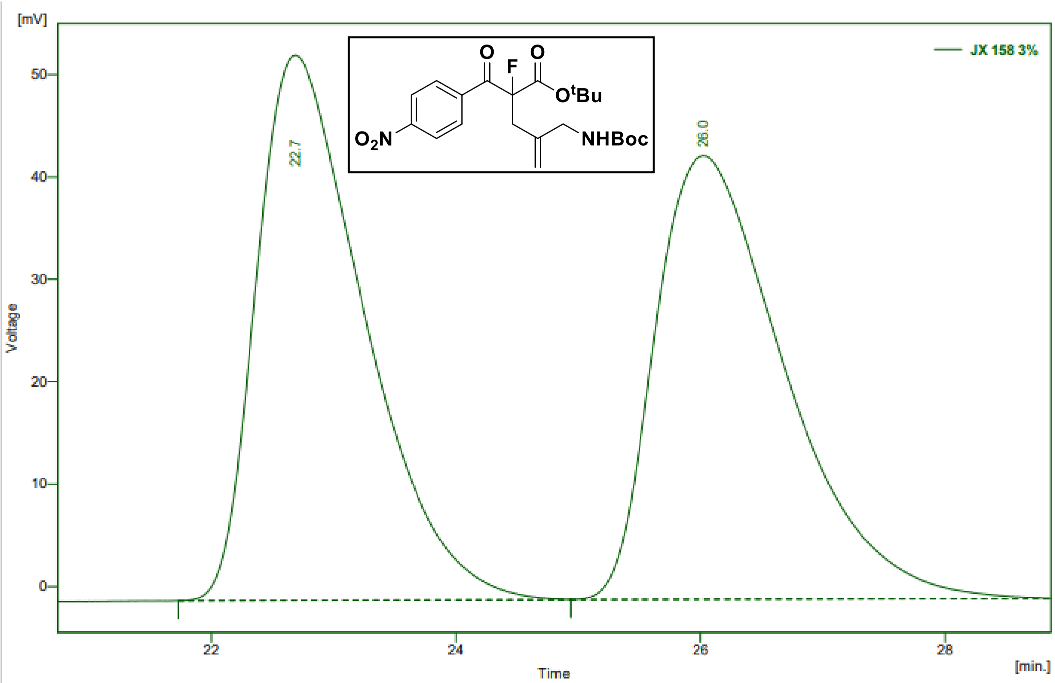

Result Table - Calculation Method Uncal

	Reten. Time [min]	Area [mV.s]	Height [mV]	Area [%]	Height [%]	W05 [min]
1	22.683	3264.334	53.264	50.5	55.1	0.95
2	26.023	3198.249	43.355	49.5	44.9	1.14
	Total	6462.583	96.619	100.0	100.0	

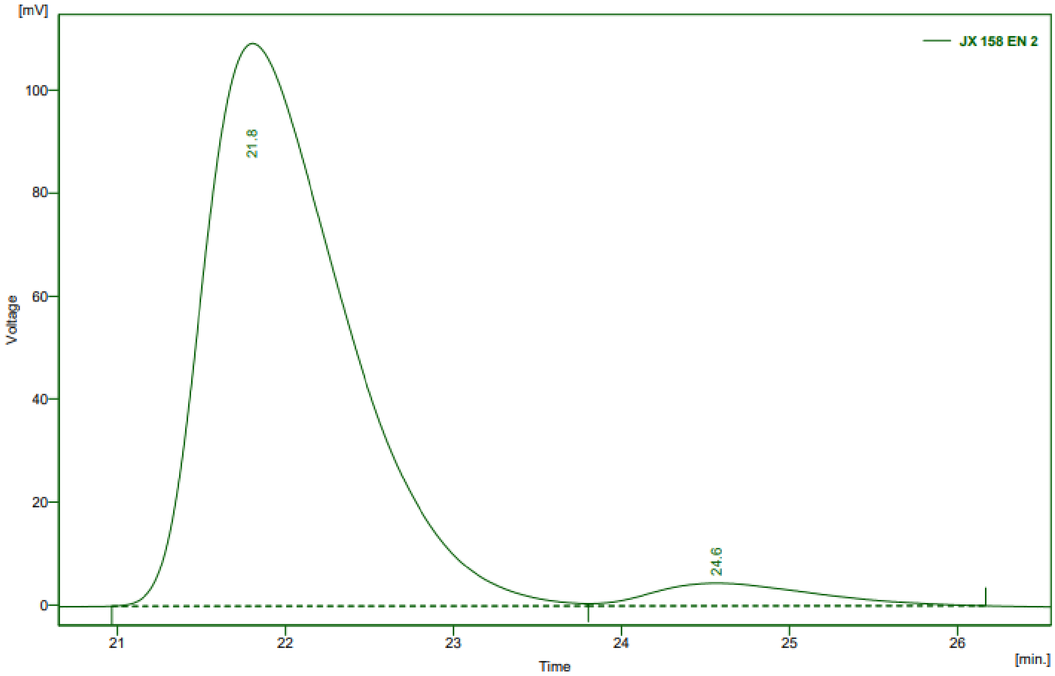

Result Table - Calculation Method Uncal

	Reten. Time [min]	Area [mV.s]	Height [mV]	Area [%]	Height [%]	W05 [min]
1	21.803	6332.865	109.243	95.6	96.1	0.90
2	24.567	294.796	4.416	4.4	3.9	1.06
	Total	6627.661	113.659	100.0	100.0	

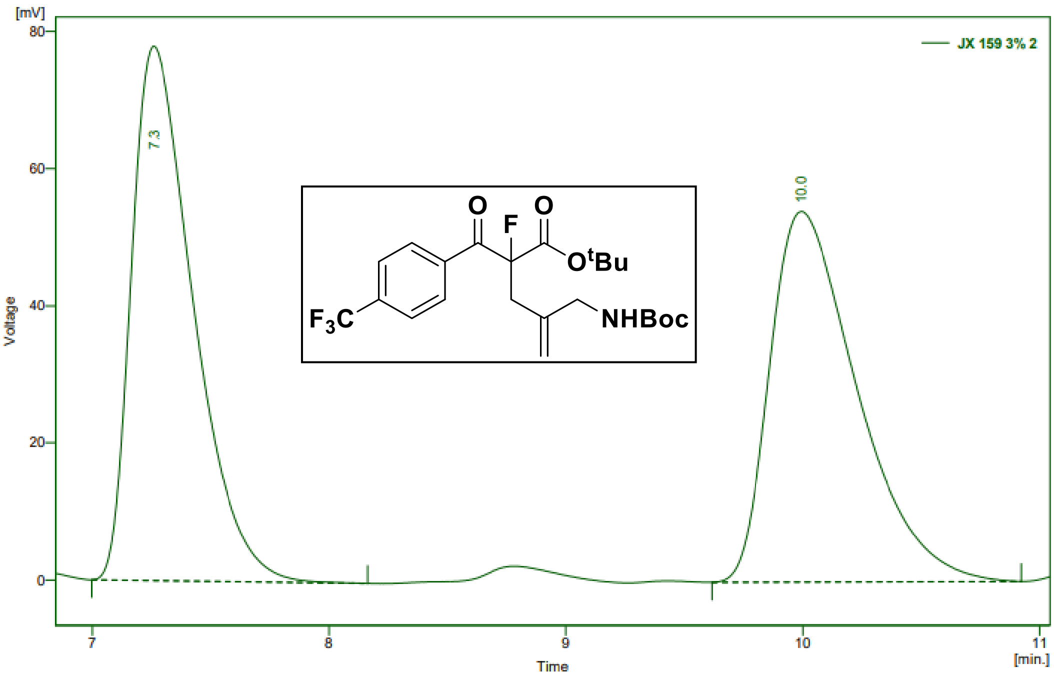

Result Table - Calculation Method Uncal

	Reten. Time [min]	Area [mV.s]	Height [mV]	Area [%]	Height [%]	W05 [min]
1	7.260	1408.234	77.951	50.5	59.1	0.28
2	9.993	1380.580	54.030	49.5	40.9	0.40
	Total	2788.813	131.980	100.0	100.0	

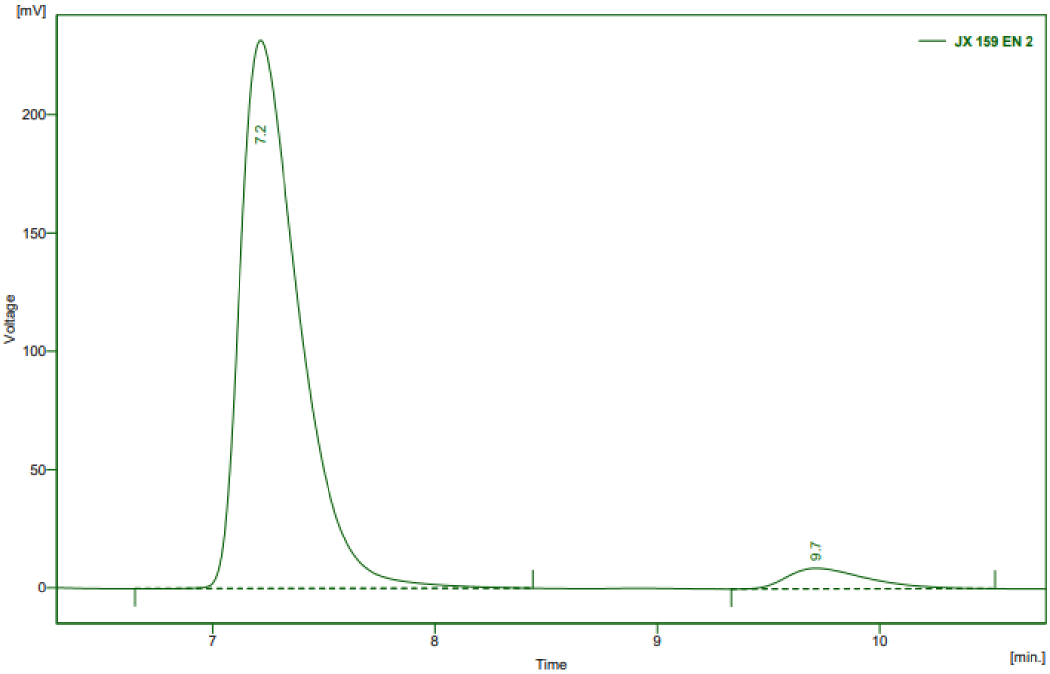

Result Table - Calculation Method Uncal

	Reten. Time [min]	Area [mV.s]	Height [mV]	Area [%]	Height [%]	W05 [min]
1	7.217	4237.319	231.637	95.2	96.4	0.28
2	9.713	215.174	8.645	4.8	3.6	0.39
	Total	4452.493	240.282	100.0	100.0	

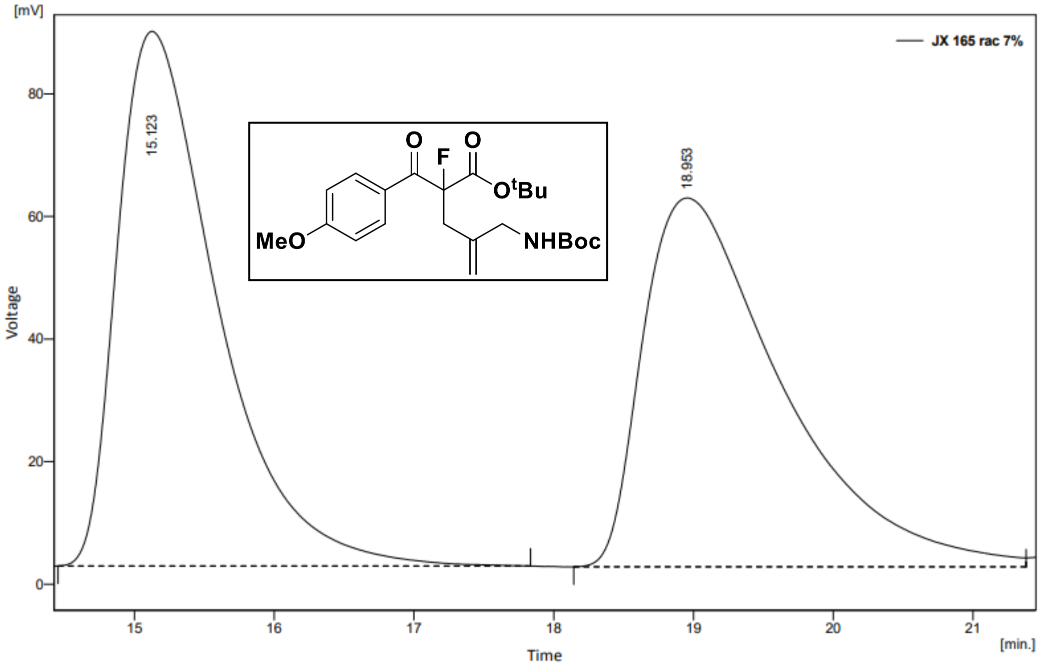

Result Table - Calculation Method Uncal

	Reten. Time [min]	Area [mV.s]	Height [mV]	Area [%]	Height [%]	W05 [min]
1	15.123	4337.351	87.223	50.9	59.2	0.74
2	18.953	4178.458	60.180	49.1	40.8	1.04
	Total	8515.810	147.403	100.0	100.0	

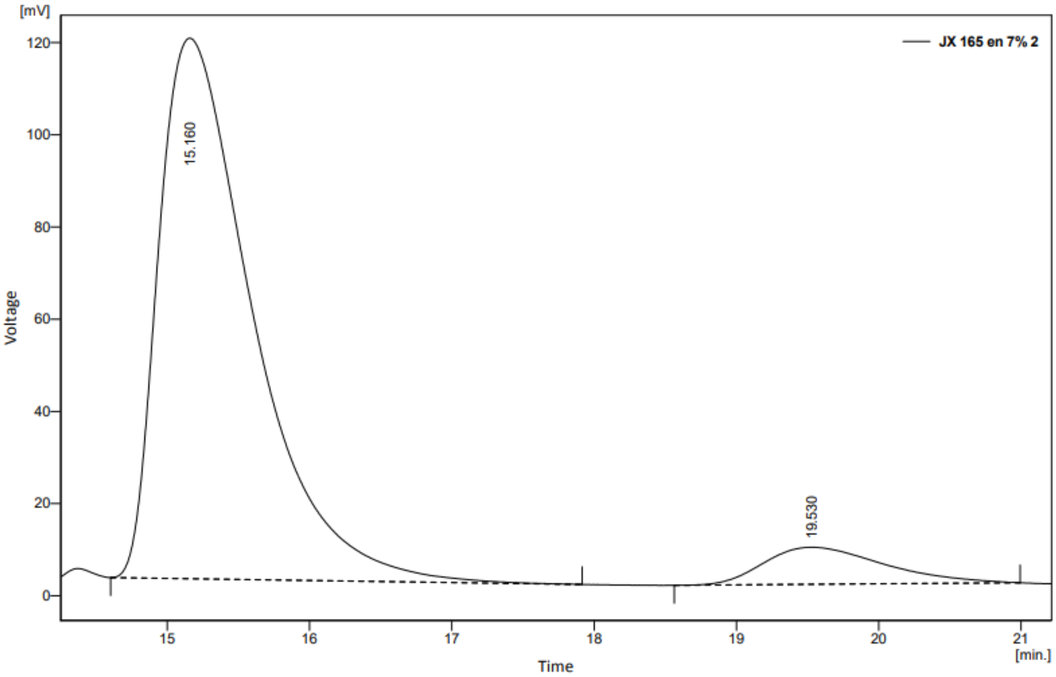

Result Table - Calculation Method Uncal

	Reten. Time [min]	Area [mV.s]	Height [mV]	Area [%]	Height [%]	W05 [min]
1	15.160	5392.299	117.261	92.0	93.6	0.69
2	19.530	471.202	8.045	8.0	6.4	0.92
	Total	5863.501	125.306	100.0	100.0	

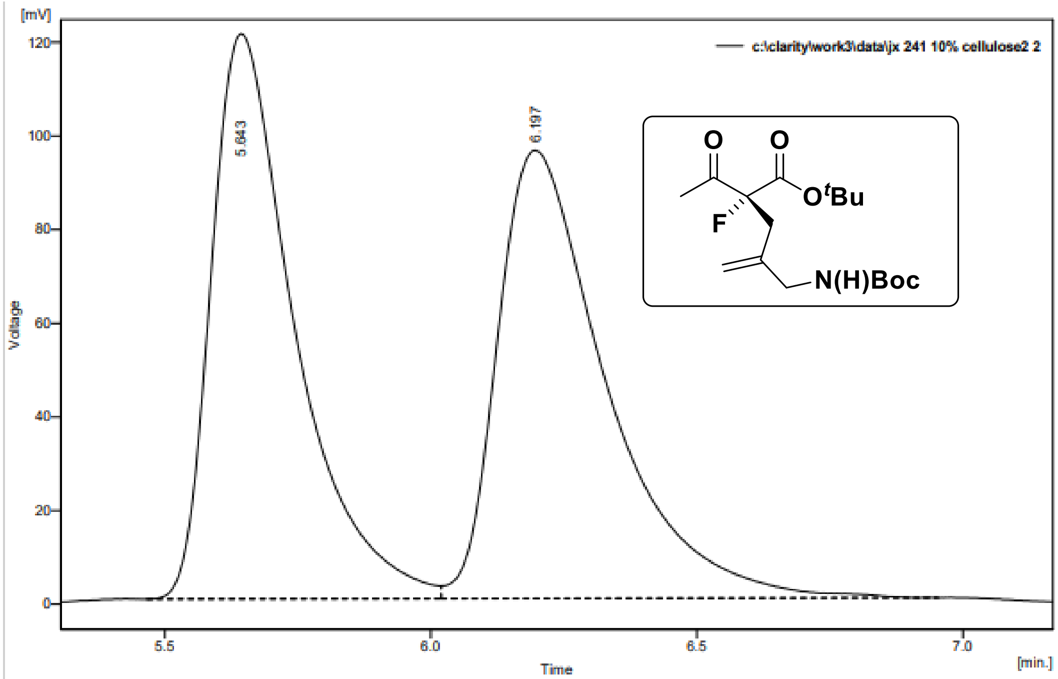

Result Table - Calculation Method Uncal

	Reten. Time [min]	Area [mV.s]	Height [mV]	Area [%]	Height [%]	W05 [min]
1	5.643	1647.220	150.948	49.2	55.8	0.16
2	6.197	1701.172	119.693	50.8	44.2	0.21
	Total	3348.392	270.641	100.0	100.0	

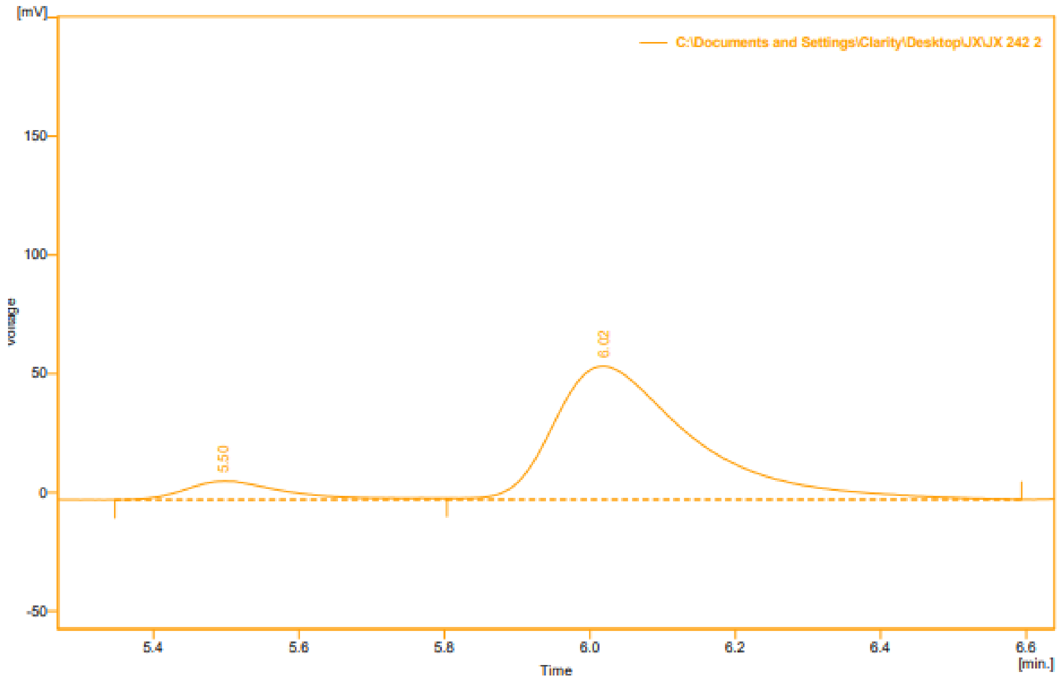

Result Table - Calculation Method Uncal

	Reten. Time [min]	Area [mV.s]	Height [mV]	Area [%]	Height [%]	W05 [min]
1	5.497	76.009	7.863	9.3	12.3	0.14
2	6.020	742.408	56.250	90.7	87.7	0.19
	Total	818.417	64.114	100.0	100.0	

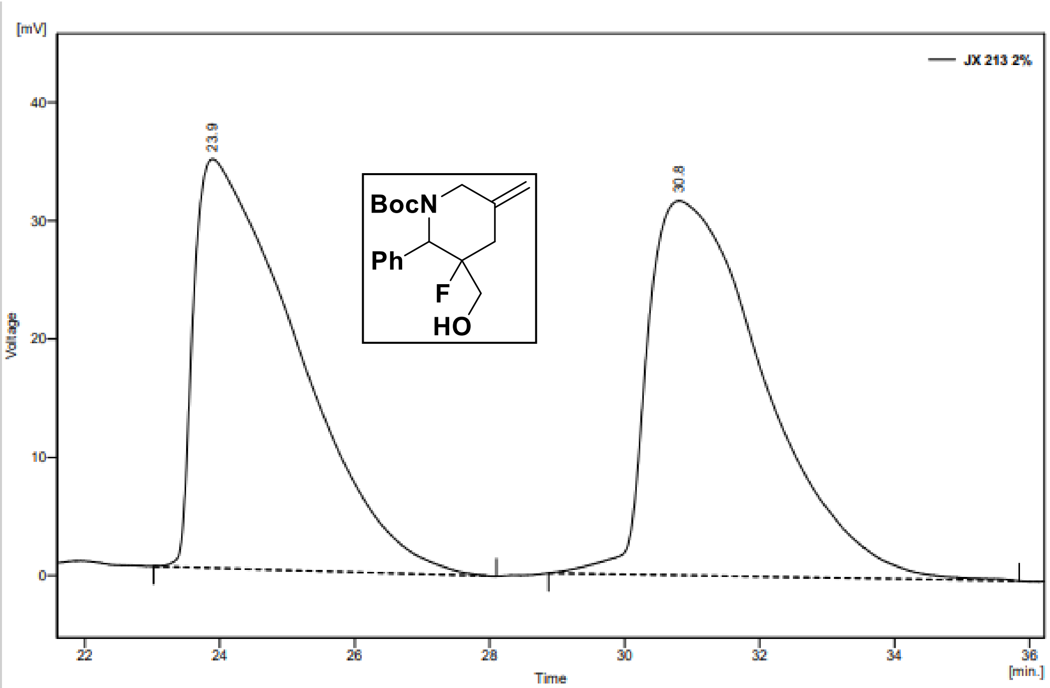

Result Table - Calculation Method Uncal

	Reten. Time [min]	Area [mV.s]	Height [mV]	Area [%]	Height [%]	W05 [min]
1	23.890	3678.455	34.598	49.1	52.2	1.68
2	30.800	3818.421	31.656	50.9	47.8	1.83
	Total	7496.876	66.255	100.0	100.0	

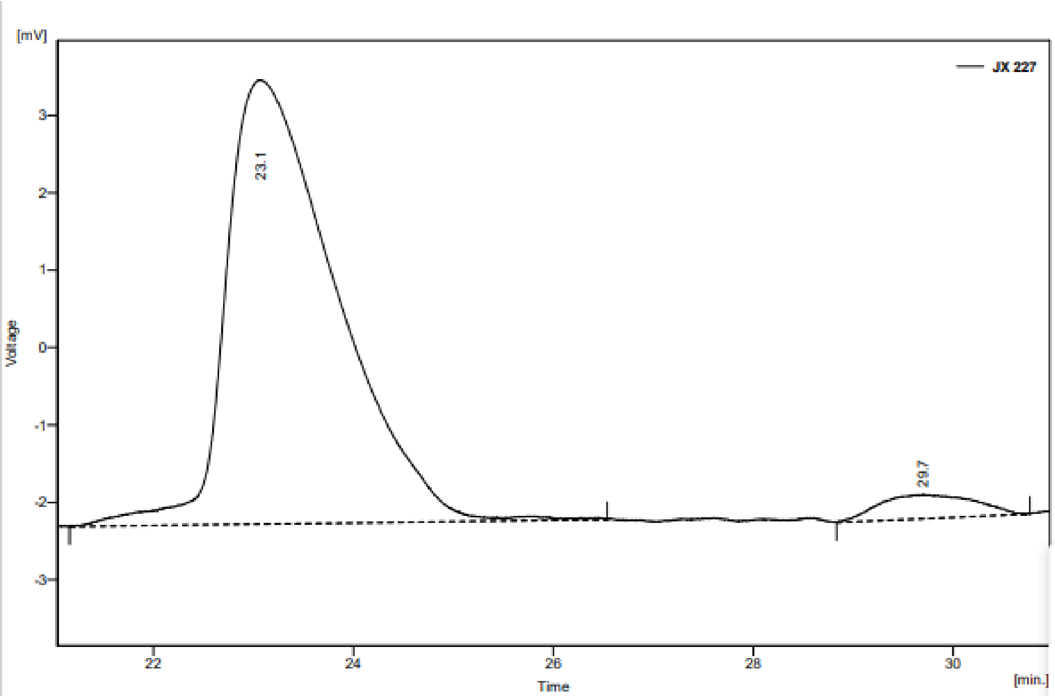

Result Table - Calculation Method Uncal

	Reten. Time [min]	Area [mV.s]	Height [mV]	Area [%]	Height [%]	W05 [min]
1	23.073	439.550	5.738	95.4	94.8	1.17
2	29.700	20.959	0.312	4.6	5.2	1.21
	Total	460.509	6.050	100.0	100.0	

<u>X-ray crystallographic analysis for compound 3b</u> (CCDC 2170247)

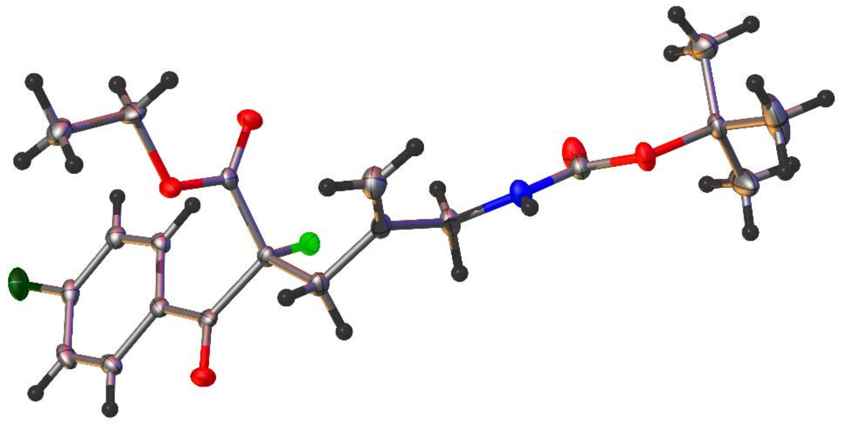

Table 1 Crystal data and structure refinement for 3b.

Identification code	OJH408s_0m
Empirical formula	$C_{20}H_{25}ClFNO_5$
Formula weight	413.86
Temperature/K	296.15
Crystal system	monoclinic
Space group	$P2_1$
a/Å	13.030(2)
b/Å	5.4670(9)
c/Å	14.728(3)
α/°	90
β/°	104.532(4)
γ/°	90
Volume/Å^3	1015.6(3)
Z	2
ρ_{calc}g/cm^3	1.353
μ/mm^{-1}	0.228
F(000)	436.0
Crystal size/mm^3	0.5 × 0.03 × 0.02
Radiation	MoKα (λ = 0.71073)
2Θ range for data collection/°	2.856 to 57.662
Index ranges	-17 ≤ h ≤ 17, -7 ≤ k ≤ 7, -19 ≤ l ≤ 19
Reflections collected	19372
Independent reflections	5281 [R_{int} = 0.0477, R_{sigma} = 0.0466]
Data/restraints/parameters	5281/1/261
Goodness-of-fit on F^2	1.029
Final R indexes [I>=2σ (I)]	R_1 = 0.0369, wR_2 = 0.0777
Final R indexes [all data]	R_1 = 0.0454, wR_2 = 0.0819
Largest diff. peak/hole / e Å^{-3}	0.32/-0.27
Flack parameter	0.04(3)

Table 2 Fractional Atomic Coordinates (×10[4]) and Equivalent Isotropic Displacement Parameters (Å[2]×10[3]) for 3b. U_{eq} is defined as 1/3 of of the trace of the orthogonalised U_{IJ} tensor.

Atom	*x*	*y*	*z*	U(eq)
Cl1	4930.1(5)	-267.6(12)	9238.8(4)	26.58(15)
F1	3464.2(10)	3792(2)	4722.9(9)	16.4(3)
O1	4703.6(13)	8414(3)	6008.9(11)	19.2(4)
O2	1527.9(12)	4477(3)	4911.2(11)	19.5(4)
O3	2017.6(12)	7970(3)	5721.5(11)	16.2(3)
O4	2259.2(16)	2405(3)	1255.8(12)	29.7(4)
O5	1774.3(14)	5590(3)	227.6(11)	20.4(4)
N1	2233.2(17)	6349(4)	1749.5(14)	19.6(4)
C1	4705(2)	1704(5)	8286.1(16)	19.0(5)
C2	5350.0(19)	3732(5)	8313.7(16)	21.3(5)
C3	5166.0(18)	5271(4)	7544.2(16)	18.6(5)
C4	4334.0(16)	4797(5)	6750.7(14)	14.8(4)
C5	3696.5(18)	2745(4)	6736.9(15)	15.9(5)
C6	3883.3(19)	1187(5)	7505.9(16)	18.2(5)
C7	4169.0(17)	6581(4)	5957.6(15)	14.4(4)
C8	3287.3(17)	6111(4)	5044.7(15)	13.3(4)
C9	3348.7(19)	8012(4)	4306.6(15)	16.7(5)
C10	2524.6(19)	7757(4)	3381.5(15)	16.8(5)
C11	2754(2)	5834(4)	2726.4(16)	19.4(5)
C12	2169.7(18)	6039(4)	5211.5(15)	13.6(4)
C13	943.7(18)	8158(5)	5862.7(18)	22.7(5)
C14	926(2)	10253(5)	6516.7(17)	22.7(5)
C15	1686(2)	9218(5)	3175.7(18)	25.0(6)
C16	2104.5(18)	4575(5)	1088.7(15)	18.5(5)
C17	1547.6(19)	4054(5)	-623.4(16)	20.1(5)
C18	657(2)	2267(6)	-618(2)	31.7(6)
C19	1162(3)	5916(6)	-1394(2)	48.1(9)
C20	2533(2)	2770(7)	-718(2)	41.0(8)

Table 3 Anisotropic Displacement Parameters (Å[2]×10[3]) for 3b. The Anisotropic displacement factor exponent takes the form: $-2\pi^2[h^2a^{*2}U_{11}+2hka^*b^*U_{12}+\ldots]$.

Atom	U_{11}	U_{22}	U_{33}	U_{23}	U_{13}	U_{12}
Cl1	35.7(3)	27.6(3)	14.5(3)	3.4(2)	2.7(2)	11.3(3)
F1	20.2(7)	11.9(6)	18.1(7)	-3.5(5)	6.7(5)	-0.3(5)
O1	16.5(8)	19.3(9)	22.1(9)	-3.3(7)	5.3(7)	-5.9(7)
O2	15.7(8)	20.8(9)	20.5(8)	-2.2(7)	1.7(6)	-4.5(7)
O3	12.1(8)	18.5(8)	18.1(8)	-4.1(7)	3.8(6)	0.9(7)
O4	47.9(12)	20.5(10)	18.7(9)	0.1(7)	4.9(8)	3.1(9)
O5	28.4(9)	19.3(9)	12.5(8)	-1.9(6)	3.2(7)	-2.0(7)
N1	25.5(11)	17.6(11)	14.1(10)	0.2(8)	2.2(8)	0.3(9)
C1	22.8(12)	20.7(12)	13.6(11)	-0.6(9)	4.5(9)	9.0(10)
C2	17.3(12)	27.7(13)	15.3(11)	-5.2(10)	-2.5(9)	6.1(10)
C3	15.5(11)	19.9(13)	19.5(11)	-6.1(9)	2.4(9)	-0.1(9)
C4	11.8(10)	17.6(10)	15.0(10)	-2.7(10)	3.4(8)	2.9(10)

Table 3 Anisotropic Displacement Parameters (Å^2×10^3) for 3b. The Anisotropic displacement factor exponent takes the form: -2π^2[h^{2}a*^{2}U$_{11}$+2hka*b*U$_{12}$+…].

Atom	U_{11}	U_{22}	U_{33}	U_{23}	U_{13}	U_{12}
C5	14.4(11)	18.0(11)	14.4(11)	-1.8(9)	2.2(8)	2.1(9)
C6	18.4(11)	18.8(12)	18.0(11)	-0.9(9)	5.6(9)	3.2(10)
C7	11.2(10)	16.9(11)	16.5(11)	-3.5(9)	5.9(8)	1.2(9)
C8	16.4(11)	10.8(10)	13.4(10)	-2.0(8)	4.8(8)	-0.8(9)
C9	21.2(12)	14.8(11)	15.2(11)	-0.5(9)	6.5(9)	-3.1(9)
C10	21.9(12)	15.7(11)	13.9(11)	1.4(9)	6.5(9)	-2.5(10)
C11	23.7(12)	20.0(12)	13.8(11)	-1.0(9)	3.2(9)	2.6(10)
C12	15.9(11)	15.1(11)	9.0(10)	1.5(8)	1.7(8)	0.9(9)
C13	12.2(11)	29.0(14)	27.9(13)	-4.8(11)	7.0(9)	1.7(10)
C14	19.7(12)	27.8(14)	22.5(12)	-1.3(10)	8.9(9)	3.4(10)
C15	29.1(14)	27.4(15)	19.1(12)	1.4(10)	7.1(10)	4.5(11)
C16	18.2(11)	21.8(12)	15.2(10)	-1.5(10)	3.6(8)	0.3(11)
C17	24.1(13)	23.4(13)	11.7(10)	-4.2(9)	2.7(9)	-2.5(10)
C18	27.3(15)	37.0(16)	32.2(15)	-11.9(12)	10.1(12)	-6.5(13)
C19	87(3)	31.6(17)	16.6(14)	-2.4(12)	-3.0(15)	-9.8(18)
C20	22.9(14)	69(2)	31.0(15)	-24.5(16)	6.2(12)	-1.3(15)

Table 4 Bond Lengths for 3b.

Atom	Atom	Length/Å	Atom	Atom	Length/Å
Cl1	C1	1.735(2)	C3	C4	1.404(3)
F1	C8	1.392(3)	C4	C5	1.393(3)
O1	C7	1.212(3)	C4	C7	1.495(3)
O2	C12	1.200(3)	C5	C6	1.389(3)
O3	C12	1.339(3)	C7	C8	1.555(3)
O3	C13	1.469(3)	C8	C9	1.521(3)
O4	C16	1.218(3)	C8	C12	1.537(3)
O5	C16	1.352(3)	C9	C10	1.515(3)
O5	C17	1.476(3)	C10	C11	1.507(3)
N1	C11	1.456(3)	C10	C15	1.326(4)
N1	C16	1.354(3)	C13	C14	1.500(3)
C1	C2	1.386(4)	C17	C18	1.518(4)
C1	C6	1.389(3)	C17	C19	1.513(4)
C2	C3	1.383(3)	C17	C20	1.500(4)

Table 5 Bond Angles for 3b.

Atom	Atom	Atom	Angle/˚	Atom	Atom	Atom	Angle/˚
C12	O3	C13	114.52(18)	C9	C8	C12	110.70(18)
C16	O5	C17	120.78(19)	C12	C8	C7	112.95(17)
C16	N1	C11	121.0(2)	C10	C9	C8	115.31(19)
C2	C1	Cl1	119.68(18)	C11	C10	C9	115.5(2)
C2	C1	C6	121.5(2)	C15	C10	C9	120.5(2)
C6	C1	Cl1	118.8(2)	C15	C10	C11	124.0(2)

Table 5 Bond Angles for 3b.

Atom	Atom	Atom	Angle/˚	Atom	Atom	Atom	Angle/˚
C3	C2	C1	118.9(2)	N1	C11	C10	112.3(2)
C2	C3	C4	120.6(2)	O2	C12	O3	125.5(2)
C3	C4	C7	117.3(2)	O2	C12	C8	124.0(2)
C5	C4	C3	119.6(2)	O3	C12	C8	110.44(18)
C5	C4	C7	123.06(19)	O3	C13	C14	108.2(2)
C6	C5	C4	120.0(2)	O4	C16	O5	125.9(2)
C5	C6	C1	119.4(2)	O4	C16	N1	124.6(2)
O1	C7	C4	121.5(2)	O5	C16	N1	109.5(2)
O1	C7	C8	118.6(2)	O5	C17	C18	110.60(19)
C4	C7	C8	119.86(19)	O5	C17	C19	102.3(2)
F1	C8	C7	107.17(17)	O5	C17	C20	110.6(2)
F1	C8	C9	109.63(16)	C19	C17	C18	109.1(2)
F1	C8	C12	106.01(17)	C20	C17	C18	111.9(2)
C9	C8	C7	110.19(18)	C20	C17	C19	112.1(3)

Table 6 Hydrogen Bonds for 3b.

D	H	A	d(D-H)/Å	d(H-A)/Å	d(D-A)/Å	D-H-A/°
N1	H1	O4[1]	0.84(3)	2.56(3)	3.392(3)	173(3)
C9	H9A	F1[1]	0.97	2.30	3.215(3)	157.2
C13	H13B	O2[2]	0.97	2.57	3.217(3)	124.4
C14	H14C	O2[2]	0.96	2.80	3.384(3)	120.4

[1]+X,1+Y,+Z; [2]-X,1/2+Y,1-Z

Table 7 Torsion Angles for 3b.

A	B	C	D	Angle/˚	A	B	C	D	Angle/˚
Cl1	C1	C2	C3	179.36(18)	C7	C4	C5	C6	-178.8(2)
Cl1	C1	C6	C5	-179.76(17)	C7	C8	C9	C10	-179.77(19)
F1	C8	C9	C10	62.5(2)	C7	C8	C12	O2	-132.3(2)
F1	C8	C12	O2	-15.2(3)	C7	C8	C12	O3	48.6(2)
F1	C8	C12	O3	165.69(16)	C8	C9	C10	C11	-79.5(2)
O1	C7	C8	F1	125.8(2)	C8	C9	C10	C15	102.6(3)
O1	C7	C8	C9	6.6(3)	C9	C8	C12	O2	103.6(3)
O1	C7	C8	C12	-117.8(2)	C9	C8	C12	O3	-75.5(2)
C1	C2	C3	C4	0.5(3)	C9	C10	C11	N1	-155.1(2)
C2	C1	C6	C5	-0.5(3)	C11	N1	C16	O4	11.0(4)
C2	C3	C4	C5	-0.6(3)	C11	N1	C16	O5	-169.4(2)
C2	C3	C4	C7	178.5(2)	C12	O3	C13	C14	174.71(18)
C3	C4	C5	C6	0.2(3)	C12	C8	C9	C10	-54.1(3)
C3	C4	C7	O1	-2.8(3)	C13	O3	C12	O2	-3.2(3)
C3	C4	C7	C8	177.80(19)	C13	O3	C12	C8	175.91(19)
C4	C5	C6	C1	0.3(3)	C15	C10	C11	N1	22.7(3)
C4	C7	C8	F1	-54.7(2)	C16	O5	C17	C18	61.3(3)

Table 7 Torsion Angles for 3b.

A	B	C	D	Angle/˚	A	B	C	D	Angle/˚
C4	C7	C8	C9	-173.96(18)	C16	O5	C17	C19	177.4(2)
C4	C7	C8	C12	61.6(3)	C16	O5	C17	C20	-63.2(3)
C5	C4	C7	O1	176.3(2)	C16	N1	C11	C10	-162.3(2)
C5	C4	C7	C8	-3.1(3)	C17	O5	C16	O4	1.0(4)
C6	C1	C2	C3	0.1(3)	C17	O5	C16	N1	-178.57(19)

Table 8 Hydrogen Atom Coordinates (Å×10^4) and Isotropic Displacement Parameters (Å^2×10^3) for 3b.

Atom	*x*	*y*	*z*	U(eq)
H1	2190(20)	7820(60)	1582(19)	24(8)
H2	5897.71	4052.91	8840.96	26
H3	5596.74	6631.89	7552.49	22
H5	3145.81	2417.38	6212.66	19
H6	3461.95	-189.69	7498.57	22
H9A	3277.67	9621.25	4561.28	20
H9B	4046.4	7923.52	4186.38	20
H11A	3514.13	5741.91	2798.18	23
H11B	2518.32	4256.02	2896.83	23
H13A	756.59	6650.32	6129.36	27
H13B	434.34	8437.75	5267.26	27
H14A	1153.86	11717.85	6265.3	34
H14B	1395.2	9907.33	7117.99	34
H14C	218.72	10473.97	6585.4	34
H15A	1197.13	9090.03	2597.03	30
H15B	1587.28	10371.72	3610.02	30
H18A	917.09	986.69	-173.5	48
H18B	402.06	1573.06	-1231.79	48
H18C	88.89	3112.07	-444.16	48
H19A	554.86	6755.09	-1290.18	72
H19B	971.48	5098.03	-1989.74	72
H19C	1716.64	7074.81	-1391.5	72
H20A	3086.13	3948.2	-691.48	62
H20B	2390.31	1923.77	-1307.68	62
H20C	2753.53	1615.76	-215.55	62

Crystal structure determination of 3b

Crystal Data for $C_{20}H_{25}ClFNO_5$ (*M* =413.86 g/mol): monoclinic, space group $P2_1$ (no. 4), a = 13.030(2) Å, b = 5.4670(9) Å, c = 14.728(3) Å, β = 104.532(4)°, V = 1015.6(3) Å^3, Z = 2, T = 296.15 K, μ(MoKα) = 0.228 mm^{-1}, *Dcalc* = 1.353 g/cm^3, 19372 reflections measured (2.856° ≤ 2Θ ≤ 57.662°), 5281 unique (R_{int} = 0.0477, R_{sigma} = 0.0466) which were used in all calculations. The final R_1 was 0.0369 (I > 2σ(I)) and wR_2 was 0.0819 (all data).

X-ray crystallographic analysis for compound 3f (CCDC 2170248)

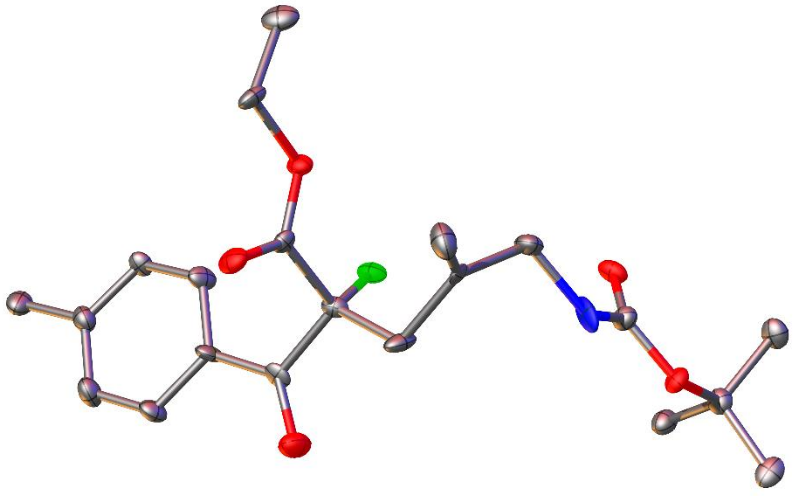

Table 1 Crystal data and structure refinement for 3f.

Identification code	OJH409v_0m
Empirical formula	$C_{21}H_{28}FNO_5$
Formula weight	393.44
Temperature/K	100.02
Crystal system	monoclinic
Space group	$P2_1$
a/Å	5.4499(6)
b/Å	8.2741(9)
c/Å	23.173(3)
α/°	90
β/°	93.304(5)
γ/°	90
Volume/Å^3	1043.2(2)
Z	2
ρ_{calc}g/cm^3	1.253
μ/mm^{-1}	0.786
F(000)	420.0
Crystal size/mm^3	0.179 × 0.082 × 0.053
Radiation	CuKα (λ = 1.54178)
2Θ range for data collection/°	3.82 to 133.504
Index ranges	-6 ≤ h ≤ 5, -9 ≤ k ≤ 9, -27 ≤ l ≤ 27
Reflections collected	18900
Independent reflections	3657 [R_{int} = 0.0541, R_{sigma} = 0.0362]
Data/restraints/parameters	3657/205/258
Goodness-of-fit on F^2	1.160
Final R indexes [I>=2σ (I)]	R_1 = 0.1442, wR_2 = 0.3843
Final R indexes [all data]	R_1 = 0.1463, wR_2 = 0.3850
Largest diff. peak/hole / e Å^{-3}	1.65/-0.82
Flack parameter	0.14(7)

Table 2 Fractional Atomic Coordinates (×10^4) and Equivalent Isotropic Displacement Parameters (Å^2×10^3) for 3f. U_{eq} is defined as 1/3 of of the trace of the orthogonalised U_{IJ} tensor.

Atom	*x*	*y*	*z*	U(eq)
F1	2588(15)	5050(10)	2771(4)	18.2(19)
O1	6900(20)	3131(15)	3506(5)	26(3)
O2	7150(20)	7509(13)	3453(5)	21(2)
O3	3560(20)	8049(12)	2971(5)	17(2)
O4	1132(19)	3412(13)	1110(5)	19(2)
O5	4302(19)	1843(12)	805(4)	16(2)
N1	5200(30)	4076(18)	1271(5)	24(3)
C1	1460(30)	4990(20)	5055(7)	21(3)
C2	3580(30)	4030(20)	5063(7)	25(3)
C3	4810(30)	3750(20)	4564(7)	20(3)
C4	4070(30)	4530(19)	4052(7)	18(3)
C5	1950(30)	5520(20)	4039(7)	20(3)
C6	650(30)	5707(19)	4535(6)	17(3)
C7	110(30)	5230(20)	5603(7)	24(3)
C8	5410(30)	4211(19)	3538(6)	15(3)
C9	5010(30)	5316(18)	2992(6)	14(3)
C10	6780(30)	4893(19)	2542(7)	21(3)
C11	6560(30)	6022(18)	2010(6)	14(3)
C12	4660(30)	5659(19)	1540(7)	21(3)
C13	5370(30)	7085(17)	3168(6)	13(3)
C14	3900(30)	9768(18)	3120(7)	18(3)
C15	1570(30)	10610(20)	2862(8)	26(4)
C16	7980(30)	7260(20)	1981(7)	27(4)
C17	3350(30)	3120(19)	1066(7)	17(3)
C18	2730(30)	515(19)	572(6)	18(3)
C19	4600(30)	-603(19)	327(7)	22(3)
C20	1030(30)	1170(20)	75(7)	23(3)
C21	1420(30)	-270(20)	1041(7)	22(3)

Table 3 Anisotropic Displacement Parameters (Å^2×10^3) for 3f. The Anisotropic displacement factor exponent takes the form: $-2\pi^2[h^2a^{*2}U_{11}+2hka^*b^*U_{12}+\ldots]$.

Atom	U_{11}	U_{22}	U_{33}	U_{23}	U_{13}	U_{12}
F1	15(4)	13(4)	26(4)	3(4)	-4(3)	-1(3)
O1	25(6)	26(6)	29(6)	3(5)	5(5)	8(5)
O2	20(5)	13(5)	29(6)	-6(4)	-3(4)	-2(4)
O3	19(5)	10(4)	21(5)	0(4)	-3(4)	-1(4)
O4	14(4)	17(6)	24(5)	4(4)	-8(4)	-4(4)
O5	16(5)	13(5)	20(5)	-6(4)	2(4)	-4(4)
N1	33(7)	24(6)	14(6)	0(5)	-12(5)	-16(6)
C1	24(7)	22(8)	17(6)	1(6)	-3(5)	-2(6)
C2	25(8)	34(9)	16(6)	3(6)	-1(5)	1(6)
C3	22(8)	21(8)	18(6)	9(6)	1(5)	3(6)

Table 3 Anisotropic Displacement Parameters (Å^2×10^3) for 3f. The Anisotropic displacement factor exponent takes the form: -2π^2[h^{2}a*^{2}U$_{11}$+2hka*b*U$_{12}$+…].

Atom	U$_{11}$	U$_{22}$	U$_{33}$	U$_{23}$	U$_{13}$	U$_{12}$
C4	19(7)	18(8)	19(6)	9(5)	1(5)	7(6)
C5	21(7)	22(8)	18(6)	8(6)	7(5)	3(6)
C6	10(6)	22(8)	18(6)	2(5)	-1(5)	-2(6)
C7	34(9)	18(9)	21(7)	4(6)	3(6)	1(7)
C8	6(6)	20(7)	18(6)	3(5)	-5(4)	-5(5)
C9	10(6)	13(6)	20(6)	3(5)	-1(4)	-2(5)
C10	25(8)	10(7)	28(7)	4(6)	12(6)	6(6)
C11	19(7)	13(6)	11(6)	-4(5)	4(5)	6(5)
C12	21(7)	18(7)	23(7)	2(6)	-4(6)	6(6)
C13	11(6)	12(6)	15(6)	-2(5)	5(4)	-1(4)
C14	21(7)	10(6)	23(7)	-3(6)	9(6)	2(5)
C15	23(8)	14(8)	42(9)	1(7)	1(7)	-4(6)
C16	34(9)	29(8)	17(8)	1(6)	-2(7)	-9(7)
C17	17(5)	15(6)	20(7)	-3(5)	-1(5)	-2(5)
C18	19(7)	16(7)	20(7)	-1(5)	0(5)	-5(5)
C19	23(8)	15(7)	29(8)	-4(6)	8(6)	-9(6)
C20	17(7)	29(9)	22(8)	-1(6)	-4(6)	-5(6)
C21	26(8)	17(8)	23(7)	0(6)	5(6)	0(6)

Table 4 Bond Lengths for 3f.

Atom	Atom	Length/Å	Atom	Atom	Length/Å
F1	C9	1.406(16)	C3	C4	1.39(2)
O1	C8	1.213(19)	C4	C5	1.41(2)
O2	C13	1.196(19)	C4	C8	1.46(2)
O3	C13	1.327(18)	C5	C6	1.40(2)
O3	C14	1.473(18)	C8	C9	1.566(19)
O4	C17	1.243(19)	C9	C10	1.50(2)
O5	C17	1.337(19)	C9	C13	1.53(2)
O5	C18	1.477(18)	C10	C11	1.55(2)
N1	C12	1.49(2)	C11	C12	1.49(2)
N1	C17	1.35(2)	C11	C16	1.29(2)
C1	C2	1.40(2)	C14	C15	1.54(2)
C1	C6	1.39(2)	C18	C19	1.51(2)
C1	C7	1.52(2)	C18	C20	1.53(2)
C2	C3	1.39(2)	C18	C21	1.48(2)

Table 5 Bond Angles for 3f.

Atom	Atom	Atom	Angle/˚	Atom	Atom	Atom	Angle/˚
C13	O3	C14	114.8(12)	C10	C9	C13	109.3(12)
C17	O5	C18	121.5(12)	C13	C9	C8	109.5(12)
C17	N1	C12	120.2(14)	C9	C10	C11	112.8(12)
C2	C1	C7	120.1(14)	C12	C11	C10	118.7(14)

Table 5 Bond Angles for 3f.

Atom	Atom	Atom	Angle/˚	Atom	Atom	Atom	Angle/˚
C6	C1	C2	118.3(15)	C16	C11	C10	120.2(15)
C6	C1	C7	121.6(15)	C16	C11	C12	121.1(15)
C3	C2	C1	121.2(15)	N1	C12	C11	109.7(12)
C4	C3	C2	120.3(15)	O2	C13	O3	125.2(13)
C3	C4	C5	119.1(14)	O2	C13	C9	121.0(13)
C3	C4	C8	118.6(13)	O3	C13	C9	113.8(12)
C5	C4	C8	122.2(13)	O3	C14	C15	104.8(13)
C6	C5	C4	119.9(14)	O4	C17	O5	126.5(14)
C1	C6	C5	121.0(15)	O4	C17	N1	124.7(15)
O1	C8	C4	123.5(14)	O5	C17	N1	108.8(13)
O1	C8	C9	116.3(13)	O5	C18	C19	101.8(12)
C4	C8	C9	120.2(13)	O5	C18	C20	108.9(13)
F1	C9	C8	106.7(11)	O5	C18	C21	110.4(12)
F1	C9	C10	109.8(12)	C19	C18	C20	109.0(13)
F1	C9	C13	110.5(11)	C21	C18	C19	111.8(13)
C10	C9	C8	111.1(12)	C21	C18	C20	114.2(14)

Table 6 Torsion Angles for 3f.

A	B	C	D	Angle/˚	A	B	C	D	Angle/˚
F1	C9	C10	C11	65.7(16)	C8	C4	C5	C6	175.9(15)
F1	C9	C13	O2	167.4(13)	C8	C9	C10	C11	-176.6(12)
F1	C9	C13	O3	-13.2(16)	C8	C9	C13	O2	50.2(18)
O1	C8	C9	F1	114.3(14)	C8	C9	C13	O3	-130.4(12)
O1	C8	C9	C10	-5.3(19)	C9	C10	C11	C12	-84.3(17)
O1	C8	C9	C13	-126.1(14)	C9	C10	C11	C16	94.8(18)
C1	C2	C3	C4	5(3)	C10	C9	C13	O2	-71.8(18)
C2	C1	C6	C5	-3(2)	C10	C9	C13	O3	107.6(14)
C2	C3	C4	C5	-4(2)	C10	C11	C12	N1	-63.5(17)
C2	C3	C4	C8	179.9(16)	C12	N1	C17	O4	-4(2)
C3	C4	C5	C6	0(2)	C12	N1	C17	O5	174.9(12)
C3	C4	C8	O1	11(2)	C13	O3	C14	C15	-178.5(12)
C3	C4	C8	C9	-167.1(14)	C13	C9	C10	C11	-55.6(17)
C4	C5	C6	C1	3(2)	C14	O3	C13	O2	1(2)
C4	C8	C9	F1	-67.1(16)	C14	O3	C13	C9	-178.3(12)
C4	C8	C9	C10	173.3(13)	C16	C11	C12	N1	117.4(17)
C4	C8	C9	C13	52.4(17)	C17	O5	C18	C19	179.9(13)
C5	C4	C8	O1	-164.7(15)	C17	O5	C18	C20	64.9(17)
C5	C4	C8	C9	17(2)	C17	O5	C18	C21	-61.2(18)
C6	C1	C2	C3	-2(3)	C17	N1	C12	C11	147.0(14)
C7	C1	C2	C3	178.0(16)	C18	O5	C17	O4	-5(2)
C7	C1	C6	C5	177.9(15)	C18	O5	C17	N1	175.6(12)

Table 7 Hydrogen Atom Coordinates (Å×10[4]) and Isotropic Displacement Parameters (Å[2]×10[3]) for 3f.

Atom	*x*	*y*	*z*	U(eq)
H1	6732.01	3763.5	1245.78	29
H2	4183.63	3553.82	5416.88	30
H3	6165.09	3029.67	4572.36	24
H5	1426.82	6067.37	3692.26	24
H6	-822.37	6328.56	4517.95	20
H7A	1114.67	5882.08	5877.39	36
H7B	-210.76	4171.62	5775.22	36
H7C	-1456.69	5778.82	5509.25	36
H10A	6481.93	3765.4	2413.69	25
H10B	8480.27	4952.96	2717.65	25
H12A	3013.25	5623.14	1701.02	25
H12B	4644.35	6521.16	1243.97	25
H14A	5385.73	10207.35	2950.58	22
H14B	4057.82	9914.91	3544.91	22
H15A	1416.58	10411.12	2443.9	39
H15B	1686.03	11770.81	2934.63	39
H15C	127.21	10170.06	3041.15	39
H16A	7817.96	7961.8	1656.81	32
H16B	9178.75	7473.98	2285	32
H19A	5544.27	-10.84	48.56	33
H19B	3748.48	-1512.72	131.84	33
H19C	5710.21	-1010.46	640.75	33
H20A	-310.84	1787.35	233.9	35
H20B	342.67	265.72	-154.99	35
H20C	1967.39	1874.51	-170.92	35
H21A	2592.31	-489.89	1368.28	33
H21B	686.32	-1281.12	898.76	33
H21C	132.11	457.05	1164.79	33

Crystal structure determination of 3f

Crystal Data for $C_{21}H_{28}FNO_5$ (M =393.44 g/mol): monoclinic, space group P2$_1$ (no. 4), a = 5.4499(6) Å, b = 8.2741(9) Å, c = 23.173(3) Å, β = 93.304(5)°, V = 1043.2(2) Å^3, Z = 2, T = 100.02 K, μ(CuKα) = 0.786 mm^{-1}, $Dcalc$ = 1.253 g/cm^3, 18900 reflections measured (3.82° ≤ 2Θ ≤ 133.504°), 3657 unique (R_{int} = 0.0541, R_{sigma} = 0.0362) which were used in all calculations. The final R_1 was 0.1442 (I > 2σ(I)) and wR_2 was 0.3850 (all data).

X-ray crystallographic analysis for compound 3i (CCDC 2170249)

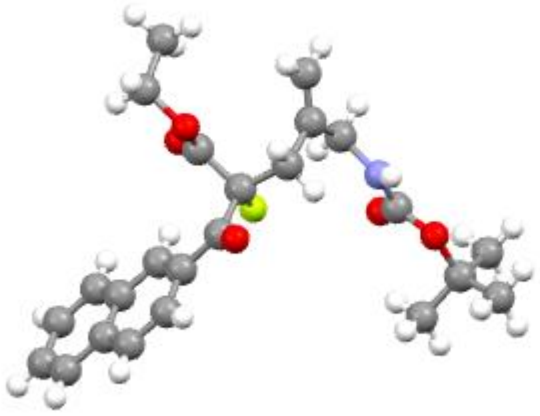

Table 1 Crystal data and structure refinement for 3i.

Identification code	OJH410v_2_0m
Empirical formula	$C_{24}H_{28}FNO_5$
Formula weight	429.47
Temperature/K	100.00
Crystal system	monoclinic
Space group	$P2_1$
a/Å	6.84040(10)
b/Å	10.0817(2)
c/Å	15.9492(3)
α/°	90
β/°	99.4250(10)
γ/°	90
Volume/Å^3	1085.05(3)
Z	2
ρ_{calc}g/cm^3	1.315
μ/mm^{-1}	0.805
F(000)	456.0
Crystal size/mm^3	0.17 × 0.11 × 0.038
Radiation	CuKα (λ = 1.54178)
2Θ range for data collection/°	5.616 to 133.254
Index ranges	-8 ≤ h ≤ 8, -12 ≤ k ≤ 12, -18 ≤ l ≤ 18
Reflections collected	36222
Independent reflections	3825 [R_{int} = 0.0374, R_{sigma} = 0.0171]
Data/restraints/parameters	3825/1/284
Goodness-of-fit on F^2	1.082
Final R indexes [I>=2σ (I)]	R_1 = 0.0250, wR_2 = 0.0615
Final R indexes [all data]	R_1 = 0.0268, wR_2 = 0.0627
Largest diff. peak/hole / e Å^{-3}	0.14/-0.21
Flack parameter	0.02(4)

Table 2 Fractional Atomic Coordinates (×10⁴) and Equivalent Isotropic Displacement Parameters (Å²×10³) for 3i. U_{eq} is defined as 1/3 of the trace of the orthogonalised U_{IJ} tensor.

Atom	*x*	*y*	*z*	U(eq)
F1	3412.4(15)	4450.6(10)	7246.1(6)	17.5(2)
O1	5632(2)	4640.7(14)	9292.5(8)	22.4(3)
O2	746.6(19)	6284.8(13)	7420.5(9)	21.7(3)
O3	2748.2(19)	7215.6(13)	8522.3(8)	18.6(3)
O4	5898(2)	3578.0(14)	5315.5(9)	23.1(3)
O5	9117(2)	3326.8(13)	5976.4(8)	20.0(3)
N1	7424(2)	5066.7(16)	6280.0(10)	16.8(3)
C1	2600(3)	3515.7(19)	8878.6(12)	15.0(4)
C2	830(3)	3392.6(18)	8326.6(11)	14.8(4)
C3	-670(3)	2522.4(18)	8510.4(12)	15.0(4)
C4	-2495(3)	2379.2(19)	7953.3(12)	17.1(4)
C5	-3912(3)	1520(2)	8142.0(12)	19.9(4)
C6	-3569(3)	762(2)	8899.8(13)	22.0(4)
C7	-1817(3)	883(2)	9444.7(12)	20.8(4)
C8	-320(3)	1756.1(18)	9269.0(12)	16.5(4)
C9	1525(3)	1868.4(19)	9810.2(12)	17.8(4)
C10	2947(3)	2717.5(19)	9627.1(12)	17.3(4)
C11	4189(3)	4473.5(18)	8752.8(11)	15.7(4)
C12	3968(3)	5313.1(19)	7924.9(11)	14.4(4)
C13	5903(3)	6003.8(19)	7824.7(11)	15.4(4)
C14	5794(3)	6777.1(19)	7002.2(12)	16.0(4)
C15	5789(3)	6011(2)	6187.9(12)	17.0(4)
C16	2283(3)	6324.0(19)	7910.1(11)	15.4(4)
C17	1225(3)	8223(2)	8583.9(13)	22.5(4)
C18	1133(3)	9246(2)	7890.3(13)	24.7(4)
C19	5740(3)	8091(2)	6997.4(14)	22.6(4)
C20	7340(3)	3955.4(19)	5803.3(11)	15.7(4)
C21	9443(3)	2053(2)	5567.0(13)	20.7(4)
C22	11581(3)	1731(2)	5946.9(15)	30.5(5)
C23	8041(3)	1005(2)	5809.1(13)	25.8(5)
C24	9234(3)	2244(2)	4609.1(13)	25.7(5)

Table 3 Anisotropic Displacement Parameters (Å²×10³) for 3i. The Anisotropic displacement factor exponent takes the form: $-2\pi^2[h^2a^{*2}U_{11}+2hka^*b^*U_{12}+\ldots]$.

Atom	U_{11}	U_{22}	U_{33}	U_{23}	U_{13}	U_{12}
F1	21.1(6)	17.0(5)	14.2(5)	-4.2(4)	2.2(4)	-4.5(4)
O1	22.3(7)	24.8(8)	17.7(7)	0.6(6)	-3.4(6)	-4.5(6)
O2	17.3(7)	18.8(7)	26.2(7)	1.7(6)	-4.9(6)	-1.9(5)
O3	19.7(7)	18.6(7)	17.5(6)	-2.9(6)	2.6(5)	3.4(6)
O4	19.9(7)	23.6(7)	24.0(7)	-7.1(6)	-1.6(6)	0.5(6)

Table 3 Anisotropic Displacement Parameters ($Å^2×10^3$) for 3i. The Anisotropic displacement factor exponent takes the form: $-2\pi^2[h^2a^{*2}U_{11}+2hka^*b^*U_{12}+…]$.

Atom	U_{11}	U_{22}	U_{33}	U_{23}	U_{13}	U_{12}
O5	18.6(7)	19.0(7)	21.5(7)	-6.1(6)	0.4(5)	2.6(5)
N1	14.1(8)	18.5(8)	17.0(8)	-3.7(6)	0.1(6)	-0.4(6)
C1	17.7(9)	13.1(9)	14.9(9)	-1.4(7)	4.4(7)	1.5(7)
C2	18.7(9)	12.7(9)	13.4(8)	1.6(7)	3.7(7)	2.5(7)
C3	19.4(9)	11.8(9)	14.7(8)	-1.3(7)	5.1(7)	3.2(7)
C4	19.9(9)	15.0(9)	16.6(9)	0.1(7)	3.5(7)	2.2(8)
C5	18.2(10)	18.2(10)	23.2(10)	-2.5(8)	3.1(8)	-1.1(8)
C6	22.0(10)	20.5(10)	25.5(10)	1.2(8)	10.0(8)	-3.5(8)
C7	26.1(10)	18.4(10)	19.9(10)	1.9(8)	9.5(8)	-0.8(8)
C8	22.5(10)	13.1(9)	15.6(9)	-0.5(7)	7.9(7)	2.9(7)
C9	23.2(10)	17.7(9)	12.8(9)	2.0(7)	4.1(7)	4.5(8)
C10	18.1(9)	18.4(10)	15.0(9)	0.4(8)	1.1(7)	3.9(8)
C11	16.9(9)	14.6(9)	15.6(9)	-2.8(7)	2.6(7)	0.3(8)
C12	15.7(9)	14.7(9)	12.4(9)	-4.0(7)	1.0(7)	-2.8(7)
C13	14.0(9)	17.4(9)	14.8(9)	-2.4(8)	2.5(7)	-1.0(7)
C14	10.5(9)	19.0(10)	18.6(9)	-0.5(8)	2.6(7)	-1.4(7)
C15	17.8(9)	16.8(9)	16.5(9)	1.0(8)	3.0(7)	1.6(8)
C16	17.8(10)	15.0(9)	14.0(8)	2.6(7)	4.2(7)	-3.8(7)
C17	23.4(10)	21.3(11)	23.9(10)	-3.0(9)	7.6(8)	7.2(8)
C18	24.7(11)	18.8(10)	30.7(11)	-0.9(9)	4.7(8)	2.9(8)
C19	22.5(11)	19.6(11)	26.7(10)	-0.3(8)	7.2(8)	-1.7(8)
C20	17.4(9)	16.4(9)	13.7(8)	0.4(7)	3.6(7)	-0.8(8)
C21	22.1(10)	17.7(10)	22.2(10)	-6.5(8)	3.0(8)	4.1(8)
C22	26.2(11)	31.0(12)	33.0(12)	-7.6(10)	1.4(9)	8.4(9)
C23	33.3(12)	19.8(10)	24.4(10)	-0.3(9)	5.4(9)	1.4(9)
C24	27.5(11)	27.4(11)	23.3(10)	-5.3(9)	7.5(9)	2.4(9)

Table 4 Bond Lengths for 3i.

Atom	Atom	Length/Å	Atom	Atom	Length/Å
F1	C12	1.392(2)	C4	C5	1.370(3)
O1	C11	1.210(2)	C5	C6	1.416(3)
O2	C16	1.203(2)	C6	C7	1.366(3)
O3	C16	1.327(2)	C7	C8	1.413(3)
O3	C17	1.470(2)	C8	C9	1.412(3)
O4	C20	1.213(2)	C9	C10	1.363(3)
O5	C20	1.358(2)	C11	C12	1.555(3)
O5	C21	1.474(2)	C12	C13	1.527(2)
N1	C15	1.458(2)	C12	C16	1.536(3)
N1	C20	1.350(2)	C13	C14	1.517(3)
C1	C2	1.381(3)	C14	C15	1.511(3)

Table 4 Bond Lengths for 3i.

Atom	Atom	Length/Å	Atom	Atom	Length/Å
C1	C10	1.427(3)	C14	C19	1.325(3)
C1	C11	1.492(3)	C17	C18	1.506(3)
C2	C3	1.417(3)	C21	C22	1.523(3)
C3	C4	1.416(3)	C21	C23	1.519(3)
C3	C8	1.422(3)	C21	C24	1.523(3)

Table 5 Bond Angles for 3i.

Atom	Atom	Atom	Angle/˚	Atom	Atom	Atom	Angle/˚
C16	O3	C17	115.46(14)	F1	C12	C13	109.54(14)
C20	O5	C21	120.79(15)	F1	C12	C16	107.08(14)
C20	N1	C15	121.61(16)	C13	C12	C11	111.83(14)
C2	C1	C10	119.43(17)	C13	C12	C16	110.91(15)
C2	C1	C11	123.67(16)	C16	C12	C11	110.04(14)
C10	C1	C11	116.88(16)	C14	C13	C12	113.84(15)
C1	C2	C3	120.85(16)	C15	C14	C13	118.27(16)
C2	C3	C8	119.04(17)	C19	C14	C13	121.06(18)
C4	C3	C2	121.81(17)	C19	C14	C15	120.66(18)
C4	C3	C8	119.15(17)	N1	C15	C14	110.72(15)
C5	C4	C3	120.62(18)	O2	C16	O3	125.37(18)
C4	C5	C6	120.25(18)	O2	C16	C12	124.19(17)
C7	C6	C5	120.06(18)	O3	C16	C12	110.42(15)
C6	C7	C8	121.21(18)	O3	C17	C18	111.64(15)
C7	C8	C3	118.71(18)	O4	C20	O5	126.20(17)
C9	C8	C3	119.07(17)	O4	C20	N1	125.21(18)
C9	C8	C7	122.20(17)	N1	C20	O5	108.58(15)
C10	C9	C8	121.15(18)	O5	C21	C22	102.39(16)
C9	C10	C1	120.41(17)	O5	C21	C23	110.22(16)
O1	C11	C1	121.44(17)	O5	C21	C24	109.74(16)
O1	C11	C12	118.86(16)	C23	C21	C22	110.84(18)
C1	C11	C12	119.66(15)	C23	C21	C24	112.54(17)
F1	C12	C11	107.26(14)	C24	C21	C22	110.64(17)

Table 6 Hydrogen Bonds for 3i.

D	H	A	d(D-H)/Å	d(H-A)/Å	d(D-A)/Å	D-H-A/°
N1	H1	O2[1]	0.88	2.10	2.937(2)	159.8

[1]1+X,+Y,+Z

Table 7 Torsion Angles for 3i.

A	B	C	D	Angle/°	A	B	C	D	Angle/°
F1	C12	C13	C14	58.13(19)	C8	C9	C10	C1	-0.1(3)
F1	C12	C16	O2	4.0(2)	C10	C1	C2	C3	2.4(3)
F1	C12	C16	O3	-177.45(14)	C10	C1	C11	O1	-5.8(3)
O1	C11	C12	F1	134.28(17)	C10	C1	C11	C12	176.26(16)
O1	C11	C12	C13	14.2(2)	C11	C1	C2	C3	-175.98(17)
O1	C11	C12	C16	-109.57(19)	C11	C1	C10	C9	176.58(17)
C1	C2	C3	C4	-179.91(17)	C11	C12	C13	C14	176.89(15)
C1	C2	C3	C8	-0.9(3)	C11	C12	C16	O2	-112.23(19)
C1	C11	C12	F1	-47.7(2)	C11	C12	C16	O3	66.29(19)
C1	C11	C12	C13	-167.80(15)	C12	C13	C14	C15	-74.6(2)
C1	C11	C12	C16	68.5(2)	C12	C13	C14	C19	106.6(2)
C2	C1	C10	C9	-1.9(3)	C13	C12	C16	O2	123.50(19)
C2	C1	C11	O1	172.67(18)	C13	C12	C16	O3	-57.98(19)
C2	C1	C11	C12	-5.3(3)	C13	C14	C15	N1	-51.0(2)
C2	C3	C4	C5	179.29(17)	C15	N1	C20	O4	-6.1(3)
C2	C3	C8	C7	-179.51(17)	C15	N1	C20	O5	175.14(15)
C2	C3	C8	C9	-1.0(3)	C16	O3	C17	C18	-75.4(2)
C3	C4	C5	C6	0.0(3)	C16	C12	C13	C14	-59.86(19)
C3	C8	C9	C10	1.5(3)	C17	O3	C16	O2	-0.2(3)
C4	C3	C8	C7	-0.5(3)	C17	O3	C16	C12	-178.73(14)
C4	C3	C8	C9	177.98(17)	C19	C14	C15	N1	127.79(19)
C4	C5	C6	C7	-0.2(3)	C20	O5	C21	C22	-179.70(16)
C5	C6	C7	C8	0.0(3)	C20	O5	C21	C23	-61.7(2)
C6	C7	C8	C3	0.3(3)	C20	O5	C21	C24	62.8(2)
C6	C7	C8	C9	-178.11(18)	C20	N1	C15	C14	154.18(16)
C7	C8	C9	C10	179.95(18)	C21	O5	C20	O4	-0.7(3)
C8	C3	C4	C5	0.3(3)	C21	O5	C20	N1	178.03(15)

Table 8 Hydrogen Atom Coordinates (Å×10^4) and Isotropic Displacement Parameters (Å^2×10^3) for 3i.

Atom	*x*	*y*	*z*	U(eq)
H1	8491.94	5224.82	6655.51	20
H2	615.18	3898.01	7817.08	18

Table 8 Hydrogen Atom Coordinates (Å×10⁴) and Isotropic Displacement Parameters (Å²×10³) for 3i.

Atom	*x*	*y*	*z*	U(eq)
H4	-2738.08	2883.82	7443.8	21
H5	-5130.57	1431.23	7763.12	24
H6	-4557.51	169.91	9029.17	26
H7	-1600.66	369.74	9951.11	25
H9	1779	1343.08	10310.68	21
H10	4178.19	2778.34	10000.23	21
H13A	6277.38	6618.35	8308.49	18
H13B	6961.06	5328.1	7850.92	18
H15A	4517.7	5530.39	6039.53	20
H15B	5910.79	6635.87	5720.33	20
H17A	1521.07	8667.78	9143.81	27
H17B	-82.49	7785.16	8544.85	27
H18A	770.32	8814.7	7335.89	37
H18B	2430.87	9670.96	7921.81	37
H18C	136.71	9916.84	7961.89	37
H19A	5698.81	8560.93	6478.4	27
H19B	5743.74	8563.76	7513.06	27
H22A	12458.44	2419.07	5783.13	46
H22B	11945.76	869.37	5734.22	46
H22C	11714.14	1698.97	6567.78	46
H23A	8233.03	917.64	6429.17	39
H23B	8316.53	153.06	5556.42	39
H23C	6669.78	1267.92	5598.05	39
H24A	7891.46	2554.58	4386.03	39
H24B	9473.84	1398.5	4340.55	39
H24C	10200.46	2902.37	4484.4	39

Crystal structure determination of 3i

Crystal Data for $C_{24}H_{28}FNO_5$ (M =429.47 g/mol): monoclinic, space group P2$_1$ (no. 4), a = 6.84040(10) Å, b = 10.0817(2) Å, c = 15.9492(3) Å, β = 99.4250(10)°, V = 1085.05(3) Å^3, Z = 2, T = 100.00 K, μ(CuKα) = 0.805 mm^{-1}, $Dcalc$ = 1.315 g/cm^3, 36222 reflections measured (5.616° ≤ 2Θ ≤ 133.254°), 3825 unique (R_{int} = 0.0374, R_{sigma} = 0.0171) which were used in all calculations. The final R_1 was 0.0250 (I > 2σ(I)) and wR_2 was 0.0627 (all data).

References

1. “A Pd-Catalyzed [4 + 2] Annulation Approach to Fluorinated N-Heterocycles”, V. García-Vázquez, L. Hoteite, C. P. Lakeland, D. W. Watson, J. P. A. Harrity, *Org. Lett.,* **2021**, *23*, 2811-2815.
2. (a) W. Wang, H. Shen, X.-L. Wan, Q.-Y. Chen, Y. Guo, *J. Org. Chem.*, **2014**, *79*, 6347-6353; (b) F.-M. Liao, Z.-Y. Cao, J.-S. Yu, J. Zhou, *Angew. Chem. Int. Ed.*, **2017**, *56*, 2459-2463.
